# Aripiprazole in the Maintenance Treatment of Bipolar Disorder: A Critical Review of the Evidence and Its Dissemination into the Scientific Literature

**DOI:** 10.1371/journal.pmed.1000434

**Published:** 2011-05-03

**Authors:** Alexander C. Tsai, Nicholas Z. Rosenlicht, Jon N. Jureidini, Peter I. Parry, Glen I. Spielmans, David Healy

**Affiliations:** 1 Robert Wood Johnson Health and Society Scholars Program, Harvard University, Cambridge, Massachusetts, United States of America; 2 Department of Psychiatry, University of California at San Francisco and San Francisco Veterans Affairs Medical Center, San Francisco, California, United States of America; 3 Discipline of Psychiatry, University of Adelaide, Adelaide, South Australia, Australia; 4 Child and Adolescent Mental Health Service, Division of Mental Health, Flinders University, Adelaide, South Australia, Australia; 5 Department of Psychology, Metropolitan State University, St. Paul, Minnesota, United States of America; 6 Department of Psychological Medicine, Cardiff University, Cardiff, Wales, United Kingdom; University of Western Sydney, Australia

## Abstract

**Background:**

Aripiprazole, a second-generation antipsychotic medication, has been increasingly used in the maintenance treatment of bipolar disorder and received approval from the U.S. Food and Drug Administration for this indication in 2005. Given its widespread use, we sought to critically review the evidence supporting the use of aripiprazole in the maintenance treatment of bipolar disorder and examine how that evidence has been disseminated in the scientific literature.

**Methods and Findings:**

We systematically searched multiple databases to identify double-blind, randomized controlled trials of aripiprazole for the maintenance treatment of bipolar disorder while excluding other types of studies, such as open-label, acute, and adjunctive studies. We then used a citation search to identify articles that cited these trials and rated the quality of their citations. Our evidence search protocol identified only two publications, both describing the results of a single trial conducted by Keck et al., which met criteria for inclusion in this review. We describe four issues that limit the interpretation of that trial as supporting the use of aripiprazole for bipolar maintenance: (1) insufficient duration to demonstrate maintenance efficacy; (2) limited generalizability due to its enriched sample; (3) possible conflation of iatrogenic adverse effects of abrupt medication discontinuation with beneficial effects of treatment; and (4) a low overall completion rate. Our citation search protocol yielded 80 publications that cited the Keck et al. trial in discussing the use of aripiprazole for bipolar maintenance. Of these, only 24 (30%) mentioned adverse events reported and four (5%) mentioned study limitations.

**Conclusions:**

A single trial by Keck et al. represents the entirety of the literature on the use of aripiprazole for the maintenance treatment of bipolar disorder. Although careful review identifies four critical limitations to the trial's interpretation and overall utility, the trial has been uncritically cited in the subsequent scientific literature.

*Please see later in the article for the Editors' Summary*

## Abstract 2

A systematic search of the literature reveals limited evidence to support use of aripiprazole, a second-generation antipsychotic medication, in maintenance therapy of bipolar disorder, despite widespread use.

## Editors' Summary

**Background:**

Bipolar disorder (manic depression) is a serious, long-term mental illness that affects about 1% of adults at some time during their life. It usually develops in late adolescence or early adulthood and affects men and women from all backgrounds. People with bipolar disorder experience wild mood swings that interfere with daily life and damage relationships. During “manic” episodes, which can last several months if untreated, they may feel euphoric (“high”), energetic, or irritable. They may be full of ambitious plans, feel creative, and spend money recklessly. They can also have psychotic symptoms—they may see or hear things that are not there. During depressive episodes, affected individuals may feel helpless, worthless, and suicidal. Treatments for bipolar disorder include drugs to stabilize mood swings (for example, lithium and anticonvulsant medications), antidepressants to treat depressive episodes, and antipsychotic drugs to treat manic episodes. Psychotherapy can also help and patients can be taught to recognize the signs of approaching manic or depressive episodes and the triggers for these episodes.

**Why Was This Study Done?:**

Treatment of bipolar disorder is divided into three phases: acute treatment lasting about 2 months to achieve remission, continuance treatment lasting from months 2 through 6 to prevent relapse, and long-term maintenance treatment to prevent recurrence. Second-generation (atypical) antipsychotics are widely used for acute treatment of manic episodes but are also used for maintenance treatment. For example, the atypical antipsychotic aripiprazole, which gained US approval for this indication in 2005, is now a popular choice among clinicians for treating bipolar disorder. But how much evidence is there to support aripiprazole's use in the maintenance treatment of bipolar disorder? Here, the researchers systematically search the published literature for double-blind randomized controlled trials of aripiprazole for this indication, critically analyze the quality of these trials, and undertake a citation search to investigate how the results of these trials have been disseminated in the scientific literature. In double-blind randomized controlled trials, patients are randomly assigned to receive a test drug or a control (generally, placebo), and the effects of these drugs compared; patients in the trial, and physicians administering treatments, would not know who is receiving the test drug or control until the trial is completed.

**What Did the Researchers Do and Find?:**

The researchers' search for reports of double-blind randomized controlled trials of aripiprazole for the maintenance treatment of bipolar disorder using predefined criteria identified only two publications, both describing a single trial—the Keck trial. Critical review of this trial identified four issues that limit its interpretation for supporting aripiprazole as a maintenance therapy: the trial was too short to demonstrate maintenance efficacy; all the trial participants had responded well to aripiprazole as an acute treatment so the generalizability of the trial's results was limited; the trial design meant that some of the apparent beneficial treatment results could have reflected the adverse effects of abrupt medication discontinuation in the control group; and the trial had a low completion rate. The researchers' citation search identified 80 publications that cited the Keck trial in discussions of the use of aripiprazole for maintenance treatment of bipolar disorder. Only a quarter of these papers presented any numerical data from the trial, only a third mentioned any of the reported adverse events, and only four papers mentioned the trial's limitations.

**What Do These Findings Mean?:**

This evaluation of the evidence base supporting the use of aripiprazole for the maintenance treatment of bipolar disorder shows that the justification for this practice relies on the results of one published trial. Moreover, the methodology and reporting of this trial mean that its results cannot easily be generalized to inform the treatment of most patients with bipolar disorder. Worryingly, the researchers' citation search indicates that the Keck trial has been cited uncritically in the ensuing scientific literature. Although the unique features of bipolar disorder make it hard to undertake controlled studies of treatment options, the researchers express concern that “the publication and apparently uncritical acceptance of this trial may be diverting patients away from more effective treatments”.

**Additional Information:**

Please access these websites via the online version of this summary at http://dx.doi.org/10.1371/journal.pmed.1000434.

- The US National Institute of Mental Health has detailed information on bipolar disorder, including an Easy to Read booklet (in English and Spanish)
- The UK National Health Service Choices website provides information on all aspects of bipolar disorder
- The UK charity Mind has information on bipolar disorder and provides links to other useful organizations
- MedlinePlus has links to further information on bipolar disorder (in English and Spanish)

## Introduction

First-generation antipsychotic medications have been used for many decades in the short-term treatment of acute manic episodes associated with bipolar disorder [1]. Second-generation antipsychotic medications have increasingly gained popularity for this use as well [2]. However, their promotion for the maintenance treatment of bipolar disorder is a more recent phenomenon [3]–[6]. In one recently published nationally representative survey of physicians, mood disorders accounted for the majority of antipsychotic medication prescriptions [7], and a recent shift to prescription of antipsychotic medications was observed in a sample of San Diego county Medicaid beneficiaries with bipolar disorder [8].

Traditionally, the clinical care of patients diagnosed with bipolar disorder has been divided into three phases (borrowed from clinical consensus about the phases of treatment for major depressive disorder [9],[10]): treatment of acute episodes to symptomatic remission, continuation treatment to prevent relapse, and maintenance treatment to prevent recurrence. The 2 mo following recovery from the acute episode is commonly described as acute phase recovery, and the continuation phase of treatment (during which the natural course of the episode is considered still active even though the patient may be asymptomatic) is defined as lasting from months 2 through 6 [11],[12]. The medication used for treatment in the acute phase is often extended for treatment in the continuation and maintenance phases [13],[14] and in this context may include lithium, valproate, lamotrigine, or a second-generation antipsychotic medication such as olanzapine, aripiprazole, quetiapine, risperidone, or ziprasidone [15]. However, although the use of second-generation antipsychotic medications to treat acute mania is supported by a relatively well-established evidence base [16]–[18], efficacy in treatment of acute mania does not necessarily imply efficacy for maintenance or prophylaxis [13],[19],[20]. As Goodwin and Jamison note: “Simply because a drug has anti-manic properties (and if continued, will protect against relapse back into mania in the months after the acute episode), one cannot assume that it will be effective in the prevention of new episodes. While this assumption may be true (to some extent) for lithium, it is not well supported by the data with respect to all the other antimanic agents” (p. 800) [21].

Despite the need for robust evidence on the maintenance and/or long-term prophylactic treatment of bipolar disorder, to date very little has been supplied in this regard [4],[15],[22],[23]. There remains little consensus about recommended courses of maintenance or prophylactic treatment, and consequently overall psychopharmacological treatment patterns vary widely [24]–[28]. Aripiprazole, first approved by the U.S. Food and Drug Administration (FDA) for the treatment of schizophrenia in 2002, is the newest of the second-generation antipsychotic medications to have obtained FDA approval for use in bipolar disorder. In 2004 it was approved for the treatment of acute manic and mixed episodes associated with bipolar disorder, and in 2005 it was granted an additional indication for the maintenance treatment of bipolar disorder [29]. Since its approval, aripiprazole has rapidly become a popular choice among clinicians in the maintenance treatment of bipolar disorder. Total U.S. sales for aripiprazole (across all indications) increased from US$1.5 billion in 2005 to US$4 billion in 2009 [30]. In a recent study in which U.S.-based physicians were queried about their preferred pharmacological treatments for schizophrenia and bipolar disorder, only 3% of psychiatrists and 7% of primary care physicians named aripiprazole as their first choice for treating schizophrenia, whereas 23% of psychiatrists and 16% of primary care physicians named aripiprazole as their first choice for treating bipolar disorder [31]. Consistent with this survey, from 2002–2007, the most common indication for the prescription of aripiprazole in office-based practice settings was for bipolar disorder (International Classification of Diseases, Ninth Revision, Clinical Modification [ICD-9-CM] Diagnosis Code 296.0) [32].

In the setting of chronic illnesses such as bipolar disorder, critical appraisal of long-term treatments has important implications for policy making. Overall medication costs for the chronically ill are driven largely by decisions about the ongoing use of prescription medications, rather than by decisions about whether to initiate their use [33]. Spending on prescription medications is the fastest-growing category of the U.S. health care budget [34], further underscoring the need for a rigorous evidence-based approach regarding their prescription and use. Given the rapid adoption and widespread use of aripiprazole in the maintenance treatment of bipolar disorder, we decided to review the scientific data supporting its use in this setting. A secondary aim of this study was to examine the diffusion of this data into the subsequent scientific literature.

## Methods

### Primary Evidence Search

We sought to identify double-blind (i.e., where participants and physicians administering medications were blind to treatment assignment), randomized controlled studies of aripiprazole for the maintenance treatment of bipolar disorder, while also avoiding inadvertent inclusion of acute treatment studies or other study designs. Therefore we required studies to have a duration greater than 4 mo in order to be included in our review, and excluded open-label, acute, and adjunctive studies. We searched for published literature as well as unpublished and ongoing clinical trials, with no language restrictions. The following systematic search strategy was employed to search PubMed: “bipolar disorder”[MeSH Terms] OR (“bipolar”[All Fields] AND “disorder”[All Fields]) OR (“bipolar disorder”[All Fields]) AND (“aripiprazole”[Substance Name] OR “aripiprazole”[All Fields]) AND (“maintenance”[MeSH Terms] OR “maintenance”[All Fields]). We also searched Scopus (including Embase and MEDLINE) using the same search terms (“bipolar disorder” OR “bipolar” AND “disorder” AND “aripiprazole” AND “maintenance”). We also searched ClincalTrials.gov, the Cochrane Central Register of Controlled Trials (Issue 3 of 4, July 2010), and the World Health Organization International Clinical Trials Registry Platform Search Portal using the terms “aripiprazole” and “bipolar.” We did not attempt to contact the manufacturer directly to inquire about possibly unpublished trials, but we screened all listings on the Bristol-Myers Squibb Clinical Trials Disclosure Web site under Clinical Trial Results, Psychiatric Disorders [35]. All searches were conducted in July 2010. And finally, we submitted a request under the U.S. Freedom of Information Act [36] for the supplemental New Drug Application (NDA) filed by the study sponsor to obtain additional labeling for the use of aripiprazole as maintenance therapy in bipolar I disorder [29], and we searched it manually for further reference to other published or unpublished studies.

### Citation Search

We also sought to better understand the influence of the primary evidence on the broader scientific literature. To do this, we used the Web of Science(R) Science Citation Index Expanded to search for articles that cited the primary evidence identified through the evidence search protocol detailed above. Next, we evaluated the articles on how they cited the primary evidence, using criteria similar to those used in a previous study on the quality of news media reports of medication trials [37]. Each of the citing articles was rated on three quality criteria by a single study author (NZR). A 15% random sample of articles (*n = *15) was double-coded independently by another study author (ACT), and the Cohen's kappa coefficient was calculated in order to assess the degree of inter-rater agreement [38]. We chose dichotomous quality ratings to provide conservative estimates of citation quality and in order to limit subjective judgments by the rater. First, articles were screened for any mention of the use of aripiprazole specifically for ongoing, maintenance, or prophylactic treatment of bipolar disorder. If the answer to this question was “yes,” then the article was further rated on the three quality criteria: (1) whether the article reported any quantitative data from the primary evidence (e.g., odds ratios, percentages, or *p*-values); (2) whether the article mentioned any adverse events described in the primary evidence; and (3) whether the article mentioned any limitations of the primary evidence.

Although our citation search protocol was not specifically targeted towards identifying treatment guidelines and review articles on pharmacological treatment strategies in bipolar disorder, we manually highlighted for further discussion those that were identified in the citation search. Our citation search protocol likely underestimates the influence of the primary evidence because we did not also use a database such as Google Scholar that could have also identified guidelines implemented by hospitals, government, or other institutions whose documents in this area have not been published in peer-reviewed journals or indexed in services such as PubMed. However, we chose to highlight treatment guidelines and reviews because they can be particularly influential in shaping prescribing behavior.

## Results

**Figure 1:**
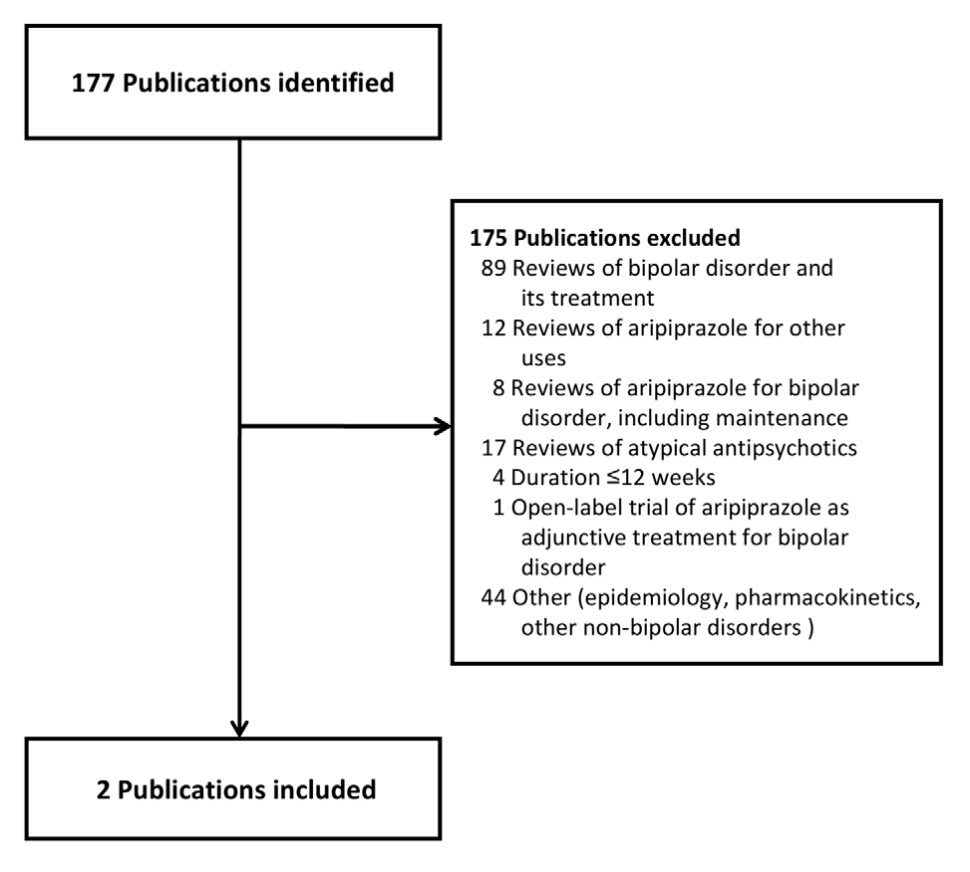
Publications identified for review. These publications were identified using a systematic search of PubMed and Scopus, as well as a manual search of the supplemental new drug application submitted to the FDA to obtain an additional indication for the use of aripiprazole in the maintenance treatment of bipolar disorder.

**Figure 2:**
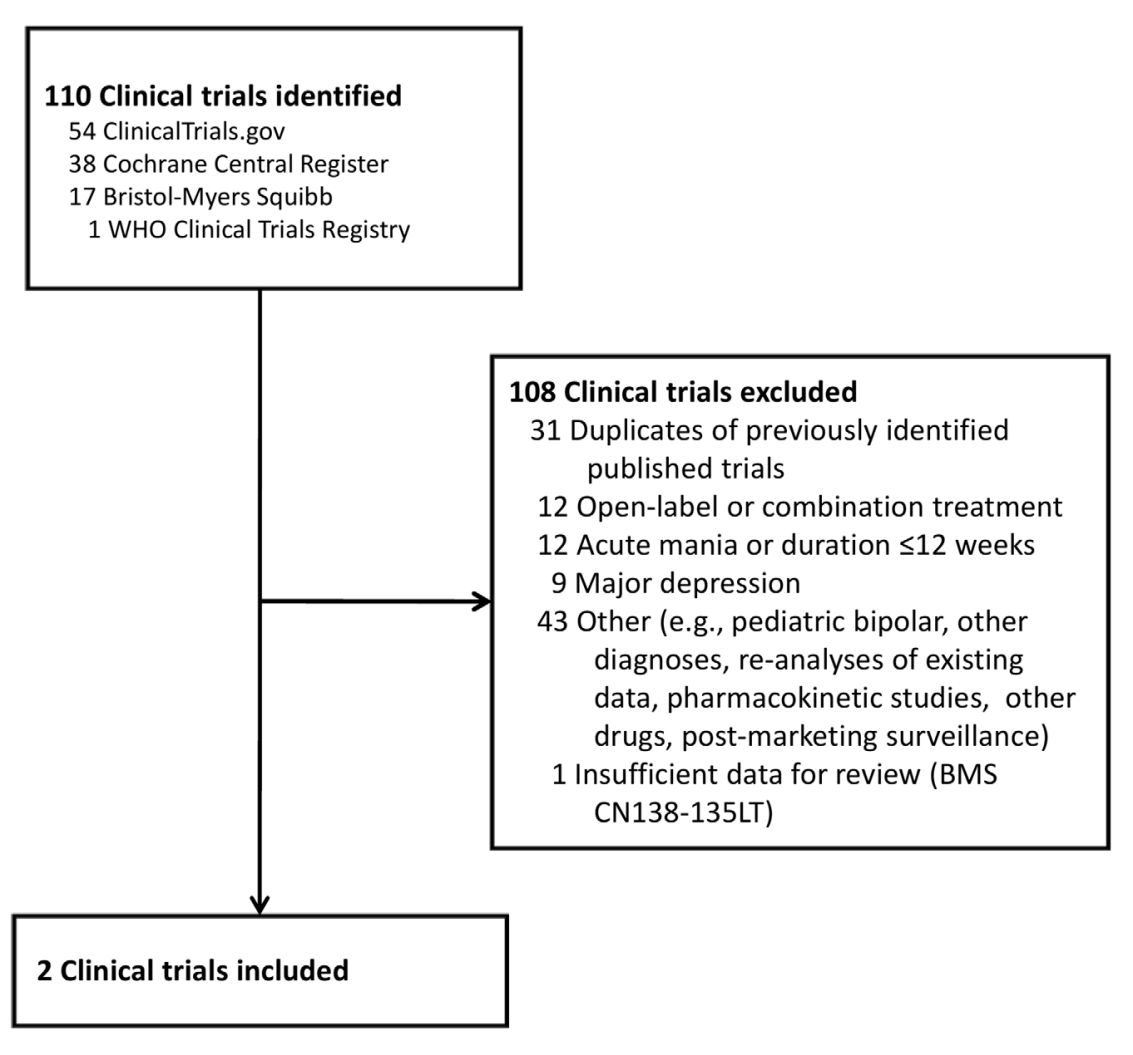
Clinical trials identified for review. These clinical trials were identified using a systematic search of ClinicalTrials.gov, the Cochrane Central Register of Controlled Trials, the World Health Organization (WHO) International Clinical Trials Registry, and the Bristol-Myers Squibb Clinical Trials Disclosure Web site, as well as a manual search of the supplemental new drug application submitted to the FDA to obtain an additional indication for the use of aripiprazole in the maintenance treatment of bipolar disorder. The “duplicates” were each matched to published studies (see Figure 1).

Our primary evidence search protocol identified 177 unique citations (Figure 1). Of these 177 citations, only two publications met criteria for inclusion in our review [39],[40]. Searching the clinical trials registries yielded two listings meeting inclusion criteria, but these referred to the two publications already identified (Figure 2) [39],[40]. Further details on the excluded acute and adjunctive studies are provided in Tables S1 and S2. Two unpublished trials initially appeared to meet criteria for inclusion but were ultimately excluded. The first, Otsuka NCT00606177 [41], was a 3-wk placebo-controlled trial of aripiprazole for treatment of acute mania with a 22-wk extension phase, but it was described as currently still recruiting study participants. The second, BMS CN138-135LT [42], was a completed 40-wk extension of a 12-wk randomized lithium- and placebo-controlled trial of aripiprazole for acute mania. Although the 12-wk acute outcomes data from BMS CN138-135 were published in a peer-reviewed journal [43], the outcomes data from the 40-wk extension have not, to our knowledge, been published (and the little data made available in the synopsis posted online by the manufacturer were inadequate for detailed critical assessment). The manufacturer's synopsis indicates that 4.5% of participants on aripiprazole completed the extension phase, compared to 8.1% for those on lithium. A third arm of the study, completed by 8.5% of participants, entailed treatment with placebo for 3 wk followed by crossover to aripiprazole. Finally, the supplemental NDA contained no references to additional studies, published or unpublished, meeting inclusion criteria (Text S1) [29].

The two peer-reviewed publications included in our review report the results of a single randomized trial (hereafter referred to as “the Keck trial”) implemented under the auspices of the Aripiprazole Study Group and sponsored by the manufacturer of the drug, Bristol-Myers Squibb Co. One publication describes the initial 26-wk double-blind phase [39], and the other its 74-wk extension [40]. We also identified a post hoc subgroup analysis of data from the Keck trial focused on participants diagnosed with the rapid-cycling variant of bipolar disorder [44]. We also identified a separate trial [45], also authored by Keck and colleagues, examining the efficacy of aripiprazole in the treatment of acute manic episodes, with outcomes assessed at 3 wk. Given the paucity of available evidence on aripiprazole for the maintenance treatment of bipolar disorder, we decided to review the Keck trial [39],[40] in detail.

### The Keck Trial

**Figure 3:**
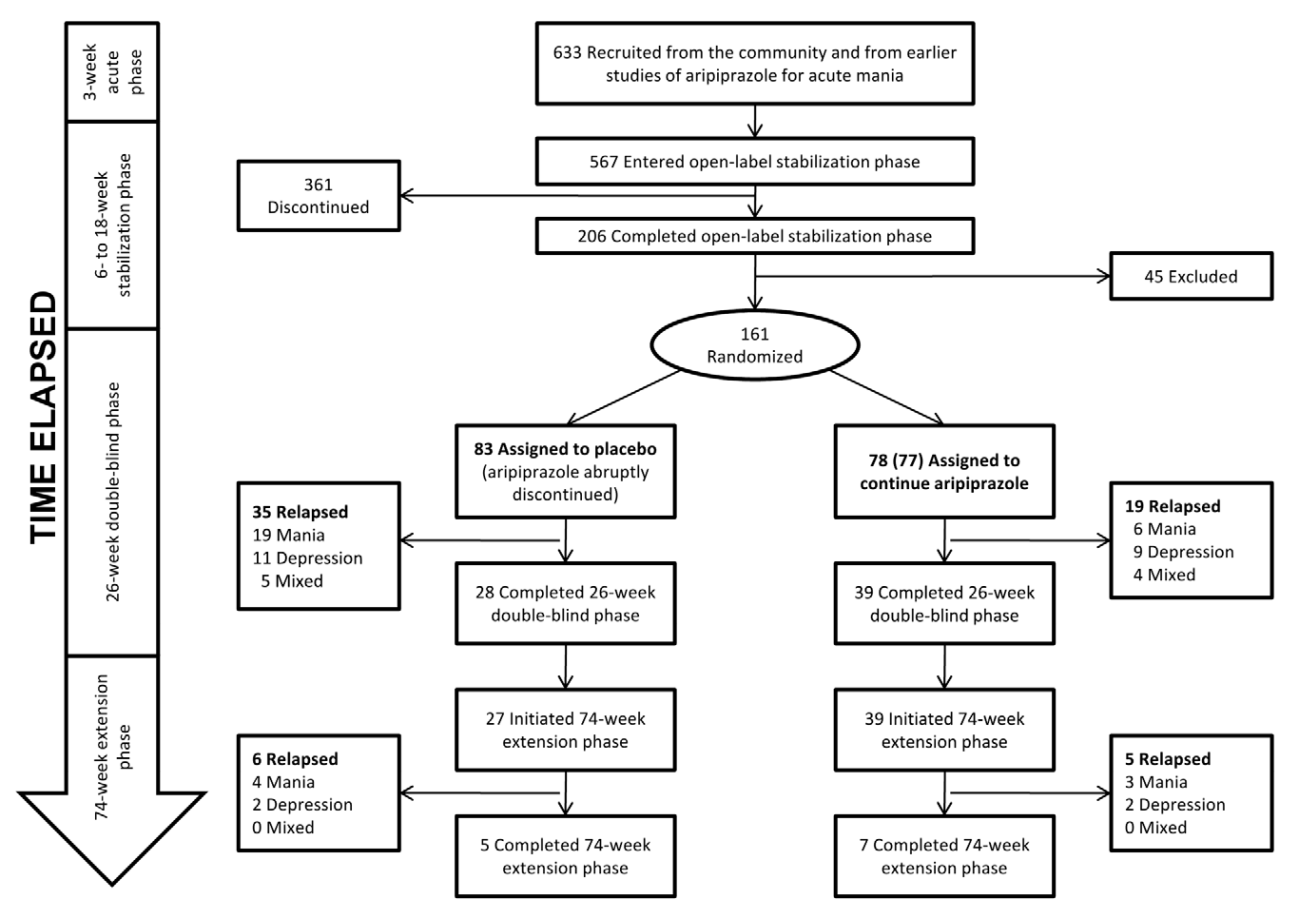
Keck study participant flow. Participants had to complete the 6- to 18-wk stabilization phase before they were eligible for randomization. After completion of the 26-wk double-blind phase, participants were invited to continue in the 74-wk extension phase.

A total of 633 adult participants meeting DSM-IV criteria for bipolar I disorder were enrolled in the Keck trial. A flow chart of the trial is shown in Figure 3. For inclusion, participants must either have completed a prior 3-wk acute mania trial [45], met eligibility criteria for a prior acute mania trial but declined participation in that trial, or experienced a manic or mixed episode within the prior 3 mo. The publication describing the 26-wk double-blind phase [39] indicates that participants were recruited from 76 sites in the U.S., Mexico, and Argentina (but does not specify the numbers of sites within each country or the numbers of patients from each site). Of the original enrollees, 567 entered the “stabilization phase,” which consisted of open-label treatment with aripiprazole for 6–18 wk. Participants remained in this phase until their Young Mania Rating Scale (YMRS) was ≤10 and their Montgomery-Asberg Depression Rating Scale (MADRS) was ≤13 during four consecutive visits over a minimum of 6 wk. 206 participants completed the stabilization phase. Of these, 161 entered the double-blind phase. The supplemental NDA indicates that participants who completed the stabilization phase and entered randomization were derived from 45 sites in the U.S. (*n = *124), three sites in Argentina (*n = *7), and two sites in Mexico (*n = *30). These 161 participants were assigned either to an intervention arm in which they continued taking aripiprazole at the stabilizing dose (*n = *77 or 78; both numbers are reported [40]) or to a placebo arm in which aripiprazole was abruptly discontinued and replaced with placebo (*n = *83). 39 (50% of the 77 or 78 who entered randomization) in the intervention arm and 28 participants (34%) in the placebo arm completed the 26-wk double-blind phase. Time to relapse was described as longer for participants treated with aripiprazole compared to those who were switched to placebo. Mean times to relapse were not provided, but the hazard ratio for relapse was given as 0.52 (95% confidence interval [CI] 0.30–0.91). When time to relapse was partitioned into manic versus depressive relapse, the difference in overall time to relapse was found to be driven primarily by an effect on manic relapse (23% relapse rate on placebo versus 8% relapse rate on aripiprazole). No differences in time to depressive relapse (13% versus 12%) or to mixed state relapse (6% versus 5%) were noted. Keck and colleagues concluded that aripiprazole “was superior to placebo in maintaining efficacy in patients with bipolar I disorder with a recent manic or mixed episode who were stabilized and maintained on aripiprazole treatment for 6 weeks” (p. 626) [39].

The extension phase of the Keck trial, published as a separate paper [40], followed the remaining participants over the subsequent 74 wk: 27 participants in the placebo group (of the 28 who completed the double-blind phase) and 39 participants in the aripiprazole group. The authors concluded: “Over a 100-week treatment period, aripiprazole monotherapy was effective for relapse prevention in patients who were initially stabilized on aripiprazole for 6 consecutive weeks, and it maintained a good safety and tolerability profile” (p. 1480) [40]. Similar to the data from the first 26 wk, time to manic relapse was reported to be longer for the aripiprazole group (with no difference between groups in time to depressive relapse).

### Four Substantive Criticisms of the Keck Trial

Although the Keck trial was the sole basis for aripiprazole receiving an additional FDA-approved indication for the maintenance treatment of bipolar disorder [29], we believe that reading the Keck trial as supporting the use of aripiprazole for this indication is an overinterpretation of the trial's design and the data it generated. First, the duration of the Keck trial was insufficient to demonstrate prophylactic efficacy. Second, the double-blind phase of the Keck trial was based on an enriched sample of patients who had already responded to the medication during the stabilization phase, thereby limiting generalizability of the trial's findings. Third, the randomized discontinuation design of the Keck trial may conflate iatrogenic adverse effects of abrupt medication discontinuation with the beneficial effects of short-term continuation treatment. All of the putative benefit occurred during the double-blind phase of the trial, and little improvement was gained during the extension phase. And finally, the overall completion rate was 1.3%, requiring unrealistic extrapolation to draw meaningful conclusions. Keck et al. [39],[40] mention lack of generalizability as a potential limitation of the enrichment design, but they do not discuss how these other limitations may have compromised the trial's internal validity.

The FDA's review of the Keck trial identified other substantive concerns, including the fact that the *p*-value for the primary analysis changed from 0.02 to 0.10 when the statistical reviewer excluded data from one of the trial's two Mexican sites (where the relapse rate among participants in the aripiprazole arm was lower than the other trial sites) [46]. While of general concern, this and other issues identified by the FDA are unrelated to our methodological critiques. All of these factors undercut even a cautious interpretation of the Keck trial as supporting the use of aripiprazole for maintenance treatment of bipolar disorder. Below we review each of these criticisms in detail.

#### Insufficient duration to demonstrate prophylactic efficacy

In the open-label phase of the Keck trial, stability was defined by whether or not a participant maintained YMRS and MADRS scores in the asymptomatic range for at least 6 consecutive weeks. To meet this criterion, on average the trial participants spent 89 d in the stabilization phase. Comparing their own work to other randomized discontinuation studies of maintenance treatment in bipolar disorder that required a shorter duration of stability [47]–[49], Keck et al. describe their stability criterion as “the most stringent criteria to date to define stability” (p. 634) [39]. Intervention-arm participants who had achieved stability on aripiprazole were then assigned to continue with aripiprazole, and placebo-arm participants abruptly switched to placebo, for the following 26 wk.

Contrary to the authors' claims, we argue here that, given the natural history of bipolar disorder, the design of the Keck trial was unsuitable for evaluating the efficacy of aripiprazole in the maintenance treatment of bipolar disorder. The episodic nature of recurrent mania and depression require investigators to randomize, enroll, and retain patients for a duration sufficient to demonstrate maintenance and/or prophylactic efficacy. While there is high interindividual variation, the median length of untreated episodes has been reported to vary from 3–6 mo in clinical trial settings and from 2–3 mo in epidemiological studies [50], with depressive episodes typically lasting longer than manic episodes [21],[51]. Thus, even if one does not accept the other methodological concerns we describe in this paper, the Keck trial, with its stabilization criterion of 6 wk, could at best be used to demonstrate a short-term benefit of continuation treatment in preventing relapse of symptoms attributable to an ongoing acute episode [52]. Demonstration of maintenance efficacy in preventing recurrence of mood episodes would require benefit to be shown at least 6 mo after the acute phase. 6 mo has been traditionally recognized as the point at which continuation treatment becomes maintenance treatment [10],[11],[12],[52]–[54]. Appropriately, the clinical review contained in the supplemental NDA describes a meeting with the study's sponsors in which the FDA's Division of Neuropharmacological Drug Products “expressed that the duration of the open-label stabilization phase defines duration of effect and noted that an optimal study design would include a six month open-label stabilization phase and randomized withdrawal of patient subgroups at specified timepoints” (p. 9) [55]. The leading textbook in the field suggests an even more stringent threshold study duration: “Because the natural history of bipolar disorder is for it to recur, on average, every 16–18 months, true prophylaxis cannot be evaluated in 6 or 12 months” (p. 801) [21]. Although somewhat controversial, the idea that demonstration of true prophylactic efficacy requires a study duration longer than that which has been typically utilized has been supported by other leading researchers as well [11],[52],[56].

#### Limited generalizability due to the enriched sample

Only participants who had responded to aripiprazole in the stabilization phase of the trial were included in the double-blind phase of the trial. Of the 567 participants who entered the stabilization phase, only 206 completed it. Some of the randomized participants received unblinded medication and were therefore discontinued [29], so the double-blind efficacy dataset consisted of 161 participants. This means that 361 of the 567 (74%) participants who entered the stabilization phase but dropped out were excluded from randomization because of adverse events, lack of efficacy, withdrawal of consent, and other reasons as detailed in the publication—leaving behind a selected group of participants who had responded favorably to aripiprazole in the stabilization phase to be subsequently randomized. This design could have the effect of biasing the trial's findings away from the null [57], and, even in the absence of such bias, the results from this enriched sample cannot be generalized to the majority of persons diagnosed with bipolar disorder. This limitation of the randomized discontinuation design has long been recognized by drug trialists [11],[12],[56],[58]–[65] and is not dissimilar to criticisms voiced about the first generation of randomized trials evaluating the use of lithium for maintenance treatment in bipolar disorder, i.e., that those study designs selected preferentially for lithium-responsive variants of the disorder [66],[67].

#### Possible conflation of iatrogenic effects with beneficial effects

The randomized discontinuation study design could explain the putatively positive findings on preventing relapse even in the absence of a true drug effect. In the Keck trial, the randomization sample was enriched with participants who had already responded to aripiprazole in the stabilization phase and were therefore more likely to experience an iatrogenic relapse of symptoms when aripiprazole was abruptly discontinued in the double-blind phase. Abrupt discontinuation, or even abrupt partial removal, of a drug used for maintenance has long been known to provoke relapse in patients diagnosed with bipolar disorder [68]–[77]. This “bipolar rebound phenomenon” has been most often described for lithium, but it has also been observed in the setting of abruptly withdrawn antiepileptic [78], antipsychotic [3],[12],[48],[56],[78]–[80], and antidepressant medications [59],[75],[78],[81] administered to persons diagnosed with other mood and psychotic disorders. For this reason, Geddes et al. specifically excluded studies with a randomized discontinuation design from their systematic review and meta-analysis of the long-term use of lithium in the treatment of bipolar disorder [82].

Thus, if aripiprazole had no effect on preventing relapse, the Keck trial might still be expected to show a higher relapse rate early in the double-blind phase among participants assigned to the placebo arm (compared to those assigned to the intervention arm), and then similar relapse rates between study arms during the extension phase. This particular design element appears to have substantially influenced the outcome of the Keck trial, as is evident from a comparison of data from the 26-wk double-blind phase with data from the 74-wk extension phase. During the 26-wk double-blind phase, 19 out of 83 participants (23%) in the placebo arm experienced a manic relapse, whereas only four (5%) did so in the subsequent 74-wk extension phase.

**Figure 4:**
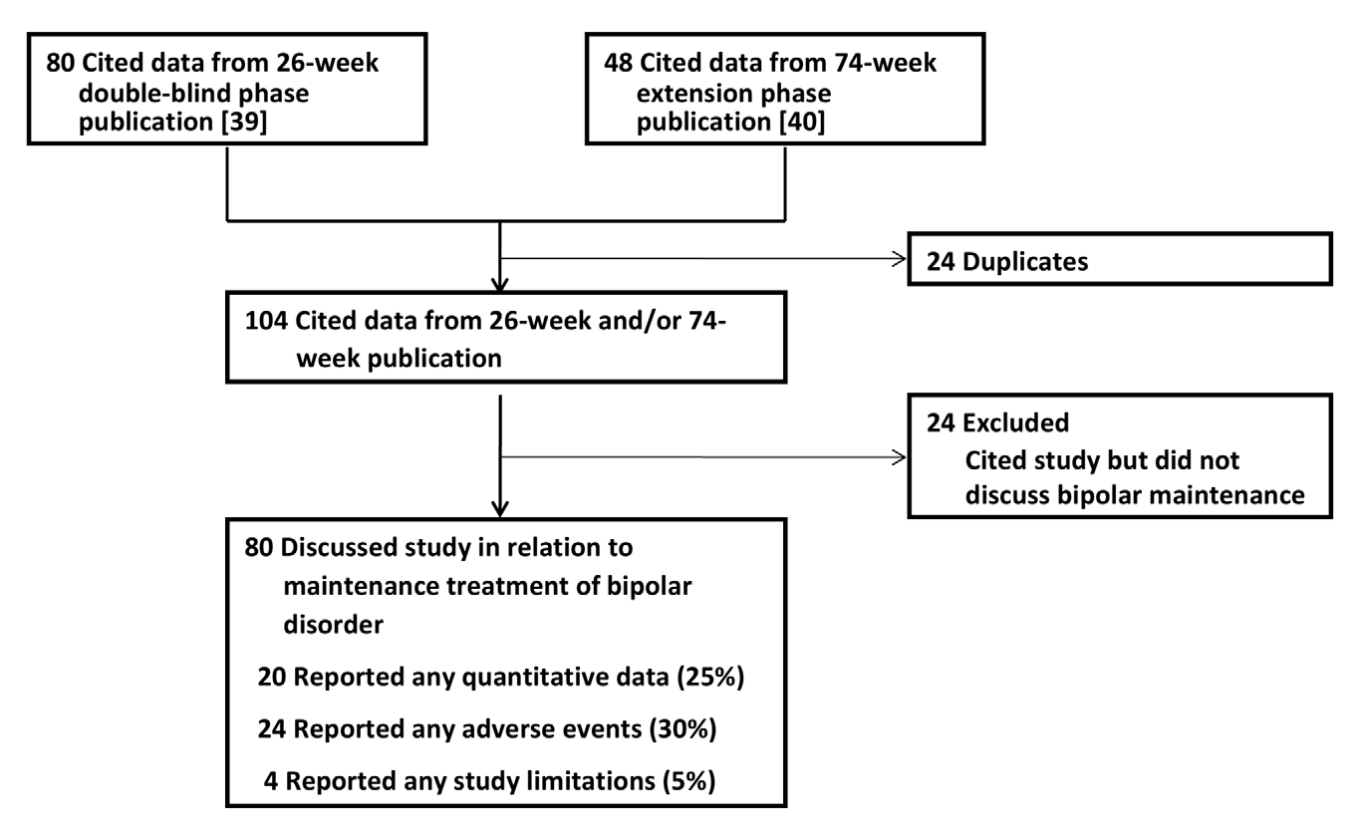
Publications citing the Keck study. These publications were identified using Web of Science(R) Science Citation Index Expanded. Those that discussed the Keck study in relation to the maintenance treatment of bipolar disorder were evaluated on three quality indicators, as shown.

When relapse data from the 74-wk extension phase are examined separately from those from the first 26 wk (Figure 3), only four participants in the placebo arm experienced a relapse to mania, compared to 3 participants in the intervention arm (4.8% versus 3.8%). This information is not explicitly presented in either paper and can only be discerned by comparing the papers side by side and calculating the differences by hand. Figure 4 in the 74-wk extension phase publication (p. 1486) [40] shows that 28% of participants in the placebo arm relapsed to mania over 100 wk of follow up. Given *n = *83 in the placebo arm, this suggests 23 participants in the placebo arm relapsed to mania over 100 wk of follow up. Because 19 participants in the placebo arm relapsed to mania in the first 26 wk (Figure 5 in the 26-wk double-blind phase publication [p. 531] [39]), this means four participants relapsed to mania during the 74-wk extension phase. We employed similar reasoning to calculate the number of participants who relapsed to mania in the intervention arm, as well as the number of participants who relapsed to depressive and mixed states. Similar patterns are observed for relapse to depression and relapse to mixed state for the placebo and aripiprazole arms. Thus, virtually all of the reported placebo-aripiprazole difference in relapse occurred during the first 26 wk of the trial.

#### Limitations of the low completion rate

Only seven of 39 (18%) aripiprazole-treated participants and five of 27 (19%) placebo-treated participants completed the 74-wk extension phase. The low completion rate in the treatment arm is especially striking given that only participants who had proven to be responders in the initial stabilization phase were included in the double-blind and extension phases and that the placebo group matched the aripiprazole group in terms of trial completion. Out of the 633 participants who entered the trial, after excluding the 83 who were switched to placebo, only seven aripiprazole-treated participants completed the 100-wk trial, for a completion rate among aripiprazole-treated participants of less than 1.3%. This is not explicitly noted anywhere in the paper [40]. Keck et al. [40] acknowledge that only 12 participants completed the trial, but the smaller denominator used for comparison is the number of participants who entered the 74-wk extension phase rather than all participants who entered the trial.

We argue that drawing meaningful conclusions from a trial with an overall completion rate of less than 1.3% is an inappropriate undertaking. The completion rate substantially limits generalizability of the trial's findings, as trial completers very likely were dissimilar to the enrolled and/or randomized participant pools. The meaningful differences between completers and noncompleters were demonstrated in a randomized trial of divalproex versus lithium for relapse prevention in bipolar disorder [83]: participants who completed the trial had milder symptoms at baseline and a less severe lifetime illness course [84]. Keck et al. identify the low completion rate in the extension phase as a potential limitation but appeal to the low completion rates observed in other maintenance trials [47],[48] to support the generalizability of their results. In contrast, we view the similar completion rates observed in other long-term studies as similarly raising concerns about how to draw inferences from these trials to inform routine clinical practice. Still, we observe that other investigators have successfully implemented long-term studies in this patient population with greater rates of completion: for example, earlier studies of lithium in the treatment of affective disorders demonstrated completion rates of 92.9% (26/28) [85] and 73.2% (74/101) [86] among lithium-treated participants.

### Impact of the Keck Trial on the Literature

Our citation search protocol identified 80 articles that cited the results from the 26-wk double-blind phase [39] and 48 articles that cited the results of the 74-wk extension phase [40]. After eliminating duplicates, the two publications from the Keck trial garnered 104 subsequent citations in total. Of these citing articles, 24 did not contain any mention of the Keck trial in relation to long-term or maintenance treatment of bipolar disorder and were excluded from further analysis (Figure 4). Double-coding revealed a high degree of inter-rater agreement on the quality assessment measures. There was 100% agreement on whether the publications were classified as mentioning aripiprazole for maintenance treatment. Among the double-coded publications mentioning maintenance treatment, there was 100% agreement on whether quantitative data and limitations were mentioned. There was one disagreement about whether adverse events were mentioned, yielding a kappa coefficient of 0.75 (95% CI 0.05–0.95). The overall kappa coefficient was 0.95 (95% CI 0.73–0.99).

Of the 80 articles that cited the Keck trial in reference to maintenance treatment of bipolar disorder, only 20 (25%) presented any quantitative data from the Keck trial; the remainder reported qualitative statements only (e.g., “Aripiprazole significantly delayed the time to relapse into a new mood episode in patients with bipolar I disorder over both 26 and 100 weeks of treatment.” [87]). 24 publications (30%) mentioned any of the adverse events reported in the trial. Only four (5%) made any mention of study limitations.

Among the articles identified through our citation search protocol were eight literature reviews [88]–[95] and three bipolar treatment guidelines [15],[96],[97] that specifically discussed the use of aripiprazole in the treatment of bipolar disorder. Because review articles and treatment guidelines can be particularly influential in shaping policy and prescribing behavior, we chose to highlight these in our discussion (Table 1). The evidence summaries employed the methodologies of consensus panel (*n = *3), narrative review (*n = *6), or systematic review (*n = *2). Ten of the 11 reviews and treatment guidelines contained a financial disclosure related to Bristol-Myers Squibb.

**Table 1:** Treatment guidelines and reviews of aripiprazole for the treatment of bipolar disorder.

Author, Year, Country	Financial Disclosure Related to Bristol-Myers Squibb	Methods	Quality Indicators^a^			Narrative Recommendation
			A	B	C	
Goodwin, 2009, Great Britain [97]	Yes	Consensus panel	No	No	No	“Aripiprazole was more effective than placebo after acute and continuation treatment of mania with aripiprazole: no effect on depression was discernable. Acute withdrawal of the active agent did not produce an excess of early relapse in this study.” **(positive)**
Yatham, 2009, Canada [96]	Yes	Consensus panel	No	No	No	“Given that efficacy was shown primarily for mania, aripiprazole is included as a first-line maintenance treatment for bipolar disorder for the treatment and prevention of mania.” **(positive)**
Suppes, 2005, United States [15]	Yes	Consensus panel	No	No	No	“Aripiprazole is recommended based on a randomized, double-blind, placebo-controlled, 6-month maintenance study in which patients received open-label aripiprazole until stable, then were randomized to either placebo or aripiprazole for the 6-month follow-up.” **(positive)**
Garcia-Amador, 2006, Spain [93]	Yes	Narrative review	Yes	Yes	No	“These data support the decision by the US FDA to approve aripiprazole for the maintenance treatment of bipolar patients, beyond the treatment of acute mania.” **(positive)**
McIntyre, 2007, Canada [91]	Yes	Narrative review	Yes	Yes	No	“Aripiprazole is established as being efficacious in acute mania and for the prevention of manic relapse in BD. Aripiprazole efficacy is confirmed on primary and secondary efficacy parameters.” **(positive)**
Fagiolini, 2008, United States [94]	Yes	Narrative review	Yes	Yes	No	“This 100-week study showed a significantly longer time to relapse with aripiprazole when compared with placebo.” **(positive)**
McIntyre, 2007, Canada [90]	Yes	Narrative review	Yes	Yes	No	“A single, randomized, double-blind, parallel group, placebo-controlled study reported on the safety and efficacy of aripiprazole in preventing relapse of a mood episode in recently manic or mixed episode patients with bipolar I disorder.” **(positive)**
Ulusahin, 2008, Turkey [95]	None disclosed	Narrative review	Yes	Yes	No	“One double-blind, randomized, placebo controlled clinical trial of 100-week aripiprazole monotherapy, which is the longest clinical trial among the trials conducted in bipolar disorder among the second-generation antipsychotics, showed that aripiprazole was effective for relapse prevention in bipolar patients.” **(positive)**
Muzina, 2009, United States [89]	Yes	Narrative review	Yes	Yes	No	“The results from a 100-week study of aripiprazole for the prevention of bipolar I episodes represent the longest maintenance study since early lithium trials and support the use of aripiprazole as maintenance treatment, primarily against manic relapses.” **(positive)**
Fountoulakis, 2009, Greece [88]	Yes	Systematic review	Yes	Yes	Yes	“Recent reviews suggest that aripiprazole is efficacious in acute mania and in the maintenance treatment of bipolar disorder, with a favourable safety and tolerability profile, with minimal propensity for clinically significant weight gain and metabolic disruption.” **(positive)**
McIntyre, 2010, Canada [92]	Yes	Systematic review	Yes	Yes	Yes	“The available evidence supports the efficacy and tolerability of aripiprazole in the maintenance treatment of bipolar disorder.” **(positive)**

a A, reported any quantitative data; B, reported any adverse events; C, reported any study limitations.

BD, bipolar disorder.

Overall, the eight reviews were favorable in their assessment as to the putative efficacy of aripiprazole in the maintenance treatment of bipolar disorder. Solely on the basis of the results of the Keck trial, the Texas Medication Algorithm Project update listed aripiprazole as having “level 2” evidence (out of five levels of quality, with level 1 being the highest-quality) for maintenance treatment of bipolar disorder [15]. The Canadian Network for Mood and Anxiety Treatments and International Society for Bipolar Disorders recommended aripiprazole as first-line maintenance treatment of bipolar disorder, although it is noted that this is “mainly for preventing mania” (p. 235) [96]. This treatment recommendation was based on the Keck trial, along with a 30-wk pediatric bipolar trial that has only been published in abstract form [98]. The British Association for Psychopharmacology (BAP) based its positive endorsement of aripiprazole for relapse prevention solely on the Keck trial [97]. Contrary to the criticisms we described earlier, the BAP guidelines note, “Acute withdrawal of the active agent did not produce an excess of early relapse in this study” (p. 26).

## Discussion

Our evaluation of the evidence base supporting the use of aripiprazole for the maintenance treatment of bipolar disorder reveals that the justification for this practice relies on the results from a single trial by Keck and colleagues published in two peer-reviewed journal articles [39],[40]. The methodology and reporting of the Keck trial are such that the results cannot be generalized to inform the treatment of most patients with bipolar disorder. Published interpretations of the data notwithstanding, in our opinion the Keck trial does not provide data to support the use of aripiprazole in the maintenance treatment of bipolar disorder. This lack of robust evidence of benefit should be weighed against the potential for long-term harms that have been described with other antipsychotic medications [3] and adverse events related to aripiprazole use, including tremor, akathisia, and significant weight gain [39]. Our concern about the critical imitations in this trial is further accentuated by the apparent widespread use of aripiprazole as a first-line agent for the maintenance treatment of bipolar disorder [31],[32].

Although we appreciate that the unique clinical features of bipolar disorder make controlled study extremely difficult [67],[84],[99]–[103], many of the weaknesses we document stem from the use of the randomized discontinuation design. Further study is needed in order to determine whether the problems described in this particular case are also more widely applicable to other continuation or maintenance treatment studies in bipolar disorder. We find unpersuasive the argument that a randomized discontinuation study such as this is valuable because it reflects common clinical practice [104],[105]. The two-arm, parallel randomized controlled trial may yield information that is more clinically useful than the discontinuation design. Under the parallel design, data from all participants (not just those who demonstrated an acute response) would contribute to our understanding of the drug's short- and long-term efficacy: one of two medications (or placebo) would be given to participants in the acute phase, and they would be followed throughout the continuation and maintenance phases (and beyond) to document response to treatment. (This study design, as well as the other study designs we describe, clearly could be used to study nonpharmacological treatments, including evidence-based psychotherapies. However, because this paper has emphasized discussion about pharmacologic treatments, we use the phrase “medication” for simplicity.) A two-arm, parallel randomized controlled trial of sufficient duration would directly answer the substantive research question, “Does aripiprazole treat symptoms to remission and prevent recurrent episodes when given to patients diagnosed with bipolar disorder presenting in a manic or mixed state?” This is clearly different from the question answered by the discontinuation design, “Among patients diagnosed with bipolar presenting in a manic or mixed state who have achieved a reasonable symptomatic improvement after being given aripiprazole, should aripiprazole be continued to maintain the initial improvement?” [106].

The primary disadvantage of the parallel design is that a greater proportion of (acutely ill) study participants would be subjected to placebo for the full duration of the trial, exactly the ethical issue that the discontinuation design was intended to address [107],[108]. Keck et al. [39] stated that they sought to minimize the extent to which stabilized participants were administered placebo. Yet their study could have been modified to diminish its exposure to the weaknesses that we have described above. First, the duration of stability required prior to randomization could be lengthened. One likely cost of this design modification is that the proportion of participants actually randomized would decrease further [109]. A second modification would be to gradually taper the discontinuation of medication among participants randomized to receive placebo. In previously published randomized discontinuation studies, medications administered during the open-label phase were tapered over a period of 2 to 3 wk rather than abruptly discontinued [47],[83]. Greenhouse et al. [59] suggest implementing the taper over several months.

Aside from these modifications to the parallel design, other alternatives have been suggested. Greenhouse et al. [59] proposed an alternative randomization scheme in which study participants are randomized to one of six treatment strategies. In the acute phase of treatment, study participants would receive one of two medications. In the maintenance phase of treatment, study participants would either remain on the medication initiated during the acute phase, be switched (gradually) to the alternative medication, or be switched (gradually) to placebo. This innovative study design would address the substantive research question, “Which treatment strategy is better in controlling and preventing the recurrence of depression?” (p. 318) [59]. A pure prophylactic design has also been recommended [10],[12],[100], in which patients previously diagnosed with a recurrent mood disorder would be enrolled during a medication-free remission period. Then, while participants are in remission, they would be offered one of two medications (or placebo). All participants would be followed in the study for a prespecified duration, and the treatment arms would be compared in terms of time to recurrence of a mood episode. This design would avoid the previously described error of possibly conflating beneficial treatment effects with iatrogenic adverse effects of abrupt medication discontinuation. However, as noted by Goodwin, Whitman, and Ghaemi [12], the failure of the divalproex study by Bowden et al. [83] was partly attributed to its enrollment of participants with low severity of illness [84]—and it was the last study to utilize the lithium-era prophylaxis design.

We recognize that the proposed study designs will be regarded by some as too costly or infeasible. Although some have suggested that a study with selected limitations may be useful in guiding clinical practice [104], we would disagree with this argument. The current “anti-Hippocratic” state of psychopharmacological practice described by Ghaemi [56],[110] raises questions about the extent to which research with substantive limitations is appropriately interpreted with conservative sensibilities. These concerns are borne out in our data. Thomson Reuters Essential Science Indicators(SM) places the 26-wk double-blind phase publication in the top 1%, and the 74-wk extension phase publication in the top 1%–2%, of papers published in the fields of psychiatry and psychology. Thus, by our conservative estimates, the Keck trial could be regarded as relatively influential. More importantly, we found that the Keck trial was cited uncritically by a subsequent generation of authors, through treatment guidelines, reviews, and other publications. All failed to note the consequences of abrupt and premature discontinuation of antimanic medication, especially during the vulnerable continuation period. The uncritical manner in which the Keck trial has been cited is reminiscent of the “echo chamber” effect described by Carey et al. [111] in their assessment of the now-discredited use of gabapentin in the treatment of bipolar disorder. Although the analogy is somewhat limited as there were no reportedly positive double-blind trials examining the use of gabapentin for this indication, we document a similar pattern of uncritical citations of the primary evidence regarding aripiprazole in the maintenance treatment of bipolar disorder.

Of further concern regarding the uncritical citation of the Keck trial's claims is that ten of the 11 treatment guidelines and review articles in our sample contained a financial disclosure related to the drug's manufacturer, Bristol-Myers Squibb Co. Financial conflicts of interest are highly prevalent across a wide range of medical subfields [112], and while they are known to be associated with recommendations in review articles [113], there is no systematic research documenting their influence on clinical practice guideline recommendations [114]. However, financial conflicts of interest have been found to be associated with biased reporting of outcomes in randomized trials [115],[116], which serve as the evidence upon which treatment guideline recommendations are based.

In summary, we provide here a critical appraisal of the available evidence regarding the use of aripiprazole for the maintenance treatment of bipolar disorder. The available evidence consists of a single trial by Keck et al. [39],[40], which is subject to several substantive methodological limitations but has nonetheless been cited uncritically in the ensuing scientific literature. Several alternative modifications or study designs may improve the probability of generating more useful data from studies in this vulnerable patient population to inform the treatment of similar patients in the future. We are concerned that the publication and apparently uncritical acceptance of this trial may be diverting patients away from more effective treatments.

## Supporting Information

Table S1 Published studies excluded from review. These five published studies were not included in the review because they were open-label, examined the use of aripiprazole as adjunctive treatment or for acute mania, or lacked sufficient duration.
(0.03 MB DOC)

Table S2 Clinical trial registry studies excluded from review. These 12 studies were not included in the review because they were open-label, examined the use of aripiprazole as adjunctive treatment or for acute mania, or lacked sufficient duration.
(0.04 MB DOC)

Text S1 Approval package for: 21-436/S-005 & S-008 & 21-713/S-003. Washington (D.C.): Center for Drug Evaluation and Research, U.S. Food and Drug Administration; 2005. This supplemental New Drug Application (sNDA), which provides for the use of aripiprazole as maintenance therapy in bipolar I disorder, was obtained through a U.S. Freedom of Information Act request.
(4.92 MB PDF)

## Abbreviations

CI: confidence interval
NDA: new drug application
