# Supplementary material for: Aripiprazole in the Maintenance Treatment of Bipolar Disorder: A Critical Review of the Evidence and Its Dissemination into the Scientific Literature
Source: PLoS Med. 2011 May 3;8(5):e1000434. doi: 10.1371/journal.pmed.1000434 (PMC3086871; doi:10.1371/journal.pmed.1000434)
Supplement: Table S1 — Published studies excluded from review. These five published studies were not included in the review because they were open-label, examined the use of aripiprazole as adjunctive treatment or for acute mania, or lacked sufficient duration. (0.03 MB DOC) [file pmed.1000434.s001.doc]

**Table S1. Published studies excluded from review.** These 5 published studies were not included in the review because they were open-label, examined the use of aripiprazole as adjunctive treatment or for acute mania, or lacked sufficient duration.

| **Citation** | **Reason for exclusion** |
| --- | --- |
| Keck PE, Orsulak PJ, Cutler AJ, et al. Aripiprazole monotherapy in the treatment of acute bipolar I mania: a randomized, double-blind, placebo- and lithium-controlled study. J Aff Disord 2009 Jan; 112(1‑3): 36-49. Epub 2008 Oct 2. | This was a randomized, placebo- and lithium-controlled study of oral aripiprazole for the treatment of acute mania or mixed episodes, with outcomes assessed at 12 weeks. Participants randomized to the "placebo" arm received oral placebo medication for only 3 weeks initially and then were crossed over to oral aripiprazole for the remaining 9 weeks. Data from these patients were not included in the analyses of efficacy beyond the third week. The 40-week extension outcomes data (CN138-135LT) were not published. |
| Suppes T, Eudicone J, McQuade R, et al. Efficacy and safety of aripiprazole in subpopulations with acute manic or mixed episodes of bipolar I disorder. J Aff Disord 2008 Apr; 107(1-3): 145-154. Epub 2007 Sep 27. | This was a secondary analysis of data pooled from two randomized controlled trials of oral aripiprazole for the treatment of acute mania, with outcomes assessed at 3 weeks. Data from these two trials were previously published as: Sachs G, Sanchez R, Marcus E, et al. Aripiprazole in the treatment of acute manic or mixed episodes in patients with bipolar I disorder: a 3-week placebo-controlled study. J Psychopharmacol 2006;20(4):536-546; and Keck PE, Marcus R, Tourkodimitris S, et al. A placebo-controlled, double-blind study of the efficacy and safety of aripiprazole in patients with acute bipolar mania. Am J Psychiatry 2003;160(9):1651-1658. |
| Vieta E, Owen R, Baudelet C, et al. Assessment of safety, tolerability and effectiveness of adjunctive aripiprazole to lithium/valproate in bipolar mania: a 46-week, open-label extension following a 6-week double-blind study. Curr Med Res Opin 2010 Jun;26(6):1485-1496. | This was a randomized study of oral aripiprazole as adjunctive treatment for acute mania in persons who were partial non-responders to open-label valproate or lithium, with outcomes assessed at 46 weeks. |
| Young AH, Oren DA, Lowy A, et al. Aripiprazole monotherapy in acute mania: 12-week randomised placebo- and haloperidol-controlled study. Br J Psych 2009 Jan; 194(1): 40-48. | This was a randomized study of oral aripiprazole for the treatment of acute mania, with outcomes assessed at 12 weeks. |
| Zimbroff DL, Marcus RN, Manos G, et al. Management of acute agitation in patients with bipolar disorder: efficacy and safety of intramuscular aripiprazole. J Clin Psychopharm 2007 Apr; 27(2): 171-176. | This was a randomized study of intramuscular aripiprazole for the treatment of acute mania, with outcomes assessed at two hours post-injection. |
