# Supplementary material for: Aripiprazole in the Maintenance Treatment of Bipolar Disorder: A Critical Review of the Evidence and Its Dissemination into the Scientific Literature
Source: PLoS Med. 2011 May 3;8(5):e1000434. doi: 10.1371/journal.pmed.1000434 (PMC3086871; doi:10.1371/journal.pmed.1000434)
Supplement: Table S2 — Clinical trial registry studies excluded from review. These 12 studies were not included in the review because they were open-label, examined the use of aripiprazole as adjunctive treatment or for acute mania, or lacked sufficient duration. (0.04 MB DOC) [file pmed.1000434.s002.doc]

**Table S2. Clinical trial registry studies excluded from review.** These 12 studies were not included in the review because they were open-label, examined the use of aripiprazole as adjunctive treatment or for acute mania, or lacked sufficient duration.

| **Citation** | **Reason for exclusion** |
| --- | --- |
| Bristol-Myers Squibb. A multicenter, randomized, double-blind study of aripiprazole and haloperidol in the maintained response to treatment for an acute manic episode. CN138-008ST (2002). | This was a randomized study of oral aripiprazole for the treatment of acute mania, with outcomes assessed at 12 weeks. This study was published as Vieta E, Bourin M, Sanchez R, et al. Effectiveness of aripiprazole v. haloperidol in acute bipolar mania: double-blind, randomised, comparative 12-week trial. B J Psychiatry 2005 sep;187:235-242. |
| Bristol-Myers Squibb. A multicenter, randomized, double-blind, placebo-controlled study of aripiprazole in the treatment of acutely manic patients with bipolar disorder. CN138-077 (2002). | This was a randomized study of oral aripiprazole for the treatment of acute manic or mixed episodes, with outcomes assessed at 3 weeks. This study was terminated early because the study sponsor had obtained sufficient data from other studies to support the safety and efficacy of aripiprazole to support the indication for acute mania. |
| Bristol-Myers Squibb. Study of aripiprazole in the treatment of patients with acute symptoms in bipolar disorder. CN138-074 (2002). | This was a randomized study of oral aripiprazole for the treatment of acute manic or mixed episodes, with outcomes assessed at 3 weeks. This study was published as Sachs G, Sanchez R, Marcus R, et al. Aripiprazole in the treatment of acute manic or mixed episodes in patients with bipolar I disorder: a 3-week placebo-controlled study. J Psychopharmacol 2006 Jul;20(4):536-546. Epub 2006 Jan 9. |
| Keck PE, Marcus R, Tourkodimitris S, et al. A placebo-controlled, double-blind study of the efficacy and safety of aripiprazole in patients with acute bipolar mania. Am J Psychiatry 2003 Sep;160(9):1651-1658. | This was a randomized study of oral aripiprazole for the treatment of acute manic or mixed episodes, with outcomes assessed at 3 weeks. |
| Bristol-Myers Squibb. Aripiprazole in patients with acute mania. CN138-135LT (2004). | This was a randomized study of oral aripiprazole for the treatment of acute mania, with outcomes assessed at 12 weeks. Participants randomized to the initial-placebo arm received oral placebo medication for only 3 weeks initially and then were crossed over to oral aripiprazole for the remaining 9 weeks. Data from these patients were not included in the analyses of efficacy beyond the third week. This study was published as Keck PE, Orsulak PJ, Cutler AJ, et al. Aripiprazole monotherapy in the treatment of acute bipolar I mania: a randomized, double-blind, placebo- and lithium-controlled study. J Aff Disord 2009 Jan; 112(1‑3): 36-49. Epub 2008 Oct 2. The 40-week extension outcomes data were not published, and the manufacturer's synopsis posted online does not provide enough detail for critical evaluation. |
| Bristol-Myers Squibb. Study of aripiprazole in patients with acute bipolar mania. NCT00097266 (2004). | This was a randomized study of oral aripiprazole for the treatment of acute mania, with outcomes assessed at 12 weeks. This study was published as Young AH, Oren DA, Lowy A, et al. Aripiprazole monotherapy in acute mania: 12-week randomised placebo- and haloperidol-controlled study. Br J Psych 2009 Jan; 194(1): 40-48. |
| University Hospitals of Cleveland. Aripiprazole in late life bipolar disorder. NCT00194038 (2005). | This was an open-label, uncontrolled study of oral aripiprazole as adjunctive treatment for patients with bipolar disorder who were partial non-responders to their existing mood stabilizer medication treatment, with outcomes assessed at 12 weeks. This study was published as Sajatovic M, Coconcea N, Ignacio RV, et al. Aripiprazole therapy in 20 older adults with bipolar disorder: a 12-week, open-label trial. J Clin Pychiatry 2008 Jan;69(1):41-46. |
| Vieta E, Bourin M, Sanchez R, et al. Effectiveness of aripiprazole v. haloperidol in acute bipolar mania: double-blind, randomised, comparative 12-week trial. Br J Psychiatry 2005;187(3):235-242. | This was a haloperidol-controlled randomized study of oral aripiprazole for the treatment of acute manic or mixed episodes, with outcomes assessed at 12 weeks. |
| Sachs G, Sanchez R, Marcus R, et al. Aripiprazole in the treatment of acute manic or mixed episodes in patients with bipolar I disorder: a 3-week placebo-controlled study. J Psychopharmacol 2006 Jul;20(4):536-546. Epub 2006 Jan 9. | This was a randomized study of oral aripiprazole for the treatment of acute manic or mixed episodes, with outcomes assessed at 3 weeks. |
| Bristol-Myers Squibb. Efficacy of aripiprazole in combination with lithium or valproate in the treatment of mania in patients with bipolar I disorder partially nonresponsive to valproate or lithium monotherapy (2006). | This was a randomized study of oral aripiprazole as adjunctive treatment for acute mania in persons who were partial non-responders to open-label valproate or lithium, with outcomes assessed at 6 weeks. This study was published as Vieta E, T'joen C, McQuade RD, et al. Efficacy of adjunctive aripiprazole to either valproate or lithium in bipolar mania patients partially nonresponsive to valproate/lithium monotherapy: a placebo-controlled study. Am J Psychiatry 2008 Oct;165(10):1316-1325. Epub 2008 Apr 1. |
| Otsuka Pharmaceutical Co., Ltd. Abilify in Bipolar Disorder for 6 Weeks Treatment Effectiveness (SMART-A). NCT00545142 (2007). | This was a randomized study of oral aripiprazole co-administered with valproic acid as treatment for acute mania, with outcomes assessed at 6 weeks. The study has been completed but has not been published. |
| Otsuka Pharmaceutical Co., Ltd. A multicenter, randomized, double-blind, placebo-controlled, parallel group-comparison trial of aripiprazole in the treatment of patients with bipolar disorder experiencing a manic or mixed episode. NCT00606281 (2008). | This is a randomized study of oral aripiprazole for the treatment of acute manic or mixed episodes, with outcomes assessed at 21 days. This study is currently enrolling participants. |
