# Supplementary material for: Aripiprazole in the Maintenance Treatment of Bipolar Disorder: A Critical Review of the Evidence and Its Dissemination into the Scientific Literature
Source: PLoS Med. 2011 May 3;8(5):e1000434. doi: 10.1371/journal.pmed.1000434 (PMC3086871; doi:10.1371/journal.pmed.1000434)
Supplement: Text S1 — Approval package for: 21-436/S-005 & S-008 & 21-713/S-003. Washington (D.C.): Center for Drug Evaluation and Research, U.S. Food and Drug Administration; 2005. This supplemental New Drug Application (sNDA), which provides for the use of aripiprazole as maintenance therapy in bipolar I disorder, was obtained through a U.S. Freedom of Information Act request. (4.92 MB PDF) [file pmed.1000434.s003.pdf]

CENTER FOR DRUG EVALUATION AND RESEARCH

<u>Approval Package for:</u>

APPLICATION NUMBER:

21-436/S-005 & S-008
& 21-713/S-003

Trade Name:	Abilify Tablets 5, 10, 15, 20, and 30 mg<br>Abilify Oral Solution 1 mg/mL
Generic Name:	aripiprazole
Sponsor:	Otsuka Pharmaceutical Company
Approval Date:	March 01, 2005
Indications:	For the treatment of schizophrenia and longer-term treatment of schizophrenia.<br>For the treatment of acute manic and mixed episodes associated with Bipolar Disorder and the use as maintenance therapy in Bipolar I Disorder.

CENTER FOR DRUG EVALUATION AND RESEARCH

APPLICATION NUMBER:

21-436/S-005 & S-008 & 21-713/S-003

CONTENTS

Reviews / Information Included in this NDA Review.

Approval Letter	X
Approvable Letter	X
Labeling	X
Summary Review	X
Officer/Employee List	
Office Director Memo	
Cross Discipline Team Leader Review	X
Medical Review(s)	X
Chemistry Review(s)	X
Environmental Assessment	
Pharmacology Review(s)	X
Statistical Review(s)	X
Microbiology Review(s)	
Clinical Pharmacology/Biopharmaceutics Review(s)	
Risk Assessment and Risk Mitigation Review(s)	
Proprietary Name Review(s)	
Other Review(s)	
Administrative/Correspondence Document(s)	X

CENTER FOR DRUG EVALUATION AND RESEARCH

APPLICATION NUMBER:

21-436/S-005 & S-008 & 21-713/S-003

APPROVAL LETTER

DEPARTMENT OF HEALTH & HUMAN SERVICES

Public Health Service

Food and Drug Administration
Rockville, MD 20857

NDA 21-436 / S-005, S-008
NDA 21-713 / S-003

Otsuka Maryland Research Institute
Attn: Dr. Kusuma Mallikaarjun
Director, Regulatory Affairs
2440 Research Boulevard
Rockville, Maryland 20850

Dear Dr. Mallikaarjun:

Please refer to your supplemental new drug application (NDA 21-436 / S-005) dated January 28, 2004, received January 30, 2004, submitted under section 505(b) of the Federal Food, Drug, and Cosmetic Act for Abilify® (aripiprazole) Tablets.

We also acknowledge receipt of your submission dated January 3, 2005 and your secure electronic mail transmissions dated January 18, 2005 (2). Your submission of January 3, 2005 constituted a Complete Response to our November 30, 2004 action letter.

Reference is also made to your supplemental new drug application (NDA 21-436 / S-008) dated December 27, 2004, received December 28, 2004, submitted under section 505(b) of the Federal Food, Drug, and Cosmetic Act for Abilify® (aripiprazole) Tablets, and to your supplemental new drug application (NDA 21-713 / S-003) dated February 16, 2005, received February 16, 2005, submitted under section 505(b) of the Federal Food, Drug, and Cosmetic Act for Abilify® (aripiprazole) Oral Solution.

Supplemental new drug application NDA 21-436 / S-005 provides for the use of Abilify® Tablets as maintenance therapy in Bipolar I Disorder; supplemental new drug application NDA 21-713 / S-003 is a labeling supplement to provide for similar use of Abilify® Oral Solution.

We have completed our reviews of these supplemental applications. They are approved effective on the date of this letter for use as recommended in the enclosed agreed-upon labeling text.

Supplemental new drug application NDA 21-436 / S-008 provided for the addition of a statement on cerebrovascular adverse events (CVAEs), reported in ABILIFY clinical studies, to the WARNINGS section of labeling. CVAE Warning language, as agreed to on January 19, 2005 between representatives of your firm and members of this Division, is also included in the enclosed agreed-upon labeling text. S-008 is therefore superseded by the inclusion of this language in the approved labeling for S-005 and S-003.

Pediatric Research Equity Act (PREA) Requirements: Phase 4 Commitment: Partial Waiver, Partial Deferral
All applications for new active ingredients, new dosage forms, new indications, new routes of administration, and new dosing regimens are required to contain an assessment of the safety and effectiveness of the product in pediatric patients unless this requirement is waived or deferred.

We are waiving this requirement entirely for NDA 21-713 / S-003. We are also waiving it for children below the age of 10 years with regard to NDA 21-436 / S-005. We are deferring submission of pediatric studies under PREA for NDA 21-436 / S-005, for children aged 10 to 17 years (children and adolescents), until April 1, 2009. PREA requirements do not apply to NDA 21-436 / S-008.

The deferred pediatric studies required under Section 2 of the Pediatric Research Equity Act (PREA) are considered required postmarketing study commitments. The status of these postmarketing commitments shall be reported annually according to 21 CFR 314.81. The associated commitments are listed below.

1. *Deferred pediatric studies under PREA (NDA 21-436 / S-005).*
   You are required to assess the safety and effectiveness of Abilify as long-term maintenance treatment for bipolar disorder in pediatric patients ages 10 to 17 (children and adolescents).

   Final Report Submission: April 1, 2009

Submit final study reports to this NDA. For administrative purposes, all submissions related to this pediatric postmarketing study commitment, whether submitted to the IND or the NDA, must be clearly designated "**Required Pediatric Study Commitments**".

Pediatric Exclusivity
Please note that Proposed Pediatric Study Requests and Pediatric Written Requests, which apply to pediatric studies conducted under the terms of section 505A of the Federal Food, Drug, and Cosmetic Act, are distinct from, and may need to be developed ***in addition to,*** pediatric studies under PREA as described above. Satisfaction of the requirements in Section 2 of PREA alone may not qualify you for pediatric exclusivity.

Additional Phase 4 Commitments (Clinical):
We remind you of your additional postmarketing commitments, agreed upon in two teleconferences on September 28, 2004 with reference to Supplement S-002 for acute treatment of bipolar I disorder, and confirmed in your submission of January 3, 2005 and your secure emails of January 18, 2005, with reference to S-005. The commitments are summarized below.

2. *Clinical Efficacy and Safety, S-002 and S-005: Adult clinical study to address longer-term efficacy and safety of aripiprazole as add-on therapy in bipolar disorder.*
   You have agreed to submit the results of a clinical study in adults examining the longer-term efficacy and safety of aripiprazole as add-on therapy in bipolar patients currently taking mood stabilizers (e.g., lithium, valproate). Fulfillment of this commitment for S-002 will also fulfill it for S-005.

   Final Report Submission: September 30, 2009

3. *Pharmacology / Toxicology, S-002 and S-005: Juvenile animal toxicity study/ies to support pediatric studies of aripiprazole in bipolar disorder.*
   You have agreed to conduct and submit a juvenile animal study or studies to support pediatric studies of aripiprazole in bipolar disorder. This study will support both S-002 and S-005 when submitted.

   Final Report(s) Submission: June 30, 2006.

4. *Drug-Drug Interaction, S-005: Drug interaction studies with lithium and valproate.*
   You have agreed to conduct and submit drug interaction studies examining the interaction of aripiprazole with lithium and with valproate (two separate studies). These studies will support S-005 as a Phase 4 commitment.

   Final Reports Submission: June 30, 2005.

Submit clinical protocols to your IND for this product. Submit nonclinical protocols and all final study reports to this NDA, including any final reports intended to support clinical efficacy claims or changes in labeling. In addition, under 21 CFR 314.81(b)(2)(vii) and 314.81(b)(2)(viii), you should include a status summary for each commitment in your annual report to this NDA. The status summary should include:

- expected summary completion dates,
- expected final report submission dates,
- any changes in plans since the last annual report,
- and, for clinical studies, the number of patients entered into each study.

All submissions, including supplements, relating to these postmarketing study commitments must be prominently labeled **"Postmarketing Study Protocol"**, **"Postmarketing Study Final Report"**, or **"Postmarketing Study Correspondence**." Please clearly mark all submissions with the supplement number or numbers that they support, for database management purposes.

Labeling
The final printed labeling (FPL) must be identical to the enclosed agreed-upon labeling (text for the package insert).

Please submit an electronic version of the FPL according to the guidance for industry titled *Providing Regulatory Submissions in Electronic Format - NDA*. Alternatively, you may submit 20 paper copies of the FPL as soon as it is available but no more than 30 days after it is printed. Individually mount 15 of the copies on heavy-weight paper or similar material. For administrative purposes, designate this submission **"FPL for approved supplemental NDAs 21-436 / S-005 and 21-713 / S-003.**" Approval of this submission by FDA is not required before the labeling is used.

Introductory Promotional Materials
In addition, please submit three copies of the introductory promotional materials that you propose to use for this product in this indication. Submit all proposed materials in draft or mock-up form, not final print. Send one copy to this division and two copies of both the promotional materials and the package insert(s) directly to:

Division of Drug Marketing, Advertising,
and Communications, HFD-42
Food and Drug Administration
5600 Fishers Lane
Rockville, MD 20857

If you issue a letter communicating important information about this product (i.e., a "Dear Health Care Professional" letter), we request that you submit a copy of the letter to this NDA and a copy to the following address:

MEDWATCH, HFD-410
Food and Drug Administration
5600 Fishers Lane
Rockville, MD 20857

We remind you that you must comply with reporting requirements for an approved NDA (21 CFR 314.80 and 314.81).

If you have any questions, please call Doris J. Bates, Ph.D., Regulatory Project Manager, at 301-594-2850.

Sincerely,

{See appended electronic signature page}

Russell Katz, M.D.
Director
Division of Neuropharmacological Drug Products
Office of Drug Evaluation I
Center for Drug Evaluation and Research

Enclosure: agreed-upon labeling

This is a representation of an electronic record that was signed electronically and this page is the manifestation of the electronic signature.

/s/

Russell Katz
3/1/05 02:25:04 PM

CENTER FOR DRUG EVALUATION AND RESEARCH

APPLICATION NUMBER:

21-436/S-005 & S-008 & 21-713/S-003

<u>APPROVABLE LETTER</u>

DEPARTMENT OF HEALTH & HUMAN SERVICES

Public Health Service

Food and Drug Administration
Rockville, MD 20857

NDA 21-436 / S-005

Otsuka Maryland Research Institute
Attn: Dr. Kusuma Mallikaarjun
Director, Regulatory Affairs
2440 Research Boulevard
Rockville, Maryland 20850

Dear Dr. Mallikaarjun:

Please refer to your supplemental new drug application (sNDA), referenced above, dated January 28, 2004, received January 30, 2004, submitted under section 505(b) of the Federal Food, Drug, and Cosmetic Act for ABILIFY (aripiprazole) Tablets.

We also acknowledge receipt of your submissions dated March 23, 2004, March 24, 2004, March 25, 2004, July 22, 2004, August 27, 2004, and November 5, 2004.

This supplemental application provides for addition of language to product labeling relating to the maintenance of efficacy in the treatment of patients with Bipolar 1 Disorder, with a recent manic or mixed episode, who have been stabilized for at least 6 consecutive weeks on aripiprazole monotherapy.

We have completed our review of this application as amended, and it is approvable. Before this application may be approved, however, you must address the following comments and/or deficiencies:

Efficacy

Although we consider this application approvable, and have included draft labeling with this letter, we have concerns about the strength of the data provided in Study CN138010. In particular, we note that, although the study is a positive study by the protocol specified analysis, this result appears to be driven by the results in Center 093 (Dr. Ignacio Rosales, Mexico City, Mexico). When this center is removed from the primary analysis, statistical significance is lost. We recognize that removing the data from this center from the analysis is, from a strictly statistical perspective, problematic; however, the dependence of the statistical significance of the overall study on the results of this small center, in which the treatment difference differs markedly from that seen in the US centers (in which there are many more patients) raises questions about the reliability of the result.

We therefore ask you to address this concern.

In addition, we note that for a number of patients who discontinued prior to completion (and who were not considered to have met relapse criteria), we do not have a detailed account of the

reasons for their early withdrawal. We acknowledge that you have provided a brief description of the reasons for each patient's early discontinuation, but in a number of cases, these descriptions are not sufficient for us to be able to confirm that the patient did not leave the trial because of worsening disease. The absence of a detailed understanding of the reasons why patients discontinued treatment in the maintenance phase of this study, therefore, in addition to the concerns raised above, also raises questions about the robustness of the result reported.

For this reason, we ask you to re-examine the data to better characterize the reason that each patient (at all centers, not just Center 093) not classified as having met relapse criteria discontinued treatment. Please provide us with a sufficiently detailed description of these reasons (e.g., a brief narrative) so that we may independently examine them (for example, a patient listed as having discontinued because of insomnia might actually have been experiencing clinical worsening of his or her bipolar disorder; further, if patients had a YMRS or MADRS score at the time of early exit, we would be interested in these data, as well as previous scores for such patients). If you determine that additional patients should have been classified as having met relapse criteria, we would expect you to re-analyze the data. In any event, even if you do not re-classify any patients, we are interested to know if any patients discontinued because of evidence of worsening of their condition.

Draft Labeling

In addition to changes related to Study CN138010, you will note that the appended draft labeling includes new WARNING language on the risk of cerebrovascular adverse events (CVAEs) in elderly patients with dementia. We believe that the draft statement represents a fair description of the data you submitted on July 30, 2003 in response to our request of January 30, 2003.

We also note changes in the labeling (package insert) addressing the manufacturer, distributor and marketer of ABILIFY. These changes were approved under supplement S-004 on July 23, 2003, and are included here.

In addition to the changes we have indicated in the attached labeling, all other previous revisions to labeling, as reflected in the most recently approved package insert, must be included. To facilitate review of your submission, please provide a highlighted or marked-up copy that clearly shows all changes. If additional information relating to the safety or effectiveness of this drug becomes available, further revision of the labeling may be required.

Phase 4 Commitments

Prior to final action on this supplement, it is necessary for us to have agreement in writing concerning all pertinent Phase 4 commitments for this application. Our approval action letter for S-002 (acute manic or mixed episodes associated with Bipolar Disorder) included the following Phase 4 commitments which we believe would adequately address post-approval data needs for longer-term treatment as well:

1. Clinical Efficacy and Safety: Adult clinical studies to address efficacy and safety of aripiprazole as add-on therapy in bipolar disorder.
   -You have previously agreed to submit the results of both short and longer-term studies of the efficacy and safety of aripiprazole as add-on therapy in bipolar patients currently taking mood stabilizers (e.g., lithium, valproate).
   -Final report submission for the longer-term study will occur on or before September 30, 2009.
   -We consider this previous commitment adequate to address Phase 4 needs for both S-002 and S-005. No additional Phase 4 commitment is necessary for S-005 if the previously agreed-upon commitment is met.

2. Pharmacology/Toxicology: Juvenile animal toxicity study/ies to support pediatric studies of aripiprazole in bipolar disorder.
   -You have previously agreed to conduct and submit results of a juvenile animal study or studies to support pediatric studies of aripiprazole in bipolar disorder.
   -Final report(s) submission will occur on or before June 30, 2006.
   - We consider this previous commitment adequate to address Phase 4 needs for both S-002 and S-005. No additional Phase 4 commitment is necessary for S-005 if the previously agreed-upon commitment is met.

In addition, we request the following new Phase 4 commitment:

3. Drug-Drug Interaction: Drug interaction studies with lithium and valproate.
   -We are aware that drug interaction studies are underway or recently completed examining the interaction of aripiprazole with lithium and with valproate (two separate studies).
   -Please propose a date or dates for submission of the final study reports.

Chemistry, Manufacturing, and Controls

We note your request for categorical exclusion from the environmental assessment requirements, as per 21 CFR 25.15 (d) and 21 CFR 25.31(a). We have reviewed this request, and it has been found acceptable. A categorical exclusion will be approved at the time of approval of the supplemental NDA.

Promotional Materials (Draft Format)

In addition, please submit three copies of the introductory promotional materials that you propose to use for this product in this indication. Submit all proposed materials in draft or mock-up form, not final print. Send one copy to this Division, and two copies of both the promotional materials and the package insert directly to:

Division of Drug Marketing, Advertising, and Communications, HFD-42
Food and Drug Administration
5600 Fishers Lane
Rockville, MD 20857

Within 10 days after the date of this letter, you are required to amend this application, notify us of your intent to file an amendment, or follow one of your other options under 21 CFR 314.110. If you do not follow one of these options, we will consider your lack of response a request to withdraw the application under 21 CFR 314.65. Any amendment should respond to all the deficiencies listed. We will not process a partial reply as a major amendment nor will the review clock be reactivated until all deficiencies have been addressed.

This product may be considered misbranded under the Federal Food, Drug, and Cosmetic Act if it is marketed for the proposed new indication before approval of this supplemental application.

If you have any questions, please call Doris J. Bates, Regulatory Project Manager, at (301) 594-2850.

Sincerely,

{See appended electronic signature page}

Russell Katz, M.D.
Director
Division of Neuropharmacological Drug Products
Office of Drug Evaluation I
Center for Drug Evaluation and Research

39 Page(s) Withheld

______ Trade Secret / Confidential (b4)

X Draft Labeling (b4)

______ Draft Labeling (b5)

______ Deliberative Process (b5)

This is a representation of an electronic record that was signed electronically and this page is the manifestation of the electronic signature.

/s/

Russell Katz
11/30/04 01:28:59 PM

CENTER FOR DRUG EVALUATION AND RESEARCH

APPLICATION NUMBER:

21-436/S-005 & S-008 & 21-713/S-003

LABELING

Rx only

ABILIFY® (aripiprazole) Tablets
ABILIFY® (aripiprazole) Oral Solution

DESCRIPTION

ABILIFY® (aripiprazole) is a psychotropic drug that is available as tablets and in solution for oral administration. Aripiprazole is 7-[4-[4-(2,3-dichlorophenyl)-1-piperazinyl]butoxy]-3,4-dihydrocarbostyril. The empirical formula is $C_{23}H_{27}Cl_2N_3O_2$ and its molecular weight is 448.38. The chemical structure is:

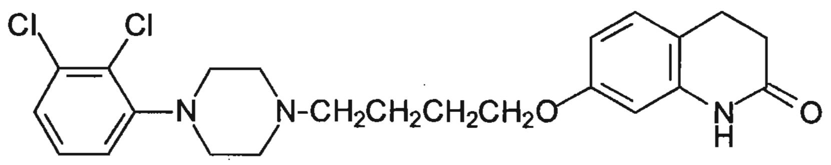

ABILIFY tablets are available in 5-mg, 10-mg, 15-mg, 20-mg, and 30-mg strengths. Inactive ingredients include cornstarch, hydroxypropyl cellulose, lactose monohydrate, magnesium stearate and microcrystalline cellulose. Colorants include ferric oxide (yellow or red) and FD&C Blue No. 2 Aluminum Lake.

ABILIFY is also available as a 1 mg/mL oral solution. The inactive ingredients for this solution include fructose, glycerin, dl-lactic acid, methylparaben, propylene glycol, propylparaben, sodium hydroxide, sucrose, and purified water. The oral solution is flavored with natural orange cream and other natural flavors.

CLINICAL PHARMACOLOGY

Pharmacodynamics

Aripiprazole exhibits high affinity for dopamine D_2 and D_3, serotonin 5-HT_{1A} and 5-HT_{2A} receptors (K_i values of 0.34, 0.8, 1.7, and 3.4 nM, respectively), moderate affinity for dopamine D_4, serotonin 5-HT_{2C} and 5-HT_7, $alpha_1$-adrenergic and histamine H_1

receptors (K_i values of 44, 15, 39, 57, and 61 nM, respectively), and moderate affinity for the serotonin reuptake site (K_i=98 nM). Aripiprazole has no appreciable affinity for cholinergic muscarinic receptors (IC_{50}>1000 nM). Aripiprazole functions as a partial agonist at the dopamine D_2 and the serotonin 5-HT_{1A} receptors, and as an antagonist at serotonin 5-HT_{2A} receptor.

The mechanism of action of aripiprazole, as with other drugs having efficacy in schizophrenia and bipolar disorder, is unknown. However, it has been proposed that the efficacy of aripiprazole is mediated through a combination of partial agonist activity at D_2 and 5-HT_{1A} receptors and antagonist activity at 5-HT_{2A} receptors. Actions at receptors other than D_2, 5-HT_{1A}, and 5-HT_{2A} may explain some of the other clinical effects of aripiprazole, eg, the orthostatic hypotension observed with aripiprazole may be explained by its antagonist activity at adrenergic $alpha_1$ receptors.

Pharmacokinetics

ABILIFY (aripiprazole) activity is presumably primarily due to the parent drug, aripiprazole, and to a lesser extent, to its major metabolite, dehydro-aripiprazole, which has been shown to have affinities for D_2 receptors similar to the parent drug and represents 40% of the parent drug exposure in plasma. The mean elimination half-lives are about 75 hours and 94 hours for aripiprazole and dehydro-aripiprazole, respectively. Steady-state concentrations are attained within 14 days of dosing for both active moieties. Aripiprazole accumulation is predictable from single-dose pharmacokinetics. At steady state, the pharmacokinetics of aripiprazole are dose-proportional. Elimination of aripiprazole is mainly through hepatic metabolism involving two P450 isozymes, CYP2D6 and CYP3A4.

Absorption

Tablet

Aripiprazole is well absorbed after administration of the tablet, with peak plasma concentrations occurring within 3 to 5 hours; the absolute oral bioavailability of the tablet formulation is 87%. ABILIFY can be administered with or without food. Administration of a 15-mg ABILIFY tablet with a standard high-fat meal did not significantly affect the

Cmax or AUC of aripiprazole or its active metabolite, dehydro-aripiprazole, but delayed Tmax by 3 hours for aripiprazole and 12 hours for dehydro-aripiprazole.

Oral Solution

Aripiprazole is well absorbed when administered orally as the solution. At equivalent doses, the plasma concentrations of aripiprazole from the solution were higher than that from the tablet formulation. In a relative bioavailability study comparing the pharmacokinetics of 30 mg aripiprazole as the oral solution to 30 mg aripiprazole tablets in healthy subjects, the solution to tablet ratios of geometric mean Cmax and AUC values were 122% and 114%, respectively (see **DOSAGE AND ADMINISTRATION.**) The single-dose pharmacokinetics of aripiprazole were linear and dose-proportional between the doses of 5 to 30 mg.

Distribution

The steady-state volume of distribution of aripiprazole following intravenous administration is high (404 L or 4.9 L/kg), indicating extensive extravascular distribution. At therapeutic concentrations, aripiprazole and its major metabolite are greater than 99% bound to serum proteins, primarily to albumin. In healthy human volunteers administered 0.5 to 30 mg/day aripiprazole for 14 days, there was dose-dependent D_2 receptor occupancy indicating brain penetration of aripiprazole in humans.

Metabolism and Elimination

Aripiprazole is metabolized primarily by three biotransformation pathways: dehydrogenation, hydroxylation, and N-dealkylation. Based on *in vitro* studies, CYP3A4 and CYP2D6 enzymes are responsible for dehydrogenation and hydroxylation of aripiprazole, and N-dealkylation is catalyzed by CYP3A4. Aripiprazole is the predominant drug moiety in the systemic circulation. At steady state, dehydro-aripiprazole, the active metabolite, represents about 40% of aripiprazole AUC in plasma.

Approximately 8% of Caucasians lack the capacity to metabolize CYP2D6 substrates and are classified as poor metabolizers (PM), whereas the rest are extensive metabolizers (EM). PMs have about an 80% increase in aripiprazole exposure and about a 30% decrease in exposure to the active metabolite compared to EMs, resulting in about a 60% higher exposure to the total active moieties from a given dose of aripiprazole compared to EMs. Coadministration of ABILIFY with known inhibitors of CYP2D6, like

quinidine in EMs, results in a 112% increase in aripiprazole plasma exposure, and dosing adjustment is needed (see **PRECAUTIONS: Drug-Drug Interactions**). The mean elimination half-lives are about 75 hours and 146 hours for aripiprazole in EMs and PMs, respectively. Aripiprazole does not inhibit or induce the CYP2D6 pathway.

Following a single oral dose of [^{14}C]-labeled aripiprazole, approximately 25% and 55% of the administered radioactivity was recovered in the urine and feces, respectively. Less than 1% of unchanged aripiprazole was excreted in the urine and approximately 18% of the oral dose was recovered unchanged in the feces.

Special Populations

In general, no dosage adjustment for ABILIFY is required on the basis of a patient's age, gender, race, smoking status, hepatic function, or renal function (see **DOSAGE AND ADMINISTRATION:** *Dosage in Special Populations*). The pharmacokinetics of aripiprazole in special populations are described below.

Hepatic Impairment

In a single-dose study (15 mg of aripiprazole) in subjects with varying degrees of liver cirrhosis (Child-Pugh Classes A, B, and C), the AUC of aripiprazole, compared to healthy subjects, increased 31% in mild HI, increased 8% in moderate HI, and decreased 20% in severe HI. None of these differences would require dose adjustment.

Renal Impairment

In patients with severe renal impairment (creatinine clearance <30 mL/min), Cmax of aripiprazole (given in a single dose of 15 mg) and dehydro-aripiprazole increased by 36% and 53%, respectively, but AUC was 15% lower for aripiprazole and 7% higher for dehydro-aripiprazole. Renal excretion of both unchanged aripiprazole and dehydro-aripiprazole is less than 1% of the dose. No dosage adjustment is required in subjects with renal impairment.

Elderly

In formal single-dose pharmacokinetic studies (with aripiprazole given in a single dose of 15 mg), aripiprazole clearance was 20% lower in elderly (≥65 years) subjects compared to younger adult subjects (18 to 64 years). There was no detectable age effect, however,

in the population pharmacokinetic analysis in schizophrenia patients. Also, the pharmacokinetics of aripiprazole after multiple doses in elderly patients appeared similar to that observed in young, healthy subjects. No dosage adjustment is recommended for elderly patients (see **PRECAUTIONS: Geriatric Use**).

Gender

Cmax and AUC of aripiprazole and its active metabolite, dehydro-aripiprazole, are 30 to 40% higher in women than in men, and correspondingly, the apparent oral clearance of aripiprazole is lower in women. These differences, however, are largely explained by differences in body weight (25%) between men and women. No dosage adjustment is recommended based on gender.

Race

Although no specific pharmacokinetic study was conducted to investigate the effects of race on the disposition of aripiprazole, population pharmacokinetic evaluation revealed no evidence of clinically significant race-related differences in the pharmacokinetics of aripiprazole. No dosage adjustment is recommended based on race.

Smoking

Based on studies utilizing human liver enzymes *in vitro*, aripiprazole is not a substrate for CYP1A2 and also does not undergo direct glucuronidation. Smoking should, therefore, not have an effect on the pharmacokinetics of aripiprazole. Consistent with these *in vitro* results, population pharmacokinetic evaluation did not reveal any significant pharmacokinetic differences between smokers and nonsmokers. No dosage adjustment is recommended based on smoking status.

Drug-Drug Interactions

Potential for Other Drugs to Affect ABILIFY

Aripiprazole is not a substrate of CYP1A1, CYP1A2, CYP2A6, CYP2B6, CYP2C8, CYP2C9, CYP2C19, or CYP2E1 enzymes. Aripiprazole also does not undergo direct glucuronidation. This suggests that an interaction of aripiprazole with inhibitors or inducers of these enzymes, or other factors, like smoking, is unlikely.

Both CYP3A4 and CYP2D6 are responsible for aripiprazole metabolism. Agents that induce CYP3A4 (eg, carbamazepine) could cause an increase in aripiprazole clearance and lower blood levels. Inhibitors of CYP3A4 (eg, ketoconazole) or CYP2D6 (eg, quinidine, fluoxetine, or paroxetine) can inhibit aripiprazole elimination and cause increased blood levels.

Potential for ABILIFY to Affect Other Drugs

Aripiprazole is unlikely to cause clinically important pharmacokinetic interactions with drugs metabolized by cytochrome P450 enzymes. In *in vivo* studies, 10- to 30-mg/day doses of aripiprazole had no significant effect on metabolism by CYP2D6 (dextromethorphan), CYP2C9 (warfarin), CYP2C19 (omeprazole, warfarin), and CYP3A4 (dextromethorphan) substrates. Additionally, aripiprazole and dehydro-aripiprazole did not show potential for altering CYP1A2-mediated metabolism *in vitro* (see **PRECAUTIONS: Drug-Drug Interactions**).

Aripiprazole had no clinically important interactions with the following drugs:

Famotidine: Coadministration of aripiprazole (given in a single dose of 15 mg) with a 40-mg single dose of the H_2 antagonist famotidine, a potent gastric acid blocker, decreased the solubility of aripiprazole and, hence, its rate of absorption, reducing by 37% and 21% the Cmax of aripiprazole and dehydro-aripiprazole, respectively, and by 13% and 15%, respectively, the extent of absorption (AUC). No dosage adjustment of aripiprazole is required when administered concomitantly with famotidine.

Valproate: When valproate (500-1500 mg/day) and aripiprazole (30 mg/day) were coadministered at steady state, the Cmax and AUC of aripiprazole were decreased by 25%. No dosage adjustment of aripiprazole is required when administered concomitantly with valproate.

Lithium: A pharmacokinetic interaction of aripiprazole with lithium is unlikely because lithium is not bound to plasma proteins, is not metabolized, and is almost entirely excreted unchanged in urine. Coadministration of therapeutic doses of lithium (1200-1800 mg/day) for 21 days with aripiprazole (30 mg/day) did not result in clinically significant changes in the pharmacokinetics of aripiprazole or its active metabolite, dehydro-aripiprazole (Cmax and AUC increased by less than 20%). No dosage adjustment of aripiprazole is required when administered concomitantly with lithium.

Dextromethorphan: Aripiprazole at doses of 10 to 30 mg per day for 14 days had no effect on dextromethorphan's O-dealkylation to its major metabolite, dextrorphan, a pathway known to be dependent on CYP2D6 activity. Aripiprazole also had no effect on dextromethorphan's N-demethylation to its metabolite 3-methyoxymorphan, a pathway known to be dependent on CYP3A4 activity. No dosage adjustment of dextromethorphan is required when administered concomitantly with aripiprazole.

Warfarin: Aripiprazole 10 mg per day for 14 days had no effect on the pharmacokinetics of R- and S-warfarin or on the pharmacodynamic end point of International Normalized Ratio, indicating the lack of a clinically relevant effect of aripiprazole on CYP2C9 and CYP2C19 metabolism or the binding of highly protein-bound warfarin. No dosage adjustment of warfarin is required when administered concomitantly with aripiprazole.

Omeprazole: Aripiprazole 10 mg per day for 15 days had no effect on the pharmacokinetics of a single 20-mg dose of omeprazole, a CYP2C19 substrate, in healthy subjects. No dosage adjustment of omeprazole is required when administered concomitantly with aripiprazole.

Clinical Studies

Schizophrenia

The efficacy of ABILIFY in the treatment of schizophrenia was evaluated in four short-term (4- and 6-week), placebo-controlled trials of acutely relapsed inpatients who predominantly met DSM-III/IV criteria for schizophrenia. Three of the four trials were able to distinguish aripiprazole from placebo, but one study, the smallest, did not. Three of these studies also included an active control group consisting of either risperidone (one trial) or haloperidol (two trials), but they were not designed to allow for a comparison of ABILIFY and the active comparators.

In the three positive trials for ABILIFY, four primary measures were used for assessing psychiatric signs and symptoms. The Positive and Negative Syndrome Scale (PANSS) is a multi-item inventory of general psychopathology used to evaluate the effects of drug treatment in schizophrenia. The PANSS positive subscale is a subset of items in the PANSS that rates seven positive symptoms of schizophrenia (delusions, conceptual disorganization, hallucinatory behavior, excitement, grandiosity, suspiciousness/persecution, and hostility). The PANSS negative subscale is a subset of

items in the PANSS that rates seven negative symptoms of schizophrenia (blunted affect, emotional withdrawal, poor rapport, passive apathetic withdrawal, difficulty in abstract thinking, lack of spontaneity/flow of conversation, stereotyped thinking). The Clinical Global Impression (CGI) assessment reflects the impression of a skilled observer, fully familiar with the manifestations of schizophrenia, about the overall clinical state of the patient.

In a 4-week trial (n=414) comparing two fixed doses of ABILIFY (15 or 30 mg/day) and haloperidol (10 mg/day) to placebo, both doses of ABILIFY were superior to placebo in the PANSS total score, PANSS positive subscale, and CGI-severity score. In addition, the 15-mg dose was superior to placebo in the PANSS negative subscale.

In a 4-week trial (n=404) comparing two fixed doses of ABILIFY (20 or 30 mg/day) and risperidone (6 mg/day) to placebo, both doses of ABILIFY were superior to placebo in the PANSS total score, PANSS positive subscale, PANSS negative subscale, and CGI-severity score.

In a 6-week trial (n=420) comparing three fixed doses of ABILIFY (10, 15, or 20 mg/day) to placebo, all three doses of ABILIFY were superior to placebo in the PANSS total score, PANSS positive subscale, and the PANSS negative subscale.

In a fourth study, a 4-week trial (n=103) comparing ABILIFY in a range of 5 to 30 mg/day or haloperidol 5 to 20 mg/day to placebo, haloperidol was superior to placebo, in the Brief Psychiatric Rating Scale (BPRS), a multi-item inventory of general psychopathology traditionally used to evaluate the effects of drug treatment in psychosis, and in a responder analysis based on the CGI-severity score, the primary outcomes for that trial. ABILIFY was only significantly different compared to placebo in a responder analysis based on the CGI-severity score.

Thus, the efficacy of 15-mg, 20-mg, and 30-mg daily doses was established in two studies for each dose, whereas the efficacy of the 10-mg dose was established in one study. There was no evidence in any study that the higher dose groups offered any advantage over the lowest dose group.

An examination of population subgroups did not reveal any clear evidence of differential responsiveness on the basis of age, gender, or race.

A longer-term trial enrolled 310 inpatients or outpatients meeting DSM-IV criteria for schizophrenia who were, by history, symptomatically stable on other

antipsychotic medications for periods of 3 months or longer. These patients were discontinued from their antipsychotic medications and randomized to ABILIFY 15 mg or placebo for up to 26 weeks of observation for relapse. Relapse during the double-blind phase was defined as CGI-Improvement score of ≥5 (minimally worse), scores ≥5 (moderately severe) on the hostility or uncooperativeness items of the PANSS, or ≥20% increase in the PANSS total score. Patients receiving ABILIFY 15 mg experienced a significantly longer time to relapse over the subsequent 26 weeks compared to those receiving placebo.

Bipolar Disorder

The efficacy of ABILIFY in the treatment of acute manic episodes was established in two 3-week, placebo-controlled trials in hospitalized patients who met the DSM-IV criteria for Bipolar I Disorder with manic or mixed episodes (in one trial, 21% of placebo and 42% of ABILIFY-treated patients had data beyond two weeks). These trials included patients with or without psychotic features and with or without a rapid-cycling course.

The primary instrument used for assessing manic symptoms was the Young Mania Rating Scale (Y-MRS), an 11-item clinician-rated scale traditionally used to assess the degree of manic symptomatology (irritability, disruptive/aggressive behavior, sleep, elevated mood, speech, increased activity, sexual interest, language/thought disorder, thought content, appearance, and insight) in a range from 0 (no manic features) to 60 (maximum score). A key secondary instrument included the Clinical Global Impression - Bipolar (CGI-BP) scale.

In the two positive, 3-week, placebo-controlled trials (n=268; n=248) which evaluated ABILIFY 15 or 30 mg/day, once daily (with a starting dose of 30 mg/day), ABILIFY was superior to placebo in the reduction of Y-MRS total score and CGI-BP Severity of Illness score (mania).

A trial was conducted in patients meeting DSM-IV criteria for Bipolar I Disorder with a recent manic or mixed episode who had been stabilized on open-label ABILIFY and who had maintained a clinical response for at least 6 weeks. The first phase of this trial was an open-label stabilization period in which inpatients and outpatients were clinically stabilized and then maintained on open-label ABILIFY (15 or 30 mg/day, with a starting dose of 30 mg/day) for at least 6 consecutive weeks. One hundred sixty-one outpatients were then randomized in a double-blind fashion, to either the same dose of ABILIFY they were on at the end of the stabilization and maintenance period or placebo

and were then monitored for manic or depressive relapse. During the randomization phase, ABILIFY was superior to placebo on time to the number of combined affective relapses (manic plus depressive), the primary outcome measure for this study. The majority of these relapses were due to manic rather than depressive symptoms. There is insufficient data to know whether Abilify is effective in delaying the time to occurrence of depression in patients with Bipolar I Disorder.

An examination of population subgroups did not reveal any clear evidence of differential responsiveness on the basis of age and gender; however, there were insufficient numbers of patients in each of the ethnic groups to adequately assess inter-group differences.

INDICATIONS AND USAGE

Schizophrenia

ABILIFY is indicated for the treatment of schizophrenia. The efficacy of ABILIFY in the treatment of schizophrenia was established in short-term (4- and 6-week) controlled trials of schizophrenic inpatients (see **CLINICAL PHARMACOLOGY: Clinical Studies**).

The efficacy of ABILIFY in maintaining stability in patients with schizophrenia who had been symptomatically stable on other antipsychotic medications for periods of 3 months or longer, were discontinued from those other medications, and were then administered ABILIFY 15 mg/day and observed for relapse during a period of up to 26 weeks was demonstrated in a placebo-controlled trial (see **CLINICAL PHARMACOLOGY: Clinical Studies**). The physician who elects to use ABILIFY for extended periods should periodically re-evaluate the long-term usefulness of the drug for the individual patient (see **DOSAGE AND ADMINISTRATION**).

Bipolar Disorder

ABILIFY is indicated for the treatment of acute manic and mixed episodes associated with Bipolar Disorder.

The efficacy of ABILIFY was established in two placebo-controlled trials (3 week) of inpatients with DSM-IV criteria for Bipolar I Disorder who were experiencing an acute manic or mixed episode with or without psychotic features (see **CLINICAL PHARMACOLOGY: Clinical Studies**).

The efficacy of ABILIFY in maintaining efficacy in patients with Bipolar I Disorder with a recent manic or mixed episode who had been stabilized and then maintained for at least 6 weeks, was demonstrated in a double-blind, placebo-controlled trial. Prior to entering the double-blind, randomization phase of this trial, patients were clinically stabilized and maintained their stability for 6 consecutive weeks on ABILIFY. Following this 6-week maintenance phase, patients were randomized to either placebo or ABILIFY and monitored for relapse (see **CLINICAL PHARMACOLOGY: Clinical Studies**). Physicians who elect to use ABILIFY for extended periods, that is, longer than 6-weeks, should periodically re-evaluate the long-term usefulness of the drug for the individual patient (see **DOSAGE AND ADMINISTRATION**).

CONTRAINDICATIONS

ABILIFY is contraindicated in patients with a known hypersensitivity to the product.

WARNINGS

Neuroleptic Malignant Syndrome (NMS)

A potentially fatal symptom complex sometimes referred to as Neuroleptic Malignant Syndrome (NMS) has been reported in association with administration of antipsychotic drugs, including aripiprazole. Two possible cases of NMS occurred during aripiprazole treatment in the premarketing worldwide clinical database. Clinical manifestations of NMS are hyperpyrexia, muscle rigidity, altered mental status, and evidence of autonomic instability (irregular pulse or blood pressure, tachycardia, diaphoresis, and cardiac dysrhythmia). Additional signs may include elevated creatine phosphokinase, myoglobinuria (rhabdomyolysis), and acute renal failure.

The diagnostic evaluation of patients with this syndrome is complicated. In arriving at a diagnosis, it is important to exclude cases where the clinical presentation includes both serious medical illness (eg, pneumonia, systemic infection, etc) and untreated or inadequately treated extrapyramidal signs and symptoms (EPS). Other important considerations in the differential diagnosis include central anticholinergic toxicity, heat stroke, drug fever, and primary central nervous system pathology.

The management of NMS should include: 1) immediate discontinuation of antipsychotic drugs and other drugs not essential to concurrent therapy; 2) intensive symptomatic treatment and medical monitoring; and 3) treatment of any concomitant

serious medical problems for which specific treatments are available. There is no general agreement about specific pharmacological treatment regimens for uncomplicated NMS.

If a patient requires antipsychotic drug treatment after recovery from NMS, the potential reintroduction of drug therapy should be carefully considered. The patient should be carefully monitored, since recurrences of NMS have been reported.

Tardive Dyskinesia

A syndrome of potentially irreversible, involuntary, dyskinetic movements may develop in patients treated with antipsychotic drugs. Although the prevalence of the syndrome appears to be highest among the elderly, especially elderly women, it is impossible to rely upon prevalence estimates to predict, at the inception of antipsychotic treatment, which patients are likely to develop the syndrome. Whether antipsychotic drug products differ in their potential to cause tardive dyskinesia is unknown.

The risk of developing tardive dyskinesia and the likelihood that it will become irreversible are believed to increase as the duration of treatment and the total cumulative dose of antipsychotic drugs administered to the patient increase. However, the syndrome can develop, although much less commonly, after relatively brief treatment periods at low doses.

There is no known treatment for established cases of tardive dyskinesia, although the syndrome may remit, partially or completely, if antipsychotic treatment is withdrawn. Antipsychotic treatment, itself, however, may suppress (or partially suppress) the signs and symptoms of the syndrome and, thereby, may possibly mask the underlying process. The effect that symptomatic suppression has upon the long-term course of the syndrome is unknown.

Given these considerations, ABILIFY should be prescribed in a manner that is most likely to minimize the occurrence of tardive dyskinesia. Chronic antipsychotic treatment should generally be reserved for patients who suffer from a chronic illness that (1) is known to respond to antipsychotic drugs, and (2) for whom alternative, equally effective, but potentially less harmful treatments are not available or appropriate. In patients who do require chronic treatment, the smallest dose and the shortest duration of treatment producing a satisfactory clinical response should be sought. The need for continued treatment should be reassessed periodically.

If signs and symptoms of tardive dyskinesia appear in a patient on ABILIFY, drug discontinuation should be considered. However, some patients may require treatment with ABILIFY despite the presence of the syndrome.

Cerebrovascular Adverse Events, Including Stroke, in Elderly Patients with Dementia

In placebo-controlled clinical studies (two flexible dose and one fixed dose study) of dementia-related psychosis, there was an increased incidence of cerebrovascular adverse events (eg, stroke, transient ischemic attack), including fatalities, in aripiprazole-treated patients (mean age: 84 years; range: 78-88 years). In the fixed-dose study, there was a statistically significant dose response relationship for cerebrovascular adverse events in patients treated with aripiprazole. Aripiprazole is not approved for the treatment of patients with dementia-related psychosis. (See also **PRECAUTIONS:** *Use in Patients with Concomitant Illness: Safety Experience in Elderly Patients with Psychosis Associated with Alzheimer's Disease.*)

Hyperglycemia and Diabetes Mellitus

Hyperglycemia, in some cases extreme and associated with ketoacidosis or hyperosmolar coma or death, has been reported in patients treated with atypical antipsychotics. There have been few reports of hyperglycemia in patients treated with ABILIFY. Although fewer patients have been treated with ABILIFY, it is not known if this more limited experience is the sole reason for the paucity of such reports. Assessment of the relationship between atypical antipsychotic use and glucose abnormalities is complicated by the possibility of an increased background risk of diabetes mellitus in patients with schizophrenia and the increasing incidence of diabetes mellitus in the general population. Given these confounders, the relationship between atypical antipsychotic use and hyperglycemia-related adverse events is not completely understood. However, epidemiological studies which did not include ABILIFY suggest an increased risk of treatment-emergent hyperglycemia-related adverse events in patients treated with the atypical antipsychotics included in these studies. Because ABILIFY was not marketed at the time these studies were performed, it is not known if ABILIFY is associated with this increased risk. Precise risk estimates for hyperglycemia-related adverse events in patients treated with atypical antipsychotics are not available.

Patients with an established diagnosis of diabetes mellitus who are started on atypical antipsychotics should be monitored regularly for worsening of glucose control.

Patients with risk factors for diabetes mellitus (eg, obesity, family history of diabetes) who are starting treatment with atypical antipsychotics should undergo fasting blood glucose testing at the beginning of treatment and periodically during treatment. Any patient treated with atypical antipsychotics should be monitored for symptoms of hyperglycemia including polydipsia, polyuria, polyphagia, and weakness. Patients who develop symptoms of hyperglycemia during treatment with atypical antipsychotics should undergo fasting blood glucose testing. In some cases, hyperglycemia has resolved when the atypical antipsychotic was discontinued; however, some patients required continuation of anti-diabetic treatment despite discontinuation of the suspect drug.

PRECAUTIONS

General

Orthostatic Hypotension

Aripiprazole may be associated with orthostatic hypotension, perhaps due to its α_1-adrenergic receptor antagonism. The incidence of orthostatic hypotension-associated events from five short-term, placebo-controlled trials in schizophrenia (n=926) on ABILIFY included: orthostatic hypotension (placebo 1%, aripiprazole 1.9%), orthostatic lightheadedness (placebo 1%, aripiprazole 0.9%), and syncope (placebo 1%, aripiprazole 0.6%). The incidence of orthostatic hypotension-associated events from short-term, placebo-controlled trials in bipolar mania (n=597) on ABILIFY included: orthostatic hypotension (placebo 0%, aripiprazole 0.7%), orthostatic lightheadedness (placebo 0.5%, aripiprazole 0.5%), and syncope (placebo 0.9%, aripiprazole 0.5%).

The incidence of a significant orthostatic change in blood pressure (defined as a decrease of at least 30 mmHg in systolic blood pressure when changing from a supine to standing position) for aripiprazole was not statistically different from placebo (in schizophrenia: 14% among aripiprazole-treated patients and 12% among placebo-treated patients and in bipolar mania: 3% among aripiprazole-treated patients and 2% among placebo-treated patients).

Aripiprazole should be used with caution in patients with known cardiovascular disease (history of myocardial infarction or ischemic heart disease, heart failure or conduction abnormalities), cerebrovascular disease, or conditions which would predispose patients to hypotension (dehydration, hypovolemia, and treatment with antihypertensive medications).

Seizure

Seizures occurred in 0.1% (1/926) of aripiprazole-treated patients with schizophrenia in short-term, placebo-controlled trials. In short-term, placebo-controlled clinical trials of patients with bipolar mania, 0.3% (2/597) of aripiprazole-treated patients and 0.2% (1/436) of placebo-treated patients experienced seizures. As with other antipsychotic drugs, aripiprazole should be used cautiously in patients with a history of seizures or with conditions that lower the seizure threshold, eg, Alzheimer's dementia. Conditions that lower the seizure threshold may be more prevalent in a population of 65 years or older.

Potential for Cognitive and Motor Impairment

In short-term, placebo-controlled trials of schizophrenia, somnolence was reported in 11% of patients on ABILIFY compared to 8% of patients on placebo; somnolence led to discontinuation in 0.1% (1/926) of patients with schizophrenia on ABILIFY in short-term, placebo-controlled trials. In short-term, placebo-controlled trials of bipolar mania, somnolence was reported in 14% of patients on ABILIFY compared to 7% of patients on placebo, but did not lead to discontinuation of any patients with bipolar mania. Despite the relatively modest increased incidence of somnolence compared to placebo, ABILIFY, like other antipsychotics, may have the potential to impair judgment, thinking, or motor skills. Patients should be cautioned about operating hazardous machinery, including automobiles, until they are reasonably certain that therapy with ABILIFY does not affect them adversely.

Body Temperature Regulation

Disruption of the body's ability to reduce core body temperature has been attributed to antipsychotic agents. Appropriate care is advised when prescribing aripiprazole for patients who will be experiencing conditions which may contribute to an elevation in core body temperature, eg, exercising strenuously, exposure to extreme heat, receiving concomitant medication with anticholinergic activity, or being subject to dehydration.

Dysphagia

Esophageal dysmotility and aspiration have been associated with antipsychotic drug use. Aspiration pneumonia is a common cause of morbidity and mortality in elderly patients, in particular those with advanced Alzheimer's dementia. Aripiprazole and other

antipsychotic drugs should be used cautiously in patients at risk for aspiration pneumonia (see **PRECAUTIONS:** *Use in Patients with Concomitant Illness*).

Suicide

The possibility of a suicide attempt is inherent in psychotic illnesses and bipolar disorder, and close supervision of high-risk patients should accompany drug therapy. Prescriptions for ABILIFY should be written for the smallest quantity consistent with good patient management in order to reduce the risk of overdose.

Use in Patients with Concomitant Illness

Safety Experience in Elderly Patients with Psychosis Associated with Alzheimer's Disease: In a flexible dose (2 to 15 mg/day), 10-week, placebo-controlled study of aripiprazole in elderly patients (mean age: 81.5 years; range: 56 to 95 years) with psychosis associated with Alzheimer's dementia, 4 of 105 patients (3.8%) who received ABILIFY died compared to no deaths among 102 patients who received placebo during or within 30 days after termination of the double-blind portion of the study. Three of the patients (age 92, 91, and 87 years) died following the discontinuation of ABILIFY in the double-blind phase of the study (causes of death were pneumonia, heart failure, and shock). The fourth patient (age 78 years) died following hip surgery while in the double-blind portion of the study. The treatment-emergent adverse events that were reported at an incidence of ≥5% and having a greater incidence than placebo in this study were accidental injury, somnolence, and bronchitis. Eight percent of the ABILIFY-treated patients reported somnolence compared to one percent of placebo patients. In a small pilot, open-label, ascending-dose, cohort study (n=30) in elderly patients with dementia, ABILIFY was associated in a dose-related fashion with somnolence.

The safety and efficacy of ABILIFY in the treatment of patients with psychosis associated with dementia have not been established. If the prescriber elects to treat such patients with ABILIFY, vigilance should be exercised, particularly for the emergence of difficulty swallowing or excessive somnolence, which could predispose to accidental injury or aspiration. (See also **WARNINGS: Cerebrovascular Adverse Events, Including Stroke, in Elderly Patients with Dementia**.)

Clinical experience with ABILIFY in patients with certain concomitant systemic illnesses (see **CLINICAL PHARMACOLOGY: Special Populations:** *Renal Impairment* and *Hepatic Impairment*) is limited.

ABILIFY has not been evaluated or used to any appreciable extent in patients with a recent history of myocardial infarction or unstable heart disease. Patients with these diagnoses were excluded from premarketing clinical studies.

Information for Patients

Physicians are advised to discuss the following issues with patients for whom they prescribe ABILIFY:

Interference with Cognitive and Motor Performance

Because aripiprazole may have the potential to impair judgment, thinking, or motor skills, patients should be cautioned about operating hazardous machinery, including automobiles, until they are reasonably certain that aripiprazole therapy does not affect them adversely.

Pregnancy

Patients should be advised to notify their physician if they become pregnant or intend to become pregnant during therapy with ABILIFY (aripiprazole).

Nursing

Patients should be advised not to breast-feed an infant if they are taking ABILIFY.

Concomitant Medication

Patients should be advised to inform their physicians if they are taking, or plan to take, any prescription or over-the-counter drugs, since there is a potential for interactions.

Alcohol

Patients should be advised to avoid alcohol while taking ABILIFY.

Heat Exposure and Dehydration

Patients should be advised regarding appropriate care in avoiding overheating and dehydration.

Sugar Content

Patients should be advised that each mL of ABILIFY oral solution contains 400 mg of sucrose and 200 mg of fructose.

Drug-Drug Interactions

Given the primary CNS effects of aripiprazole, caution should be used when ABILIFY is taken in combination with other centrally acting drugs and alcohol. Due to its α_1-adrenergic receptor antagonism, aripiprazole has the potential to enhance the effect of certain antihypertensive agents.

Potential for Other Drugs to Affect ABILIFY

Aripiprazole is not a substrate of CYP1A1, CYP1A2, CYP2A6, CYP2B6, CYP2C8, CYP2C9, CYP2C19, or CYP2E1 enzymes. Aripiprazole also does not undergo direct glucuronidation. This suggests that an interaction of aripiprazole with inhibitors or inducers of these enzymes, or other factors, like smoking, is unlikely.

Both CYP3A4 and CYP2D6 are responsible for aripiprazole metabolism. Agents that induce CYP3A4 (eg, carbamazepine) could cause an increase in aripiprazole clearance and lower blood levels. Inhibitors of CYP3A4 (eg, ketoconazole) or CYP2D6 (eg, quinidine, fluoxetine, or paroxetine) can inhibit aripiprazole elimination and cause increased blood levels.

Ketoconazole: Coadministration of ketoconazole (200 mg/day for 14 days) with a 15-mg single dose of aripiprazole increased the AUC of aripiprazole and its active metabolite by 63% and 77%, respectively. The effect of a higher ketoconazole dose (400 mg/day) has not been studied. When concomitant administration of ketoconazole with aripiprazole occurs, aripiprazole dose should be reduced to one-half of its normal dose. Other strong inhibitors of CYP3A4 (itraconazole) would be expected to have similar effects and need similar dose reductions; weaker inhibitors (erythromycin, grapefruit juice) have not been studied. When the CYP3A4 inhibitor is withdrawn from the combination therapy, aripiprazole dose should then be increased.

Quinidine: Coadministration of a 10-mg single dose of aripiprazole with quinidine (166 mg/day for 13 days), a potent inhibitor of CYP2D6, increased the AUC of aripiprazole by 112% but decreased the AUC of its active metabolite, dehydro-

aripiprazole, by 35%. Aripiprazole dose should be reduced to one-half of its normal dose when concomitant administration of quinidine with aripiprazole occurs. Other significant inhibitors of CYP2D6, such as fluoxetine or paroxetine, would be expected to have similar effects and, therefore, should be accompanied by similar dose reductions. When the CYP2D6 inhibitor is withdrawn from the combination therapy, aripiprazole dose should then be increased.

Carbamazepine: Coadministration of carbamazepine (200 mg BID), a potent CYP3A4 inducer, with aripiprazole (30 mg QD) resulted in an approximate 70% decrease in Cmax and AUC values of both aripiprazole and its active metabolite, dehydro-aripiprazole. When carbamazepine is added to aripiprazole therapy, aripiprazole dose should be doubled. Additional dose increases should be based on clinical evaluation. When carbamazepine is withdrawn from the combination therapy, aripiprazole dose should then be reduced.

No clinically significant effect of famotidine, valproate, or lithium was seen on the pharmacokinetics of aripiprazole (see **CLINICAL PHARMACOLOGY: Drug-Drug Interactions**).

Potential for ABILIFY to Affect Other Drugs

Aripiprazole is unlikely to cause clinically important pharmacokinetic interactions with drugs metabolized by cytochrome P450 enzymes. In *in vivo* studies, 10- to 30-mg/day doses of aripiprazole had no significant effect on metabolism by CYP2D6 (dextromethorphan), CYP2C9 (warfarin), CYP2C19 (omeprazole, warfarin), and CYP3A4 (dextromethorphan) substrates. Additionally, aripiprazole and dehydro-aripiprazole did not show potential for altering CYP1A2-mediated metabolism *in vitro* (see **CLINICAL PHARMACOLOGY: Drug-Drug Interactions**).

Alcohol: There was no significant difference between aripiprazole coadministered with ethanol and placebo coadministered with ethanol on performance of gross motor skills or stimulus response in healthy subjects. As with most psychoactive medications, patients should be advised to avoid alcohol while taking ABILIFY.

Carcinogenesis, Mutagenesis, Impairment of Fertility

Carcinogenesis

Lifetime carcinogenicity studies were conducted in ICR mice and in Sprague-Dawley (SD) and F344 rats. Aripiprazole was administered for 2 years in the diet at doses of 1, 3, 10, and 30 mg/kg/day to ICR mice and 1, 3, and 10 mg/kg/day to F344 rats (0.2 to 5 and 0.3 to 3 times the maximum recommended human dose [MRHD] based on mg/m^2, respectively). In addition, SD rats were dosed orally for 2 years at 10, 20, 40, and 60 mg/kg/day (3 to 19 times the MRHD based on mg/m^2). Aripiprazole did not induce tumors in male mice or rats. In female mice, the incidences of pituitary gland adenomas and mammary gland adenocarcinomas and adenoacanthomas were increased at dietary doses of 3 to 30 mg/kg/day (0.1 to 0.9 times human exposure at MRHD based on AUC and 0.5 to 5 times the MRHD based on mg/m^2). In female rats, the incidence of mammary gland fibroadenomas was increased at a dietary dose of 10 mg/kg/day (0.1 times human exposure at MRHD based on AUC and 3 times the MRHD based on mg/m^2); and the incidences of adrenocortical carcinomas and combined adrenocortical adenomas/carcinomas were increased at an oral dose of 60 mg/kg/day (14 times human exposure at MRHD based on AUC and 19 times the MRHD based on mg/m^2).

Proliferative changes in the pituitary and mammary gland of rodents have been observed following chronic administration of other antipsychotic agents and are considered prolactin-mediated. Serum prolactin was not measured in the aripiprazole carcinogenicity studies. However, increases in serum prolactin levels were observed in female mice in a 13-week dietary study at the doses associated with mammary gland and pituitary tumors. Serum prolactin was not increased in female rats in 4- and 13-week dietary studies at the dose associated with mammary gland tumors. The relevance for human risk of the findings of prolactin-mediated endocrine tumors in rodents is unknown.

Mutagenesis

The mutagenic potential of aripiprazole was tested in the *in vitro* bacterial reverse-mutation assay, the *in vitro* bacterial DNA repair assay, the *in vitro* forward gene mutation assay in mouse lymphoma cells, the *in vitro* chromosomal aberration assay in Chinese hamster lung (CHL) cells, the *in vivo* micronucleus assay in mice, and the unscheduled DNA synthesis assay in rats. Aripiprazole and a metabolite (2,3-DCPP)

were clastogenic in the *in vitro* chromosomal aberration assay in CHL cells with and without metabolic activation. The metabolite, 2,3-DCPP, produced increases in numerical aberrations in the *in vitro* assay in CHL cells in the absence of metabolic activation. A positive response was obtained in the *in vivo* micronucleus assay in mice, however, the response was shown to be due to a mechanism not considered relevant to humans.

Impairment of Fertility

Female rats were treated with oral doses of 2, 6, and 20 mg/kg/day (0.6, 2, and 6 times the maximum recommended human dose [MRHD] on a mg/m^2 basis) of aripiprazole from 2 weeks prior to mating through day 7 of gestation. Estrus cycle irregularities and increased corpora lutea were seen at all doses, but no impairment of fertility was seen. Increased pre-implantation loss was seen at 6 and 20 mg/kg, and decreased fetal weight was seen at 20 mg/kg.

Male rats were treated with oral doses of 20, 40, and 60 mg/kg/day (6, 13, and 19 times the MRHD on a mg/m^2 basis) of aripiprazole from 9 weeks prior to mating through mating. Disturbances in spermatogenesis were seen at 60 mg/kg, and prostate atrophy was seen at 40 and 60 mg/kg, but no impairment of fertility was seen.

Pregnancy

Pregnancy Category C

In animal studies, aripiprazole demonstrated developmental toxicity, including possible teratogenic effects in rats and rabbits.

Pregnant rats were treated with oral doses of 3, 10, and 30 mg/kg/day (1, 3, and 10 times the maximum recommended human dose [MRHD] on a mg/m^2 basis) of aripiprazole during the period of organogenesis. Gestation was slightly prolonged at 30 mg/kg. Treatment caused a slight delay in fetal development, as evidenced by decreased fetal weight (30 mg/kg), undescended testes (30 mg/kg), and delayed skeletal ossification (10 and 30 mg/kg). There were no adverse effects on embryofetal or pup survival. Delivered offspring had decreased bodyweights (10 and 30 mg/kg), and increased incidences of hepatodiaphragmatic nodules and diaphragmatic hernia at 30 mg/kg (the other dose groups were not examined for these findings). (A low incidence of diaphragmatic hernia was also seen in the fetuses exposed to 30 mg/kg.) Postnatally, delayed vaginal opening was seen at 10 and 30 mg/kg and impaired reproductive

performance (decreased fertility rate, corpora lutea, implants, and live fetuses, and increased post-implantation loss, likely mediated through effects on female offspring) was seen at 30 mg/kg. Some maternal toxicity was seen at 30 mg/kg, however, there was no evidence to suggest that these developmental effects were secondary to maternal toxicity.

Pregnant rabbits were treated with oral doses of 10, 30, and 100 mg/kg/day (2, 3, and 11 times human exposure at MRHD based on AUC and 6, 19, and 65 times the MRHD based on mg/m^2) of aripiprazole during the period of organogenesis. Decreased maternal food consumption and increased abortions were seen at 100 mg/kg. Treatment caused increased fetal mortality (100 mg/kg), decreased fetal weight (30 and 100 mg/kg), increased incidence of skeletal abnormality (fused sternebrae at 30 and 100 mg/kg) and minor skeletal variations (100 mg/kg).

In a study in which rats were treated with oral doses of 3, 10, and 30 mg/kg/day (1, 3, and 10 times the MRHD on a mg/m^2 basis) of aripiprazole perinatally and postnatally (from day 17 of gestation through day 21 postpartum), slight maternal toxicity and slightly prolonged gestation were seen at 30 mg/kg. An increase in stillbirths, and decreases in pup weight (persisting into adulthood) and survival, were seen at this dose.

There are no adequate and well-controlled studies in pregnant women. It is not known whether aripiprazole can cause fetal harm when administered to a pregnant woman or can affect reproductive capacity. Aripiprazole should be used during pregnancy only if the potential benefit outweighs the potential risk to the fetus.

Labor and Delivery

The effect of aripiprazole on labor and delivery in humans is unknown.

Nursing Mothers

Aripiprazole was excreted in milk of rats during lactation. It is not known whether aripiprazole or its metabolites are excreted in human milk. It is recommended that women receiving aripiprazole should not breast-feed.

Pediatric Use

Safety and effectiveness in pediatric and adolescent patients have not been established.

Geriatric Use

Of the 7951 patients treated with aripiprazole in premarketing clinical trials, 991 (12%) were ≥65 years old and 789 (10%) were ≥75 years old. The majority (88%) of the 991 patients were diagnosed with dementia of the Alzheimer's type.

Placebo-controlled studies of aripiprazole in schizophrenia or bipolar mania did not include sufficient numbers of subjects aged 65 and over to determine whether they respond differently from younger subjects. There was no effect of age on the pharmacokinetics of a single 15-mg dose of aripiprazole. Aripiprazole clearance was decreased by 20% in elderly subjects (≥65 years) compared to younger adult subjects (18 to 64 years), but there was no detectable effect of age in the population pharmacokinetic analysis in schizophrenia patients.

Studies of elderly patients with psychosis associated with Alzheimer's disease have suggested that there may be a different tolerability profile in this population compared to younger patients with schizophrenia (see **PRECAUTIONS:** *Use in Patients with Concomitant Illness*). The safety and efficacy of ABILIFY in the treatment of patients with psychosis associated with Alzheimer's disease has not been established. If the prescriber elects to treat such patients with ABILIFY, vigilance should be exercised.

ADVERSE REACTIONS

Aripiprazole has been evaluated for safety in 7951 patients who participated in multiple-dose, premarketing trials in schizophrenia, bipolar mania, and dementia of the Alzheimer's type, and who had approximately 5235 patient-years of exposure. A total of 2280 aripiprazole-treated patients were treated for at least 180 days and 1558 aripiprazole-treated patients had at least 1 year of exposure.

The conditions and duration of treatment with aripiprazole included (in overlapping categories) double-blind, comparative and noncomparative open-label studies, inpatient and outpatient studies, fixed- and flexible-dose studies, and short- and longer-term exposure.

Adverse events during exposure were obtained by collecting volunteered adverse events, as well as results of physical examinations, vital signs, weights, laboratory analyses, and ECG. Adverse experiences were recorded by clinical investigators using terminology of their own choosing. In the tables and tabulations that follow, modified

COSTART dictionary terminology has been used initially to classify reported adverse events into a smaller number of standardized event categories, in order to provide a meaningful estimate of the proportion of individuals experiencing adverse events.

The stated frequencies of adverse events represent the proportion of individuals who experienced at least once, a treatment-emergent adverse event of the type listed. An event was considered treatment emergent if it occurred for the first time or worsened while receiving therapy following baseline evaluation. There was no attempt to use investigator causality assessments; ie, all reported events are included.

The prescriber should be aware that the figures in the tables and tabulations cannot be used to predict the incidence of side effects in the course of usual medical practice where patient characteristics and other factors differ from those that prevailed in the clinical trials. Similarly, the cited frequencies cannot be compared with figures obtained from other clinical investigations involving different treatment, uses, and investigators. The cited figures, however, do provide the prescribing physician with some basis for estimating the relative contribution of drug and nondrug factors to the adverse event incidence in the population studied.

Adverse Findings Observed in Short-Term, Placebo-Controlled Trials of Patients with Schizophrenia

The following findings are based on a pool of five placebo-controlled trials (four 4-week and one 6-week) in which aripiprazole was administered in doses ranging from 2 to 30 mg/day.

Adverse Events Associated with Discontinuation of Treatment in Short-Term, Placebo-Controlled Trials

Overall, there was no difference in the incidence of discontinuation due to adverse events between aripiprazole-treated (7%) and placebo-treated (9%) patients. The types of adverse events that led to discontinuation were similar between the aripiprazole and placebo-treated patients.

Adverse Findings Observed in Short-Term, Placebo-Controlled Trials of Patients with Bipolar Mania

The following findings are based on a pool of 3-week, placebo-controlled, bipolar mania trials in which aripiprazole was administered at doses of 15 or 30 mg/day.

Adverse Events Associated with Discontinuation of Treatment in Short-Term, Placebo-Controlled Trials

Overall, in patients with bipolar mania, there was no difference in the incidence of discontinuation due to adverse events between aripiprazole-treated (11%) and placebo-treated (9%) patients. The types of adverse events that led to discontinuation were similar between the aripiprazole and placebo-treated patients.

Commonly Observed Adverse Events in Short-Term, Placebo-Controlled Trials of Patients with Bipolar Mania

Commonly observed adverse events associated with the use of aripiprazole in patients with bipolar mania (incidence of 5% or greater and aripiprazole incidence at least twice that for placebo) are shown in Table 1. There were no adverse events in the short-term trials of schizophrenia that met these criteria.

Table 1: Commonly Observed Adverse Events in Short-Term, Placebo-Controlled Trials of Patients with Bipolar Mania

	Percentage of Patients Reporting Event	
	**Aripiprazole**	**Placebo**
**Adverse Event**	**(n=597)**	**(n=436)**
Accidental Injury	6	3
Constipation	13	6
Akathisia	15	4

Adverse Events Occurring at an Incidence of 2% or More Among Aripiprazole-Treated Patients and Greater than Placebo in Short-Term, Placebo-Controlled Trials

Table 2 enumerates the pooled incidence, rounded to the nearest percent, of treatment-emergent adverse events that occurred during acute therapy (up to 6 weeks in schizophrenia and up to 3 weeks in bipolar mania), including only those events that occurred in 2% or more of patients treated with aripiprazole (doses ≥2 mg/day) and for which the incidence in patients treated with aripiprazole was greater than the incidence in patients treated with placebo in the combined dataset.

Table 2: Treatment-Emergent Adverse Events in Short-Term, Placebo-Controlled Trials

	Percentage of Patients Reporting Event[a]	
**Body System** / **Adverse Event**	**Aripiprazole (n=1523)**	**Placebo (n=849)**
**Body as a Whole**		
Headache	31	26
Asthenia	8	7
Accidental Injury	5	4
Peripheral Edema	2	1
**Cardiovascular System**		
Hypertension	2	1
**Digestive System**		
Nausea	16	12
Dyspepsia	15	13
Vomiting	11	6
Constipation	11	7
**Musculoskeletal System**		
Myalgia	4	3
**Nervous System**		
Agitation	25	24
Anxiety	20	17
Insomnia	20	15
Somnolence	12	8
Akathisia	12	5
Lightheadedness	11	8
Extrapyramidal Syndrome	6	4
Tremor	4	3
Increased Salivation	3	1
**Respiratory System**		
Pharyngitis	4	3
Rhinitis	4	3
Coughing	3	2
**Special Senses**		
Blurred Vision	3	1

[a] Events reported by at least 2% of patients treated with aripiprazole, except the following events, which had an incidence equal to or less than placebo: abdominal pain, back pain, dental pain, diarrhea, dry mouth, anorexia, psychosis, hypertonia, upper respiratory tract infection, rash, vaginitis[f], dysmenorrhea[f].

[f] Percentage based on gender total.

An examination of population subgroups did not reveal any clear evidence of differential adverse event incidence on the basis of age, gender, or race.

Dose-Related Adverse Events

Schizophrenia

Dose response relationships for the incidence of treatment-emergent adverse events were evaluated from four trials in patients with schizophrenia comparing various fixed doses (2, 10, 15, 20, and 30 mg/day) of aripiprazole to placebo. This analysis, stratified by study, indicated that the only adverse event to have a possible dose response relationship, and then most prominent only with 30 mg, was somnolence (placebo, 7.7%; 15 mg, 8.7%; 20 mg, 7.5%; 30 mg, 15.3%).

Extrapyramidal Symptoms

In the short-term, placebo-controlled trials of schizophrenia, the incidence of reported EPS for aripiprazole-treated patients was 6% vs. 6% for placebo. In the short-term, placebo-controlled trials in bipolar mania, the incidence of reported EPS-related events excluding events related to akathisia for aripiprazole-treated patients was 17% vs. 12% for placebo. In the short-term, placebo-controlled trials in bipolar mania, the incidence of akathisia-related events for aripiprazole-treated patients was 15% vs. 4% for placebo. Objectively collected data from those trials was collected on the Simpson Angus Rating Scale (for EPS), the Barnes Akathisia Scale (for akathisia) and the Assessments of Involuntary Movement Scales (for dyskinesias). In the schizophrenia trials, the objectively collected data did not show a difference between aripiprazole and placebo, with the exception of the Barnes Akathisia Scale (aripiprazole, 0.08; placebo, -0.05). In the bipolar mania trials, the Simpson Angus Rating Scale and the Barnes Akathisia Scale showed a significant difference between aripiprazole and placebo (aripiprazole, 0.61; placebo, 0.03 and aripiprazole, 0.25; placebo, -0.06). Changes in the Assessments of Involuntary Movement Scales were similar for the aripiprazole and placebo groups.

Similarly, in a long-term (26-week), placebo-controlled trial of schizophrenia, objectively collected data on the Simpson Angus Rating Scale (for EPS), the Barnes Akathisia Scale (for akathisia), and the Assessments of Involuntary Movement Scales (for dyskinesias) did not show a difference between aripiprazole and placebo.

Laboratory Test Abnormalities

A between group comparison for 3- to 6-week, placebo-controlled trials revealed no medically important differences between the aripiprazole and placebo groups in the proportions of patients experiencing potentially clinically significant changes in routine serum chemistry, hematology, or urinalysis parameters. Similarly, there were no aripiprazole/placebo differences in the incidence of discontinuations for changes in serum chemistry, hematology, or urinalysis.

In a long-term (26-week), placebo-controlled trial there were no medically important differences between the aripiprazole and placebo patients in the mean change from baseline in prolactin, fasting glucose, triglyceride, HDL, LDL, and total cholesterol measurements.

Weight Gain

In 4- to 6- week trials in schizophrenia, there was a slight difference in mean weight gain between aripiprazole and placebo patients (+0.7 kg vs. -0.05 kg, respectively), and also a difference in the proportion of patients meeting a weight gain criterion of ≥7% of body weight [aripiprazole (8%) compared to placebo (3%)]. In 3-week trials in mania, the mean weight gain for aripiprazole and placebo patients was 0.0 kg vs. -0.2 kg, respectively. The proportion of patients meeting a weight gain criterion of ≥7% of body weight was aripiprazole (3%) compared to placebo (2%).

Table 3 provides the weight change results from a long-term (26-week), placebo-controlled study of aripiprazole, both mean change from baseline and proportions of patients meeting a weight gain criterion of ≥7% of body weight relative to baseline, categorized by BMI at baseline:

Table 3: Weight Change Results Categorized by BMI at Baseline: Placebo-Controlled Study in Schizophrenia, Safety Sample

	BMI <23		BMI 23-27		BMI >27	
	Placebo	Aripiprazole	Placebo	Aripiprazole	Placebo	Aripiprazole
Mean change from baseline (kg)	-0.5	-0.5	-0.6	-1.3	-1.5	-2.1
% with ≥7% increase BW	3.7%	6.8%	4.2%	5.1%	4.1%	5.7%

Table 4 provides the weight change results from a long-term (52-week) study of aripiprazole, both mean change from baseline and proportions of patients meeting a weight gain criterion of ≥7% of body weight relative to baseline, categorized by BMI at baseline:

Table 4: Weight Change Results Categorized by BMI at Baseline: Active-Controlled Study in Schizophrenia, Safety Sample

	BMI <23	BMI 23-27	BMI >27
Mean change from baseline (kg)	2.6	1.4	-1.2
% with ≥7% increase BW	30%	19%	8%

ECG Changes

Between group comparisons for a pooled analysis of placebo-controlled trials in patients with schizophrenia or bipolar mania, revealed no significant differences between aripiprazole and placebo in the proportion of patients experiencing potentially important changes in ECG parameters. Aripiprazole was associated with a median increase in heart rate of 5 beats per minute compared to a 1 beat per minute increase among placebo patients.

Additional Findings Observed in Clinical Trials

Adverse Events in Long-Term, Double-Blind, Placebo-Controlled Trials

The adverse events reported in a 26-week, double-blind trial comparing ABILIFY and placebo in patients with schizophrenia were generally consistent with those reported in the short-term, placebo-controlled trials, except for a higher incidence of tremor [9% (13/153) for ABILIFY vs. 1% (2/153) for placebo]. In this study, the majority of the cases of tremor were of mild intensity (9/13 mild and 4/13 moderate), occurred early in therapy (9/13 ≤49 days), and were of limited duration (9/13 ≤10 days). Tremor infrequently led to discontinuation (<1%) of ABILIFY. In addition, in a long-term (52-week), active-controlled study, the incidence of tremor for ABILIFY was 4% (34/859). A similar adverse event profile was observed in a long-term study in bipolar disorder.

Other Adverse Events Observed During the Premarketing Evaluation of Aripiprazole

Following is a list of modified COSTART terms that reflect treatment-emergent adverse events as defined in the introduction to the **ADVERSE REACTIONS** section reported by patients treated with aripiprazole at multiple doses ≥2 mg/day during any phase of a trial within the database of 7951 patients. All reported events are included except those already listed in Table 2, or other parts of the **ADVERSE REACTIONS** section, those considered in the **WARNINGS** or **PRECAUTIONS**, those event terms which were so general as to be uninformative, events reported with an incidence of ≤0.05% and which did not have a substantial probability of being acutely life-threatening, events that are otherwise common as background events, and events considered unlikely to be drug related. It is important to emphasize that, although the events reported occurred during treatment with aripiprazole, they were not necessarily caused by it.

Events are further categorized by body system and listed in order of decreasing frequency according to the following definitions: frequent adverse events are those occurring in at least 1/100 patients (only those not already listed in the tabulated results from placebo-controlled trials appear in this listing); infrequent adverse events are those occurring in 1/100 to 1/1000 patients; rare events are those occurring in fewer than 1/1000 patients.

Body as a Whole: Frequent - flu syndrome, fever, chest pain, rigidity (including neck and extremity), neck pain, pelvic pain; *Infrequent* - face edema, suicide attempt, malaise, migraine, chills, photosensitivity, tightness (including abdomen, back, extremity, head, jaw, neck, and tongue), jaw pain, bloating, enlarged abdomen, chest tightness, throat pain; *Rare* - moniliasis, head heaviness, throat tightness, Mendelson's syndrome, heat stroke.

Cardiovascular System: Frequent - tachycardia (including ventricular and supraventricular), hypotension, bradycardia; *Infrequent* - palpitation, hemorrhage, heart failure, myocardial infarction, cardiac arrest, atrial fibrillation, AV block, prolonged QT interval, extrasystoles, myocardial ischemia, deep vein thrombosis, angina pectoris, pallor, cardiopulmonary arrest, phlebitis; *Rare* - bundle branch block, atrial flutter, vasovagal reaction, cardiomegaly, thrombophlebitis, cardiopulmonary failure.

Digestive System: Frequent - nausea and vomiting; *Infrequent* - increased appetite, dysphagia, gastroenteritis, flatulence, tooth caries, gastritis, gingivitis,

gastrointestinal hemorrhage, hemorrhoids, gastroesophageal reflux, periodontal abscess, fecal incontinence, rectal hemorrhage, stomatitis, colitis, tongue edema, cholecystitis, mouth ulcer, oral moniliasis, eructation, fecal impaction, cholelithiasis; *Rare* - esophagitis, hematemesis, intestinal obstruction, gum hemorrhage, hepatitis, peptic ulcer, glossitis, melena, duodenal ulcer, cheilitis, hepatomegaly, pancreatitis.

Endocrine System: Infrequent - hypothyroidism; *Rare* - goiter, hyperthyroidism.

Hemic/Lymphatic System: Frequent - ecchymosis, anemia; *Infrequent* - hypochromic anemia, leukocytosis, leukopenia (including neutropenia), lymphadenopathy, eosinophilia, macrocytic anemia; *Rare* - thrombocythemia, thrombocytopenia, petechiae.

Metabolic and Nutritional Disorders: Frequent - weight loss, creatine phosphokinase increased, dehydration; *Infrequent* - edema, hyperglycemia, hypercholesteremia, hypokalemia, diabetes mellitus, hypoglycemia, hyperlipemia, SGPT increased, thirst, BUN increased, hyponatremia, SGOT increased, creatinine increased, cyanosis, alkaline phosphatase increased, bilirubinemia, iron deficiency anemia, hyperkalemia, hyperuricemia, obesity; *Rare* - lactic dehydrogenase increased, hypernatremia, gout, hypoglycemic reaction.

Musculoskeletal System: Frequent - muscle cramp; *Infrequent* - arthralgia, myasthenia, arthrosis, bone pain, arthritis, muscle weakness, spasm, bursitis, myopathy; *Rare* - rheumatoid arthritis, rhabdomyolysis, tendonitis, tenosynovitis.

Nervous System: Frequent - depression, nervousness, schizophrenic reaction, hallucination, hostility, confusion, paranoid reaction, suicidal thought, abnormal gait, manic reaction, delusions, abnormal dream; *Infrequent* - emotional lability, twitch, cogwheel rigidity, impaired concentration, dystonia, vasodilation, paresthesia, impotence, extremity tremor, hypesthesia, vertigo, stupor, bradykinesia, apathy, panic attack, decreased libido, hypersomnia, dyskinesia, manic depressive reaction, ataxia, visual hallucination, cerebrovascular accident, hypokinesia, depersonalization, impaired memory, delirium, dysarthria, tardive dyskinesia, amnesia, hyperactivity, increased libido, myoclonus, restless leg, neuropathy, dysphoria, hyperkinesia, cerebral ischemia, increased reflexes, akinesia, decreased consciousness, hyperesthesia, slowed thinking; *Rare* - blunted affect, euphoria, incoordination, oculogyric crisis, obsessive thought, hypotonia, buccoglossal syndrome, decreased reflexes, derealization, intracranial hemorrhage.

Respiratory System: Frequent - sinusitis, dyspnea, pneumonia, asthma; *Infrequent* - epistaxis, hiccup, laryngitis, aspiration pneumonia; *Rare* - pulmonary edema, increased sputum, pulmonary embolism, hypoxia, respiratory failure, apnea, dry nasal passages, hemoptysis.

Skin and Appendages: Frequent - skin ulcer, sweating, dry skin; *Infrequent* - pruritus, vesiculobullous rash, acne, eczema, skin discoloration, alopecia, seborrhea, psoriasis; *Rare* - maculopapular rash, exfoliative dermatitis, urticaria.

Special Senses: Frequent - conjunctivitis; *Infrequent* - ear pain, dry eye, eye pain, tinnitus, cataract, otitis media, altered taste, blepharitis, eye hemorrhage, deafness; *Rare* - diplopia, frequent blinking, ptosis, otitis externa, amblyopia, photophobia.

Urogenital System: Frequent - urinary incontinence; *Infrequent* - urinary frequency, leukorrhea, urinary retention, cystitis, hematuria, dysuria, amenorrhea, vaginal hemorrhage, abnormal ejaculation, kidney failure, vaginal moniliasis, urinary urgency, gynecomastia, kidney calculus, albuminuria, breast pain, urinary burning; *Rare* - nocturia, polyuria, menorrhagia, anorgasmy, glycosuria, cervicitis, uterus hemorrhage, female lactation, urolithiasis, priapism.

Other Events Observed During the Postmarketing Evaluation of Aripiprazole

Voluntary reports of adverse events in patients taking aripiprazole that have been received since market introduction and not listed above that may have no causal relationship with the drug include rare occurrences of allergic reaction (eg, anaphylactic reaction, angioedema, laryngospasm, pruritis, or urticaria).

DRUG ABUSE AND DEPENDENCE

Controlled Substance

ABILIFY (aripiprazole) is not a controlled substance.

Abuse and Dependence

Aripiprazole has not been systematically studied in humans for its potential for abuse, tolerance, or physical dependence. In physical dependence studies in monkeys, withdrawal symptoms were observed upon abrupt cessation of dosing. While the clinical

trials did not reveal any tendency for any drug-seeking behavior, these observations were not systematic and it is not possible to predict on the basis of this limited experience the extent to which a CNS-active drug will be misused, diverted, and/or abused once marketed. Consequently, patients should be evaluated carefully for a history of drug abuse, and such patients should be observed closely for signs of ABILIFY misuse or abuse (eg, development of tolerance, increases in dose, drug-seeking behavior).

OVERDOSAGE

Human Experience

In clinical studies, accidental or intentional acute overdosage of aripiprazole was identified in patients with estimated doses up to 1080 mg with no fatalities. The reported signs and symptoms observed with aripiprazole overdose included nausea, vomiting, asthenia, diarrhea, and somnolence. In the patients who were evaluated in hospital settings, there were no reported observations indicating clinically significant adverse change in vital signs, laboratory assessments, or ECG.

During postmarketing experience, the reported signs and symptoms observed in adult patients who overdosed with aripiprazole alone at doses up to 450 mg included tachycardia. In addition, reports of accidental overdose with aripiprazole (up to 195 mg) in children have been received. The potentially medically serious signs and symptoms reported include extrapyramidal symptoms and transient loss of consciousness with recovery.

Management of Overdosage

No specific information is available on the treatment of overdose with aripiprazole. An electrocardiogram should be obtained in case of overdosage and, if QTc interval prolongation is present, cardiac monitoring should be instituted. Otherwise, management of overdose should concentrate on supportive therapy, maintaining an adequate airway, oxygenation and ventilation, and management of symptoms. Close medical supervision and monitoring should continue until the patient recovers.

Charcoal: In the event of an overdose of ABILIFY, an early charcoal administration may be useful in partially preventing the absorption of aripiprazole. Administration of 50 g of activated charcoal, one hour after a single 15-mg oral dose of aripiprazole, decreased the mean AUC and Cmax of aripiprazole by 50%.

Hemodialysis: Although there is no information on the effect of hemodialysis in treating an overdose with aripiprazole, hemodialysis is unlikely to be useful in overdose management since aripiprazole is highly bound to plasma proteins.

DOSAGE AND ADMINISTRATION

Schizophrenia

Usual Dose

The recommended starting and target dose for ABILIFY is 10 or 15 mg/day administered on a once-a-day schedule without regard to meals. ABILIFY has been systematically evaluated and shown to be effective in a dose range of 10 to 30 mg/day, when administered as the tablet formulation, however, doses higher than 10 or 15 mg/day, the lowest doses in these trials, were not more effective than 10 or 15 mg/day. Dosage increases should not be made before 2 weeks, the time needed to achieve steady state.

Dosage in Special Populations

Dosage adjustments are not routinely indicated on the basis of age, gender, race, or renal or hepatic impairment status (see **CLINICAL PHARMACOLOGY: Special Populations**).

Dosage adjustment for patients taking aripiprazole concomitantly with potential CYP3A4 inhibitors: When concomitant administration of ketoconazole with aripiprazole occurs, aripiprazole dose should be reduced to one-half of the usual dose. When the CYP3A4 inhibitor is withdrawn from the combination therapy, aripiprazole dose should then be increased.

Dosage adjustment for patients taking aripiprazole concomitantly with potential CYP2D6 inhibitors: When concomitant administration of potential CYP2D6 inhibitors such as quinidine, fluoxetine, or paroxetine with aripiprazole occurs, aripiprazole dose should be reduced at least to one-half of its normal dose. When the CYP2D6 inhibitor is withdrawn from the combination therapy, aripiprazole dose should then be increased.

Dosage adjustment for patients taking potential CYP3A4 inducers: When a potential CYP3A4 inducer such as carbamazepine is added to aripiprazole therapy, the aripiprazole dose should be doubled (to 20 or 30 mg). Additional dose increases should

be based on clinical evaluation. When carbamazepine is withdrawn from the combination therapy, the aripiprazole dose should be reduced to 10 to 15 mg.

Maintenance Therapy

While there is no body of evidence available to answer the question of how long a patient treated with aripiprazole should remain on it, systematic evaluation of patients with schizophrenia who had been symptomatically stable on other antipsychotic medications for periods of 3 months or longer, were discontinued from those medications, and were then administered ABILIFY 15 mg/day and observed for relapse during a period of up to 26 weeks, demonstrated a benefit of such maintenance treatment (see **CLINICAL PHARMACOLOGY: Clinical Studies**). Patients should be periodically reassessed to determine the need for maintenance treatment.

Switching from Other Antipsychotics

There are no systematically collected data to specifically address switching patients with schizophrenia from other antipsychotics to ABILIFY or concerning concomitant administration with other antipsychotics. While immediate discontinuation of the previous antipsychotic treatment may be acceptable for some patients with schizophrenia, more gradual discontinuation may be most appropriate for others. In all cases, the period of overlapping antipsychotic administration should be minimized.

Bipolar Disorder

Usual Dose

In clinical trials, the starting dose was 30 mg given once a day. A dose of 30 mg/day was found to be effective when administered as the tablet formulation. Approximately 15% of patients had their dose decreased to 15 mg based on assessment of tolerability. The safety of doses above 30 mg/day has not been evaluated in clinical trials.

Dosage in Special Populations

See *Dosage in Special Populations* under **DOSAGE AND ADMINISTRATION: Schizophrenia**.

Maintenance Therapy

While there is no body of evidence available to answer the question of how long a patient treated with aripiprazole should remain on it, patients with Bipolar I Disorder who had been symptomatically stable on ABILIFY Tablets (15 mg/day or 30 mg/day with a starting dose of 30 mg/day) for at least 6 consecutive weeks and then randomized to ABILIFY Tablets (15 mg/day or 30 mg/day) or placebo and monitored for relapse, demonstrated a benefit of such maintenance treatment (see **CLINICAL PHARMACOLOGY: Clinical Studies**). While it is generally agreed that pharmacological treatment beyond an acute response in mania is desirable, both for maintenance of the initial response and for prevention of new manic episodes, there are no systematically obtained data to support the use of aripiprazole in such longer-term treatment (ie, beyond 6 weeks).

Oral Solution

The oral solution can be given on a mg-per-mg basis in place of the 5-, 10-, 15-, or 20-mg tablet strengths. Solution doses can be substituted for the tablet doses on a mg-per-mg basis up to 25 mg of the tablet. Patients receiving 30-mg tablets should receive 25 mg of the solution (see **CLINICAL PHARMACOLOGY: Pharmacokinetics**).

ANIMAL TOXICOLOGY

Aripiprazole produced retinal degeneration in albino rats in a 26-week chronic toxicity study at a dose of 60 mg/kg and in a 2-year carcinogenicity study at doses of 40 and 60 mg/kg. The 40- and 60-mg/kg doses are 13 and 19 times the maximum recommended human dose (MRHD) based on mg/m^2 and 7 to 14 times human exposure at MRHD based on AUC. Evaluation of the retinas of albino mice and of monkeys did not reveal evidence of retinal degeneration. Additional studies to further evaluate the mechanism have not been performed. The relevance of this finding to human risk is unknown.

HOW SUPPLIED

ABILIFY® (aripiprazole) Tablets are available in the following strengths and packages.

The 5-mg ABILIFY tablets are blue, modified rectangular tablets, debossed on one side with "A-007" and "5".

Bottles of 30	NDC 59148-007-13
Blister of 100	NDC 59148-007-35

The 10-mg ABILIFY tablets are pink, modified rectangular tablets, debossed on one side with "A-008" and "10".

Bottles of 30	NDC 59148-008-13
Blister of 100	NDC 59148-008-35

The 15-mg ABILIFY tablets are yellow, round tablets, debossed on one side with "A-009" and "15".

Bottles of 30	NDC 59148-009-13
Blister of 100	NDC 59148-009-35

The 20-mg ABILIFY tablets are white, round tablets, debossed on one side with "A-010" and "20".

Bottles of 30	NDC 59148-010-13
Blister of 100	NDC 59148-010-35

The 30-mg ABILIFY tablets are pink, round tablets, debossed on one side with "A-011" and "30".

Bottles of 30	NDC 59148-011-13
Blister of 100	NDC 59148-011-35

ABILIFY® (aripiprazole) Oral Solution (1 mg/mL) is supplied in child-resistant bottles along with a calibrated oral dosing cup. ABILIFY oral solution is available as follows:

150-mL bottle	NDC 59148-012-15

Storage

Tablets

Store at 25° C (77° F); excursions permitted to 15° C to 30° C (59° F to 86° F) [see USP Controlled Room Temperature].

Oral Solution

Store in a refrigerator at 2° C to 8° C (36° F to 46° F). Open bottles of ABILIFY oral solution should be stored in a refrigerator and can be used for up to 6 months after opening.

Tablets manufactured by Otsuka Pharmaceutical Co, Ltd, Tokyo, 101-8535 Japan or Bristol-Myers Squibb Company, Princeton, NJ 08543 USA

Oral solution manufactured by Bristol-Myers Squibb Company, Princeton, NJ 08543 USA

Distributed and marketed by Otsuka America Pharmaceutical, Inc, Rockville, MD 20850 USA

Marketed by Bristol-Myers Squibb Company, Princeton, NJ 08543 USA

US Patent Nos 4,734,416 and 5,006,528

Bristol-Myers Squibb Company   Otsuka America Pharmaceutical, Inc.

[coding and version control information appear here]   Revised March, 2005

©2005, Otsuka Pharmaceutical Co, Ltd, Tokyo, 101-8535 Japan

CENTER FOR DRUG EVALUATION AND RESEARCH

APPLICATION NUMBER:

21-436/S-005 & S-008 & 21-713/S-003

SUMMARY REVIEW

MEMORANDUM

Date: November 30, 2004

FROM: Director
Division of Neuropharmacological Drug Products/HFD-120

TO: File, NDA 21-436/S-005

SUBJECT: Action Memo for NDA 21-436/S-005, for the use of Abilify (aripiprazole) as maintenance treatment in patients with Bipolar I Disorder

NDA 21-436/S-005, for the use of Abilify (aripiprazole) as maintenance treatment in patients with Bipolar I Disorder, was submitted by Otsuka Maryland Research Institute on 1//28/04. Abilify is currently approved for the treatment of schizophrenia, and the acute treatment of manic and mixed episodes associated with Bipolar I Disorder. The current application contains the result of a single randomized withdrawal study in patients with Bipolar I Disorder who had been stable on aripiprazole treatment for at least 6 consecutive weeks.

This application has been reviewed by Dr. Teresa Podruchny, medical officer, Dr. Kun He, statistician, Dr. Sonia Tabacova, pharmacologist, Dr. Ni Khin, Division of Scientific Investigations, Dr. Sherita McLamore, chemist, and Dr. Paul Andreason, psychiatric drugs team leader. Relevant data pertaining to the safety of this drug, not submitted in this application, has also been reviewed by Dr. Marc Stone, safety reviewer.

As the clinical team has described in detail, the single study submitted by the sponsor in support of the effectiveness of aripiprazole as maintenance treatment in patients with Bipolar I Disorder required patients to have been considered stable on aripiprazole treatment at 30 mg/day (or less if not tolerated) for at least 4 consecutive visits separated by 2 weeks (a minimum of 6 weeks of stability). At that point, patients were randomized to continued treatment on aripiprazole or placebo, for up to 26 weeks. The primary outcome was the time to relapse, appropriately defined.

The study was performed in the US (N=123), Argentina (N=7), and Mexico (2 centers, N=30).

The following chart displays patient flow in the randomized phase:

	Arip (%)	Pbo (%)
Randomized	78	83
Completed	39 (50%)	28 (34%)
Relapsed	19 (24%)	36 (43%)
Withdrew Consent	6 (8%)	6 (7%)
Subject Unreliable	3 (4%)	5 (6%)
Adverse Event	5 (6%)	1 (1%)
Lost to F/U	1 (1%)	1 (1%)
Missing	0	1 (1%)
Other known causes	4 (5%)	6 (7%)

The p-value for the drug-placebo difference (log-rank test) was 0.02.

Dr. He performed an analysis in which he considered all patients who discontinued the trial early (other than those classified as having relapsed) as having relapsed: the p-value for the drug-placebo contrast was 0.06. At my request, he also performed an analysis in which patients who discontinued due to an adverse event were considered to have met relapse criteria; the p-value was 0.09.

He also performed analyses of time to manic relapse (p=0.008) and time to depressive relapse (p=0.68).

The primary finding of interest, to which all clinical reviewers allude, is represented in the following charts:

Country	Arip		Placebo	
	N	Relapsed	N	Relapsed
Argentina	4	1 (25%)	3	3 (100%)
Mexico	14	1 (7%)	16	7 (44%)
US	59	17 (29%)	64	26 (41%)

Mexican Results by Center

Center	Arip		Placebo	
	N	Relapsed	N	Relapsed
093	6	0 (0%)	7	5 (71%)
118	8	1 (13%)	9	2 (22%)

As can be seen in the above tables, the relapse rate in aripiprazole-treated patients in Mexico is considerably lower than that seen elsewhere, especially in the US, where there are considerably more patients. Indeed, Dr. He has performed the following analyses that exclude various sub-sets of the data:

P-value without Mexican data	P=0.113
P-value without Center 093	P=0.1
P-value without Center 118	P=0.02
P-value with only US data	P=0.195

Clearly, based on these retrospective analyses, the study loses significance when the data from the very small Mexican center 093 is excluded.

As noted earlier, Dr. Marc Stone has reviewed additional safety data for aripiprazole.

Specifically, based on findings of increased risk of cerebrovascular adverse events with risperidone and olanzapine in patients with dementia, the division had asked sponsors of atypical antipsychotic drug products to examine their own databases for any similar potential risk. Otsuka has responded to this request with data from three controlled trials in patients with psychosis associated with Alzheimer's Disease.

Dr. Stone has reviewed these data in detail. Two of the studies (Studies 005 and 006) examined flexible doses of aripiprazole (from 2-15 mg/day; I do not know what the distribution of actual doses was in these studies), and in one (Study 004), patients were randomized to placebo or aripiprazole 2, 5, or 10 mg/day.

In these three controlled trials, a total of 343 patients were randomized to placebo, and 595 were randomized to aripiprazole (Study 004, N=480; Study 005, N=251; Study 006, N=207). The following chart displays the comparisons between the risk of CVAEs with aripiprazole and placebo:

Risk across all three randomized trials

	Any CVAE	Risk (Per 100 Pt-Yrs)
Placebo (N=343)	2	3.7
Aripiprazole (N=595)	8	8.4

P-value (log-rank) 0.29 Risk Ratio 2.27

Risk in Study 004 (Per 100 pt-yrs) by dose

	Pbo (N=120)	Ari 2 mg (N=116)	Ari 5 mg (N=121)	Ari 10 mg (N=123)
# of Events	0	1	2	4
Rate (Per 100 pt-yrs)	0	5.3	10.7	21.7

P-value (CMH)=0.03

In the 2 mg group, the event was reported as a cerebrovascular accident (CVA). In the 5 mg group, one event was listed as a CVA, the other as facial paralysis. In the 10 mg group, 2 events were listed as cerebral ischemia, one was listed as a CVA, and one was listed as an intracerebral hemorrhage.

As can be inferred from the above two displays, there was no signal for CVAE risk in the flexible dose studies (in both studies together, there was one event in the aripiprazole groups and two events in the placebo-treated patients).

As Dr. Stone notes (see his Table 8, page 11), the rate ratio for CVAEs for aripiprazole (all controlled trials) is remarkably similar to that for olanzapine and risperidone (2.43 for aripiprazole and olanzapine; 2.60 for risperidone).

COMMENTS

The sponsor has submitted a single controlled trial that, on face, provides evidence that aripiprazole continues to be effective in patients with Bipolar I Disorder who have been stabilized for at least 6 weeks. Two issues, however, raise concerns about how reliable these results are.

First, as noted above, the statistical significance of the study is driven by the results at a single, small center in Mexico, in which the treatment effect is considerably greater than in the combined US centers, which enrolled many more patients. Removing the data from this single center causes the study to lose significance. Although, strictly speaking, there is no formal statistical justification for removing this center's data from the analysis, the clear dependence of the study's overall statistical significance on the results of this small center raises questions about how robust and reliable these results really are. We have discussed the data from this center with Dr. Khin of the Division of Scientific Investigations (DSI), both because of the results themselves, but also because we have little experience with Mexican clinical centers. As Dr.

Podruchny notes, according to Dr. Khin, there is no obvious reason to believe that the conduct of the study at this center differed materially from that at other centers, or that this conduct deviated in important ways from that at centers in areas with which we have more experience. Nonetheless, we will ask the sponsor to address the questions raised by the extreme results at this site.

Second, as Dr. Podruchny notes, there are questions about the reasons why some patients who were not classified as having met relapse criteria discontinued treatment early. In these studies, it is critical to be able to account for the reason each patient left the trial early. Inadequate descriptions of these reasons (e.g., patient withdrew consent) raise the possibility that some of these patients may have left the study because of worsening of their clinical state, even if they did not meet formal relapse criteria (for example, the onset of insomnia, given as a reason for early discontinuation of one patient, might be the beginning of the return of manic or depressive symptoms). For these reasons, then, we will ask the sponsor to provide a more detailed account of the reasons why each patient (not just those in Center 093) who left the study early did so.

Regarding the data on cerebrovascular adverse events reviewed by Dr. Stone, there are reasons to question the propriety of combining data from all three aripiprazole controlled trials. As Dr. Stone notes, not only were the patients in Study 006 potentially different from those in the other two studies (for example, the patients in Study 006 had [some] fewer risk factors for stroke), but the study designs were importantly different (Study 004 was the only fixed dose study). Dr. Stone notes that the sponsor concludes that the absence of any signal in the two flexible dose studies "sheds doubt" on the (weak) signal seen in Study 004. Dr. Stone, on the other hand, does not agree, and suggests that "The paucity of CAEs in both the drug and placebo groups in the [005 and 006] studies..." provides little useful data (one way or the other) on the question. He concludes, for example, that the lower risk for CVAEs in patients in Study 006 makes it "unlikely" that any meaningful differences in CVAE risk could be seen in a study of that size (the total number of patients treated with aripiprazole in these two studies was 235). He does conclude, however, that "...almost all of the meaningful information comes from [Study 004].".

In this, I believe Dr. Stone, the sponsor, and I agree. I am not sure that the patients in Study 006 are, in fact, at so much less risk for stroke than patients in the other two studies (for example, although Dr. Stone believes that baseline differences in MMSE scores suggest that patients in Study 006 were not as impaired, I am not convinced that the small differences are meaningful in this regard, nor do I believe that the patients in Study 006 are materially younger than patients in the other two studies).

Whether we would have expected to see events in Studies 005 and 006 (assuming patients in 006 are reasonably similar), it bears pointing out that there were 235 patients in these studies combined who were treated with aripiprazole;

a total of 4 events were seen in the 123 patients treated with 10 mg in Study 004. Of course, as I noted above, I do not know the actual doses achieved in the flexible dose studies, and, clearly, dose could be a critical factor (the results of Study 004 clearly suggest that dose is a critical risk factor).

In any event, the dose response data from Study 004, although not confirmed in any other study, do suggest that aripiprazole may increase the risk of CVAEs in patients with Alzheimer's Disease (I do agree with Dr. Stone that the "negative" data from Studies 005 and 006 do not **necessarily** cast doubt on the findings in Study 004, mainly because the designs of these studies are significantly different from that of Study 004, and, for the reasons discussed by Dr. Stone, may not have been expected to yield similar results). Whether this is also true for elderly patients in general, we cannot know, given that no other elderly subjects have been so studied. Nonetheless, we will propose that a statement describing these results be placed in the Warning section of labeling, analogous to statements in the risperidone and olanzapine product labels.

For the reasons discussed above, then, I will issue the attached Approvable letter, with appended draft labeling.

Russell Katz, M.D.

This is a representation of an electronic record that was signed electronically and this page is the manifestation of the electronic signature.

/s/

Russell Katz
11/30/04 02:24:38 PM
MEDICAL OFFICER

MEMORANDUM

DATE: February 26, 2005

FROM: Director
Division of Neuropharmacological Drug Products/HFD-120

TO: File, NDA 21-436/S-005, S-008 & NDA 21-713/S-003

SUBJECT: Action Memo for NDA 21-436/S-005, S-008 Abilify (aripiprazole) Tablets & NDA 21-713/S-003 Abilify (aripiprazole) Oral Solution for the use as maintenance treatment in Bipolar Disorder

NDA 21-436/S-005, for the use of Abilify (aripiprazole) Tablets as maintenance treatment in Bipolar Disorder, was submitted on 1/28/04 by Otsuka Maryland Research Institute. The Division issued an Approvable letter on 11/30/04 asking the sponsor for additional information (see below). Supplement 008 to this application was submitted by Otsuka on 12/27/04, and proposed specific language to describe the occurrence of strokes in elderly patients with dementia. NDA 21-713/S-003 Abilify (aripiprazole) Oral Solution, for the granting of the maintenance claim for the oral solution, was submitted by Otsuka on 2/16/05.

As noted above, in our 11/30/04 Approvable letter to NDA 21-436/S-005, we asked the sponsor to address the following issues:

1) Although the single study submitted by the sponsor to support the maintenance claim reached statistical significance on its primary effectiveness measure, the trial lost significance when the data from a single Mexican center were removed (i.e., the treatment effect seen at that center was markedly greater than that seen in the combined US centers). Although there was no obvious reason to believe that the results at that center were compromised (a DSI inspection found no major flaws), we asked the sponsor to address this issue.
2) A number of patients had discontinued the study prior to having reached an endpoint or completing the entire duration of the study. For many of these patients, the reasons for these discontinuations were unclear, and the results of the trial could have been different had some of these patients left the trial early because of worsening of their condition. For this reason, we asked the sponsor to re-evaluate these discontinuations.
3) We asked the sponsor for written confirmation of numerous Phase 4 commitments.
4) We asked the sponsor to include language in product labeling related to cerebrovascular adverse events (CVAEs; this language was submitted as supplement 008 to the NDA; see above).

The sponsor responded to the Approvable letter on 1/3/05. The response has been reviewed by Dr. Greg Dubitsky, medical officer and Dr. Paul Andreason, Psychiatry Drugs Team Leader. The review team recommends that the application be approved once agreement has been reached with the sponsor on language for the label.

I agree that the application may be approved.

Specifically, with regard to our concern that the overall outcome was dependent upon the results at a single Mexican center, the sponsor has argued that there is no valid reason for excluding this center, and that the crude relapse rates (for all relapses, as well as for depressive and manic relapses), as well as the mean changes in the Y-MRS, although not the primary outcomes, show minimal changes when the Mexican site is excluded. Although we had similar doubts about the validity of excluding this site, we believed it was worth asking the sponsor to examine the question; I am now convinced that we should accept the results of the study when analyzed as planned (that is, with the inclusion of the site).

Regarding the potential re-classification of (some) patients as having met relapse criteria, the sponsor has re-evaluated all of these patients, and believes that, in 12 of these patients, a relapse could not have been "absolutely" ruled out. When these patients were included in a re-analysis as having met relapse criteria, the results still achieve statistical significance. Further, Dr. Dubitsky has reviewed descriptions of all of these cases. In his view, only 2 of these patients could reasonably have been considered to have met relapse criteria; a re-analysis including only these 2 additional patients also was significant. I agree that this answers our second question.

All other issues raised in the Approvable letter have been resolved satisfactorily. In particular, we have reached agreement with the sponsor on final labeling. For this reason, then, I will issue the attached Approval letter, with appended agreed-upon labeling.

Russell Katz, M.D.

This is a representation of an electronic record that was signed electronically and this page is the manifestation of the electronic signature.

/s/

Russell Katz
3/1/05 10:37:45 AM
MEDICAL OFFICER

CENTER FOR DRUG EVALUATION AND RESEARCH

APPLICATION NUMBER:

21-436/S-005 & S-008 & 21-713/S-003

CROSS DISCIPLINE TEAM LEADER REVIEW

MEMORANDUM **DEPARTMENT OF HEALTH AND HUMAN SERVICES**
PUBLIC HEALTH SERVICE
FOOD AND DRUG ADMINISTRATION
CENTER FOR DRUG EVALUATION AND RESEARCH

DATE: November 30, 2004

FROM: Paul J. Andreason, M.D.
Team Leader, Psychiatric Drug Products
Division of Neuropharmacological Drug Products
HFD-120

SUBJECT: Recommendation for Approvable Action for NDA 21-436 Supplement 005
Extended Efficacy of Aripiprazole in the Treatment of Bipolar Mania

TO: File, NDA 21-436
[Note: This memo should be filed with the January 28, 2004 original submission of this NDA.]

1.0 BACKGROUND

Aripiprazole is an atypical antipsychotic that is approved for the treatment of schizophrenia and the acute treatment of bipolar mania. It was approved in the U.S. for use in the treatment of schizophrenia on November 15, 2002 and for acute manic and mixed episodes associated with Bipolar Disorder on September 28, 2004.

The sponsor's efficacy claim is supported by one clinical study, CN 138010, a multi-center double-blind, randomized trial. Since the acute efficacy claim was recently reviewed and approved, it was decided by the Division that a single longer term study was sufficient for a longer term maintenance claim.

The primary clinical reviewer was Teresa Podruchny, MD and the primary statistical review was performed by Kun He, PhD of the Division of Biometrics (HFD-710).

2.0 CHEMISTRY

There were no chemistry issues on this submission as aripiprazole is already a marketed product.

3.0 PHARMACOLOGY

There were no animal pharmacology issues on this submission as aripiprazole is already a marketed product.

4.0 BIOPHARMACEUTICS

There were no biopharmaceutical issues on this submission given the recent review and approval of the acute treatment claim.

5.0 CLINICAL DATA

5.1 Efficacy Data

The sponsor's proposed efficacy claim was supported by the single study 131010 in this submission. Study 138010 was based on an open-label stabilization of a cohort of patients with DSM-IV Bipolar Disorder who presented for treatment with an acute Mixed or Manic episode. The sponsor describes study 138010 as a three phase study: 1) stabilization, 2) maintenance and 3) extension. The sponsor appears to imply that the duration of the claim of efficacy is 26-weeks; however, the sponsor was informed in pre-NDA discussions that the duration of the maintenance claim would reflect the duration of the open label period of stabilization and not the nominal duration of the double blind observation period.

The phases of the study progressed in the following manner. After open-label treatment with aripiprazole a patient met stabilization criteria when a Young-Mania Rating Scale (Y-MRS) Score of ≤ 10 and a Montgomery-Asberg Depression Rating Scale (MADRS) Score of ≤ 13 during 4 consecutive visits. The patient continued into a maintenance phase (double blind phase) after they remained stable for at least 6 weeks (4 consecutive visits separated by periods of 2-weeks). Patients at this point were randomized to either continue aripiprazole or placebo. The sponsor labels this the "maintenance" phase; however, this is a double blind treatment withdrawal period. The Division defines the maintenance phase of a relapse-prevention designed trial as the 6-week period of 4 consecutively stable rating scale scores. Time to relapse was then measured over a period of up to 26-weeks. If a patient remained stable for the entire 26-week period then they had the option of continuing on open-label aripiprazole (extension phase).

A total of 633 subjects enrolled in the study. Of these 633 there were 206 who met randomization criteria. 191 were randomized, but 35 patients across several sites received randomized yet unblinded study drug. These 35 patients were disqualified so that there were 161 patients who went on to be randomized to the double-blind phase. The ITT population included 83 subjects in placebo group and 77 subjects in aripiprazole group. The primary efficacy endpoint was the time from randomization to relapse during the maintenance phase. The primary analysis was a log-rank test of time to relapse. Relapse was defined clinically as taking place when a patient discontinued from the study due to lack of efficacy, if they were hospitalized or required an addition to or increase in their allowed psychotropic medications, other than the study medication, for manic or depressive symptoms.

The log-rank test produced a p-value of 0.02 where there were 36 out of 83 (43%) patients who relapsed in placebo, and 19 out of 77 (25%) patients who relapsed in aripiprazole groups, respectively. Dr. Kun He noted, "One issue is whether the study is robust because center 093 in Mexico, where there were 7 in placebo and 6 in aripiprazole groups, respectively, had 5 (71%) relapsed in placebo and 0 (0%) relapsed in aripiprazole groups, respectively. The primary analysis is not significant after removing center 093."

5.2 Safety

In a safety review that was not part of this submission, the Safety Team concluded that aripiprazole labeling required the addition of the description of cerebrovascular adverse events (CVAE) in the elderly to the WARNINGS section. Judith Racoosin, MD the Safety Team Leader, provided the following draft labeling:

Cerebrovascular Adverse Events, Including Stroke, in Elderly Patients with Dementia —
[ ] (e.g., stroke, transient ischemic attack), including fatalities,
[ ] patients [ ]. In [ ] b(4)
[ ] fixed dose [ ] there was a statistically significant dose response

relationship for cerebrovascular adverse events in patients treated with aripiprazole. Aripiprazole is not approved for the treatment of patients with dementia-related psychosis.

5.3 Clinical Sections of Labeling
Draft labeling is attached to this package.

6.0 WORLD LITERATURE
A world literature review was provided in the safety update for the response to the approvable action letter for supplement 002. That review of the literature is adequate and supercedes the one performed in this submission.

7.0 FOREIGN REGULATORY ACTIONS
I am not aware of any foreign regulatory actions regarding this claim in non-US labeling.

8.0 PSYCHOPHARMACOLOGICAL DRUGS ADVISORY COMMITTEE (PDAC) MEETING
We decided not to take this supplement to the PDAC.

9.0 DSI INSPECTIONS
The Division of Scientific Investigations (DSI) inspected three sites; one U.S. site (64) and two sites in Mexico (93 and 118). These sites were chosen due to either sample size or impact on study significance. The DSI inspection report was written by Dr. Ni Khin. Dr Khin felt that the sites generally followed good research practices and despite some deficiencies the data were acceptable.

10.0 APPROVABLE LETTER
An approvable letter and proposed draft labeling is attached to this review package.

11.0 CONCLUSIONS AND RECOMMENDATIONS
Study 138010 is positive and supports the claim of 6-weeks of extended efficacy in the maintenance treatment of Bipolar I Disorder. Prior to approval the sponsor should investigate the reasons behind the unusually high rate of placebo dropout and unusually high rate of aripiprazole retention at this site. If it can be determined that there was a bias in favor of keeping aripiprazole patients and discontinuing placebo patients in this study site, then I would consider 138010 a failed study and would not recommend approving supplement 005. In order to reach final approval, I believe that the sponsor needs to address the following issues:

1. Investigate and explain the possible causes for the disproportionately high patient dropout rate in the placebo group and disproportionately high retention rate in the aripiprazole group at site 93.
2. Reach agreement on final labeling language which shall include WARNING language on the risk of cerebrovascular adverse events (CVAE) in the elderly.

This is a representation of an electronic record that was signed electronically and this page is the manifestation of the electronic signature.

/s/

Paul Andreason
11/30/04 04:46:59 PM
MEDICAL OFFICER

CENTER FOR DRUG EVALUATION AND RESEARCH

APPLICATION NUMBER:

21-436/S-005 & S-008 & 21-713/S-003

MEDICAL REVIEW(S)

Review and Evaluation of Clinical Data

NDA: 21-436
Sponsor: Bristol-Myers Squibb / Otsuka
Drug: Aripiprazole (Abilify®)
Material Reviewed: Response to request for information
Subject: Association between aripiprazole and cerebrovascular adverse events
Reviewer: Marc Stone, M.D.
Submission Dates: 7/30/2003, 5/19/2004
Date Review Completed: November 9, 2004

Background

On January 30, 2003 the Agency requested from sponsors information on the incidence of Cerebrovascular Adverse Events (CAEs) in the population of elderly patients from the Psychosis associated with Alzheimer's Disease (PAD) clinical trial database and any post-marketing experience. Otsuka and Bristol-Myers Squibb originally responded on July 30, 2003 with data from studies CN138005 and CN138006, with a data cutoff of May 7, 2003. Since the original submission, a placebo-controlled trial in this patient population, CN138004, and open label extension phases for the -004 and -005 studies were completed and were analyzed with a data cutoff of January 8, 2004.

Methodology

Patient population
The sponsors report the results of clinical studies that enrolled a total of 968 subjects with a diagnosis of PAD. Thirty of these subjects were in a small open label study. There were three randomized placebo-controlled trials in which 595 patients were treated with aripiprazole in the initial study phase while 343 were treated with placebo. These trials all included subsequent open-label extensions in which 269 subjects originally in the placebo groups received aripiprazole, and 625 patients originally randomized to aripiprazole continued on that therapy. Table 1 gives brief descriptions of each of these studies. The inclusion criteria of the four studies allowed only patients with diagnosis of dementia of the Alzheimer's type. Patients with diagnosis of dementia of the vascular type or mixed type were excluded. Analyses were performed on both the "Placebo Controlled Data Set" (data from the double-blind placebo controlled phases of CN138004, CN138005, and CN138006) and the "All Aripiprazole Alzheimer's Dementia Data Set" (data from all patients exposed to aripiprazole in all four studies, including the open label phases). Except where noted, this review will focus on the results of the placebo-controlled phases of CN138004, CN138005, and CN138006.

Study	Status As of Jan 8, 2004	Study Description
**Table 1: Studies in Patients with Dementia in the Aripiprazole Clinical Program**		
31-98-203	Completed	21-day open label pilot study in patients with early dementia, N=30 patients, Aripiprazole 5-30 mg in 5 dosing groups.
CN138004	10-wk phase and open label extension completed	10-wk double-blind, 4-arm, fixed dose placebo-controlled inpatient study in patients with psychosis in Alzheimer's dementia, 3 fixed aripiprazole dose groups (2, 5, or 10 daily) vs. placebo (Randomized to placebo=121, aripiprazole 2mg=118, aripiprazole 5mg=122, aripiprazole 10mg=126). 130-wk open label aripiprazole (2-15 mg) extension phase for eligible 10-wk phase completers
CN138005	10-wk phase and open label extension completed	10-wk double-blind flexible dose, placebo-controlled inpatient study in patients with psychosis in Alzheimer's dementia, aripiprazole (2-15 mg) (N=131) vs. placebo (N=125). 130-wk open label aripiprazole (2-15 mg) extension phase for eligible 10-wk phase completers
CN138006	10-wk phase completed, open label extension ongoing	10-wk double-blind flexible dose, placebo-controlled outpatient study in patient with psychosis in Alzheimer's dementia, aripiprazole (2-15 mg) (N=106) vs. placebo (N=102). 130-wk open label aripiprazole (2-15 mg) extension phase for eligible 10-wk phase completers

Definition of terms

The sponsor searched and reviewed the adverse event data from all four studies for potential cerebrovascular adverse events (CAEs). The initial search included a search for specific text strings in either the investigator verbatim term reported on the adverse event case report form or the COSTART preferred terms that might indicate CAEs. The text strings searched included 'CERE', 'ISCHEMIA', 'STROKE', 'CVA', 'C.V.A.', 'TIA', and 'T.I.A.'. In addition, COSTART preferred terms reported for all four studies were reviewed for possible CAEs. All of the adverse event data for each patient reporting a possible CAE term were reviewed to determine if the event in question would be considered a potential CAE. The terms actually occurring in the reports that were considered to indicate potential CAEs were cerebrovascular accident, cerebral ischemia, intracerebral hemorrhage, intracranial hemorrhage, subarachnoid hemorrhage, mydriasis (verbatim term mentioned 'possible TIA'), facial paralysis, subdural hematoma, cerebral edema, and consciousness decreased (the verbatim term includes transient ischemic attack).

Analytic methods

For all CAE incidence summaries (except time of first onset), the incidence rate of a potential CAE was calculated two ways; 1) using a patient based denominator - by

dividing the number of patients with at least one report of a CAE during the study or phase by the number of patients exposed to drug during the study or phase in the particular treatment arm, and 2) using a patient year denominator - by dividing the number of patients with at least one report of a CAE during the study or phase by the total number of patient years exposure in the particular treatment arm. All CAEs were considered as treatment-emergent unless the onset date proved otherwise (i.e., CAEs with an onset prior to the first day of study medication). For completed studies, CAEs with an onset more than 30 days after the last day of study medication were excluded. For ongoing studies, complete dosing information may not have been available at the time of the database cut-off. All events entered into the database as of the database cut-off, regardless of the time of onset relative to the last day of dosing, were reported for the ongoing studies.

Time to first onset of a potential CAE was analyzed for the placebo-controlled studies. A log-rank test was used to compare the time to first onset of a potential CAE between aripiprazole and placebo. Patients who did not have a potential CAE were censored on the day of their last dose of double-blind medication. The paucity of potential CAEs in these studies precluded additional statistical testing adjusting for other factors; these factors were examined using tabular comparisons. The Cochran-Mantel-Haenszel correlation test was performed to identify any positive dose response for CAEs.

Findings

Baseline Patient Characteristics in the Pool of PAD studies

Despite random allocation, there were some small differences between the populations of subjects who were assigned to placebo and those assigned to aripiprazole. Subjects assigned to aripiprazole were slightly more likely to be male, non-white, and have a history of stroke. They were also more likely to have two or more risk factors for stroke, including a history of atrial fibrillation, hypertension and diabetes but were less likely to have a history of heart attack (Table 2).

Table 2: Selected Baseline Characteristics by Treatment Assignment

Variable	N (%)	Placebo N = 343	Arip N = 595	Total N = 938
Sex	Men	77 (22)	146 (25)	223 (24)
	Women	266 (78)	449 (75)	715 (76)
Race	White	313 (91)	530 (89)	843 (90)
	Black	16 (5)	35 (6)	51 (5)
	Hispanic	8 (2)	17 (3)	25 (3)
	Asian	6 (2)	11 (2)	17 (2)
	Other	0	2 (<1)	2 (<1)

Any History of Stroke	Yes	23 (7)	65 (11)	88 (9)
	No	320 (93)	530 (89)	850 (91)
Number of Risks in Medical History (including History of Stroke)	None	101 (29)	159 (27)	260 (28)
	1	122 (36)	198 (33)	320 (34)
	2	74 (22)	143 (24)	217 (23)
	>= 3	46 (13)	95 (16)	141 (15)
Atrial Fibrillation History	Yes	24 (7)	59 (10)	83 (9)
	No	319 (93)	536 (90)	855 (91)
CHD/CHF History	Yes	66 (19)	126 (21)	192 (20)
	No	277 (81)	469 (79)	746 (80)
Hypertension History	Yes	178 (52)	323 (54)	501 (53)
	No	165 (48)	272 (46)	437 (47)
Heart Attack History	Yes	20 (6)	14 (2)	34 (4)
	No	323 (94)	581 (98)	904 (96)
Diabetes History	Yes	41 (12)	86 (14)	127 (14)
	No	302 (88)	509 (86)	811 (86)

Subjects for the placebo-controlled studies were not drawn from the same population and could have significant differences in demographic characteristics and stroke risk factors among them (Table 3). In general, the demographics and risk factors were balanced between the CN138004 and CN138005 studies. The population in CN138006 had a smaller percentage of stroke risk factors and was generally younger and less neurologically impaired (c.f. the MMSE).

Table 3: Selected Baseline Characteristics by Clinical Trial

Variable N (%)		CN138004<br>N = 480	CN138005<br>N = 251	CN138006<br>N = 207	005 vs. 006<br>p value*
Age (Yrs)	Mean	82.5	83.0	81.5	0.018
	18-64	15 (3)	6 (2)	4 (2)	0.188
	65-74	47 (10)	21 (8)	28 (14)	
	75-84	202 (42)	110 (44)	97 (47)	

	>= 85	216 (45)	114 (45)	78 (38)	
Sex	Men	103 (21)	61 (24)	59 (29)	0.337
	Women	377 (79)	190 (76)	148 (71)	
Race	White	417 (87)	224 (89)	202 (98)	0.001
	Black	28 (6)	20 (8)	3 (1)	
	Hispanic	19 (4)	6 (2)	0	
	Asian	14 (3)	1 (0)	2 (1)	
	Other	2 (<1)	0	0	
Baseline MMSE Total	Mean	12.4	12.8	13.6	0.062
Any History of Stroke	Yes	51 (11)	27 (11)	10 (5)	0.025
	No	429 (89)	224 (89)	197 (95)	
Any Risk History	Yes	357 (74)	191 (76)	115 (56)	<0.001
	No	123 (26)	60 (24)	92 (44)	
Number of Risks in Med. History (including History of Stroke)	Mean	1.4	1.6	0.8	<0.001
	None	116 (24)	54 (22)	90 (43)	<0.001
	1	172 (36)	73 (29)	75 (36)	
	2	115 (24)	72 (29)	30 (14)	
	>= 3	77 (16)	52 (21)	12 (6)	
Tobacco History	Yes	30 (6)	25 (10)	3 (1)	<0.001
	No	450 (94)	226 (90)	204 (99)	
Atrial Fibrillation History	Yes	52 (11)	23 (9)	8 (4)	0.011
	No	428 (89)	228 (91)	199 (96)	
CHD/CHF History	Yes	106 (22)	64 (25)	22 (11)	<0.001
	No	374 (78)	187 (75)	185 (89)	
Dyslipidemia History	Yes	66 (14)	44 (18)	17 (8)	0.004

	No	414 (86)	207 (82)	190 (92)	
Hypertension History	Yes	270 (56)	149 (59)	82 (40)	<0.001
	No	210 (44)	102 (41)	125 (60)	
Diabetes History	Yes	64 (13)	43 (17)	20 (10)	0.021
	No	416 (87)	208 (83)	187 (90)	

* Calculated by reviewer

CN138004 was the only trial that randomly assigned subjects to a fixed dosage. Potential confounders (demographic characteristics and stroke risk factors that correlate with dosage) are shown in Table 4.

Table 4: Selected Baseline Characteristics by Treatment in Study CN138004

Variable	N (%)	Placebo N = 120	Arip 2 mg N = 116	Arip 5 mg N = 121	Arip 10 mg N = 123
Sex	Men	22 (18)	23 (20)	28 (23)	30 (24)
	Women	98 (82)	93 (80)	93 (77)	93 (76)
Baseline MMSE Total	Mean	11.8	12.3	12.5	13.1
	Median	11.0	12.0	12.0	13.0
CHD/CHF History	Yes	24 (20)	22 (19)	29 (24)	31 (25)
	No	96 (80)	94 (81)	92 (76)	92 (75)

Incidence of Cerebrovascular Adverse Events

The incidence of treatment-emergent CAEs determined from the pooled sample of placebo-controlled studies (Table 5) was 1.3% (n=8) for aripiprazole and 0.6% (n=2) for placebo (log-rank, chi-sq= 1.14, p= 0.286). The incidence rate ratio was 2.25 with a 95% confidence interval of 0.45 to 21.78. Fisher's Exact test comparing the incidence rates (not adjusting for exposure time) was also consistent with chance (p= 0.341). One subject was taking warfarin and had a reported INR of 5.9. The incidence excluding this subject is aripiprazole 1.2% (7/595) and placebo 0.6% (2/343) (log rank chi-sq=0.76, p=0.383).

Table 5: Incidence of CAEs: Placebo-Controlled Studies in Alzheimer's Dementia						
	Placebo (N = 343) (53.8 pys)			Aripiprazole (N = 595) (95.5 pys)		
Primary Term	N	(% Pts)	(per 100py)	N	(% Pts)	(per 100 py)
Any CAE	2	(0.6)	(3.7)	8	(1.3)	(8.4)
Cerebrovascular Accident	1	(0.3)	(1.9)	3	(0.5)	(3.1)
Ischemia Cerebral	1	(0.3)	(1.9)	3	(0.5)	(3.1)
Hemorrhage Intracerebral	0			1	(0.2)	(1.0)
Paralysis Facial	0			1	(0.2)	(1.0)

py = patient exposure year

The incidence of treatment-emergent CAEs determined from the dose groups in the CN138004 study is seen in Table 6. The incidence of treatment emergent potential CAEs increased significantly with the dose of aripiprazole (p=0.030, CMH Row Means Score test). This dose-response effect was not seen in the flexible dosing studies, CN138005 and CN138006; the number of events was too small to establish any pattern.

Table 6: Incidence of CAEs By Dose Group: CN138004 Study												
	Placebo (N = 120) (17.8 py)			Arip 2mg (N = 116) (18.9 py)			Arip 5 mg (N =121) (18.7 py)			Arip 10 mg (N =123) (18.4 py)		
Primary Term	N	(%)	(per 100 py)	N	(%)	(per 100 py)	N	(%)	(per 100 py)	N	(%)	(per 100 py)
Any CAE	0			1	(0.9)	(5.3)	2	(1.7)	(10.7)	4	(3.3)	(21.7)
Ischemia Cerebral	0			0			0			2	(1.6)	(10.9)
Cerebrovascular Accident	0			1	(0.9)	(5.3)	1	(0.8)	(5.3)	1	(0.8)	(5.4)
Hemorrhage Intracerebral	0			0			0			1	(0.8)	(5.4)
Paralysis Facial	0			0			1	(0.8)	(5.3)	0		

py = patient exposure year

A number of factors showed a possible relationship with the incidence of CAEs. The incidence was greatest in the oldest patients. All of the CAEs occurred in females. This may reflect the greater proportion of females in the studies (76%); the difference had

borderline statistical significance. In the patients with a low (<14) MMSE, there is a higher percentage of subjects in the aripiprazole group with CAEs (1.2% vs 0 for placebo); again, statistical significance was borderline. All of the eight cases in the aripiprazole group had a prior medical history of CVA/stroke or stroke risk while there were no cases in the placebo group among those with a prior medical history of CVA/stroke or stroke risk.

Twenty-one (3.5%) aripiprazole treated patients died during or within 30 days of discontinuing the placebo-controlled phase. Six (1.7%) placebo treated patients died within the same period. The difference in mortality rates between aripiprazole and placebo during the placebo-controlled phase was not significant (log rank test, p=0.139).

Summary of Patient Narratives

Table 7: List of Aripiprazole Treated Patients Reporting a Treatment-Emergent Potential CAE in Placebo-Controlled Studies in Alzheimer's Dementia

Unique ID	Actual Treatment Group	Primary Term (COSTART)	Serious AE	Death	Study Day of Onset	Dose at Onset (mg)	Days on Dose
138004-110-405	Ari 2 mg	Cerebrovascular Accident	Yes	Yes	[ ]	2	26
138004-28-631	Ari 5 mg	Cerebrovascular Accident	No	No	30	5	30
138004-52-308	Ari 5 mg	Facial Paralysis	No	No	47	5	47
138004-15-9	Ari 10 mg	Cerebral Ischemia	Yes	No	44	10	37
138004-20-154	Ari 10 mg	Cerebrovascular Accident	Yes	No	10	10	3
138004-24-43	Ari 10 mg	Cerebral Ischemia	No	No	48	10	10
138004-75-161	Ari 10 mg	Intracerebral Hemorrhage	Yes	Yes	[ ]	10	28
138006-40-148	Ari 2-15 mg	Cerebral Ischemia	No	No	34	5	18

b(6)

Subject 138004-110-405 presented in a comatose state with an INR level of 5.9 while taking warfarin. No imaging studies were obtained so it could not be confirmed that hemorrhage caused the CVA. The subject with a confirmed diagnosis of intracerebral hemorrhage, 138004-75-161, was taking warfarin but did not have reported INR or other measures of anticoagulation.

Observations from Open-Label Studies

There were 36 subjects with CAEs in the sponsor's open label studies observing 894 patients over 648 patient-years, an incidence rate of 5.6 per 100 patient-years. All but 30 of these subjects had entered these studies after participating in placebo-controlled trials. Notably, one subject suffering a CVA during an open-label study was also taking prazosin.

Sponsors' Conclusions

The sponsors conclude that a potential signal exists for an increased incidence of Treatment Emergent Potential Cerebrovascular Adverse Events in the population of elderly patients with psychosis associated with Alzheimer's Disease. They note that this result is a consequence of 1 of 3 placebo-controlled trials and it was not statistically different from the incidence in the placebo-controlled population. All eight CAEs in the aripiprazole group occurred in the presence of a history of CVA/stroke or other stroke risk factors; no events occurred in the absence of these factors. They request an update to the label in the Precautions Section. The sponsors also note updated all-cause mortality in this population was 3.5% for aripiprazole and 1.7% for placebo

Reviewer's Comments

Adverse Event Coding and Pooling of Data

The approach taken by the sponsors to identify cerebrovascular-related adverse events is acceptable. As a result of reviewing all adverse event verbatim terms using a string search, they appear to have effectively identified a number of events that were not covered by the preferred term search.

There are some potential problems with the pooling of data from the three studies. The population in CN138006 began the study as outpatients, had a smaller percentage of stroke risk factors, and was generally younger and less neurologically impaired. These differences could lead to lower risk for true events in the CN138006 study because subjects were at lower risk but a greater likelihood of detecting minor or false positive events because such events may be easier to detect in subjects who are less impaired. Combining these two populations runs the risk of diluting different signals from each of them. Although the populations in the CN138005 and CN138004 studies were similar in age, neurological impairment, and percentage of risk factors, differences in dosing protocols in the two studies (flexible dosing in CN138005 and fixed dosing in CN138004) could lead to very different adverse event profiles and other effects. In a flexible dosing regimen, subjects with similar disease severity will be titrated to dosages that produce similar pharmacologic effect while a fixed dosing regimen is more likely to show a true dose-response effect. Rather than pool the three trials together, it would be preferable to combine the observed effects of the three trials using a random effects meta-analysis technique.

Analysis of Results

Baseline Characteristics

Differences in baseline characteristics may have led to a small bias against aripiprazole; those subjects who received active drug were slightly more likely to have a history of stroke and other risk factors. As all of the subjects who experienced CAEs were women, this bias may have been offset by the lower proportion of women among those receiving active drug if female sex were a genuine risk factor.

In the CN138004 study, where a dose-response effect was observed, increasing dosages of aripiprazole correlated with increasing baseline MMSE, male sex, and a history of CHF. The first two factors were associated with no observed increased incidence of CAEs while the last is a weak risk factor for CAEs. Taken together, the imbalance of baseline characteristics in this study probably acted to diminish the probability of observing the dose-response relationship that was seen.

Incidence of Cerebrovascular Adverse Events

Combining the effects of the three trials using meta-analysis with a random effects model, the incidence rate ratio for CAEs from aripiprazole relative to placebo is 2.43 (95% CI: 0.23-25.35, p=0.46). This is, of course, not statistically significant and is entirely consistent with what we would expect to observe if there was no real difference. The data are also completely consistent with a substantially elevated risk; CAEs were simply too uncommon in the clinical trial experience to make any conclusions based solely on these data.

The more impressive observation is the statistically significant dose-response effect observed in the CN138004 study. Because subjects in this study were randomly assigned their dosages of aripiprazole (or to placebo), drug effect should not be confounded by pharmacokinetic differences or by indication as was possible in the other two trials.

Observations from Open-Label Studies

The incidence rate of CAEs observed in the open label studies was between those observed for the respective active and placebo groups in the controlled studies. None of these differences is statistically significant. Additionally, the population observed in the open label studies was subject to selection bias; almost all of the subjects entered the open-label phase after passing through a controlled trial without significant adverse experiences.

Note was made of one subject in the open label studies who suffered a CVA while taking prazosin. One postulated mechanism for an increase in CAEs with aripiprazole is hypotension resulting from the drug's adrenergic blocking effects. This effect may be magnified with the use of anti-hypertensive drugs, particularly an alpha-adrenergic blocker such as prazosin. Because hypertension itself is a risk factor for CAEs and most of the subjects experiencing CAEs had a history of hypertension and were taking anti-hypertensive drugs, it cannot be determined whether hypertension or the combination of aripiprazole with an anti-hypertensive drug was the cause of a CAE.

Sponsors' Conclusions

The sponsors correctly recognize that the slightly elevated incidence of CAEs with aripiprazole observed in the three studies combined is very weak evidence of a harmful effect from the drug. I would disagree with the implication that the absence of any increase in observed CAEs in two of the studies (CN138005 and CN138006) sheds doubt on the significance of what was observed in CN138004. The paucity of CAEs in both the

drug and placebo groups in the CN138005 and CN138006 studies means that these two studies provide little information as to whether the risk from aripiprazole is or is not elevated; almost all the meaningful information comes from CN138004. In particular, the lower risk for CAEs in the CN138006 population makes it unlikely that any significant (or even suspicious) difference would be observed with the number of patients involved. The differences in dosing practices between CN138004 and the other two studies could also significantly affect whether a signal would be observed. The sponsors overlook the significance of the dose-response effect observed in the fixed dose study, CN138004. Such an effect would be much less likely to be observed in the other two studies where dosages were titrated.

Comparison with Other Drugs in Its Class

A summary of the observed risks for CAEs with aripiprazole compared with olanzapine and risperidone is given in Table 8.

Table 8: Meta-Analysis of Rates for CAEs in Trials of Aripiprazole, Olanzapine, and Risperidone

	Type of Dementia	Incidence Rate (per 100 py)		Incidence Rate Ratio	95% Confidence Interval		p value
		Drug	Placebo		Lower	Upper	
Aripiprazole	Alzheimer	8.4	3.7	2.43	0.23	25.35	0.46
Olanzapine	Alzheimer	3.7	1.5	2.43	0.54	22.36	0.24
Risperidone	Alzheimer	15.3	9.0	2.60	0.78	8.72	0.12
All Alzheimer's Combined				2.54	1.01	6.43	0.05
All Trials and Dementia Types Combined				3.24	1.47	7.14	0.004

While the observed risk ratio for aripiprazole has very wide confidence intervals and is, by itself, highly consistent with chance, these findings should be analyzed in the context of the findings for olanzapine and risperidone. All three drugs have alpha$_1$-adrenergic blocking activity, suspected to cause hypotension and diminished cerebral perfusion leading to CAEs. The observed risk ratios for all three drugs in Alzheimer's Dementia are remarkably similar. Differences in incidence rates appear to be due to differences in risk for the selected patient populations as reflected in the different incidence rates among the placebo groups. From a Bayesian perspective, the observed results for olanzapine and risperidone constitute a prior expectation for aripiprazole. The observed aripiprazole results are strongly consistent with that expectation. The posterior distribution is the same as that resulting from the combination of all of these studies using meta-analysis. The combined results for Alzheimer's patients over all trials gives a ratio (2.54) that has statistical significance with no evidence for heterogeneity (p=0.932).

The clinical trials in dementia for olanzapine and risperidone included patients with vascular or mixed dementia as well as Alzheimer's without stratifying for type of dementia. Combining all trials and including all subjects with dementia of any etiology

gives an overall ratio of 3.24 that is statistically significant without evidence for heterogeneity (p=0.856).

Reviewer's Conclusions

There is good evidence for an increased risk for CAEs from aripiprazole based upon three factors:

- the presence of a dose-response effect
- remarkably similar findings with other drugs in its class
- a plausible mechanism: hypotension from adrenergic blockade

One factor that may possibly mitigate this risk is the complete lack of a signal for increased risk in the flexible dosing studies. While chance is a plausible explanation for these differences, it is possible that careful titration of dosage reduces any risk, something not possible in a fixed dose study. At the same time, the dosage titration done under protocol in the clinical trials may be much more careful than what might be seen in typical medical practice.

Labeling addressing the risk of CAEs with aripiprazole should be similar to that for olanzapine and risperidone. The excess risk for CAEs associated with aripiprazole and other drugs in its class appears to be proportional to the patient's underlying risk; the greatest caution should be exerted for patients at highest risk for CAEs.

Appears This Way
On Original

This is a representation of an electronic record that was signed electronically and this page is the manifestation of the electronic signature.

/s/

Marc Stone
11/22/04 12:26:54 PM
MEDICAL OFFICER

Judith Racoosin
11/22/04 02:27:51 PM
MEDICAL OFFICER

CLINICAL REVIEW

Application Type	sNDA
Submission Number	21-436
Submission Code	SE1-005
Letter Date	January 28, 2004
Stamp Date	January 30, 2004
PDUFA Goal Date	November 30th, 2004
Reviewer Name	Teresa A. Podruchny
Review Completion Date	December 07, 2004
Established Name	Aripiprazole
(Proposed) Trade Name	Abilify
Therapeutic Class	Anti-psychotic
Applicant	BMS-Otsuka
Priority Designation	S
Formulation	Oral
Dosing Regimen	daily
Indication	Maintenance Mania
Intended Population	Bipolar I Disorder

Table of Contents

1 EXECUTIVE SUMMARY 4

1.1 RECOMMENDATION ON REGULATORY ACTION 4
1.2 RECOMMENDATION ON POSTMARKETING ACTIONS 4
1.2.1 Risk Management Activity 4
1.2.2 Required Phase 4 Commitments 4
1.2.3 Other Phase 4 Requests 4
1.3 SUMMARY OF CLINICAL FINDINGS 5
1.3.1 Brief Overview of Clinical Program 5
1.3.2 Efficacy 5
1.3.3 Safety 6
1.3.4 Dosing Regimen and Administration 6
1.3.5 Drug-Drug Interactions 7
1.3.6 Special Populations 7

2 INTRODUCTION AND BACKGROUND 7

2.1 PRODUCT INFORMATION 7
2.2 CURRENTLY AVAILABLE TREATMENT FOR INDICATIONS 7
2.3 AVAILABILITY OF PROPOSED ACTIVE INGREDIENT IN THE UNITED STATES 8
2.4 IMPORTANT ISSUES WITH PHARMACOLOGICALLY RELATED PRODUCTS 8
2.5 PRESUBMISSION REGULATORY ACTIVITY 8
2.6 OTHER RELEVANT BACKGROUND INFORMATION 9

3 SIGNIFICANT FINDINGS FROM OTHER REVIEW DISCIPLINES 9

4 DATA SOURCES, REVIEW STRATEGY, AND DATA INTEGRITY 10

4.1 SOURCES OF CLINICAL DATA 10
4.2 TABLES OF CLINICAL STUDIES 10
4.3 REVIEW STRATEGY 10
4.4 DATA QUALITY AND INTEGRITY 10
4.5 COMPLIANCE WITH GOOD CLINICAL PRACTICES 12
4.6 FINANCIAL DISCLOSURES 12

5 CLINICAL PHARMACOLOGY 13

6 INTEGRATED REVIEW OF EFFICACY 13

6.1 INDICATION 13
6.1.1 Methods 13
6.1.2 General Discussion of Endpoints 13
6.1.3 Study Design 14
6.1.6 Efficacy Conclusions 25

7 INTEGRATED REVIEW OF SAFETY 26

7.1 METHODS AND FINDINGS 26
7.1.1 Deaths 26
7.1.2 Other Serious Adverse Events 27
7.1.3 Dropouts and Other Significant Adverse Events 27
7.1.4 Other Search Strategies 28
7.1.5 Common Adverse Events 28
7.1.6 Less Common Adverse Events 31
7.1.7 Laboratory Findings 31
7.1.8 Vital Signs 33

7.1.9 Electrocardiograms (ECGs) 34
7.1.12 Special Safety Studies 35
7.1.13 Withdrawal Phenomena and/or Abuse Potential 35
7.1.14 Human Reproduction and Pregnancy Data 35
7.1.15 Assessment of Effect on Growth 35
7.1.16 Overdose Experience 35
7.1.17 Postmarketing Experience 35
7.2 ADEQUACY OF PATIENT EXPOSURE AND SAFETY ASSESSMENTS 36
7.2.1 Description of Primary Clinical Data Sources (Populations Exposed and Extent of Exposure) Used to Evaluate Safety 36
7.2.4 Adequacy of Special Animal and/or In Vitro Testing 40
7.2.5 Adequacy of Routine Clinical Testing 40
7.2.6 Adequacy of Metabolic, Clearance, and Interaction Workup 40
7.2.7 Adequacy of Evaluation for Potential Adverse Events for Any New Drug and Particularly for Drugs in the Class Represented by the New Drug; Recommendations for Further Study 40
7.2.8 Assessment of Quality and Completeness of Data 40
7.2.9 Additional Submissions, Including Safety Update 41
7.3 SUMMARY OF SELECTED DRUG-RELATED ADVERSE EVENTS, IMPORTANT LIMITATIONS OF DATA, AND CONCLUSIONS 41
7.4 GENERAL METHODOLOGY 41
7.4.1 Pooling Data Across Studies to Estimate and Compare Incidence 41
7.4.2 Explorations for Predictive Factors 42

8 ADDITIONAL CLINICAL ISSUES 42

8.1 DOSING REGIMEN AND ADMINISTRATION 42
8.2 DRUG-DRUG INTERACTIONS 42
8.6 LITERATURE 43

9 OVERALL ASSESSMENT 43

9.1 CONCLUSIONS 43
9.2 RECOMMENDATION ON REGULATORY ACTION 43
9.3 RECOMMENDATION ON POSTMARKETING ACTIONS 43
9.3.1 Risk Management Activity 43
9.3.2 Required Phase 4 Commitments 44
9.5 COMMENTS TO APPLICANT 44

10 APPENDICES 45

10.1 REVIEW OF INDIVIDUAL STUDY REPORTS 45

1 EXECUTIVE SUMMARY

1.1 Recommendation on Regulatory Action

I recommend the Division consider an approvable action on this supplement. While the p value is significant when comparing aripiprazole to placebo for time from randomization to relapse in the maintenance phase, it is unclear to me that this reflects efficacy of the drug. While the larger pool of data favors aripiprazole, removal of one site in Mexico (site 93) causes the study to lose significance. This site appears to have a different relapse rate than the conglomerate U.S. sites. DSI inspection at this site revealed protocol violations, however, overall the data were deemed acceptable. As this is the only study for maintenance and given that a large number of U.S. sites were involved but alone are not powered to show significance and for other reasons listed within this review, I recommend we ask for further exploration of the data in this study with attention to the Mexican sites, more so to site 93.

1.2 Recommendation on Postmarketing Actions

1.2.1 Risk Management Activity

I recommend addition of PE/DVT to the postmarketing list as well as consideration of addition of the events of hypersensitivity, hepatobiliary events, and increased creatine phosphokinase/rhabdomyolysis.

A change to the label regarding the risk of cerebrovascular adverse events in elderly patients with dementia has been added as a WARNING. This is based on a recent review by Dr. Marc Stone in DNDP. Additionally, the OVERDOSAGE/Human Experience subsection currently is under review in SLR007.

1.2.2 Required Phase 4 Commitments

Required Phase 4 commitments were delineated in the action letter for this supplement. As commitments were made regarding adult studies to address short and longer term efficacy as add-on therapy in bipolar patients and pharmacology-toxicology studies needed to support pediatric trials with the action on supplement 002 (acute mania), no additional studies are required at this time. However, the sponsor was asked to state a date of submission of the clinical study reports for the recently completed drug interaction studies.

1.2.3 Other Phase 4 Requests

There are no other phase 4 requests at this time.

1.3 Summary of Clinical Findings

1.3.1 Brief Overview of Clinical Program

Aripiprazole was approved in the United States for the treatment of schizophrenia on November 15, 2002 and for the treatment of acute manic and mixed episodes on September 29, 2004.

[ ]
[ ] b(4)

1.3.2 Efficacy

Study CN138010 is the pivotal study submitted to support the indication of maintenance of stability in Bipolar I Disorder. Study CN138010 was a double-blind, placebo-controlled trial conducted at multiple U.S. sites and several sites outside of the U.S. The trial consisted of an open-label aripiprazole stabilization phase, a double-blind randomized withdrawal phase entered after meeting time and scale criteria in stabilization, and a continuation double-blind extension phase.

633 patients enrolled in the study and 196 patients were randomized. However, the efficacy maintenance dataset is comprised of 161 patients as 35 randomized patients received unblinded medication and were discontinued. The primary efficacy measure was time from randomization to relapse in the maintenance phase for aripiprazole treated versus placebo treated patients. Key secondary measures were time to manic and time to depressive relapse analyzed using a hierarchical procedure.

CN 138010 data demonstrated significance on the primary efficacy measure (p=0.02). One site in Mexico (site 93) appears to have both a low relapse rate in the aripiprazole group and a high relapse rate in the placebo group when compared to the conglomerate U.S. sites, which contain 77% of the patients in the study. The second site in Mexico (118), has low relapse rates in both the placebo and aripiprazole groups. Although the study is not powered to examine treatment by center nor for the U.S. sites to stand alone, when dropping the other large site in Mexico (118), the study does not lose significance. However, removal of site 93 causes the study to lose significance.

Admittedly, this is post-hoc analysis. DSI noted protocol violations however, as a whole the data were not felt to be globally unacceptable although a limitation of the data was that the source documents were in Spanish. It is unclear to me whether the results (site 93) represent a spectrum of the efficacy of this drug or reflect an aberrant finding at this site that is not generalizable. For reasons outlined in the body of this review, I recommended an approvable action with further exploration of the data regarding the robustness of the p-value.

The study is not fixed dose and cannot assess dose response.

1.3.3 Safety

The safety data to support use in the maintenance treatment of mania are derived primarily from study CN138010. Quantitative safety review with respect to EKG data, vital signs, and clinical laboratory measures was limited secondary to the design of this study. The data were reviewed for deaths, non-fatal serious adverse events, and discontinuations secondary to adverse events. Akathisia appears to remain a common adverse event in this population. CPK elevations were the cause of discontinuation of two patients in the open label phase.

Quality control review of lists of protocol violations and the Division of Scientific Investigation report indicate missed laboratories or EKGs and the sponsor submitted data from source documents at site 118 that were not included in the CRFs (email 8-12-04). The data from the 35 randomized patients who received unblinded study medication were presented separately from the randomized population. An audit of the COSTART terms was performed. Recommendations to the sponsor resulting from this audit are made in section 9.5.

Review of deaths, non-fatal serious adverse events, and discontinuations secondary to adverse events and review of adverse event terms for this trial as per the JMP dataset did not reveal any new, previously undescribed adverse events for the bipolar population such as to preclude approval for this indication.

Non-bipolar indications:

- Review of incidence data for other indications, as supplied in incidences table in the ISS of this supplement and in supplement 002 submissions, was not performed as these tables are not exposure and placebo adjusted.
- Line listings of patients who died, experienced a non-fatal serious adverse event, or discontinued secondary to an adverse event in studies blinded or newly reported since September, 2002 were submitted with supplement 002, the 120 day safety update, the response to the approvable, and supplement 005. Line listings of the deaths generally do not include the cause of death.
- Review of these line listings was cursory. Review of events that require further exploration is in progress but these data are not discussed in this review and will be completed as per Division leadership advisement.

Post-marketing data are discussed in section 7 of this review.

1.3.4 Dosing Regimen and Administration

Study CN138010 was not a fixed dose study. In this study, dosing started in the stabilization phase at 30 mg daily with reduction to 15 mg daily as tolerated or efficacious. During the maintenance phase, the initial dose of aripiprazole was the end dose at stabilization and could be adjusted as necessary for either efficacy or tolerability issues.

The mean daily dose at the endpoint of stabilization was 25.25 mg daily. For those who completed this phase and remained eligible for maintenance, the mean daily dose was similar at

24.39 mg/day. The mean daily dose of aripiprazole for patients at endpoint (n=82) of the maintenance phase was 24.29 with a range of about 13 to 30 mg per day.

1.3.5 Drug-Drug Interactions

b(4)

With regard to other drugs used in the treatment of bipolar disorder, the label included with this submission (1-28- 04) contains information on valproate, lithium, and carbamazepine. As per this label, no dosage adjustment of aripiprazole is required when concomitantly administered with either valproate or lithium. However, if carbamazepine is added to aripiprazole treatment, it is recommended that the dose of aripiprazole be doubled and in converse, if carbamazepine therapy is withdrawn from combination therapy, the dose of aripiprazole should be reduced.

1.3.6 Special Populations

No additional studies in special populations were submitted with this application.

Cerebrovascular adverse events in dementia patients were reviewed by Dr. Marc Stone of the DNDP safety team. Although not labeled for this indication, additional language to the label will be added as a warning for this group.

2 INTRODUCTION AND BACKGROUND

2.1 Product Information

Aripiprazole, Abilify™, is an atypical antipsychotic approved in the U.S. for use in the treatment of schizophrenia and acute manic and mixed episodes associated with Bipolar Disorder. It is a partial D_2 agonist acting as an agonist in an animal model of dopaminergic hypoactivity and an antagonist in animal models of dopaminergic hyperactivity. Aripiprazole also is a 5-HT_{1A} partial b(4)
agonist and a 5-HT[ ] antagonist.

2.2 Currently Available Treatment for Indications

Drugs approved for monotherapy in the maintenance treatment of Bipolar Disorder include lithium and lamotrigine. The only atypical antipsychotic currently approved is olanzapine, which received approval for two weeks of maintenance treatment, in January of 2004. The combination of olanzpine and flouxetine (Symbyax™) is approved for use for up to eight weeks in the treatment of depressive episodes of bipolar depression and lamotrigine (Lamictal®) is

approved for use as maintenance monotherapy of bipolar depression and mania in bipolar disorder, although it is not approved for acute episodes of bipolar disorder.

Mutiple agents are used off label for maintenance treatment as either mono or adjunctive therapy. These include carbamazepine, oxcarbamazepine, valproate, other atypical antipsychotics and gabapentin and topirimate.

2.3 Availability of Proposed Active Ingredient in the United States

Aripiprazole, Abilify™, is available in the U.S. It was approved in the U.S. for use in the symptomatic treatment of schizophrenia on November 15, 2002 and for acute manic and mixed episodes associated with Bipolar Disorder on September 28, 2004.

Aripiprazole carries warnings for neuroleptic malignant sydrome, tardive dyskinesia, and hyperglycemia and diabetes. A warning for use in the psychosis of Alzheimer's Disease will be included with other label changes made during the review of this supplement. Aripiprazole precautions are for orthostatic hypotension, seizure, potential for cognitive and motor impairment, body temperature regulation, dysphagia, and suicide. (see section 2.4 for class labeling.)

2.4 Important Issues With Pharmacologically Related Products

Neuroleptic malignant syndrome, tardive dyskinesia, and hyperglycemia and diabetes mellitus are labeled "WARNINGS" for atypical antipsychotics. Aripiprazole will soon receive a "WARNING" for cerebrovascular adverse events in the psychosis of dementia. Olanzapine and risperidone already have such language.

Other "WARNINGS" on individual atypicals include:

- Clozapine- Black box warnings for agranuloctyosis, seizures, myocarditis, other adverse cardiovascular and respiratory effects (including collapse, respiratory arrest, and cardiac arrest during initial treatment). There is a required hematologic monitoring program in place for the prescribed use of this product.
- Ziprasidone carries an additional warning for QT prolongation and sudden death.
- Quetiapine carries an additional bolded "PRECAUTION" for cataract development seen in animal studies and recommends monitoring for cataract development.
- Olanzapine carries and additional warning for a higher incidence of death in dementia-related psychosis treated with olanzapine versus those treated with placebo, although the drug is not approved for use in this population.

2.5 Presubmission Regulatory Activity

September, 1999- BMS and Otsuka entered a co-development agreement with respect to the development of aripiprazole. This resulted in a program that allowed for additional indications beyond schizophrenia.

February, 2000- BMS/Otsuka and the Division of Neuropharmacological Drug Products (DNDP) met to discuss the planned development for indications other than schizophrenia which included development for acute mania and a bipolar disorder relapse prevention study.

October 31, 2001- the original NDA was submitted for the indication of schizophrenia only as one of the key bipolar studies did not show efficacy on the primary efficacy variable.

May 9, 2003- a pre-sNDA meeting was held to discuss the acute mania program and submission.

June 23, 2003- [ ] suppmental NDAs were submitted for acute bipolar mania b(4)

December 5, 2003- a pre-sNDA meeting was held with Otsuka/BMS to discuss the content and format of the maintenance treatment supplement [ ] b(4)
[ ] The design of study CN138010 was discussed. DNDP noted that the study design would support some additional labeling. However, DNDP expressed that the duration of the open-label stabilization phase defines duration of effect and noted that an optimal study design would include a six month open-label stabilization phase and randomized withdrawal of patient subgroups at specified timepoints. Additionally, the timing of safety updates was discussed as were safety data for other indications.

January 28, 2004- the current supplemental NDA was submitted for the use of aripiprazole in maintaining stability in patients with Bipolar I Disorder.

2.6 Other Relevant Background Information

Aripiprazole is approved for marketing for the treatment of symptoms of schizophrenia (acute and maintenance or acute) in multiple countries including Brazil, [ ] Puerto Rico, Australia, Peru, Korea, [ ] and Mexico. It has received approval b(4)
for the treatment of acute manic and mixed episodes of bipolar disorder in [ ]

3 SIGNIFICANT FINDINGS FROM OTHER REVIEW DISCIPLINES

Statistical review through the review of the Office of Biometrics was performed by Dr. Kun He and is discussed in the efficacy section of this review (section 6.1.4).

The Division of Scientific Investigations conducted reviews of three sites. The report was authored by Dr. Ni Khin and is discussed in section 4.4 Data Quality and Integrity of this review and in the efficacy section of this review (section 6.1.4).

4 DATA SOURCES, REVIEW STRATEGY, AND DATA INTEGRITY

4.1 Sources of Clinical Data

CN 138010, a single multi-center double-blind, randomized pivotal trial was submitted in support of this indication. Open label safety data from trial CN138037 was reviewed for deaths and non-fatal serious adverse events.

4.2 Tables of Clinical Studies

Trial	Dosing Type	Design	Phases	Dosing	Patient numbers
CN138037	Flexible Dose	Open label	Stabilization, Maintenance, Extension phases	Aripiprazole 15 and 30 mg	25 entered/ 24 treated
*CN138010	Flexible	Placebo-controlled randomized phase(s)	Open-label stabilization, randomized withdrawal maintenance phase, and extension of continued double-blind	Stab: Maint: 15 to 30 mg or placebo	Stab: 635 entered/552 treated<br>Maint: 196 entered. 35 dc'd sec to unblinding. 161 randomized<br>U.S. sites (76.9%)<br>Mexico (18.8%)<br>Argentina (4.4%)

*Submitted in support of this indication. CN138037 was reviewed for deaths and non-fatal serious adverse events only.

4.3 Review Strategy

The Clinical Study Report for CN138010, JMP files submitted with this supplement as needed, updated appendices submitted 8-27-04, the ISS for this supplement with attention focused on maintenance mania, and narratives, and case report forms were utilized in the preparation of this document. Safety data for study CN138037 was taken from the CSR as supplied with the submission for supplement 002 on 6-23-04.

4.4 Data Quality and Integrity

There were several issues relative to data quality and integrity
- 35 patients treated unblinded initially and
- the findings of the DSI inspection.

35 patients were randomized into study CN138010, across several sites, and received unblinded medications. When the sponsor became aware of the problem, randomization was closed and

ongoing patients discontinued. The Division discussed the circumstances of this with the sponsor. The explanations offered as to how data were handled were considered generally acceptable.

The Division of Scientific Investigations (DSI) inspected three sites; one U.S. site (64) and two sites in Mexico (93 and 118). These sites were chosen due to either sample size or impact on study significance. Dr. Ni Khin's report of these inspections notes that site 64 was classified as "minor deviations, data acceptable" (VAI). Site 93 and 118 were classified as having deviation(s) from regulations, response received and reviewed (VAI-RR).

- Site 93 screened and enrolled 18 patients and randomized thirteen patients. Records from all 18 subjects at this site were audited. Dr. Khin noted seven specific patients who either did not receive lithium/divalproex levels pre-randomization or had these laboratory collections after randomization and eight patients who were in the open-label stabilization phase after meeting criteria for randomization. Four of these were due to the sponsor being unable to supply blinded medication. Dr. Khin's review notes there were "multiple instancesof protocol required clinical laboratory tests" that were not performed, ranging from one to fifteen per subject. Dr. Khin also note that adverse events were not reported to the sponsor on two subjects and that there were several instances when events documented in the source document did not match the CRF.

- Site 118 screened 28 subjects and enrolled 25. Temperatures for storage of the medication were outside of recommended ranges (stored at 3°C-28°C versus 15-25°C). . During stabilization, four patients received lorazepam outside of protocol specifications. Safety data problems included two subjects who experienced serious adverse events that were not reported for several weeks and sixteen of seventeen EKGs reported missing in the clinical study report (CSR) for the stabilization were later recovered by the sponsor when querying the data differently.

Given the importance of the data at site 93, an internal meeting was held with Dr. Khin to discuss the inspection results and data integrity at site 93. It was my interpretation from this internal discussion that there was no obvious major problem nor the appearance of fraud such as to disqualify all data. However, it appeared the investigators may have been inexperienced in the conduct of clinical trials.

An audit of safety data was conducted by comparing CRFs, narratives, and line listing data for a sample of patients for internal consistency. With the exception of the selection of more patients at site 93, patients were randomly selected for audit.

The patients who were audited are listed in the appendix of this document. In the comparison of CRF data to narratives to line listing, most were acceptable although there were some discrepancies and two CRFs at site 93 were corrected several times (93-184, and 93-504).

- Patient 132-355: the CRF describes the serious adverse event as manic reaction and suicidal ideation. Suicidal ideation is not captured in the line listing (App. 12.1.A) and is not discussed in the narrative.

- Patient 146-437: this patient was coded as discontinuation secondary to an adverse event during stabilization on the CRF. The narrative notes he was discontinued secondary to a severe manic episode with psychotic features. While both may be correct, it appears this patient may have had lack of efficacy.
- Patient 64-441 is listed in the line listing as discontinuation due to an adverse event and the narrative concurs. The CRF captured this discontinuation as a withdrawal of consent.
- Patient 146-459: the narrative and text were not in agreement as to the reason for discontinuation. The sponsor was asked to clarify and noted the narrative was "incomplete".

4.5 Compliance with Good Clinical Practices

The sponsor notes that the study was conducted in accordance with Good Clinical Practice and with generally accepted standards for the protection of patient safety and welfare including the Declaration of Helsinki and amendments. Otsuka America certified that it had not used the services of any person listed as debarred as of the Date of Debarment List in connection with the application.

4.6 Financial Disclosures

The sponsor notes that [redacted] investigator Financial Disclosure Forms were received by November 17, 2003 and that no investigators had information to disclose. Investigators at sites [redacted] appear to have submitted disclosure forms and had nothing to disclose. (A list of the investigators in study CN138010 may be found in the appendix of this document.) b(6)

Of the 938 subinvestigator forms, 909 were returned. One subinvestigator, [redacted] M.D., of sites [redacted], received $18, 500 in 1999, $27,837 in 2000, $1414 in 2001, and $2350 in 2002. These monies included funding for a [redacted] study and honoraria fees. As this was a blinded, randomized, study, it is unlikely that these payments biased the study conduct such as to disqualify the data. 29 responses had not been received as of the date of writing the original submission document. b(6)

Otsuka submitted a certification as an applicant submitting the study that due diligence had been exercised to obtain financial information from non-responders. BMS submitted certification that as the sponsor of the study, they had not entered in to any financial arrangement with the listed clinical investigators in which the compensation to the investigator could be affected by the outcome of the study as per 21 CFR 54 and that any investigator who was required to disclose did not disclose any such interests.

5 CLINICAL PHARMACOLOGY

b(4)

The ISS for the maintenance supplement included a section on safety experience in clinical pharmacology studies. In this ISS, the sponsor notes data from 129 patients were analyzed for deaths, serious adverse events (SAEs), and discontinuations due to adverse events. The sponsor reports there were no deaths or SAEs in the clinical pharmacology studies. The lists of patients who discontinued secondary to an adverse event appears identical to the one in the acute mania 120-day update with the exception of one additional patient who discontinued secondary to vomiting.

6 INTEGRATED REVIEW OF EFFICACY

6.1 Indication

With regard to primary efficacy, the sponsor seeks the following claim, "The efficacy of ABILIFY in maintaining stability in patients with Bipolar I Disorder with a recent manic or mixed episode, was demonstrated in a double-blind, placebo-controlled, 6-month maintenance phase of a longer-term trial."

6.1.1 Methods

Study CN138010, *"A Multicenter, Randomized, Double-Blind, Placebo-Controlled Study of Aripiprazole in the Maintenance Treatment of Patients with Bipolar Disorder"* is the pivotal and sole efficacy study for this indication.

6.1.2 General Discussion of Endpoints

The primary efficacy measure was "time to relapse" (ie, discontinuation due to lack of efficacy) during the Maintenance Phase and was evaluated on the maintenance safety sample. The log rank test was used to compare the survival distributions of the two treatment groups with estimated survival curves obtained from Kaplan-Meier estimates. Discontinuation due to lack of efficacy was defined as either hospitalization for manic or depressive symptoms or requiring an additional medication or an increase in the allowed psychotropic medications.

Key secondary endpoints were time to manic and time to depressive relapses. These analyses were performed on the efficacy sample. A hierarchical testing procedure was employed with time to manic relapse tested first after the primary analysis, then time to depressive relapse.

6.1.3 Study Design

This was a randomized, double-blind, multi-center, placebo-controlled trial of aripiprazole for the maintenance of stability of patients meeting DSM-IV criteria for Bipolar I disorder. Patients were in- or outpatients who were from either a recently completed 3-week acute mania study of aripiprazole or were eligible for one of the 3-week studies but declined, or patients who were not from one of these studies but with a recent (≤ 3 months) manic or mixed episode requiring hospitalization and treatment. Patients who did not enter from an acute study participated in a screening period of up to 28 days before stabilization. For these patients, all antipsychotics and psychotropics outside of the prescribed protocol medications were discontinued with a minimum one day wash out for antipsychotics.

The study consisted of an open-label stabilization phase of 6-18 weeks, a blinded, randomized maintenance phase of up to 26 weeks, and a blinded extension phase of an additional 74 weeks. During open-label stabilization, visits occurred every two weeks. Eligibility for randomization required meeting both time (minimum 6 weeks) and scale criteria (YMRS and MADRS criteria of ≤ 10 and ≤ 13 respectively for 4 consecutive visits).

In the maintenance phase, patients assigned to aripiprazole received the same dose of drug as they were taking at the end of the stabilization phase. The dose could be adjusted for either efficacy or tolerability purposes. Patients who completed the maintenance phase without relapse were given the option to continue their current blinded study drug for an additional 74 weeks.

Complete **Inclusion criteria/exclusion** criteria for the study phases are included in the appendix of this document. Suicidal patients, patients requiring ECT in the previous 2 months, and patients likely to require additional prohibited medications were excluded from entering the stabilization phase.

Prohibited **concomitant medications** included carbamazepine, valproic acid, divalproate sodium, sodium valproate and lithium carbonate and citrate. Fluoxetine, long acting antipsychotics, other IND drugs, all other psychotropics, gingko biloba and St. John's were generally prohibited.

Allowed concomitant medications:
Lorazepam and anticholinergics for symptomatic EPS were allowed. Lorazepam was allowed in doses up to 6mg/day during screening and the first four weeks of stabilization, 3mg/day for the fifth week and 2 mg/day thereafter in stabilization. In the maintenance phase, lorazepam up to 2 mg/day during the first month, 1 mg/day during the second month, and 1 mg/day up to 4x weekly during the remaining 18 weeks was allowed. IM flunitrazepam and midazolam were allowed in Mexico and Brazil respectively when oral lorazapam was ineffective.

Anticholinergics for EPS were allowed for EPS symptom control in doses not to exceed 6mg/day equivalents of benztropine. No doses were to be given during the day before the baseline visit and 12 hours before either efficacy or safety rating scales.

6.1.4 Efficacy Findings

Six hundred and thirty-three patients (663) enrolled in the study. Of these, 567 entered the stabilization phase (333 from previous aripiprazole studies) and 206 completed the stabilization phase (37%). The most common reasons for discontinuation from the stabilization phase were adverse event (22%), lack of efficacy (12%), and withdrawal of consent (12%).

Of the 206 patients who completed the stabilization phase, 196 were randomized to the double-blind maintenance phase. However, only 161 are included for efficacy analysis as 35 patients across multiple sites received unblinded medication. Of the 161 maintenance phase patients, 58% discontinued (50% of the aripiprazole patients and 66% of the placebo patients). The most common reason for discontinuation in this phase in both groups was lack of efficacy (43% placebo, 24% aripiprazole).

The sponsor's table of disposition is duplicated from the CSR and included below.

Appears This Way
On Original

Table 8.1 Disposition of Patients

Patient Status	Number (%) of Patients Placebo	Aripiprazole	Total
**Enrolled**	n/a	n/a	633
**Baseline failures**	n/a	n/a	66
**Entered Stabilization Phase**	n/a	567	567
Discontinued Stabilization Phase	n/a	361 (64)	361 (64)
Adverse event[a]	n/a	126 (22)	126 (22)
Lack of efficacy	n/a	66 (12)	66 (12)
Subject withdrew consent	n/a	66 (12)	66 (12)
Subject unreliability	n/a	25 (4)	25 (4)
Lost to follow-up	n/a	49 (9)	49 (9)
Pregnancy	n/a	1	1
Death	n/a	1	1
Other known cause[b]	n/a	27 (5)	27 (5)
Completed Stabilization Phase	n/a	206 (37)	206 (37)
**Randomized to Double-Blind Treatment**[c]	83	78	161
Discontinued from Maintenance	55 (66)	39 (50)	94 (58)
Lack of Efficacy	36 (43)	19 (24)	55 (34)
Subject withdrew consent	6 (7)	6 (8)	12 (7)
Subject Unreliability	5 (6)	3 (4)	8 (5)
Adverse Event[a]	1 (1)	5 (6)	6 (4)
Lost to Follow-up	1 (1)	1 (1)	2 (1)
Missing	0	1 (1)	1 (1)
Other known cause[d]	6 (7)	4 (5)	10 (6)
Completed Maintenance Phase	28 (34)	39 (50)	67 (42)
**Entered Extension**	27	39	66
Discontinued from Extension	22 (81)	32 (82)	54 (82)
Lack of efficacy[e]	7 (26)	5 (13)	12 (18)
Subject withdrew consent	3 (11)	8 (21)	11 (17)
Subject unreliability	2 (7)	2 (5)	4 (6)
Lost to follow-up	0	1 (3)	1 (2)

Table 8.1 Disposition of Patients

	Number (%) of Patients		
Patient Status	Placebo	Aripiprazole	Total
Pregnancy	0	1 (3)	1 (2)
Adverse event[a]	0	1 (3)	1 (2)
Other known cause[f]	10 (37)	14 (36)	24 (36)
Completed Extension Phase	5 (19)	7 (18)	12 (18)

Protocol CN138010
Source: Appendix 8.1A

[a] Data obtained from end-of-study CRF page.

[b] During the Stabilization Phase, "other known causes" included such things as screen failure, positive drug screen, did not meet inclusion criteria, and site closed by sponsor because prespecified number of relapses had been attained. In addition, 1 patient discontinued because of an SAE (thought suicidal) and was included in this category.

[c] Forty-six patients completed the Stabilization Phase: 35 patients were randomized to the double-blind Maintenance Phase but were discontinued because of a labeling error; 11 patients discontinued because of other reasons (eg, Y-MRS or MADRS criteria not met for randomization, reason not stated) and were not randomized to the double-blind Maintenance Phase; and 1 patient (Patient 138010-141-266) did not complete the Stabilization Phase but was randomized in error to double-blind treatment.

[d] During the Maintenance Phase, "other known causes" included positive drug screen, patient relocating, and site closed by sponsor because prespecified number of relapses had been attained.

[e] Patient 138010-147-604 relapsed during the Extension Phase, according to the relapse form, but discontinued from the Extension Phase because of "other known cause" according to the end-of-study form.

[f] During the Extension Phase, the primary "other known cause" (study closed by sponsor because prespecified number of relapses had been attained)

Reasons of discontinuation:
Table S.8.1 of the submission (not included in the appendix of this document) listed comments for patients who discontinued secondary to "withdrew consent" or "Other known cause". Under "withdrawal of consent", some of the comments include the subject feeling conventional therapy would have greater symptom reduction (7-278), perceived adverse events (13-557), starting a new job (16-272), and "hospitalized without previous 'advice' to the investigator and withdrew consent" (93-126). "Other" also represented many reasons including the patients terminated due to unblinding, positive drug screen, and failing to meet criteria.

Information in Table S.8.1 indicates that some of these events could have been better classified such as patient 92-136, who was noted to have a serious adverse event but was coded as "Other", patient 6-142, also coded as "Other" is listed as making suicidal threats, and patient 10-273, coded as "withdrawal of consent" is noted to have increased depression and diarrhea.

Efficacy Data: Figure 10.1, Table 10.1A, and Table 10 A (excerpted) display efficacy data and are copied from the submission below. A sponsor provided graph of the impact of censoring is included in the appendix of this document.

Figure 10.1: Time from Randomization to Relapse, Maintenance Safety Sample

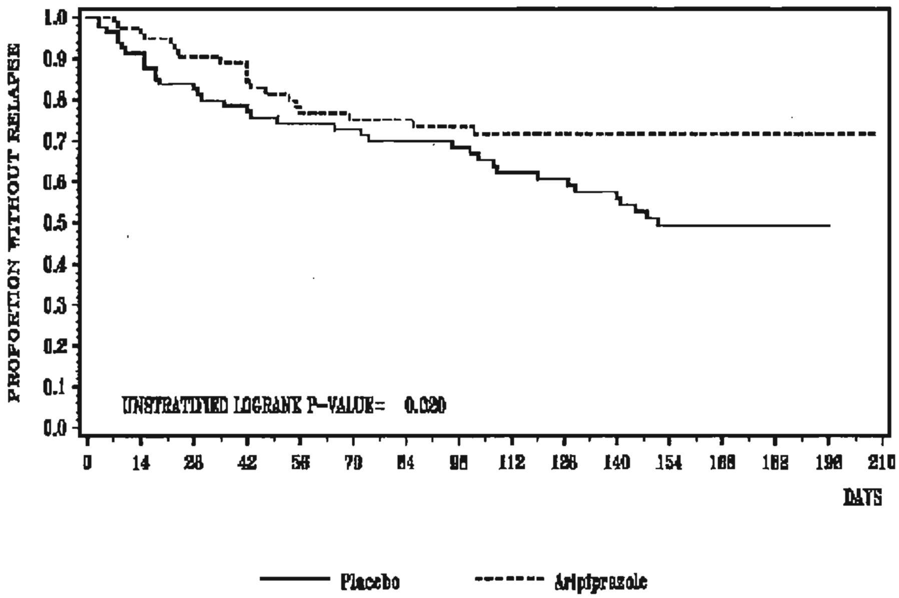

SUBJECTS AT RISK																
Placebo	83	74	62	56	53	52	48	45	41	39	35	30	30	25	1	0
Aripipra	77	75	61	58	50	47	46	43	42	41	40	40	40	36	2	0

HAZARD RATIO Aripiprazole OVER Placebo : 0.523 (95% CI= 0.300- 0.913)

Appears This Way
On Original

Table 10.1A Time from Randomization to Relapse, Maintenance Safety Sample

Time from Randomization to Relapse[a,b]				
Log-rank test p-value for equality of survival curves			0.020	
Hazard ratio (Aripiprazole:Placebo) , 95% CI[c]			0.523 (0.300, 0.913)	
**Patients Not Experiencing Relapse**				
	**Placebo**		**Aripiprazole**	
**Study Week**	**Number at Risk**	**Proportion[d] (SE)[e]**	**Number at Risk**	**Proportion[d] (SE)[e]**
0	83	1.00 (0.00)	77	1.00 (0.00)
1	74	0.91 (0.03)	75	0.97 (0.02)
2	71	0.88 (0.04)	73	0.95 (0.03)
3	64	0.84 (0.04)	61	0.91 (0.03)
4	59	0.80 (0.05)	58	0.89 (0.04)
6	54	0.76 (0.05)	52	0.81 (0.05)
8	53	0.74 (0.05)	49	0.77 (0.05)
10	48	0.70 (0.05)	47	0.75 (0.05)
12	48	0.70 (0.05)	44	0.73 (0.05)
14	43	0.65 (0.06)	42	0.72 (0.06)
16	39	0.61 (0.06)	42	0.72 (0.06)
18	36	0.58 (0.06)	42	0.72 (0.06)
20	32	0.53 (0.06)	42	0.72 (0.06)
22	30	0.49 (0.06)	42	0.72 (0.06)
24	30	0.49 (0.06)	42	0.72 (0.06)
26	30	0.49 (0.06)	42	0.72 (0.06)

Protocol CN138010

Source: Appendix 10.1A

NOTE: Median time to relapse and 95% CIs were not reported, as they were not estimable in the aripiprazole group.

[a] Defined as discontinuation due to lack of efficacy.

[b] For Patients 138010-118-214 and 138010-147-604, who were randomized in error upon entry into the Stabilization Phase, time from randomization to relapse is measured from the first day of dosing in the Maintenance Phase.

[c] Cox's proportional hazards model. Hazard ratio = aripiprazole:placebo. A hazard ratio < 1 favors aripiprazole.

[d] Kaplan-Meier Estimated Survival Rates.

[e] SE using Greenwood's formula from PROC LIFETEST.

Table 10A Summary of Efficacy Results at Endpoint, LOCF Data Set, Maintenance Phase

	Treatment Group	
	Placebo	Aripiprazole
Variable	N=83	N=77
**PRIMARY EFFICACY ENDPOINT**		
**Time to relapse for any event**[a]		
Hazard ratio (95% CI)[b]	0.523 (0.300, 0.913)	
P-value[c]	0.020*	
**KEY SECONDARY ENDPOINTS**		
**Time to manic relapse**		
Hazard ratio (95% CI)[b]	0.309 (0.123, 0.774)	
P-value[c]	0.008**	
**Time to depressive relapse**		
Hazard ratio (95% CI)[b]	0.833 (0.345, 2.011)	
P-value[c]	0.684	
**OTHER EFFICACY ENDPOINTS**		
**Number of Relapses (%)**[d]	36 (43%)	19 (25%)*
Relative Risk (Aripiprazole:Placebo) (95% CI)[e]	0.569 (0.359, 0.902)	
**Number of manic relapses** (%)	19 (23%)	6 (8%)
**Number of depressive relapses** (%)	11 (13%)	9 (12%)
**Number of mixed relapses** (%)	5 (6%)	4 (5%)
**Number of relapses of unknown type** (%)	1 (1%)	0 (0%)
**Y-MRS**		
Mean Score at Last Stabilization Visit	2.06	2.55
(95% CI)	(1.51, 2.62)	(1.98, 3.13)
Mean Change at Week 26	7.50	3.42**
(95% CI)	(5.39, 9.61)	(1.23, 5.62)

Protocol CN138010
Source: Appendices 10.1A, 10.2A-1, 10.2A-2, 10.3.3, 10.3.4, 10.3.5, 10.3.6
**($p \leq 0.01$), * ($0.01 < p \leq 0.05$), compared with placebo
[a] Defined as discontinuation due to lack of efficacy
[b] Cox's Proportional Hazards model, aripiprazole:placebo. A hazard ratio < 1 favors aripiprazole.
[c] Log-Rank Test for equality of Kaplan-Meier survival curves.
[d] Statistical testing not done on specific relapse type.
[e] CMH General Association Test, aripiprazole:placebo. A relative risk < 1 favors aripiprazole.
[f] CGI-BP mania change score is from 1 (very much improved) to 7 (very much worse).
[g] CGI-BP depression change score is from 1 (very much improved) to 7 (very much worse).
[h] CGI-BP overall change score is from 1 (very much improved) to 7 (very much worse).

Time in open-label stabilization:
Table 2M:

	Placebo n=83	Aripiprazole n=77
Mean days	88.06 ± 32.23	89.70±44.29
Median days	85	84
mode	56	42
range	41-159	37-264
Time in stabilization		
29-42 days	4 (4.8%)	9 (11.7%)
43-56 days	19 (22.9%)	16 (20.8%)
57-70 days	6 (7.2%)	8 (10.4%)
71-84	11 (13.3%)	6 (7.8%)
85-98	12 (14.5%)	9 (11.7%)
99-112	10 (12.1%)	7 (9.1%)
113-126	9 (10.8%)	8 (10.4%)
127-140	9 (10.8%)	9 (11.7%)
141-154	2 (2.4%)	0

Data in this table is excerpted from the sponsor's table (Table 2) email response dated September 10, 2004. Table 2 is duplicated in the appendix of this document.

Table 3 displays time in stabilization IND versus Non-IND and is excerpted from a Table 3 as provided by the sponsor on September 10, 2004.

Table 3: Number of Patients Stabilized by Study Day Interval and Site IND Status, Maintenance Safety Sample

	IND Sites		Non-IND Sites[a]	
	Placebo N = 64	Aripiprazole N = 59	Placebo N = 19	Aripiprazole N = 18
Time in Stabilization	N (%)	N (%)	N (%)	N (%)
0 - 14 days	0	0	0	0
15 - 28 days	0	0	0	0
29 - 42 days	4 (6.3)	8 (13.6)	0	1 (5.6)
43 - 56 days	19 (29.7)	14 (23.7)	0	2 (11.1)
57 - 70 days	6 (9.4)	7 (11.9)	0	1 (5.6)
71 - 84 days	7 (10.9)	4 (6.8)	4 (21.1)	2 (11.1)
85 - 98 days	7 (10.9)	6 (10.2)	5 (26.3)	3 (16.7)
99 - 112 days	8 (12.5)	7 (11.9)	2 (10.5)	0
113 - 126 days	6 (9.4)	5 (8.5)	3 (15.8)	3 (16.7)
127 - 140 days	6 (9.4)	6 (10.2)	3 (15.8)	3 (16.7)

Concomitant therapy: During stabilization, the most common class of medication used concomitantly was the anxiolytics (51.5%). During maintenance, the most common class used concomitantly was the anxiolytics for the placebo group (46%) and the anxiolytics and anticholinergics and for aripiprazole group (39% each).

Potential Problems with the interpretation of the data:

- relapse rate and concomitant medication use

Relapse Rates:

There were 50 sites that randomized patients; 45 were IND U.S. sites and 5 were non-IND, non-U.S. sites. Of the five non-U.S. sites, three were in Argentina and two were in Mexico. The U.S. sites randomized 124/161 (77%) patients, Argentina 7/161(4%) and Mexico 30/161 (19%).

Dr. He performed the primary statistical review and concluded that the primary analysis log-rank test gave a p-value of .0199 with 36/83 relapsing in the placebo group and 19/77 relapsing in the aripiprazole group. His review notes that the relapse rates for the aripiprazole group in both Mexico and Argentina are lower than in the U.S. sites and that the Mexico rate is "extremely lower" compared to Argentina and the U.S. He noted that when data from site 93 is removed, the primary analysis is not significant (log rank p = .1043) and suggested consideration of the quality of the data at this site when making final decisions. As per his review,

Table 3.1.8.4.2 Relapse Rate by Center in Mexico

Center	Placebo		Aripiprazole	
	N	Relapsed	N	Relapsed
093	7	5 (71%)	6	0 (0%)
118	9	2 (22%)	8	1 (13%)

He also notes that baseline measures are balanced between the groups at site 93 and that one patient randomized to aripiprazole (93-533) actually received placebo. If recreating the above table using the patient's randomization code, the relapse rate in the placebo group would be 67% and 14% for the aripiprazole group.

It is noted that the relapse rate at site 118 is low for both aripiprazole and placebo groups when compared to the conglomerate U.S. site(s) (13% aripiprazole, 22% placebo versus 29%aripiprazole and 41% placebo). The placebo relapse rates for the combined sites in Mexico are about the same as the U.S. conglomerate, however, the aripiprazole relapse rates for the combined sites in Mexico are not similar to the U.S. rate.

Dr. He calculated time to relapse for the U.S. versus non-U.S. sites.

	IND		Non-IND	
	Aripiprazole n=59	Placebo n=64	Aripiprazole n=18	Placebo n=19
Mean # days	109 ± 74	101 ± 74	143 ± 69	115 ± 69
Median # days	102	99	138	129
mode	183	182	183	183
range	7-209	1-196	8-188	3-195

- Informal analyses to look for obvious demographic differences at baseline:

Dr. He performed several informal analyses at site 93 as there appeared to be an abnormally low relapse rate in the drug group and a higher one in the placebo group. These analyses were for baseline YMRS/MADRS scores at stabilization and pre-randomization and the time of randomization versus the time of eligibility for randomization. The latter exploration was to see whether there was a difference between site 93 and the U.S. sites in terms of when patients were randomized versus when they met criteria to be randomized and by how long. The latter analysis probably should be duplicated by the sponsor. The analyses for baseline scale scores did not yield obvious clinical differences that could be expected to differentially affect the outcome.

- **Concomitant Medication and Relapse:**

Nine patients from site 118 (5 placebo, 4 aripiprazole) and 3 from site 93 are listed in the appendix of prohibited or excessive concomitant medications or missing medication start or stop dates-maintenance phase (Appendix 7.3B amended). Given what appears to be a lower relapse rate in the drug group at these sites, I researched some of the patients in these lists. Of the three listed patients at site 93, two appear to have received the lorazepam in screening (as seen in the CRFs-stop date) and for the third, there is no CRF to verify.

For site 118, one could argue that as 5 patient were placebo and 4 aripiprazole, this might suggest that randomization would then basically "equal" out the effects of this type of error. As a site, taken in its entirety, dropping the entire site 118, does not render the study insignificant (log-rank p test value =.0206). One could also argue that, if some patients were maintained by the use of excessive medications, these protocol violators might represent relapse and assessment would need to be made on a case-by-case basis.

As defined by the protocol, relapse was "Patients were discontinued for lack of efficacy if they were hospitalized and/or required an addition to or increase in allowed psychotropic medication, other than study medication, for manic or depressive symptoms.". This was somewhat difficult when adverse events were not listed specifically as manic or depressive exacerbations and is second-guessing the researcher who was at the site. Examples of this are:

- Patient 118-97 was taking aripiprazole from June 6, 2001 to December 9, 2001 in the maintenance phase. This patient received lorazepam from August 15, 2001 to August 28, 2001 at 1 mg daily. This would appear to be a protocol violation as the protocol notes

that in the 3rd month of the maintenance phase, lorazepam can be used only for 1mg per day/ 4 days a week. There is no CRF for this patient but line listing of adverse events indicated "anxiety" during the time period of the lorazepam use.

- Patient 118-246 was taking aripiprazole in the maintenance phase from July 13, 2001 to January 10, 2002. From October 5, 2001 (week 12) to October 18, 2001, lorazepam at 3mg daily was used for "anxiety". The MADRS was 12 at week 12, which was increased from 0 at week 10. This is a protocol violation, whether it should have been a relapse is not clear.

- Patient 118-214 (placebo) was taking 1 mg daily lorazepam for about 3 weeks in maintenance beginning at week 12 (July 23 –August 19) for insomnia. This patient relapsed September 4, 2001. The use of lorazepam during this 3 weeks would appear to be a protocol violation.

- Patient 118-269 (placebo) is listed in appendix 7.3 B as taking lorazepam 1 mg daily for about 5 weeks in his/her 3rd and 4 month of the study (November 12-December 21). This is a protocol violation. There is no CRF but line listing notes "insomnia" as an AE during this time. This patient relapsed on January 4.

The appendix listing 7.3B is on-face rather confusing as even though the appendix is to include violators in the maintenance phase, sometimes the medication listed was given in the stabilization phase. Additionally, I was not able to reconcile the data on patient 118-148 as per the CRF and appendix 9.5.2 (by patient listing of concomitant medications) with that in appendix 7.3B.

Other efficacy related subgroup analysis:

The sponsor performed several subgroup type analyses of the data with regard to primary efficacy: episode type, gender and rapid cycler status. Dr. He's review notes that the study is not powered for subgroup analysis with respect to gender, race, and age. The following were provided by the sponsor:

- Episode type- 112 manic and 48 mixed. The log rank p was significant for the manic group (0.047) and not so for the mixed group (0.385).
- Gender analysis- 53 males and 107 females. The log rank p was significant for the females (0.065), not for the males (0.206).
- Cycling status- 28 rapid cycling patients and 132 non-rapid cycling. The log-rank test p value was significant for the rapid cycling group (0.033) and not the non-rapid cycling group (0.114).

6.1.6 Efficacy Conclusions

I am unsure how to interpret this study data and have some discomfort with the certain aspects of the data. Site 93 appears to have a low relapse rate in the aripiprazole group and a high one in the placebo group. Admittedly, this is found on post-hoc analysis, is difficult to interpret, and alone does not invalidate the results. Site 118 does not relapse many patients in either group (1/8 aripiprazole and 2/9 placebo) and due to this, it is perhaps not surprising that removal of this site, does not change the primary efficacy analysis.

It is possible that the data at site 93 is not aberrant and represents the spectrum of response to this drug. It is also possible that it is not a " real" finding. The DSI inspection report does not suggest disqualification of the entire site although the report notes the source documents were in Spanish and there were certain protocol violations at the site. In an internal group meeting with Dr. Khin, types of protocol violations seen at the site were discussed. At this meeting, when reviewed by types of violations, it was not clear these violations would have created a general bias against placebo and randomization would be expected to "protect" the integrity of the data.

I recommend we take an approvable action, define factors that could have affected the outcome at these sites, and ask the sponsor to demonstrate that these findings did not bias the outcome at this site. These factors might include investigator training and site monitoring (were the non-IND sites handled the same as IND sites?), demographics at baseline (type of episode, psychiatric history, in-patient or out patient status), the use and timing of concomitant anxiolytics, enrollment to randomization ratios, time in stabilization before randomization, and the number of patients who met criteria and were not randomized at that point and by how long. It might be helpful to have translation of all source documents.

It is my opinion that it is reasonable to consider asking the sponsor to re-examine the data because although the trend in the U.S. conglomerate site is in the same direction as the final efficacy result, this is the sole study to support maintenance monotherapy and

1) the study significance seems to depend on site 93. Dropping site 118, a larger site, does not make the study lose significance.
2) the patients in the randomized phase are already an enriched group (about 64% of the patients who start stabilization complete stabilization)
3) there were some quality control type violations at the sites inspected and quality control type issues involved in the initial conduct of this study in general with 35 patients receiving unblinded medications after randomization
4) the drug did not show efficacy on-face in time to relapse for depression
5) this class of drugs is associated with EPS (including akathisia), NMS, and TD.

7 INTEGRATED REVIEW OF SAFETY

7.1 Methods and Findings

This review of safety focused on the maintenance bipolar study, CN 138010. Another longer term but open-label study in the bipolar population, Study CN138037, was reviewed for deaths and non-fatal serious adverse events only. Quality control included DSI inspection and audits of CRFs and COSTART terms. Non-bipolar indications are discussed below. Post marketing discussion may be found in section 7.1.17.

With respect to study CN138010, safety assessments included adverse event reporting, measures of vital signs, EKGs, clinical laboratory tests, physical examinations, and body weight. The summary tables and analyses from the maintenance safety sample did not include data from the 35 patients who were treated with unblinded medications. Data from these 35 patients were reviewed for deaths and serious adverse events. One additional person excluded from the safety sample was a patient who became pregnant. This patient (141-266) was randomized but did not receive medication in the randomized phase.

Review of deaths, non-fatal serious adverse events, and discontinuations secondary to adverse events and review of adverse event terms for this trial as per the JMP dataset did not reveal any new or previously undescribed serious adverse events for the bipolar population.

Non-bipolar indications:

- Review of incidence data for other indications, as supplied in incidences table in the ISS of this supplement and in supplement 002 submissions, was not performed as these tables are neither exposure nor placebo adjusted.
- Line listings of patients who died, experienced a non-fatal serious adverse event, or discontinued secondary to an adverse event in studies blinded or newly reported since September, 2002 were submitted with supplement 002, the 120 day safety update, the response to the approvable, and supplement 005. As a point, the line listings of the deaths generally do not include the cause of death.
- Review of these listings was cursory. Review of events that require further exploration is in progress but these data are not discussed in this review and will be completed as per Division leadership advisement.

7.1.1 Deaths

There were two (2) deaths reported in study CN138010; one aripiprazole patient died during the stabilization phase of the study from heroin intoxication (10-47-85) and one died from a suspected pulmonary embolism 61 days after discontinuation of aripiprazole (10-134-341). The narratives of these deaths are copied from the submission in the appendix of this document.

There were no deaths in study CN138037.

7.1.2 Other Serious Adverse Events

Study 13810:
Stabilization: Seventy-three patients (13.2%) experienced non-fatal serious adverse events (SAE). Psychiatric related events were the most common of these: "reaction manic" (3.4%), "depression" (2.9%), "reaction manic depressive" (2.5%), and "thought suicidal" (2.5%). There was one case each of pancreatitis (history of chronic pancreatitis with recurrent attacks), suicide attempt, chest pain, seizure, and spontaneous abortion.
Double blind maintenance: 13.3% of the placebo group and 7.8% of the aripiprazole group experienced at least one serious adverse event.

- Placebo: reaction manic (6.0% placebo, 5.2% aripiprazole) and depression (3.6% placebo, 0% aripiprazole) were the most common serious adverse events. One suicide attempt occurred in the placebo group.
- Aripiprazole: reaction manic (5.2%), paralysis (1.3 % aripiprazole, 0% placebo) and alcohol intolerance (1.3% aripiprazole, 0% placebo) were the most common serious adverse events in the drug treated group. No suicide attempt occurred in the aripiprazole group in this phase. The paralysis is indicated to have occurred after an automobile accident.

Extension: 29.6% of the placebo group and 7.7% of the aripiprazole group experienced a SAE.

- Placebo group: reaction manic was the #1 event at 18.5% and anxiety and depression each accounted for 3.7%.
- Aripiprazole group: reaction manic (5.1%) and reaction manic depressive (2.6%) were the only reported SAEs.

Study 138037: Three patients experienced SAEs in study CN138037. These were events of manic-depressive reaction. One patient became pregnant (day 56) and was discontinued.

7.1.3 Dropouts and Other Significant Adverse Events

7.1.3.1 Overall profile of dropouts

(The table of disposition for trial CN138010 is in section 6.1.4 Efficacy findings of this review.)

7.1.3.2 Adverse events associated with dropouts

Study CN138010:
During the **stabilization phase**, 23.7% of the patients, all on aripiprazole treatment, experienced an adverse event that led to discontinuation. The most common of these events were psychiatric in nature (depression 6.3%, reaction manic 2.9%, thought suicidal 2.4%, reaction manic depressive 2.0%, agitation 1.8%, and akathisia, anxiety, insomnia, and somnolence each at 1.6%, nervousness 0.7%). GI related events (nausea 1.1%, dyspepsia 0.4%, vomiting 0.4%), blurred vision (0.7%), EPS (0.5%) and laboratory related (CPK increased 0.4%) events also contributed to drop-outs in this phase.

During the **double blind maintenance** phase of the study, 19.3% of the placebo-treated patients and 10.4% of the aripiprazole treated patients experienced a treatment emergent adverse event (TEAE) that led to discontinuation.

- Placebo: Depression (7.2 % versus 1.3 %), reaction manic (6.0% versus 3.9%), insomnia (4.8% versus 0%), and agitation (2.4% versus 1.3%) were the most common TEAEs leading to discontinuation in the placebo group. One patient (placebo) discontinued secondary to a suicide attempt.
- Aripiprazole: Reaction manic (3.9%) was the most common TEAE in the aripiprazole group with agitation, akathisia (1.3% versus 0% placebo), depression, hypertension, and alcohol intolerance each contributing 1.3% incidence of discontinuation.
- There were no discontinuations secondary to laboratory abnormalities in this phase.

During the **extension phase**, 25.9% of the placebo group and 10.3% of the aripiprazole group experienced a TEAE leading to discontinuation:.

- placebo group: (reaction manic (14.8%, depression 7.4%, and anxiety 3.7%)
- aripiprazole group (5.1% reaction manic, 2.6% each for akathisia and reaction manic depressive).
- There were no discontinuations secondary to laboratory abnormalities in this phase.

Study CN138037: Four of the 24 patients discontinued secondary to an adverse event. The most common adverse event leading to discontinuation was reaction manic depressive in two patients.

7.1.4 Other Search Strategies

The JMP file of adverse events for study CN138010 was screened for terms coded (AETXT) hepatitis, liver failure, kidney failure, renal failure, rhabdomyolysis, and jaundice. No instances were seen. The laboratory JMP files for Study CN138010 were screened for CPK elevations. The highest value noted was about 11,500. This patient is discussed elsewhere in the safety section of this review.

7.1.5 Common Adverse Events

The common adverse event profile for the bipolar population was characterized in the acute mania supplement.

Data from study CN138010 are somewhat difficult to interpret. The stabilization phase has no control group, however offers some idea perhaps of introduction to the drug. The maintenance phase is confounded by withdrawal in the placebo group, differential exposure time to placebo and aripiprazole, and selection bias. The extension phase group is small and likely reflects groups who suffer from selection bias.

7.1.5.1 Eliciting adverse events data in the development program

Patients were asked about adverse events by the investigator at weekly assessments beginning at the initiation of study treatment and recorded on the CRF. (A copy of the schedule of events, as duplicated from the submission, is included in the safety appendix of this document.)

7.1.5.2 Appropriateness of adverse event categorization and preferred terms

- Preferred terms (PTERM) from the JMP dataset of adverse events for trial CN138010 were compared to the adverse event text terms (AETXT). There were no obvious systematic coding issues. There were some coding terms that should be coded to more appropriate terms. For example, patient 10-64-288 PTERM is akathisia when the text term was tardive dyskinesia and patient 10-34-94. Abnormal Behavior is the PTERM-check this patient # while the text term describes intermittent, non, purposeful lip smacking.
- Preferred terms scanned (cursory review) in other QUADR.xpt files submitted with the ISS to supplement 005 (limited to events since June, 2002) did reveal several occasions where coding might alter how serious the event would be perceived or would create the need to look multiple places in an incidence table to "cover" an occurrence. Additionally, there were missing preferred terms for which text terms were present. The safety appendix of this document contains further information. It is recommended that we ask the sponsor to link these missing terms to an appropriate preferred term and optimize the translation of text from the CRF to preferred terms.

7.1.5.3 Incidence of common adverse events

The adverse event profile of the drug was derived from the acute studies.

7.1.5.6 Additional analyses and explorations

(Copies of the study procedures/schedules are in the safety appendix of this document.)

EPS: The sponsor performed scales directed at assessing treatment emergent Parkinsonism (Simpson-Angus Scale, SAS), dyskinesia (Abnormal Involuntary Movement Scale,AIMS), and akathisia (Barnes Akathisia Global Assessment Score) during stabilization, maintenance, and extension phases. SAS scores range from 10-50, AIMS total scores range from 0-28, the Barnes Akathisia scale scores range from 0 to 5 (5 =severe). With all scales, a negative change indicates improvement.

Although the data generated are confounded by withdrawal from drug in the placebo group, by differential exposure times to drug in the groups, and possibly by concomitant medication use, the difference in the mean changes from baseline measures between the groups is small anyway and likely not clinically meaningful.

EPS-related adverse events: The data for these events are difficult to interpret secondary to the study design and are not discussed in detail in this part of the review secondary to this. A table is duplicated from the submission in the appendix of this document. One person in the aripiprazole

group (1/77) discontinued secondary to akathisia in the maintenance phase; no placebo patient did (0/83).

Suicidality: The interpretation of the change in MADRS data is also questionable as there is a differential exposure time between the placebo and aripiprazole groups in the maintenance phase. MADRS scores were acquired at every study visit during the maintenance study. The MADRS item 10 score was used to assess treatment emergent suicidality. The sponsor notes that among patients with a baseline score of 0-2, the incidence of scores of 5-6 at any time during the study was 0% for (0/76) for the aripiprazole patients and 1.25% (1/80) for the placebo patients. This analysis appears to have been performed on the maintenance phase sample.

- Treatment emergent adverse events

1) Two events classified as "suicide attempt" occurred in the stabilization phase and 17 events (3.07%) of "thought suicidal" occurred.
2) One event of suicide attempt occurred in maintenance phase; this was a placebo patient. Two events of "thought suicidal" occurred in placebo patients and one in aripiprazole patients (1.30%) during the maintenance phase.
3) One event of "thought suicidal" occurred in the placebo group during the extension phase.

- Serious adverse events related to suicidality:
  1) During stabilization, there was one suicide attempt and 12 "thought suicidal" (2.5%).
  2) During maintenance, there was 1 suicide attempt in a placebo patient and one incidence of "thought suicidal".
- Discontinuation secondary to suicide related events:
  1) One discontinuation secondary to suicide attempt and 13 secondary to "thought suicidal" occurred in the stabilization phase.
  2) One discontinuation secondary to suicide attempt occurred in a placebo patient during the maintenance phase (0 in the aripiprazole group) and two discontinuations secondary to "thought suicidal" occurred in the placebo group with 0 in the aripiprazole group.

Glucose Metabolism:
The sponsor notes that no adverse events related to glucose metabolism were reported in either treatment group during the longer-term maintenance study (ISS-005).

Overdose: The sponsor searched the database for all Phase 2/3 studies to identify overdose of aripiprazole defined as >60 mg. Since the safety update of 2002, 11 patients were identified. None of these were from the bipolar mania trials.

Abuse, tolerance, and physical dependence have not been specifically studied in humans using aripiprazole.

Seizure: One patient experienced an event captured as seizure-related in the stabilization phase. No patients experienced a seizure related event in the combined maintenance and extension phases.

Pregnancy- Four patients became pregnant during Study CN138010; two in the stabilization

phase (10-509, 141-266), one in the extension phase (100-116), and one before treatment (91-181). One patient became pregnant during study CN138037. This patient and patient 141-266 terminated or induced abortion. The baby of patient 100-116 experienced shoulder dislocation and jaundice which reportedly resolved.

Patient 10-509, a 44 year old female, experienced spontaneous abortion on day 151. At the time of discontinuation secondary to severe depression on day 112, her pregnancy testing was negative. It appears she was treated with risperidone and buproprion with resolution of the event noted on day 138 when she returned for a follow-up visit. At this visit, she expressed that she suspected she was pregnant. This was confirmed on day 141. The patient could not recall the date of her last menstrual period. However, the narrative notes the gestational age when pregnancy was "diagnosed" was estimated at 1-4 weeks. Concomitant medication use of oral contraceptive appears to be before the pregnancy (days 38-92), if this gestational age is correct. This patient had no previous history of spontaneous abortion or stillbirth.

7.1.6 Less Common Adverse Events

In study CN138010, one case of Raynaud's and a case of retrograde ejaculation were noted in the maintenance population. Three patients are coded as tardive dyskinesia in the JMP set. Two were placebo patients who experienced dyskinesia in the maintenance phase and one was an aripiprazole patient in the extension phase.

The appendix with this listing may be found in the submission to supplement 002 dated May 26, 2004 (p.109-150). Labeling review as per Dr. Andreason.

7.1.7 Laboratory Findings

Two patients dropped out of therapy in the stabilization phase secondary to elevated CPK values; patients 99-229 (AEs include leg cramps and myalgia) and 108-348 (SAE of mania; foot wounds, patient also had increased LDH). Narratives indicate that both patients experienced elevations of CK/CK-MB while on 30 mg daily of aripiprazole. Neither patient was coded as having NMS or rhabdomyolysis. An additional patient was (146-459) was originally noted to be dropped out secondary to elevated prolactin, however it was found this was incorrect as this elevation occurred after discontinuation for other adverse events.

7.1.7.1 Overview of laboratory testing in the development program

In the stabilization phase, screening EKG, urine and blood samples for routine hematology and chemistry laboratories, urinalysis, pregnancy, and drugs of abuse were not required for patients from the acute mania trials. Prolactin levels were measured during stabilization at baseline if the patient had not entered from an acute study.

During the maintenance phase and extension phases, samples were collected for routine laboratory analysis at scheduled intervals (a schedule of events may be found in the appendix of

this document). Prolactin levels were measured at randomization and throughout the double-blind phases.

Quality Control issues: It appears from the amended appendices of protocol deviations, there were a fairly large number of patients who did not get lithium/valproic acid levels or had other missing labs at stabilization. About 25 patients had some clinical lab tests missing on or before the maintenance phase start date and five women did not receive pregnancy testing on or before the maintenance phase dose start date. Dr. Khin's inspection report noted that some laboratory measures were missing on many patients at site 93.

7.1.7.2 Selection of studies and analyses for drug-control comparisons of laboratory values

Study CN138010 was the only study submitted to support the indication for maintenance use in bipolar patients.

7.1.7.3 Standard analyses and explorations of laboratory data

7.1.7.3.1 Analyses focused on measures of central tendency

Median change from baseline data were provided for the maintenance phase and combined for the maintenance and extension phases. These data were not reviewed as interpretation is problematic for the reasons discussed previously

7.1.3.3.3 Marked outliers and dropouts for laboratory abnormalities

(PCS criteria are reproduced as from the submission and may be found in the appendix of this document.)

CK:

1) Stabilization: 8/452 patients experienced potentially clinically significant PCS values in the stabilization period. The JMP files of adverse events does not contain events of renal failure, rhabdomyolysis, or NMS.
   - Patient 13-543 had an increase of up to about 11500 from 67. This patient experienced myalgias during treatment.
   - Several patients had normal baselines and went to >1000 (10-8-331, 10-109-115)
   - 2 patients discontinued secondary to increased CPK (99-229 and 108-348)
2) Maintanance: 3/ 73 placebo patients and 5/74 aripiprazole patients experienced PCS values.
3) Extension: One placebo patient (1/26) and one aripiprazole patient (1/38) experienced a PCS value.
4) The sponsor notes there were no discontinuations secondary to abnormal laboratory values in either the maintenance or extension phases.

LFTs: The ISS notes that there were four patients with treatment emergent abnormal hepatic laboratory measures. One patient had both an elevated AST and ALT during stabilization, two patients had a single transaminase elevation, and a fourth had an elevated bilirubin at the visit

prior to the last visit. It was noted that none of these patients had simultaneous elevations of transaminase and bilirubin.

Prolactin:

Stabilization: Although the CRS noted one discontinuation secondary to elevated prolactin, upon further information, this was not correct as it appears the elevated prolactin level was after discontinuation.

7.1.8 Vital Signs

Quantitative interpretation of these data are limited secondary to similar issues as discussed in section 7.1.5 Common Adverse Events.

7.1.8.1 Overview of vital signs testing in the development program

Supine and standing systolic and diastolic blood pressures and radial artery pulses were measured at scheduled visits after the patient was supine for five minutes. Upon standing, the measurement was taken after two minutes. Vital signs scheduled on simultaneous visits as blood draws were measure before the blood draw. (A schedule of events is included in the safety appendix of this document.)

Quality Control: Additionally, Dr. Khin' review noted stabilization phase EKGs on 17 patients at site 118 were noted as missing in the original study report, however, upon inspection, the sponsor had identified all except one. The updated appendices noting violations have adjusted for these EKGs (8-27-04 submission).

7.1.8.2 Selection of studies and analyses for overall drug-control comparisons

Study CN138010 is the only maintenance study with placebo-controlled data

7.1.8.3 Standard analyses and explorations of vital signs data

Review of the analyses of central tendency was not performed. Outlier data review was limited. The interpretation of these data given this study design is problematic. This review has focused on serious adverse events and drop-outs secondary to vital sign related events.

7.1.8.3.3 Marked outliers and dropouts for vital sign abnormalities

During stabilization three patients treated with aripiprazole dropped out of therapy secondary to a vital sign related adverse event; hypotension (10-359), syncope (71-59-amphetamine use also had reportedly seizure) and tachycardia (64-605). One aripiprazole patient discontinued therapy during the maintenance phase for hypertension (73-574).

7.1.8.4 Additional analyses and explorations

QTc: QT data were described in the review for the indication of acute mania. Due to the difficulty in interpretation of these data secondary to the study design, limited discussion of QT is included in this review other than section 7.1.9.3.2 below.

Orthostatic Blood Pressure Measures: An orthostatic blood pressure measure was defined as any systolic blood pressure decrease ≥ 30 mm Hg supine to standing. Interpretation of this type of information is also limited. In the combined extension and maintenance phases, the difference between the treatment groups in patients experiencing orthostatic blood pressure measures was about 7%.

The overall incidences of orthostatic-related adverse events during the maintenance phase were about the same in the two groups. Syncope was seen in one placebo patient and no aripiprazole patient.

Body weight: Body weight and waist circumference were recorded at scheduled visits (see appendix for copy of schedules) on the same scale for a given patient and in a standardized manner. Waist circumference was measured at the level of the umbilicus.

Most of the data from CN138010 relative to this will not be discussed in detail as the interpretation is limited. The mean change from baseline in patient weight using endpoint LOCF in the stabilization safety sample was only computed on patients who discontinued because weight was not measured in patients who continued. The mean change in 308 patients was 0.16 kg.

7.1.9 Electrocardiograms (ECGs)

7.1.9.1 Overview of ECG testing in the development program, including brief review of preclinical results

Twelve-lead EKGs were acquired during study CN138010. The schedule of procedures may be found in the safety appendix of this document.

7.1.9.3 Standard analyses and explorations of ECG data

7.1.9.3.1 Analyses focused on measures of central tendency

These data were not reviewed due to problems with interpretation as previously discussed throughout this review.

7.1.9.3.2 Analyses focused on outliers or shifts from normal to abnormal

The following information is provided, however, the interpretation of this is limited. During the stabilization phase, two patients had potentially clinically significant QT interval changes(≥450

msec and 10% increase from baseline) when corrected with Bazett's formula and none did when corrected with the DNDP formula (QTcN=QT/RR). The sponsor notes that no aripiprazole treated patient had PCS QTc changes during the Maintenance or Extension phases and no patient discontinued secondary to a QTc abnormality.

7.1.9.3.3 Marked outliers and dropouts for ECG abnormalities

One patient discontinued for an EKG related event (109-67) that was an SAE. This patient was hospitalizaed secondary to bigeminy on day 56 of the study and medication was discontinued. The event was reported as resolved on day 58. The CRF notes there was no previous cardiac history. The patient was noted to have palpitations on a visit 19 days earlier.

There was symmetrical T-wave inversion in one/75 aripiprazole patient (36-399) in the maintenance phase and 0/77 placebo patient. There was no adverse event listed in the line listing that would correlate with this EKG abnormality.

7.1.12 Special Safety Studies

EPS: The Simpson-Angus scale (SAS), the Abnormal Involuntary Movement Scale (AIMS), the Barnes Akathisia Rating Scale, and EPS-related events were assessed (see section 7.1.5.6 above). (Tables of the data are reproduced in the appendix of this document.)

7.1.13 Withdrawal Phenomena and/or Abuse Potential

No additional studies of abuse were submitted with this submission.

7.1.14 Human Reproduction and Pregnancy Data

Pregnancy issues in longer term studies of mania were discussed above in section 7.1.5.6. A complete listing of patients who have become pregnant while on aripiprazole treatment is included in the appendix of this document.

7.1.15 Assessment of Effect on Growth

Not assessed in this supplement.

7.1.16 Overdose Experience

No overdoses of study medication occurred in study CN138010.

7.1.17 Postmarketing Experience

The postmarketing information with supplement 002 encompasses or supercedes that of this submission.

There were four cases of DVT/PE events in the reporting period from July 17, 2003 – January 16, 2004. One was of DVT, two were PE, and one case with PE and DVT. Although there are confounders in these cases such as history of smoking, obesity, or the use of other medications such as clozapine, venlafaxine, or risperidone, causality cannot be totally ruled out. One case of pulmonary embolism occurred in a 27 year old bipolar patient treated for one month. [ ] b(5)
[ ]

The sponsor also notes that cumulative reports of hypersensitivity, hepatobiliary events, increased creatine phosphokinase/rhabdomyolysis, and syncope "suggest a possible causal relationship". [ ] b(5)

- Hepatobiliary events included 17 cases of AST/ALT elevation-10 were classified as serious. Values for the peak ALT were reported in 14 cases, the highest was 684. Values for the peak ALT was reported in 8 cases and was 374. The sponsor notes that in the majority of the 17 cases although confounded, a causal role for aripiprazole could not be totally ruled out and that in six cases there was positive dechallenge. A hepatitis reported as drug induced occurred in a 34 year old patient who was taking concomitant medications and had taken a months worth of multivitamins at once one week prior to the event. However, the synopsis notes her AST/ALT were normal two months before and returned to normal after discontinuation of aripiprazole.
- One case synopsis of increased bilirubin (12379806) did not provide adequate information for reasonable interpretation.

7.2 Adequacy of Patient Exposure and Safety Assessments

7.2.1 Description of Primary Clinical Data Sources (Populations Exposed and Extent of Exposure) Used to Evaluate Safety

7.2.1.1 Study type and design/patient enumeration

567 patients enrolled in study CN138010. Of these 206, completed stabilization and 196 were randomized. Due to unblinding, 35 were discontinued from the randomized phase leaving 161 patients. Of these, 78 were aripiprazole and 83 were placebo treated. 28 placebo and 39 aripiprazole patients completed the maintenance phase of which 27 placebo and all aripiprazole patients entered the continued double-blind extension phase. Five placebo patients and seven aripiprazole patients completed the extension phase (about 37% of each group discontinued this phase because the sponsor terminated the study).

7.2.1.2 Demographics

(Tables are reproduced from the submission in the safety appendix of this document.)

The mean age at randomization was similar between groups. About 60% of the aripiprazole patients in the maintenance phase were female and about 70% of the placebo patients. Most patients in both groups were White (67% aripiprazole, 62% placebo). Hispanic/Latino comprised the next largest group representing 26% of the aripiprazole patients and 20% of the placebo patients.

With respect to psychiatric history, 78% (458/633) of the enrolled patients were not rapid cycling patients. Most patients enrolled were coded as current episode manic (61%). In the randomized population, about 17% were rapid cycling patients and most were coded as current episode manic (78% placebo and 62% aripiprazole).

Dr. He's review notes that the study was not powered for subgroup analysis with respect to gender, race, and age. Relapse rates were lower for male and female aripiprazole treated patients than for placebo treated patients.

Appears This Way On Original

7.2.1.3 Extent of exposure (dose/duration) Tables are duplicated from the submission.

Table 9.1A **Number of Patients Receiving Study Medication and Mean and Range of Daily Dose During the Stabilization Phase, Stabilization Safety Sample**

	Aripiprazole		
**Day (Intervals)**	**N**	**Mean (mg)**	**Range of Daily Dose[a]**
1-7	541[c]	27.77	12.86 - 30.00
8-14	487	26.78	7.50 - 47.14
15-21	438	26.01	8.57 - 30.00
22-28	397	25.65	0.00 - 30.00
29-35	361	25.12	12.86 - 30.00
36-42	339	24.84	4.29 - 34.29
43-49	302	24.66	8.57 - 30.00
50-56	268	24.73	6.43 - 30.00
57-63	227	24.69	10.71 - 30.00
64-70	204	24.38	11.25 - 34.29
71-77	181	24.11	10.71 - 30.00
78-84	167	24.42	10.71 - 30.00
85-91	145	24.36	10.71 - 30.00
92-98	123	24.74	12.86 - 30.00
99-105	101	24.86	15.00 - 30.00
106-112	84	25.48	10.71 - 30.00
113-119	74	25.34	12.86 - 30.00
120-126	65	25.05	15.00 - 30.00
> 126	43	23.90	7.50 - 30.00
Endpoint	541	25.25	8.57 - 34.29

Protocol CN138010
Source: Appendix 9.1

[a] Range of daily doses take into account patients who deviated from the dose specified in the protocol or who were noncompliant.

[b] Twelve patients in the Stabilization Safety Sample were excluded from the table because of incomplete dosing dates.

Appears This Way
On Original

Table 9.1B-1 **Number of Patients Receiving Study Medication and Mean and Range of Daily Dose, Patients Who Completed Stabilization and Remained Eligible for the Maintenance Phase**

	Aripiprazole		
**Day (Interval)**	**N**	**Mean (mg)**	**Range of Daily Dose[a]**
**Patients Who Remained Eligible for the Maintenance Phase**			
1-7	196	28.28	15.00 - 30.00
8-14	196	27.43	12.86 - 32.14
15-21	195	26.26	8.57 - 30.00
22-28	195	26.02	0.00 - 30.00
29-35	196	25.10	12.86 - 30.00
36-42	196	24.56	4.29 - 30.00
43-49	179	24.11	8.57 - 30.00
50-56	163	24.33	6.43 - 30.00
57-63	135	24.02	10.71 - 30.00
64-70	125	23.88	12.86 - 30.00
71-77	116	23.46	10.71 - 30.00
78-84	110	23.67	10.71 - 30.00
85-91	97	23.77	12.86 - 30.00
92-98	84	23.95	12.86 - 30.00
99-105	71	24.18	15.00 - 30.00
106-112	60	24.75	15.00 - 30.00
113-119	51	24.54	15.00 - 30.00
120-126	47	24.12	15.00 - 30.00
> 126	30	23.50	15.00 - 30.00
Endpoint	196	24.39	12.86 - 30.00
Number (%)of patients with endpoint dose of 15 mg			73 (37%)
Number (%)of patients with endpoint dose of 30 mg			123 (63%)
**Patients Who Were Randomized into the Maintenance Safety Sample**			
Endpoint	161	24.38	12.86 -30.00
Number (%)of patients with endpoint dose of 15 mg			60 (37%)
Number (%)of patients with endpoint dose of 30 mg			101 (63%)

Protocol CN138010
Source: Appendix 9.1
[a] Range of daily doses take into account patients who deviated from the dose specified in the protocol or who were noncompliant.

7.2.2.2 Postmarketing experience

The postmarketing information with supplement 002 encompasses or supercedes that of this submission.

There were four cases of DVT/PE events in the reporting period from July 17, 2003 – January 16, 2004. One was of DVT, two were PE, and one case with PE and DVT. Although there are confounders in these cases such as history of smoking, obesity, or the use of other medications such as clozapine, venlafaxine, or risperidone, causality cannot be totally ruled out. One case of pulmonary embolism occurred in a 27 year old bipolar patient treated for one month. [ ] [ ] b(5)

The sponsor also notes that cumulative reports of hypersensitivity, hepatobiliary events, increased creatine phosphokinase/rhabdomyolysis, and syncope "suggest a possible causal relationship". [ ] b(5)

7.2.2.3 Literature

The sponsor notes that an update to previously submitted literature searches was conducted with the search time frame from March 14, 2003 to August 31, 2003 by Atsuko Nakano (licensed pharmacist) and Julia Jui-mei Chuang (Information Scientist, Master's Organic Chemistry).. 159 articles were reviewed by Dr. Joy Parris, M.D. or Dr. Margaretta Nyilas, M.D. and certification of no adverse findings were provided.

Also, see section 8.6 for further literature.

7.2.4 Adequacy of Special Animal and/or In Vitro Testing

No new pre-clinical data were submitted in support of this supplement.

7.2.5 Adequacy of Routine Clinical Testing

From a clinical point of view, the planned monitoring appears adequate. From an interpretative point of view, the design of the study makes it difficult to interpret the vital sign, clinical laboratory, and EKG data quantitatively.

From a quality control point of view, it appears that violations of missing labs occurred, many focused on lithium and valproate levels. However, manipulations of the JMP files indicate missing baseline values on some patients for clinical labs. At one of the sites in Mexico, 17 EKGs were uncovered at an audit by the sponsor. These EKGs were reported as missing in the stabilization phase in the Clinical Study Report and therefore probably are not captured in summary tables. Serious EKG related events should have been captured as serious adverse events elsewhere, so this may not be a practical safety issue but it possibly does raise the issue of how well or uniform the sites were monitored.

7.2.6 Adequacy of Metabolic, Clearance, and Interaction Workup

7.2.7 Adequacy of Evaluation for Potential Adverse Events for Any New Drug and Particularly for Drugs in the Class Represented by the New Drug; Recommendations for Further Study

7.2.8 Assessment of Quality and Completeness of Data

From an interpretative point of view, the design of study CN138010 makes it difficult to interpret the vital sign, clinical laboratory, and EKG data quantitatively.

From a quality control point of view, it appears that violations of missing labs occurred, many involved lithium and valproate levels (as per the appendices and Dr. Khin's report from DSI). However, manipulations of the JMP files indicate missing baseline values on some patients for

clinical labs. At one of the sites in Mexico, 17 EKGs were uncovered at an audit by the sponsor. These EKGs were reported as missing in the stabilization phase in the Clinical Study Report and therefore probably are not captured in summary tables. Serious EKG or laboratory- related events should have been captured as serious adverse events elsewhere, so this may not be a practical safety issue but it does raise issues as to how sites were monitored and how quality control was conducted during compilation of the study report.

The coding of text from the CRFs to the COSTART term for study CN138010 appears generally to be adequate and is discussed in section 7.1.5.2. Recommendations to the sponsor regarding the non-bipolar indications and coding are included in sections 7.1.5.2 and 9.5.

- With regard to non-bipolar indications, the incidence tables of adverse events by indications for all aripiprazole treated patients cannot be meaningfully interpreted and were not reviewed as tables are not exposure and placebo adjusted.
- Line listings of patients who died, experienced a non-fatal serious adverse event, or discontinued secondary to an adverse event in studies blinded or newly reported since September, 2002 were submitted with supplement 002, the 120 day safety update, the response to the approvable, and supplement 005. Line listings of the deaths generally do not include the cause of death.

7.2.9 Additional Submissions, Including Safety Update

There were no safety updates for supplement 005. Data from other submissions such as line listings as discussed in section 7.1 were utilized as were safety updates from submissions to S002.

7.3 Summary of Selected Drug-Related Adverse Events, Important Limitations of Data, and Conclusions

Drug related adverse events were captured in the acute studies. Limitations of the data are discussed above.

7.4 General Methodology

7.4.1 Pooling Data Across Studies to Estimate and Compare Incidence

Study data from CN138010 was submitted and categorized by the phase of the study.

7.4.1.1 Pooled data vs. individual study data

Appendix 6.4.3.1 in the ISS is a list of the incidences of treatment-emergent adverse events that occurred in all aripiprazole treated patients in all phase 2 and 3 studies by indication. This table is separated by patient type however there is no placebo group for comparison or exposure adjustment.

7.4.2 Explorations for Predictive Factors

7.4.2.1 Explorations for dose dependency for adverse findings

This was a flexible dose study.

7.4.2.2 Explorations for time dependency for adverse findings

This study design cannot support this type of interpretation.

7.4.2.3 Explorations for drug-demographic interactions

Not explored in this study

7.4.2.4 Explorations for drug-disease interactions

Not explored in this study

7.4.2.5 Explorations for drug-drug interactions

b(4)

8 ADDITIONAL CLINICAL ISSUES

8.1 Dosing Regimen and Administration

Study CN138010 was not a fixed dose study. At the end of stabilization, the mean daily dose was about 25mg with a range from approximately 9-34 mg. Of patients who completed the stabilization phase and remained eligible for the maintenance phase (161), the mean dose was about the same at 24 mg and the range was about 13 to 30 mg daily. 63% of these patients were on 30 mg daily at endpoint and 37% were on 15mg daily.

8.2 Drug-Drug Interactions

There is no new information provided with the supplement.

8.6 Literature

Possible Induction of Mania or Hypomania by Atypical Antipsychotics: An Updated Review of Reported Cases. J. Clinical Psychiatry 2004; 65: 1537-1545

This is an update to a previous article published in 2000. MEDLINE was searched (1999-2003) using terms for various drugs such as the atypical antipsychotics, including aripiprazole, with the terms hypomania and mania. 34 new cases of mood switch were noted. The authors conclude that more than half of the new cases are "highly suggestive" of a causal link. The majority of these cases did not have a diagnosis of bipolar disorder and many were schizophrenia or schizophreniform disorder. The authors note that no reported cases were with clozapine. Although none were with aripiprazole, the authors note that the lack of reporting with aripiprazole and sertindole may reflect worldwide drug use.

9 OVERALL ASSESSMENT

9.1 Conclusions

9.2 Recommendation on Regulatory Action

I recommend the Division consider an approvable action on this supplement. While the p value is significant when comparing aripiprazole to placebo for time from randomization to relapse in the maintenance phase, it is unclear to me that this reflects efficacy of the drug. While the larger pool of data favors aripiprazole, removal of one site in Mexico (site 93) causes the study to lose significance. This site appears to have a different relapse rate than the conglomerate U.S. sites. DSI inspection at this site revealed protocol violations, however, overall the data were deemed acceptable. As this is the only study for maintenance and given that a large number of U.S. sites were involved but alone are not powered to show significance and for other reasons listed within this review, I recommend we ask for further exploration of the data in this study with attention to the Mexican sites, more so to site 93.

9.3 Recommendation on Postmarketing Actions

9.3.1 Risk Management Activity

b(5)

A change to the label regarding the risk of cerebrovascular adverse events in elderly patients with dementia has been added as a WARNING. This is based on a recent review by Dr. Marc

Stone in DNDP. Additionally, the OVERDOSAGE/Human Experience subsection currently is under review in SLR007.

9.3.2 Required Phase 4 Commitments

Required Phase 4 commitments were delineated in the action letter for this supplement. As commitments were made regarding adult studies to address short and longer term efficacy as add-on therapy in bipolar patients and pharmacology-toxicology studies needed to support pediatric trials with the action on supplement 002 (acute mania), no additional studies are required at this time. However, the sponsor was asked to state a date of submission of the clinical study reports for the recently completed drug interaction studies.

9.5 Comments to Applicant

It is recommended that the sponsor re-examine the JMP databases for ISSQADR1, ISSQADR2, and ISSQADR3 for text terms that are missing preferred terms. Additionally, there appear to be terms such as "Abnormal Lab" or "Abnormal ECG" when more optimal terms for these instances would have noted that these were clinically relevant or at high levels (for example, patient 4-83-271 and patient 4-68-582). It is recommended that preferred terms be modified to more accurately reflect the potential seriousness of the text terms.

Appears This Way
On Original

10 APPENDICES

CRF AUDITS of Study CN138010:
Site-patient: 1-250
6-46
34-494
10-512
64-441
69-519
92-145
93-184
93-504
99-40
111-132
132-355
141-401
146-437

Appears This Way
On Original

10.1 Review of Individual Study Reports

INVESTIGATORS FOR STUDY CN138010:

005	Lawrence W. Adler, M.D.
006	Asaf Aleem, M.D.
071	Dan Anderson, M.D.
140	Michael Banov, M.D.
007	Mohammed Bari, M.D.
008	Bijan Bastani, M.D.
127	Louise Beckett, M.D.
035	Gregory Bishop, M.D.
146	Jeffrey Borenstein, M.D.
009	Charles Lee Bowden, M.D.
108	Ronald Brenner, M.D.
010	David Brown, M.D.
077	E. Sherwood Brown, M.D., Ph.D.
012	Jose M. Canive, M.D.
001	Brendan T. Carroll, M.D.
013	Franca Centorrino, M.D.
015	Christopher Chung, M.D.
071	Evagelos Coskinas, M.D., Ph.D.
098	Evagelos Coskinas, M.D., Ph.D.

016	Andrew Cutler, M.D.
073	David Daniel, M.D.
017	Larry Davis, M.D.
018	Lori L. Davis, M.D.
019	Kathleen Degen, M.D.
074	G. Michael Dempsey, M.D.
098	Himasiri DeSilva, M.D.
145	Robert B. DeTrinis, M.D.
099	Bradley C. Diner, M.D.
020	John Downs, M.D.
041	Eduardo Dunayevich, M.D.
021	Rif S. El-Mallakh, M.D.
144	Louis F. Fabre, M.D., Ph.D.
106	Richard Farrer, M.D.
080	Ronald Fieve, M.D.
111	Roxana B. Galeno, M.D.
141	Natalie Gershman, M.D.
122	Lawrence D. Ginsberg, M.D.
081	John W. Goethe, M.D.
023	Joseph F. Goldberg, M.D.
055	Clifford Goldman, M.D.
073	Ramanath Gopalan, M.D.
026	Laszlo Gyulai, M.D.
027	Mahlon S. Hale, M.D.
028	Mark B. Hamner, M.D.
082	Barbara Harris, Ph.D.
110	Harold Harsch, M.D.
029	Radwan Haykal, M.D.
118	Miguel Angel Herrera Estrella, M.D.
100	Scott Hoopes, M.D.
109	Robert Horne, M.D.
055	Robert C. Jamieson, M.D.
033	Philip G. Janicak, M.D.
034	Anita Kablinger, M.D.
089	Eduardo Kalina, M.D.
131	Jasbir S. Kang, M.D.
041	Paul E. Keck, Jr., M.D.
036	Terence Ketter, M.D.
147	Arif Khan, M.D.
130	Mary Ann Knesevich, M.D.
047	Michael T. Lambert, M.D.
143	Mark Lerman, M.D.
128	Michael T. Levy, M.D.

002	H. Edward Logue, M.D.
132	Adam F. Lowy, M.D.
136	M. Azfar Malik, M.D.
083	Paul Markovitz, M.D., Ph.D.
040	Howard Keith Mason, M.D.
043	Denis Mee-Lee, M.D.
088	Jose Luis Mendiola, M.D.
142	Ricky S. Mofsen, D.O.
091	Alberto Monchablon, M.D.
072	David Morin, M.D.
046	Richard Pearlman, M.D.
047	Frederick Petty, M.D., Ph.D.
134	Sohail Punjwani, M.D.
003	Joachim Raese, M.D.
049	Rakesh Ranjan, M.D.
052	Neil M. Richtand, M.D., Ph.D.
053	Samuel Craig Risch, M.D.
075	Barry R. Rittberg, M.D.
054	Judy S. Rivenbark, M.D.
093	Ignacio Rosales, M.D.
126	Leon Rubenfaer, M.D.
135	David Sack, M.D.
057	Frederick Schaerf, M.D., Ph.D.
103	Rahim Shafa, M.D.
058	Anantha Shekhar, M.D., Ph.D.
092	Kenneth Sokolski, M.D.
085	Vicky E. Spratlin, M.D.
084	Patricia Suppes, M.D., Ph.D.
060	Norman Sussman, M.D.
061	Alan Swann, M.D.
062	Kathleen Toups, M.D.
063	Mark H. Townsend, M.D.
094	J. Charlene Tracy, D.O.
064	Tram K. Tran-Johnson, Pharm.D.
065	Adam Travis, M.D., Ph.D.
066	Harold D. Udelman, M.D.
094	Marilyn J. Vache, M.D.
068	Richard Wang, M.D.
069	Richard H. Weisler, M.D.
070	Andrew Winokur, M.D., Ph.D.
071	Craig Wronski, D.O.
072	Carlos A. Zarate, Jr. M.D.
094	Jill Zweig, D.O.

INCLUSION AND EXCLUSION CRITERIA:

INCLUSION:

Inclusion Criteria into the Stabilization phase:

1) Patients with DSM-IV diagnosis of Bipolar I Disorder who had experienced at least 2 previous manic or mixed episodes including the most recent episode.
2) Patients who experienced a recent manic or mixed episode requiring hospitalization and treatment with medications that began no more than 3 months before entry into the stabilization phase
3) Patients who were eligible for an acute mania study but declined.
4) Men and women 18 and older. Women of child bearing potential must have had a negative pregnancy test within 72 hours of starting study medication, were to use an acceptable form of contraception, and could not be pregnant or lactating.
5) Patients were able to give informed consent or had an acceptable legal representative ot give consent prior to initiation of any protocol procedures
6) Patients were able to comprehend and satisfactorily comply with the protocol.

Inclusion Maintenance:

7) Patients who continued to meet criteria 1, 3, and 5.
8) Patients who were in the stabilization phase for at least six weeks.
9) Stable as per YMRS $\leq$ 10 and MADRS $\leq$ 13 during 4 consecutive visits.

Inclusion Extension:

10) Patients who completed 26 weeks of maintenance.
11) Women as in criteria 4 above.
12) Patients deemed suitable for participation in a long-term trial, for example, regarding compliance.

EXCLUSION:

Exclusion Criteria, Stabilization Phase:

1) Patients with a clinical picture or history consistent with a DSM-IV Axis I diagnosis of delirium, dementia, amnesia, other cognitive disorder, schizophrenia, or schizoaffective disorder.
2) Patients with psychotic symptoms better accounted for by another general medical condition or direct physiological effects of a substance.
3) Patients unresponsive to clozapine.
4) Patients likely to need prohibited concomitant therapy during the trial
5) Patients who met DSM-IV for any significant psychoactive or substance use disorder within the past 3 months including benzodiazepines but excluding caffeine or nicotine.
6) A positive cocaine screen (could be reassessed at randomization). Patients with screens positive for stimulants or drugs of abuse were to be discussed with the BMS monitor.
7) Known allergy or hypersensitivity to aripiprazole or the quinolinones.
8) Patients with significant suicide or homicide risk based on history or mental status exam.
9) Patients with unstable thyroid pathology or treatment within the past 3 months.
10) Patients with a history of NMS.
11) Patients with a history or evidence of a medical condition that would create undue risk to them or interfere with safety or efficacy assessments.
12) Patients with clinically significant abnormal laboratory tests, vital signs, or EKG findings.
13) Women who did not meet the inclusion criteria.
14) Recent treatment with a long-acting antipsychotic in which the last dose was < one full cycle + one week (haloperidol decanoate treatment within the past 5 weeks or fluphenazine decanoate within the past 3 weeks).
15) Use of psychotropics, other than benzodiazepines, within 1 day of baseline.
16) Fluoxetine within the past 4 weeks.
17) Patients in other investigational trials (except aripiprazole) within the past month.
18) ECT treatment within the past 2 months.

19) Patients with a history of seizure disorder.
Exclusion Maintenance:
20) Patients who were not compliant in the stabilization phase.
21) Patients in the stabilization phase > 18 weeks.
22) Patients + for lithium, divalproex acid, or drugs of abuse.
23) Patients with significant protocol violations in the stabilization phase.
Exclusion Extension:
24) Patients not compliant in the maintenance phase.
25) Patients with + drug screen.
26) Patients with significant protocol violations in the maintenance phase.
27) Women who planned to become pregnant while in the study.
28) Patients who likely would need prohibited medication therapy.

CENSORING:

Figure S.10.1 Assessment of the Impact of Censoring on the Primary Analysis

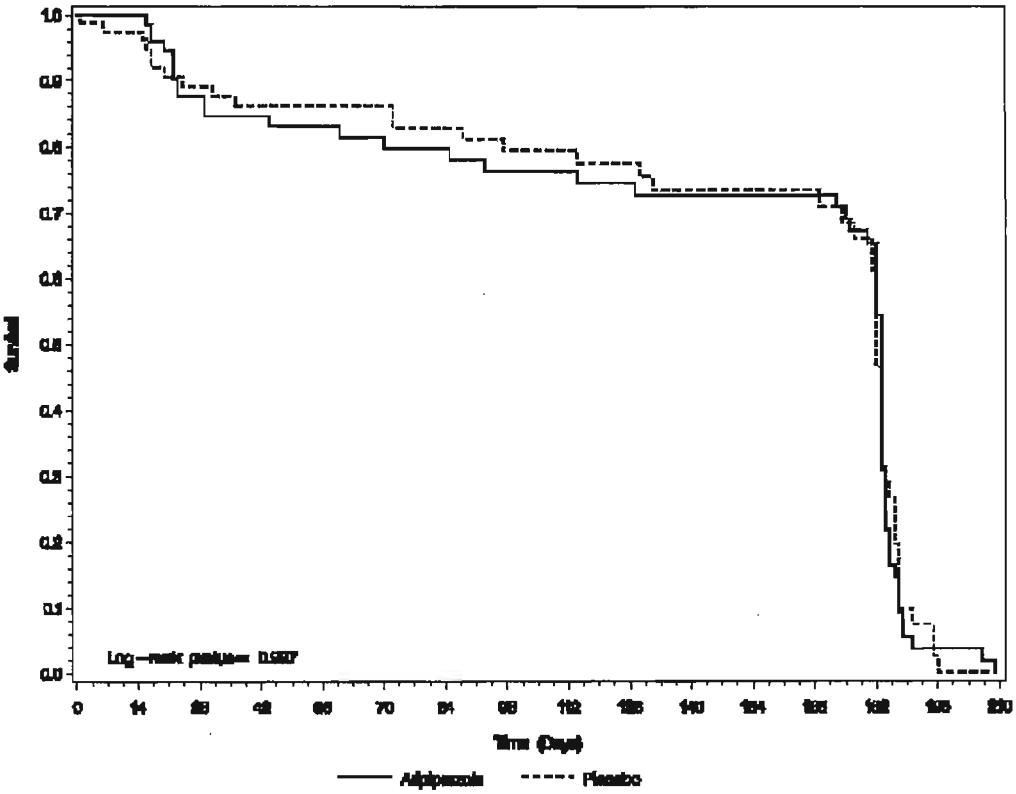

Protocol CN138010
Source: Appendix 10.1A

Number of Patients Stabilized by Study Day Interval

Table 2: Number of Patients Stabilized by Study Day Interval, Maintenance Safety Sample

	Placebo N=83	Aripiprazole N=77
**Time in Stabilization**	**Number (%) of Patients**	**Number (%) of Patients**
0 - 14 days	0	0
15 - 28 days	0	0
29 - 42 days	4 (4.8)	9 (11.7)
43 - 56 days	19 (22.9)	16 (20.8)
57 - 70 days	6 (7.2)	8 (10.4)
71 - 84 days	11 (13.3)	6 (7.8)
85 - 98 days	12 (14.5)	9 (11.7)
99 - 112 days	10 (12.1)	7 (9.1)
113 - 126 days	9 (10.8)	8 (10.4)
127 - 140 days	9 (10.8)	9 (11.7)
141 - 154 days	2 (2.4)	0
155 - 168 days	1 (1.2)	1 (1.3)
169 - 182 days	0	1 (1.3)
183 - 196 days	0	0
197 - 210 days	0	1 (1.3)
211 - 224 days	0	1 (1.3)
225 - 238 days	0	0
239 - 252 days	0	0
253 - 266 days	0	1 (1.3)

Protocol CN138010

Time in Stabilization, IND versus Non-IND sites

Table 3: **Number of Patients Stabilized by Study Day Interval and Site IND Status, Maintenance Safety Sample**

	IND Sites				Non-IND Sites[b]			
	Placebo N = 64		Aripiprazole N = 59		Placebo N = 19		Aripiprazole N = 18	
**Time in Stabilization**	N	(%)	N	(%)	N	(%)	N	(%)
0 - 14 days	0		0		0		0	
15 - 28 days	0		0		0		0	
29 - 42 days	4	(6.3)	8	(13.6)	0		1	(5.6)
43 - 56 days	19	(29.7)	14	(23.7)	0		2	(11.1)
57 - 70 days	6	(9.4)	7	(11.9)	0		1	(5.6)
71 - 84 days	7	(10.9)	4	(6.8)	4	(21.1)	2	(11.1)
85 - 98 days	7	(10.9)	6	(10.2)	5	(26.3)	3	(16.7)
99 - 112 days	8	(12.5)	7	(11.9)	2	(10.5)	0	
113 - 126 days	6	(9.4)	5	(8.5)	3	(15.8)	3	(16.7)
127 - 140 days	6	(9.4)	6	(10.2)	3	(15.8)	3	(16.7)
141 - 154 days	1	(1.6)	0		1	(5.3)	0	
155 - 168 days	0		1	(1.7)	1	(5.3)	0	
169 - 182 days	0		1	(1.7)	0		0	
183 - 196 days	0		0		0		0	
197 - 210 days	0		0		0		1	(5.6)
211 - 224 days	0		0		0		1	(5.6)
225 - 238 days	0		0		0		0	
239 - 252 days	0		0		0		0	
253 - 266 days	0		0		0		1	(5.6)

Protocol CN138010

The following 5 sites were Non-IND sites: 089 (Argentina), 091 (Argentina), 093 (Mexico), 111 (Argentina), and 118 (Mexico).

SAFETY APPENDIX

Appears This Way
On Original

Table S.12.2B: Narratives for Patients Who Died

Patient 138010-47-85

Patient 138010-47-85, a 52-year-old male, with a history of colon polyps, enlarged prostate, deep vein thrombosis following surgical repair of fractures of left femur, clotting disorder, fractured skull 3 times, fractured right wrist, left hand, left wrist, and lower jaw, fine finger tremors bilaterally, allergy to morphine, alcoholic (no alcohol since 1992), tobacco use, elevated white blood cells-etiology unknown, positive purified protein derivative, tuberculosis, hypertension, post traumatic stress disorder, and kidney dialysis in 1994 due to lithium toxicity. The patient completed three weeks of 30 mg of aripiprazole on study CN138007 (138007-47-103) and entered the open-label stabilization phase of the Maintenance study (CN138010) on Day 1, at a dose of 30 mg per day. The patient then entered the double-blind maintenance phase and received 30mg of aripiprazole daily on Days 158 - 259. On Day 259 the patient discontinued study medication due to lack of efficacy (relapse) and the patient discontinued from the study on Day 260. The total time of exposure to aripiprazole between the two studies was 177 days. Adverse events ongoing at the time of study discontinuation were increased saliva, nightmares (abnormal dream), protein in urine (albuminuria) and intermittent post traumatic stress disorder nightmares (neurosis). Concomitant medications taken within 14 days prior to discontinuation were docusate, finasteride, and Habitrol™. On Day 320, 60 days after discontinuing from the study and 61 days after discontinuing from study medication, the patient died of a suspected pulmonary embolism. No clinically significant vital sign, ECG, or laboratory abnormalities were reported. Potentially clinically significant weight gain (*) is as follows:

Study Day	Weight (kg)
-1	120
157	135 (*)
164	135 (*)
199	135 (*)

Patient 138010-134-341

Patient 138010-134-341, a 39-year-old male, presented with a history of right foot injury, headaches, panic disorder, and past drug and alcohol use (none last year). The patient completed 3 weeks of aripiprazole on study CN138007 (138007-108-470) and entered the open-label stabilization phase of the Maintenance study (CN138010). The patient received 30 mg of aripiprazole on Days 1 - 20. The total time of exposure to aripiprazole between the two studies was 41 days. On Day 20, the patient was taken to the emergency room for low blood pressure and poor respiratory rate, and was discharged from the hospital later that day. On Day 21 (Day 42 of combined study participation), the patient returned to the emergency room unresponsive, in severe distress, and later died at 18:30. The patient had a blood pressure value of 159/103 mmHg and a respiratory rate of 30 breaths/minute. Results from the autopsy report indicated that the patient died due to a very severe heroin intoxication (drug dependence). The investigator designated this event as unrelated to study therapy. No adverse events were reported during the study. No concomitant medications were reported to study staff during the study. Toxicology report showed blood levels of alprazolam. No other clinically significant vital signs, ECG, or laboratory abnormalities were reported during the study.

Appears This Way
On Original

PREFERRED TERMS:

COSTART Audit ISSQADR1, ISSQADR2, ISSQADR3 events since June 30, 2002

PATIENT #	Preferred Term (PTERM)	Text from CRF (AETXT)
4-68-582 (ISSAQADR1)	Abnormal ECG	Clinically relevant ECG
4-83-271	Abnormal Lab	Critical value CK
97301-188-1	Overdose	Suicide Attempt (Overdosing of study medication)
4-113-290	nodule	Liver nodules
5-42-250- event was an SAE	Abnormal thinking	Acute altered mental status
5-43-228	Myasthenia	Right sided weakness
87-536-1276	Myasthenia	Weak in stomach
87-149-1459 (ISSQADR3)	Failure Heart	Cardiomyopathy due to Lescol
32-65-129	Inflammation	"Cervical Spondyiosis"
87-289-502	Ketosis	Diabetic Ketoacidosis
87-416-657	Ketosis	Diabetic Ketoacidosis
100-142-119	(blank)	Lack of Efficacy
3-20-750	(blank)	Pregnancy
100-213-177	(blank)	relapse
100-14-446	(blank)	Subarachnoid bleed
100-149-84	(blank)	Text discusses suicidal ideations
6-20-160 (ISSQADR2)	(blank)	Death

Appears This Way
On Original

STUDY PROCEDURES:

Table 5.8A: Screening and Stabilization Phases, Schedule of Study Procedures and Observations

	Screening Phase (up to 28 days)		Stabilization Phase (6 - 18 weeks)				
Procedure	Screening[a]	Baseline Visit[a]	Week 2	Week 4	Week 6	Weeks 8 - 18[b]	Early Term Visit[c]
Informed Consent	X						
Demographic Data	X						
Entrance Criteria	X						
Medical History	X[d]						
Psychiatric History	X[d]						
Previous Medications	X[d]						
SCID or MINI	X[d]						
**Efficacy**							
Y-MRS		X	X	X	X	X	X
CGI-BP		X	X	X	X	X	X
PANSS		X					X
MADRS		X	X	X	X	X	X
**Safety**							
Physical Exam	X[d]						X
Vital signs	X[d]	X[e]	X	X	X	X	X[e]
Weight		X					X
ECG 12-Lead	X[d]						X
Clinical Laboratory Tests	X[d]						X
Prolactin Level		X[d]					X
Pregnancy Test (WOCBP)	X[d]	X[f]		X		X[f]	X
Drug Screen[g]	X[d]			X		X[g]	X
SAS		X	X	X		X	X
AIMS		X					X
Barnes Akathisia		X	X	X		X	X
Adverse Events			X	X	X	X	X

Appears This Way
On Original

STUDY PROCEDURES:

Table 5.8A: Screening and Stabilization Phases, Schedule of Study Procedures and Observations

	Screening Phase (up to 28 days)		Stabilization Phase (6 - 18 weeks)				
Procedure	Screening[a]	Baseline Visit[a]	Week 2	Week 4	Week 6	Weeks 8 - 18[b]	Early Term Visit[c]
**Other**							
Telephone Contact[h]		X--X					
Concomitant Therapy Form	X[d]	X	X	X	X	X	X
Study Therapy Form			X	X	X	X	X
Drug Accountability Form		X	X	X	X	X	X
Baseline Visit Form		X					
End-of-Stabilization Phase Form[i]							X

Protocol CN138010
Source: Appendices 5.1A, 5.1B, 5.1C

a Screening and baseline visits may coincide with each other and the end-of-study visit from acute study for patients entering the study after participating in an acute study.

b After Week 6 patients had visits every 2 weeks only until their Bipolar Disorder was stable as defined by the protocol. SAS & Barnes Akathisia were not required at Weeks 10 & 14. Patients then entered the Maintenance Phase.

c Completed if patient discontinued prior to entering Maintenance Phase.

d Not required for patients entering the study after participating in an acute study of aripiprazole. If more than 3 days past screening window, BMS monitor was consulted.

e Waist circumference was also measured.

f Serum or urine pregnancy test performed within 72 hours of the first administration of study medication, every 4 weeks thereafter, and at the early termination visit.

g Drug screen for drugs of abuse must have been negative. Drug screen done at Week 4, 8, 12, 16, and at the early termination visit.

h Patients were contacted by telephone at the end of every odd numbered week (eg, Week 1, Week 3) to monitor compliance with prescribed medication and assure the patients well-being.

i Completed at last visit in Stabilization Phase.

Appears This Way
On Original

STUDY PROCEDURES:

Table 5.8B: Randomization and Maintenance Phase, Schedule of Study Procedures and Observations

		Study Week															
**Procedure**	**Day 1[a]**	**1**	**2**	**3**	**4**	**6**	**8**	**10**	**12**	**14**	**16**	**18**	**20**	**22**	**24**	**26[b]**	**Early Term Visit[c]**
Entrance Criteria	X																
**Efficacy**																	
Y-MRS		X	X	X	X	X	X	X	X	X	X	X	X	X	X	X	X
CGI-BP		X	X	X	X	X	X	X	X	X	X	X	X	X	X	X	X
PANSS	X						X				X					X	X
MADRS		X	X	X	X	X	X	X	X	X	X	X	X	X	X	X	X
**Safety**																	
Physical Exam																X	X
Vital signs	X[d]	X	X	X	X	X	X	X	X	X	X	X	X	X	X	X	X
Weight	X															X	X
ECG 12-Lead	X						X				X					X	X
Clinical Laboratory Tests	X						X				X					X	X
Lithium and Divalproex Levels[e]	X																
Prolactin Level	X															X	X
Pregnancy Test (WOCBP)[f]	X				X		X		X		X		X		X	X	X

Table 5.8B: Randomization and Maintenance Phase, Schedule of Study Procedures and Observations

		Study Week															
**Procedure**	**Day 1[a]**	**1**	**2**	**3**	**4**	**6**	**8**	**10**	**12**	**14**	**16**	**18**	**20**	**22**	**24**	**26[b]**	**Early Term Visit[c]**
Drug Screen[g]	X	X	X	X	X	X	X	X	X	X	X	X	X	X	X	X	X
SAS			X		X		X		X		X		X			X	X
AIMS	X						X				X					X	X
Barnes Akathisia			X		X		X		X		X		X			X	X
AEs	X	X	X	X	X	X	X	X	X	X	X	X	X	X	X	X	X
**Other**																	
Telephone Contact[h]						X------	------	------	------	------	------	------	------	------	------	------X	
Concomitant Therapy Form	X		X		X	X	X	X	X	X	X	X	X	X	X	X	X
Study Therapy Form			X		X	X	X	X	X	X	X	X	X	X	X	X	X
Drug Accountability Form	X		X		X	X	X	X	X	X	X	X	X	X	X	X	X
Relapse Form																X	X
End-of-Study Form																X	X

Protocol CN138010

Source: Appendices 5.1A, 5.1B, 5.1C

[a] Performed at randomization/Day 1, in addition to procedures performed at the last visit in the Stabilization Phase

[illegible] Clinical Study Report

[b] End-of-study or at the time of earlier discontinuation.

[c] Completed if patient discontinued prior to entering the Extension Phase

[d] Waist circumference was also measured

[e] Serum levels for lithium and divalproex must have been negative

[f] Urine pregnancy test performed every 4 weeks

[g] Drug screen for drugs of abuse must have been negative. Drug screen done at every study visit starting at Week 1 until Week 26/Early Termination Visit

[h] Patients were contacted by telephone at the end of every odd numbered week after Week 4 to assess compliance with prescribed medication and ensure the patients well-being.

Table 5.8C: Extension Phase, Schedule of Study Procedures and Observations

	Study Week													
**Procedure**	**28**	**32**	**36**	**40**	**44**	**48**	**52**	**60**	**68**	**76**	**84**	**92**	**100**	**Early Term Visit**
Adverse Events	X	X	X	X	X	X	X	X	X	X	X	X	X	X
**Other**														
Telephone Contact[d]		X—	—	—	—	—	—	—	—	—	—X			
Concomitant Therapy Form	X	X	X	X	X	X	X	X	X	X	X	X	X	X
Study Therapy Form	X	X	X	X	X	X	X	X	X	X	X	X	X	X
Drug Accountability Form	X	X	X	X	X	X	X	X	X	X	X	X	X	X
End-of-Study Form[e]													X	X
Relapse Form														X

Protocol CN138010
Source: Appendices 5.1A, 5.1B, 5.1C

[a] CGI-BP Improvement was not assessed.

[b] Waist circumference was also measured.

[c] Drug screen for cocaine must have been negative. Drug Screen performed every 4 weeks (up to Week 52) and then every 8 weeks thereafter.

[d] Patients were contacted by telephone at every week when they don't have a study visit to assess compliance with prescribed medication and assure the patients well-being.

[e] Or at the time the patient was discontinued if prior to end-of-study visit/when 45 patients had relapsed in the Maintenance Phase.

Table 5.8C: Extension Phase, Schedule of Study Procedures and Observations

	Study Week													
**Procedure**	**28**	**32**	**36**	**40**	**44**	**48**	**52**	**60**	**68**	**76**	**84**	**92**	**100**	**Early Term Visit**
**Efficacy**														
Y-MRS	X	X	X	X	X	X	X	X	X	X	X	X	X	X
CGI-BP (Severity)[a]	X	X	X	X	X	X	X	X	X	X	X	X	X	X
PANSS				X			X		X				X	X
MADRS	X	X	X	X	X	X	X	X	X	X	X	X	X	X
**Safety**														
Physical Exam													X	X
Vital signs	X	X	X	X	X	X	X			X			X[b]	X
Weight													X	X
ECG 12-Lead						X	X			X			X	X
Clinical Laboratory Tests				X			X			X			X	X
Prolactin Level				X			X			X			X	X
Pregnancy Test (WOCBP)	X	X	X	X	X	X	X	X	X	X	X	X	X	X
Drug Screen[c]	X	X	X	X	X	X	X	X	X	X	X	X	X	X
SAS				X			X		X		X		X	X
AIMS				X			X		X		X		X	X
Barnes Akathisia				X			X		X		X		X	X

Table 6.3.3.1A: Incidence of Treatment-Emergent Adverse Events that Occurred in ≥ 5% in Any Treatment Group During the Maintenance Phase: Longer-Term Maintenance of Stability Study in Bipolar Mania (CN138010), Maintenance Safety Sample

Body System/ Primary Term[a]	Number (%) Patients Placebo N = 83	Aripiprazole N = 77
**Any Adverse Event**	58 (69.9)	57 (74.0)
**Body as a Whole**		
Asthenia	7 (8.4)	6 (7.8)
Headache	14 (16.9)	6 (7.8)
Pain extremity	1 (1.2)	4 (5.2)
Pain back	5 (6.0)	3 (3.9)
**Cardiovascular System**		
Hypertension	3 (3.6)	4 (5.2)
**Digestive System**		
Nausea	4 (4.8)	7 (9.1)
**Nervous System**		
Anxiety	12 (14.5)	13 (16.9)
Insomnia	16 (19.3)	12 (15.6)
Depression	12 (14.5)	9 (11.7)
Nervousness	5 (6.0)	8 (10.4)
Tremor	1 (1.2)	7 (9.1)
Agitation	9 (10.8)	6 (7.8)
Akathisia	1 (1.2)	5 (6.5)
Reaction manic	11 (13.3)	5 (6.5)
Somnolence	6 (7.2)	4 (5.2)
Depersonalization	8 (9.6)	3 (3.9)
**Respiratory System**		
URI	8 (9.6)	7 (9.1)
**Urogenital System**		
Vaginitis[b]	0	3 (6.4)

Appears This Way
On Original

Table 6.3.3.1A: Incidence of Treatment-Emergent Adverse Events that Occurred in ≥ 5% in Any Treatment Group During the Maintenance Phase: Longer-Term Maintenance of Stability Study in Bipolar Mania (CN138010), Maintenance Safety Sample

	Number (%) Patients	
**Body System/** Primary Term[a]	**Placebo N = 83**	**Aripiprazole N = 77**
Infection urinary tract	3 (3.6)	4 (5.2)

[a] Modified COSTART term.

[b] Incidence adjusted for gender (women): placebo N = 60; aripiprazole N = 47.

Appears This Way
On Original

POTENTIALLY SIGNIFICANT VALUES

Table S.6.3.3.4: Criteria for Identifying Potentially Clinically Significant Laboratory Values

Laboratory Tests	Criteria[a]
Chemistry[b]	
AST (SGOT)	≥ 3 x upper limit of normal (ULN)
ALT (SGPT)	≥ 3 x ULN
Alkaline phosphatase	≥ 3 x ULN
LDH	≥ 3 x ULN
BUN	≥ 30 mg/dL
Creatinine	≥ 2.0 mg/dL
Uric acid	
Men	≥ 10.5 mg/dL
Women	≥ 8.5 mg/dL
Bilirubin (total)	≥ 2.0 mg/dL
Total cholesterol	≥ 200 mg/dL
Hematology	
Hematocrit	
Men	≤ 37 % and decrease of ≥ 3 percentage points from Baseline
Women	≤ 32 % and decrease of ≥ 3 percentage points from Baseline
Hemoglobin	
Men	≤ 11.5 g/dL
Women	≤ 9.5 g/dL
White blood count	≤ 2800/ mm^3 or ≥ 16,000/ mm^3
Eosinophils	≥ 10%
Neutrophils	≤ 15%
Platelet count	≤ 75,000/ mm^3 or ≥ 700,000/ mm^3
Urinalysis	
Protein	Increase of ≥ 2 units
Glucose	Increase of ≥ 2 units
Casts	Increase of ≥ 2 units

Protocol CN138010
Source: Appendix 5.1A

[a] As defined in "Supplementary Suggestions for Preparing an Integrated Summary of Safety Information in an Original NDA Submission and for Organizing Information in Periodic Safety Updates," FDA Division of Neuropharmacological Drug Products draft (2/27/87).

[b] In addition to the above-listed laboratory tests, the following tests were evaluated for the aripiprazole program: prolactin > upper limit of normal; CPK ≥ 3x upper limit of normal.

Table S.6.3.3.5: Criteria for Identifying Potentially Clinically Significant Vital Sign Measurements

Vital Sign	Criterion Value[a]	Change from Baseline[a]
Heart rate	120 bpm	≥ 15 bpm increase
	50 bpm	≥ 15 bpm decrease
Systolic blood pressure	180 mmHg	≥ 20 mmHg increase
	90 mmHg	≥ 20 mmHg decrease
Diastolic blood pressure	105 mmHg	≥ 15 mmHg increase
	50 mmHg	≥ 15 mmHg decrease

Protocol CN138010

Source: Appendix 5.1A

[a] As defined in "Supplementary Suggestions for Preparing an Integrated Summary of Safety Information in an Original NDA Submission and for Organizing Information in Periodic Safety Updates," FDA Division of Neuropharmacological Drug Products draft (2/27/87).

Appears This Way
On Original

Table S.6.3.3.6: Criteria for Identifying Potentially Clinically Significant ECG Measurements

Variable	Criterion Value[a]	Change Relative to Baseline[a]
**Rate**		
Tachycardia	≥ 120 bpm	increase of ≥ 15 bpm
Bradycardia	≤ 50 bpm	decrease of ≥ 15 bpm
**Rhythm**		
Sinus tachycardia[b]	≥ 120 bpm	increase of ≥ 15 bpm
Sinus bradycardia[c]	≤ 50 bpm	decrease of ≥ 15 bpm
Supraventricular premature beat	≥ 2 per 10 seconds	any increase
	all	not present → present
Ventricular premature beat	≥ 1 per 10 seconds	any increase
Supraventricular tachycardia	all	not present → present
Ventricular tachycardia	all	not present → present
Atrial fibrillation	all	not present → present
Atrial flutter	all	not present → present
**Conduction**		
1° atrioventricular block	PR ≥ 0.20 second	increase of ≥ 0.05 second
2° atrioventricular block	all	not present → present
3° atrioventricular block	all	not present → present
Left bundle-branch block	all	not present → present
Right bundle-branch block	all	not present → present
Pre-excitation syndrome	all	not present → present
Other intraventricular conduction block[d]	QRS ≥ 0.12 second	increase of ≥ 0.02 second
**Infarction**		
Acute or subacute	all	not present → present
Old	all	not present → present ≥ 12 weeks post study entry
**ST/T Morphological**		
Myocardial ischemia	all	not present → present
Symmetrical T-wave inversions	all	not present → present
Increase in QT	$QT_c \geq 450$	≥ 10% increase

Protocol CN138010
Source: Appendix 5.1A

[a] Criteria developed for a previous BMS filing based upon discussions with the FDA Division of Neuropharmacological Drug Products.

[b] No current diagnosis of supraventricular tachycardia, ventricular tachycardia, atrial fibrillation, atrial flutter, or other rhythm abnormality.

[c] No current diagnosis of atrial fibrillation, atrial flutter, or other rhythm abnormality.

[d] No current diagnosis of left bundle branch block or right bundle branch block.

Appears This Way
On Original

Aripiprazole
BMS-337039/OPC-14597

Integrated Summary of Safety
Bipolar Maintenance

Table 9.1.4.3: Mean Change from Baseline to Endpoint and Highest Score, SAS and AIMS Total Score, and Barnes Akathisia Global Clinical Assessment, LOCF Data Set During the Maintenance Phase: Longer-Term Maintenance of Stability Study in Bipolar Mania (CN138010), Maintenance Safety Sample

EPS Scale	Placebo	Aripiprazole
**SAS Total Score**[a]	N = 81	N = 76
Mean at last visit Stabilization Phase (SE)	10.79 (0.18)	10.59 (0.18)
Change from last visit Stabilization Phase at Endpoint (SE)	-0.14 (0.17)	0.19 (0.18)
Change from last visit Stabilization Phase at Highest Score (SE)	0.44 (0.18)	0.91 (0.19)
**AIMS Total Score**[b]	N = 79	N = 73
Mean at randomization (SE)	0.25 (0.10)	0.14 (0.10)
Change from randomization at Endpoint (SE)	0.06 (0.10)	0.09 (0.10)
Change from randomization at Highest Score (SE)	0.11 (0.15)	0.40 (0.15)
**Barnes Akathisia**[c]	N = 81	N = 76
Mean at last visit Stabilization Phase (SE)	0.28 (0.07)	0.37 (0.08)
Change from last visit Stabilization Phase at Endpoint (SE)	-0.14 (0.06)	-0.05 (0.07)
Change from last visit Stabilization Phase at Highest Score (SE)	0.13 (0.08)	0.29 (0.09)

Note: For each analysis, patients in the Safety Sample were required to have both an assessment at the last visit in the Stabilization Phase or prior to randomization and an assessment during the Maintenance Phase for the rating scale that was analyzed.

[a] SAS Total Score ranges from 10 to 50. A negative change score indicates improvement.

[b] AIMS Total Score ranges from 0 to 28. A negative change score indicates improvement.

[c] Global Clinical Assessment Score ranges from 0 (absent) to 5 (severe akathisia). A negative change score indicates improvement.

Appears This Way
On Original

Table 8.3B Demographic Characteristics, Randomized Sample

Variable		Placebo N = 83	Aripiprazole N = 78	Total N = 161
Age (years)	Mean	40.3	39.0	39.6
	Median	40.0	38.5	40.0
	Range	18.0-62.0	18.0-80.0	18.0-80.0
	SE	1.2	1.5	0.9
Gender N (%)	Male	23 (28)	30 (38)	53 (33)
	Female	60 (72)	48 (62)	108 (67)
Race N (%)	White	56 (67)	48 (62)	104 (65)
	Hispanic/Latino	17 (20)	20 (26)	37 (23)
	Black	5 (6)	5 (6)	10 (6)
	Asian/Pacific Islander	4 (5)	2 (3)	6 (4)
	American/Alaskan Native	0	1 (1)	1 (1)
	Other	1 (1)	2 (3)	3 (2)

Protocol CN138010
Source: Appendix 8.3

Table 8.4A Psychiatric History of Bipolar Disorder, Enrolled Sample

Variable		Aripiprazole N = 633
Age current episode began	Mean	39.8
(derived from of onset of episode)	Median	40.0
	Minimum-Maximum	18.0-80.0
	SE	0.5
	Missing	64
Rapid Cycling	Yes	126 (22)
	No	458 (78)
	Missing	49
Current Episode is N (%)	Manic	353 (61)
	Mixed	228 (39)
	Missing	52

Protocol CN138010
Source: Appendices 8.4A, 8.4B

Table S.9.4.4: By-Patient Listing of Pregnancy: All Aripiprazole Data Set, Safety Sample

Patient Number[a]	Patient Number[b]	Age	Outcome	New Report[c]
97201-18-7	97203-18-7	36	Ectopic pregnancy	No
98204-359-3	98222-359-3	21	Elective abortion	No
98215-366-10	98222-366-10	21	Normal delivery of healthy infant	No
98304-409-56	97303-200-56	31	Missed abortion[d]	No
98304-525-73	97303-140-73	32	Elective Abortion	Yes[e]
98304-527-54	97303-527-54	40	Elective abortion	No
98304-527-62	97303-527-62	24	Elective abortion	No
98304-528-50	--	36	Normal delivery of healthy infant	No
138003-11-749	--	37	Elective Abortion	Yes[e]
138003-20-750	138112-32-1	35	Normal delivery of healthy infant	Yes
138003-39-712	--	31	Elective Abortion	Yes[e]
138003-45-259	--	30	Spontaneous abortion	Yes[e]
138003-45-707	--	32	Normal delivery of health twins	Yes[e]
138003-49-700	--	30	Elective abortion	Yes[e]
138009-42-120	138010-100-116	26	Live infant with medical problem but no birth defect[f]	Yes[g]
138010-10-509	--	44	Spontaneous abortion	Yes[g]
138010-141-266	--	18	Elective abortion	No
138032-54-30	--	23	Normal delivery of healthy infant	Yes[e]
138074-16-98	138037-19-23	27	Elective abortion	Yes[g]
138087-31-413	--	19	Elective abortion	Yes[e]
138087-100-129	--	30	Normal delivery of healthy infant	Yes[e]

[a] Patients are identified by their unique identification number based on their original study.

[b] Patient identification in follow-on study.

[c] New report since the November 30, 2002/February 7, 2003 data cut-off for the Bipolar Mania ISE/ISS.

[d] In the ISS, outcome was reported as an elective abortion. Per Safety Update #4 (December 21, 2001) that was received after the database lock date for the 120-Day Safety Update (February 2002), the embryo was not viable at the time of the abortion. Therefore, the new Primary Term for this event is "Missed Abortion".

[e] Narratives were previously submitted.

[f] The baby was born with a dislocated shoulder and jaundice that resolved within 24 hours.

[g] Narratives for these patients are presented in the Clinical Study Report for that study.

This is a representation of an electronic record that was signed electronically and this page is the manifestation of the electronic signature.

/s/

Teresa Podruchny
12/7/04 03:57:03 PM
MEDICAL OFFICER

Paul Andreason
12/17/04 04:50:34 PM
MEDICAL OFFICER
I agree that supplement-05 is approvable, but site 93 results should be varified as explored before considering final approval. I note Dr Podruchny's labeling recommendations for the post marketing adverse event section.

Review and Evaluation of Clinical Data
NDA #21-436

**Sponsor:**	BMS/Otsuka
**Drug:**	Abilify (aripiprazole)
**Proposed Indication:**	Maintenance of Efficacy in Bipolar I Disorder
**Material Submitted:**	Response to 11-30-04 Approvable Letter
**Correspondence Date:**	January 3, 2005
**Date Received:**	January 3, 2005

I. Background

Aripiprazole is an atypical antipsychotic agent that is currently approved for the acute and maintenance treatment of schizophrenia and for the treatment of acute manic or mixed episodes associated with bipolar disorder. This supplement (S-005) was submitted on 1-28-04 to gain approval for maintenance treatment in bipolar disorder. Supporting clinical data was derived from a single trial (CN138-010) which consisted of an open-label aripiprazole stabilization phase followed by randomization of stabilized patient to continued aripiprazole treatment or placebo for a 26 week maintenance period; patients who remained stable during the 26 week period could receive open-label aripiprazole during an extension phase.

The clinical data was reviewed by Dr. Teresa Podruchny and an approvable letter for S-005 was sent on 11-30-04. The sponsor was informed that the following issues would need to be addressed before this supplement could be approved:

1) The efficacy results of study CN138-010 appear to be driven by a single center (93) in Mexico City. The treatment effect at this center differs markedly from the effect observed at U.S. centers. This finding raises questions about the reliability of the efficacy results. The sponsor was requested to address this concern.

2) A number of patients dropped out of study CN138-010 during the maintenance treatment phase but were not counted as relapses. The available information on these patients

was insufficient to assure us that these patients did not prematurely discontinue study participation due to worsening bipolar disorder. The sponsor was requested to carefully reexamine the data for these patients and provide us with a sufficiently detailed description of the reasons for dropout so that we could independently verify that the patients were not relapsing at the time of discontinuation. If any patients were reclassified as relapses, we asked the sponsor to reanalyze the data after reclassification.

3) The draft labeling attached to the approvable letter included a new section under WARNINGS that described the risk of cerebrovascular adverse events (CVAE's) in elderly patients with dementia who received aripiprazole. Additionally, we asked that all previous revisions to labeling, as reflected in the most recently approved package insert, be included. We requested that their revised labeling proposal clearly indicate all changes.

4) We requested written agreement regarding all pertinent Phase 4 commitments:

a) adult clinical studies to address the clinical efficacy and safety of aripiprazole as add-on therapy in bipolar disorder.
b) juvenile animal toxicity studies to support pediatric studies of aripiprazole in bipolar disorder.
c) drug interaction studies with lithium and valproate.

The first two commitments were agreed to as part of the approval action of S-002 (for the treatment of acute manic and mixed episodes associated with bipolar disorder).

This submission contains adequate responses to all issues above. These responses are summarized below.

II. Review of Approvable Letter Response

A. Center 93

Center 93 had the second largest number of randomized patients (N=13 or 8% of the total sample) and had results strongly favoring drug over placebo (0% (0/6) of aripiprazole patients relapsed compared to 71% (5/7) of placebo patients). Based on crude relapse rates, the results at this center were markedly superior to those in the overall study (28% for aripiprazole and 51% for

placebo). In the primary analysis of time to relapse using Kaplan-Meier methodology, the log-rank p-values based on all sites was 0.020; with center 93 excluded, the p-value becomes non-significant: 0.104.

BMS asserts that this loss of significance with the exclusion of center 93 is, in part, due to loss of statistical power. The sponsor also points out that there was no a priori rationale to exclude the data from center 93 in the efficacy analysis.

The FDA Division of Scientific Investigations (DSI) inspected center 93 and found the data from this site to be acceptable despite a number of deficiencies (see 11-3-04 review by Dr. Ni Khin). Additionally, BMS states that their internal monitoring reports were consistent with the DSI conclusions. Due to the small numbers of patients enrolled at most of the sites in study CN138-010, statistical testing for an interaction between treatment and center was not feasible.[1]

The sponsor also cites the crude relapse rates for all relapse, for depressive relapse, and for manic relapse as well as the mean changes in the Y-MRS from randomization to endpoint with all sites versus with center 93 excluded to demonstrate minimal impact from excluding center 93. Since the primary and key secondary variables were time to event and not crude relapse rates or mean change from baseline, this data has less relevance.

Reviewer's Comments

In the absence of evidence of a study characteristic which would have produced a biased result at center 93, this site should be retained in the primary analysis. This is consistent with the intent-to-treat principle. Also, in my opinion, to embark on a policy of discarding a site solely on the basis of an outlying result invites a number of difficult questions, such as how to quantitatively define an outlying result which merits exclusion of the site and whether we would allow the exclusion of a very poorly performing site which would render an otherwise negative study positive. Furthermore, it is not entirely clear that quantitatively defining an outlying result that merits exclusion is a reasonable approach because it begs the question of whether a deviant finding was part of the

---

[1] Of the 50 centers in this trail, only 14 treated 2 or more patients per treatment group and only 9 centers treated 6 or more patients.

natural variation in the responsivity of the illness to the drug versus the result of a peculiarity in the patient sample or study conduct. This question would be extremely difficult to answer in many cases. Admittedly, my approach does not preclude the possibility of a source of bias that has been undetected. The inspection done by DSI and monitoring performed by BMS may not have been sufficiently sensitive to reveal the source of the remarkable result at center 93. Nonetheless, without a clear reason to do so, I do not feel that the exclusion of data from center 93 is warranted.

B. Potential Misclassification of Relapse

A total of 38 patients in study CN138-010 dropped out during the maintenance phase for reasons other than relapse, which was defined a priori as either 1) hospitalization or 2) the addition or increase in allowed psychotropic medication for manic or depressive symptoms.

Individual patient data for all 38 patients was provided by the sponsor. This information consisted of a narrative summary; Y-MRS, MADRS, and CGI scores during the maintenance phase; and the End of Study form completed by the investigator.

The sponsor reexamined the information for these patients and concluded that in 12 cases worsening of bipolar illness could not be absolutely ruled out as a reason for dropout. If these 12 patients were reclassified as relapses, aripiprazole remained superior to placebo in the survival analysis of time to any relapse (p=0.033) and time to manic relapse (p=0.003). The comparison for time to depressive relapse remained non-significant.

Reviewer's Comments

In the above reanalysis, the sponsor appeared to have used very broad criteria for relapse. To verify that the results would not be changed by using stricter criteria, I personally examined the patient data for all 38 patients to identify any where relapse could be reasonably inferred. My examination revealed two patients who, in my opinion, appear to have experienced a relapse prior to discontinuation from the study:

- Patient 118-438 (placebo) and
- Patient 146-496 (aripiprazole).

In both cases, the relapse appeared to be a manic episode. Based on submitted data for these patients, I assumed that the times to relapse were days 128 and 16 of the maintenance phase, respectively. The Statistical Reviewer, Dr. Kun He, then reanalyzed the data assuming that these two patient experienced a manic relapse on the above days. In the survival analyses for any relapse and for manic relapse, aripiprazole was superior to placebo (p-values of 0.0216 and 0.0104, respectively).[2] Thus, the reclassification of these two cases did not change the conclusions of the original analysis.

C. Labeling

The sponsor included proposed labeling in this submission. The following revisions (indicated by strikethrough font or underlining) to the approvable labeling are proposed by the sponsor:

- INDICATIONS/Bipolar Mania, third paragraph:

The efficacy of ABILIFY in maintaining efficacy in patients with Bipolar I Disorder with a recent manic or mixed episode who had been stabilized [ ] was demonstrated in a double-blind, placebo-controlled trial. Prior to entering the [ ] [ ] patients were clinically [ ] 6 consecutive weeks on ABILIFY. Following this [ ] [ ] phase, patients were randomized to either placebo or ABILIFY and monitored for relapse (see CLINICAL PHARMACOLOGY: Clinical Studies). Physicians who elect to use ABILIFY for extended periods [ ] [ ] should periodically re-evaluate the long-term usefulness of the drug for the individual patient (see DOSAGE AND ADMINSITRATION). b(4)

- DOSAGE AND ADMINISTRATION/Bipolar Mania:

Maintenance [ ] b(4)
While there is no body of evidence available to answer the question of how long a patient treated with aripiprazole should remain on it, patients with Bipolar I Disorder who had been symptomatically stable on ABILIFY (15mg/day or

---

[2] The results for depressive and mixed relapse remained unchanged since these two patients were assumed to have had manic relapses. These results were communicated to me by Dr. He in an Email on 1-18-05.

30mg/day with a starting dose of 30mg/day) for at least 6 [ ] weeks and then randomized to ABILIFY (15mg/day or 30mg/day) or placebo [ ] for relapse demonstrated a benefit of such maintenance treatment [ ] it is generally agreed that pharmacological treatment beyond an acute response in mania is desirable, both for maintenance of the initial response and for prevention of new manic episodes, there are no systematically obtained data to support the use of aripiprazole in such longer-term treatment[ ] [ ] b(4)

I have no strong objection to the above changes proposed by the sponsor. However, there are three additional revisions that are warranted:

1) The survival analysis of any relapse and of manic relapse, the latter being one of two key secondary variables, showed a statistically significant advantage of aripiprazole over placebo. However, the survival analysis of depressive relapse, the other key secondary variable, did not demonstrate statistical superiority of aripiprazole over placebo. Without an active control, it is impossible to know whether this represents lack of effectiveness of aripiprazole in delaying depressive relapse or an inability to detect such an effect due to other factors in this trial. Nonetheless, it is important for prescribers to be aware that such an effect for depressive relapse has not been demonstrated. This information regarding the key secondary variables should be added to both the Clinical Trials section of CLINICAL PHARMACOLOGY and INDICATIONS.

2) Final language for the labeling of information regarding cerebrovascular adverse events in elderly patients with dementia under WARNINGS was successfully negotiated with the sponsor on 1-19-05.[3] This language is as follows:

Cerebrovascular Adverse Events, Including Stroke, in Elderly Patients with Dementia
In placebo-controlled clinical studies (2 flexible dose and 1 fixed dose study) of dementia-related psychosis, there was an increased incidence of cerebrovascular adverse events (e.g., stroke, transient ischemic attack), including fatalities, in aripiprazole-treated patients (mean age: 84 years; range: 78-88 years). In the fixed dose study, there

---

[3] See Emails between Steven Hardeman, FDA Project Manager, and Susan Behling, of BMS, on that date.

was a statistically significant dose response relationship for cerebrovascular adverse events in patients treated with aripiprazole. Aripiprazole is not approved for the treatment of patients with dementia-related psychosis. (See also PRECAUTIONS: Use in Patients with Concomitant Illness: Safety Experience in Elderly Patients with Psychosis Associated with Alzheimer's Disease.)

3) The section under ADVERSE REACTIONS entitled Additional Findings Observed in Clinical Trials/Adverse Events in a Long-Term, Double-Blind, Placebo-Controlled Trial should clarify that the cited figures were derived from the long-term study in schizophrenia and that similar findings were observed in the long-term study in bipolar disorder [ ]

[ ] b(4)

D. Phase 4 Commitments

In the cover letter of this submission, BMS commits to providing reports of the two lithium/valproate interaction studies by 6-30-05.

Regarding the adult studies of aripiprazole as add-on therapy and the juvenile animal toxicity studies to support pediatric studies in bipolar disorder, the sponsor has reaffirmed their commitment to complete these trials and submit final study reports on or before 9-30-09 and 6-30-06, respectively.

III. Conclusions and Recommendations

The sponsor has provided reasonable responses to our concerns about the robustness of the efficacy results from study CN138-010. Additionally, both the sponsor and I have proposed some minor revisions to labeling which are described above. Lastly, the sponsor has agreed to the three Phase 4 commitments delineated above.

From a clinical standpoint, once final revisions to labeling have been agreed upon, this supplement may be approved.

Gregory M. Dubitsky, M.D.
January 25, 2005

APPEARS THIS WAY ON ORIGINAL

cc: NDA #21-436
HFD-120 (Div. File)
HFD-120/GDubitsky
/PAndreason
/TLaughren
/DBates
/SHardeman

This is a representation of an electronic record that was signed electronically and this page is the manifestation of the electronic signature.

/s/

Greg Dubitsky
1/25/05 04:11:41 PM
MEDICAL OFFICER
Electronic submission. No hardcopy provided.

Paul Andreason
1/27/05 11:21:39 AM
MEDICAL OFFICER
I agree that this supplement may be approved once mutually agreeable labeling language is negotiated. Please see memo to the file.

M E M O R A N D U M

DEPARTMENT OF HEALTH AND HUMAN SERVICES
PUBLIC HEALTH SERVICE
FOOD AND DRUG ADMINISTRATION
CENTER FOR DRUG EVALUATION AND RESEARCH

DATE: January 27, 2005

FROM: Paul J. Andreason, M.D.
Team Leader, Psychiatric Drug Products
Division of Neuropharmacological Drug Products
HFD-120

SUBJECT: Recommendation for Approvable Action NDA 21-436-S-005 Abilify-Maintenance of Efficacy in Bipolar I Disorder

TO: File NDA 21-436
[Note: This memo should be filed with the January 3, 2005 original submission of this NDA.]

BACKGROUND

The primary reviewer for this "Response to Approvable Action" letter was Greg Dubitsky, MD. His excellent review outlines the Division's previous questions and comments on the sponsor's responses. He concludes that the sponsor adequately addressed the Divisions concerns that were outlined in the November 30, 2004 action letter.

1 I concur with Dr Dubitsky's view that site 93 should be included in the ITT analysis as the basis for approval of this submission.
2 I concur with Dr. Dubitsky' assessment of the efficacy analysis and re-analysis that explores the results of the study based on some deviations in patients' dropout status.
3 The sponsor revised draft labeling differs slightly yet significantly from the Division's previously proposed labeling in the Approvable Action letter of November 30, 2004. This draft labeling implies a maintenance claim of /-weeks. I do not agree with this language. **b(4)**
4 The sponsor has agreed to the Divisions proposed phase IV requirements.

CONCLUSIONS/RECOMMENDATIONS

I agree with Dr Dubitsky that the sponsor has adequately addressed our questions about site 93 and addressed our concerns about proper accounting of patients who dropped out due to relapse versus other reasons. I also note that the sponsor has committed to the Divisions proposed phase IV studies. Negotiating mutually agreeable labeling remains the only outstanding task to perform before approval of this supplement.

LABELING

There are two outstanding labeling topics: the length of the implied maintenance claim, and whether or not differential treatment effects on depression and mania should be mentioned.

- **Length of Maintenance Claim-** The sponsor has submitted draft labeling that refers to the [ ] **b(4)**
[ ]

b(4)

[ ] The Division has adopted the policy that the length of time that patients are stable in the open-label treatment phase shall be used as the description of the length of a maintenance claim that is supported by a relapse-prevention designed study. The double blind "observation" period of the study tests whether treatment with aripiprazole that was continued to that point is still helpful at that point.

- **Differential Effects on Treatment of Mania and Depression**- Dr Dubitsky suggests that we include labeling that states that Abilify was not effective in preventing depressive relapse and the study was positive solely on the basis of preventing manic relapse. I am not sure that this distinction needs to be made; however, there is some precedence for doing this. Lamotrigine labeling for the maintenance treatment of bipolar disorder mentions a sub-analysis that states that the strongest treatment effect was seen for preventing relapse of the depressive state. In the case of Lamotrigine, there were two pooled studies from which this conclusion was drawn, and there was no acute treatment effect. In one study, the patients entered with an acutely depressive index episode and in the other patients entered with an acutely manic index episode.

In this case with Abilify, there is an approved acute treatment with only one maintenance study that was positive. The requirement for only one positive study was approved in advance because Abilify was approved for acute treatment of bipolar disorder. In that study patients entered after having an initial manic episode. I hesitate to draw a conclusion of a differential effect on depression from just this one study of initially manic patients especially when we are only granting an additional 6-weeks efficacy. Nonetheless, I do not strongly object either.

The study was originally designed to observe for both manic and depressive relapses in the primary efficacy analysis. The sponsor was able to pass this overall test and therefore may claim 6-weeks of maintenance efficacy. Had the study only been positive for maintenance of non-mania, but had failed the overall test, we would not have approved it for this indication.

APPEARS THIS WAY
ON ORIGINAL

This is a representation of an electronic record that was signed electronically and this page is the manifestation of the electronic signature.

/s/

Paul Andreason
1/27/05 11:28:14 AM
MEDICAL OFFICER

M E M O R A N D U M

DEPARTMENT OF HEALTH AND HUMAN SERVICES
PUBLIC HEALTH SERVICE
FOOD AND DRUG ADMINISTRATION
CENTER FOR DRUG EVALUATION AND RESEARCH

DATE: February 15, 2005

FROM: Paul J. Andreason, M.D.
Team Leader, Psychiatric Drug Products
Division of Neuropharmacological Drug Products
HFD-120

SUBJECT: Addendum to Supplement 005-Review of Pooled ECG Data for Schizophrenia and Bipolar Patients

TO: File NDA 21-436
[Note: This memo should be filed with the original January 3, 2005 submission of this NDA.]

BACKGROUND

On February 9, 2005 the sponsor submitted a new data analysis of the pooled ECG data from the short term schizophrenia trials with the short term bipolar disorder trials [ ] [ ] This newly provided analysis was part of a response to draft labeling that was proposed by the Division. Item #5 of this e-mail from the company stated, **b(4)**

> "ECGs: During the final labeling discussions for the acute mania approval, Dr. Katz requested that we submit a pooled analysis of the ECG data from the short term schizophrenia and bipolar disorder studies in conjunction with this application. We are sending the pooled ECG results in the attached document to support the change to this section."

The results of the pooled analyses may be found in the appendix of this memo.

The currently approved Abilify labeling states,

> Between group comparisons for pooled, placebo-controlled trials in patients with schizophrenia, revealed no significant differences between aripiprazole and placebo in the proportion of patients experiencing potentially important changes in ECG parameters; in fact, within the dose range of 10 to 30 mg/day, aripiprazole tended to slightly shorten the QTc interval. Aripiprazole was associated with a median increase in heart rate of 4 beats per minute compared to a 1 beat per minute increase among placebo patients.

Review of Pooled ECG Analysis

The analysis of the pooled ECG data does not give data on the dose range of from 10-30-mg [ ] [ ] The new analysis also shows a mean increase in the heart rate of 5 beats per minute instead (BPM) of 4 BPM. Pooled placebo patients continued to show a mean increase in heart rate of 1 BPM in the placebo group. **b(4)**

The mean changes in QTcE and QTcN were roughly equal. The Abilify QTcB showed a lesser decrease over the treatment period than placebo. This is likely due to the greater mean heart rate with Abilify treatment over placebo. Given this higher mean heart rate with Abilify treatment, the QTcB will show a falsely increased duration and is therefore not an appropriate correction method for raw QT. Therefore, QTcN and QTcE are more appropriate correction methods over QTcB.

Outlier analyses of QTcN and QTcE also showed a roughly equal proportion of patients meeting outlier criteria. QTcB showed a higher proportion of patients meeting outlier criteria for the aripiprazole group; however, due to the higher heart rate with aripiprazole the QTcB is not an appropriate correction method.

CONCLUSIONS/RECOMMENDATIONS

Given this new analysis I propose the following labeling changes to the current ECG section of labeling:

> Between-group comparisons for a pooled analysis of placebo-controlled trials in patients with schizophrenia or bipolar mania, revealed no significant differences between aripiprazole and placebo in the proportion of patients experiencing potentially important changes in ECG parameters. Aripiprazole was associated with a median increase in heart rate of 5 beats per minute compared to a 1 beat per minute increase among placebo patients

APPEARS THIS WAY
ON ORIGINAL

Table 7.1.9.1: Incidence of Treatment-Emergent ECG Abnormalities of Potential Clinical Significance: Short-term Placebo-Controlled Studies in Acute Bipolar Mania and Schizophrenia, Safety Sample

ECG Measurement	Number of Patients with Potentially Clinically Significant Abnormality[a] (%) Placebo	Aripiprazole
**Rate**		
Tachycardia	5/ 721(0.7)	3/1315(0.2)
Bradycardia	8/ 721(1.1)	7/1315(0.5)
**Rhythm**		
Sinus tachycardia	5/ 721(0.7)	3/1315(0.2)
Sinus bradycardia	8/ 721(1.1)	7/1315(0.5)
Supraventricular premature beat	0/ 721(0.0)	0/1315(0.0)
Ventricular premature beat	10/ 721(1.4)	13/1315(1.0)
Supraventricular tachycardia	0/ 721(0.0)	0/1315(0.0)
Ventricular tachycardia	0/ 721(0.0)	0/1315(0.0)
Atrial fibrillation	0/ 721(0.0)	0/1315(0.0)
Atrial fibrillation with rapid ventricular response	0/ 721(0.0)	0/1315(0.0)
Atrial flutter	0/ 721(0.0)	0/1315(0.0)
**Conduction**		
1° atrioventricular block	0/ 721(0.0)	2/1314(0.2)
2° atrioventricular block	0/ 721(0.0)	0/1315(0.0)
3° atrioventricular block	0/ 721(0.0)	0/1315(0.0)
Left bundle branch block	0/ 721(0.0)	0/1315(0.0)
Right bundle branch block	0/ 721(0.0)	3/1315(0.2)
Pre-excitation syndrome	0/ 721(0.0)	0/1315(0.0)
Other intraventricular conduction	0/ 721(0.0)	1/1315(0.1)
**Infarction**		
Acute infarction	0/ 721(0.0)	0/1315(0.0)
Subacute (recent) infarct	0/ 721(0.0)	0/1315(0.0)
Old infarction	0/ 721(0.0)	0/1315(0.0)
Myocardial ischemia	0/ 721(0.0)	0/1315(0.0)
Symmetrical T-wave inversion	1/ 721(0.1)	2/1315(0.2)

Table 9.3.3.1: Analysis of QT_{cE} (Fractional Exponent Correction): Short-term Placebo-Controlled Studies in Acute Bipolar Mania and Schizophrenia, Safety Sample

	Placebo	Aripiprazole
Sample Size[a]	714	1294
Baseline QT_{cE} (msec)	390.9	389.7
Mean Change at Endpoint (msec)	-3.11	-3.15
Mean Change at Max QT_{cE} (msec)	-0.84	-0.29
	Number of Patients/Number Assessed (%)	
> 450 msec[b]	2 / 721 (0.3)	2 / 1316 (0.2)
> 500 msec[b]	0 / 721 (0.0)	0 / 1316 (0.0)
≥ 30 msec increase[c]	31 / 717 (4.3)	68 / 1302 (5.2)
≥ 60 msec increase[c]	1 / 717 (0.1)	2 / 1302 (0.2)

** ($P \leq 0.01$), * ($0.01 < P \leq 0.05$) significantly different from placebo. Comparisons of means were done by ANCOVA, controlling for baseline QT_{cE}. Comparisons of proportions were done by Fisher's exact test.

QT_{cE} = Aripiprazole Fractional Exponent Correction Formula ($QT/RR^{0.35}$).

a Includes all patients with both a baseline and an endpoint measurement.

b Includes all patients with an on-study measurement.

c Includes all patients with both a baseline and an on-study measurement.

APPEARS THIS WAY ON ORIGINAL

Table S9.3.3.1A-1: Analysis of QT_{cB} (Bazette's Correction): Short-term Placebo-Controlled Studies in Acute Bipolar Mania and Schizophrenia, Safety Sample

	Placebo	Aripiprazole
Sample Size[a]	714	1294
Baseline QT_{cB} (msec)	405.6	404.1
Mean Change at Endpoint (msec)	-2.70	-0.65*
Mean Change at Max QT_{cB} (msec)	0.02	2.91**
	Number of Patients/Number Assessed (%)	
> 450 msec[b]	21 / 721 (2.9)	37 / 1316 (2.8)
> 500 msec[b]	0 / 721 (0.0)	1 / 1316 (0.1)
≥ 30 msec increase[c]	56 / 717 (7.8)	155 / 1302 (11.9)**
≥ 60 msec increase[c]	8 / 717 (1.1)	11 / 1302 (0.8)

** ($P \leq 0.01$), * ($0.01 < P \leq 0.05$) significantly different from placebo. Comparisons of means were done by ANCOVA, controlling for baseline QT_{cB}. Comparisons of proportions were done by Fisher's exact test.

QT_{cB} = Bazette's Formula ($QT/RR^{0.5}$).

[a] Includes all patients with both a baseline and an endpoint measurement.

[b] Includes all patients with an on-study measurement.

[c] Includes all patients with both a baseline and an on-study measurement.

APPEARS THIS WAY
ON ORIGINAL

Table S9.3.3.1A-2: Analysis of QT_{cN} (Neuropharm Correction): Short-term Placebo-Controlled Studies in Acute Bipolar Mania and Schizophrenia, Safety Sample

	Placebo	Aripiprazole
Sample Size[a]	714	1294
Baseline QT_{cN} (msec)	392.8	391.6
Mean Change at Endpoint (msec)	-3.05	-2.85
Mean Change at Max QT_{cN} (msec)	-0.75	0.06
	Number of Patients/Number Assessed (%)	
> 450 msec[b]	2 / 721 (0.3)	3 / 1316 (0.2)
> 500 msec[b]	0 / 721 (0.0)	0 / 1316 (0.0)
≥ 30 msec increase[c]	30 / 717 (4.2)	71 / 1302 (5.5)
≥ 60 msec increase[c]	1 / 717 (0.1)	2 / 1302 (0.2)

** ($P \le 0.01$), * ($0.01 < P \le 0.05$) significantly different from placebo. Comparisons of means were done by ANCOVA, controlling for baseline QT_{cN}. Comparisons of proportions were done by Fisher's exact test.

QT_{cN} = Aripiprazole Fractional Exponent Correction Formula ($QT/RR^{0.37}$).

a Includes all patients with both a baseline and an endpoint measurement.

b Includes all patients with an on-study measurement.

c Includes all patients with both a baseline and an on-study measurement.

APPEARS THIS WAY
ON ORIGINAL

This is a representation of an electronic record that was signed electronically and this page is the manifestation of the electronic signature.

/s/

Paul Andreason
2/15/05 10:04:23 AM
MEDICAL OFFICER

CENTER FOR DRUG EVALUATION AND RESEARCH

APPLICATION NUMBER:

21-436/S-005 & S-008 & 21-713/S-003

CHEMISTRY REVIEW(S)

CHEMIST REVIEW
OF **SUPPLEMENT**

1. **ORGANIZATION:** HFD-120
2. **NDA** **21-436**
3. **SUPPLEMENT NUMBER AND DATES:** **SE1-005**
   LETTER DATE: 01-26-04
   STAMP DATE: 01-30-04
4. **AMENDMENT/REPORTS/DATES:**
5. **RECEIVED BY CHEMIST:** 02-06-04

6. **APPLICANT NAME & ADDRESS:** Otsuka Pharmaceuticals, Inc.
2440 Research Boulevard
Rockville, MD 20850

7. **NAME OF DRUG:** Abilify™ Tablets

8. **NONPROPRIETARY NAME:** Aripiprazole

9. **CHEMICAL NAME and STRUCTURE:** 7-[4-[4-(2,3-Dichlorophenyl)-1-piperazinyl]butoxy]-3,4-dihydrocarbostyril

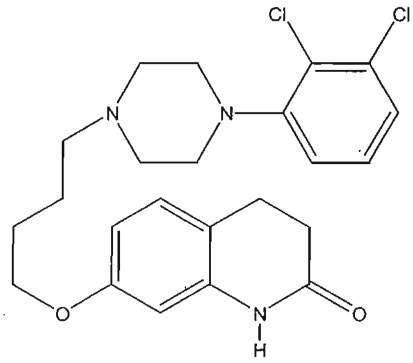

10. **DOSAGE FORMS:** Tablets

11. **POTENCY:** 2, 5, 10, 15, 20 and 30 mg

12. **PHARMACOLOGICAL CATEGORY:** Schizophrenia

13. **HOW DISPENSED:** __X__(Rx) _______(OTC)

14. **RECORD and REPORTS CURRENT:** __X__Yes _________No

15. **RELATED IND/NDA/DMF:** n/a

16. **SUPPLEMENT PROVIDES FOR:** This supplement provides for the drug product, Abilify™ Tablets, to be use for maintaining stability in patients with Bipolar I Disorder.

17. **ADDITIONAL COMMENTS:** The applicant has not identified any changes to the CMC portion of this application.

18. **CONCLUSIONS & RECOMMENDATIONS:**
The applicant has provided adequate information to support this change. From a CMC perspective, it is recommended that this supplement be **APPROVED**.

cc: NDA 21-436 Division file
TOliver
SMclamore
DBates

Review Notes:

1. DRUG SUBSTANCE
NDA 21-436 was approved November 15, 2002. The applicant has not identified any additional changes to the drug substance portion of this application.

   Evaluation: Adequate

2. DRUG PRODUCT
NDA 21-436 was approved November 15, 2002. The applicant has not identified any additional changes to the drug product portion of this application

   Evaluation: Adequate

3. PACKAGE INSERT AND LABELING

   Evaluation: Adequate
The package insert was reviewed and there were no changes to the Description or to the How Supplied Section of the package insert.

4. ENVIRONMENTAL ASSESSMENT
Under item 20 in the electronic document, the applicant requested a categorical exclusion for the environmental assessment based on 21CFR 25.15 (d) and 21 CFR 25.31(b). The applicant further indicated that there are no known extraordinary circumstances that will adversely effect the environment.

   Evaluation: Adequate
Based on 21 CFR 25.31(b), a categorical exclusion should be granted as the expected introduction of the substance at the point of entry into the aquatic environment will be below 1 ppb.

Appears This Way
On Original

This is a representation of an electronic record that was signed electronically and this page is the manifestation of the electronic signature.

/s/

Sherita McLamore
2/11/04 03:47:03 PM
CHEMIST

Thomas Oliver
2/12/04 06:49:35 AM
CHEMIST

CENTER FOR DRUG EVALUATION AND RESEARCH

APPLICATION NUMBER:

21-436/S-005 & S-008 & 21-713/S-003

PHARMACOLOGY REVIEW(S)

PHARMACOLOGY/TOXICOLOGY MEMORANDUM

NDA number: **21-436**
Sequence number/date/type of submission: SE1-005/ January 28, 2004
Information to sponsor: No

Sponsor and/or agent:
Otsuka Pharmaceutical Company, Ltd.
2440 Research Boulevard, Rockville, MD 20850
Phone (301) 497-0900

Reviewer name: Sonia Tabacova, Ph.D.
Division name: Neuropharmacological Drug Products, HFD #: 120
Review completion date: October 28, 2004

Drug:

Trade name: ABILIFY™
Generic name: Aripiprazole
Code name: OPC-14597, BMS-337039
Chemical name: *7-[4-[4-(2, 3-dichlorophenyl)-1-piperazinyl]butoxy]-3,4-dihydro-2(1H)-quinolinone*
Formulation: Tablet (5 mg, 10 mg, 15 mg, 30 mg)

Drug class: Psychotropic (partial D_2 and $5HT_{1A}$ agonist, $5HT_2$ antagonist)
Indication: Maintaining stability in patients with Bipolar I Disorder

Relevant INDs/NDAs/DMFs: NDA 21-436 for ABILIFY™ (aripiprazole) tablets for treatment of schizophrenia (Approved); NDA 21-436 S-002[ ] for use of for ABILIFY™ in the treatment of acute mania in patients with Bipolar I Disorder (Approved). b(4)

The preclinical section of the present application contains two reports. Both are pharmacological studies (completed between June 30 and September 2, 2003):

- Study 014065 - Analysis of molecular mechanisms of aripiprazole and other antipsychotics on prolactin production: Special emphasis on Dopamine D2 receptors (Preliminary Studies)
- Study 015812 - Validation of the assay method for OPC-14597 in 1% lactic acid solution by high performance liquid chromatography.

Study 014065: Analysis of molecular mechanisms of aripiprazole and other antipsychotics on prolactin production: Special emphasis on Dopamine D2 receptors (Preliminary Studies)

Background: Dopamine is known to regulate prolactin release in lactotroph cells through D2 receptors [Ben-Jonathan, N. Dopamine: A prolactin-inhibiting hormone. Endocr. Rev.

1985, 6, 564-589 (as cited by the sponsor)]. D2 receptor has two isoforms, the long form (D2L) and the short form (D2S), co-expressed in a ratio favoring the D2L. According to literature data cited by the sponsor, the long form acts mainly at post-synaptic sites and the short form serves pre-synaptic autoreceptor function [Usiello et al. Distinct functions of the two isoforms of dopamine D2 receptors. Nature, 2000, 408, 199-203 (as referenced by the sponsor)]. Aripiprazole exhibits an antagonism to post-synaptic D2 receptors, and agonistic activity at pre-synaptic dopamine autoreceptors [Elsworth J.D. and Roth, R.H. Dopamine autoreceptor pharmacology and function. In: The Dopamine Receptors. Eds.: Neve, K.A. and Neve R. L., Humana Press Inc., Totowa 223-265, 1997 (as cited by the sponsor)].

The submitted study demonstrates that aripiprazole is a partial D2 agonist in pituitary cells *in vitro*. The authors transduced retrovirally the short or the long form of human dopamine D2 receptor gene into rat pituitary cell line (GH4C1) and examined the effect of aripiprazole on prolactin release and cAMP accumulation in either D2L or D2S receptor expressing GH4C1 cells. Aripiprazole inhibited forskolin-stimulated prolactin release in both D2Lor D2S receptors, however the maximal inhibition of prolactin release was less than that of dopamine. In addition, aripiprazole antagonized the suppression attained by dopamine in both cells. The maximal inhibition of prolactin release and cAMP level by aripiprazole were greater for the D2S- than for D2L- receptor expressing cells. Saturation binding analysis showed that the maximal binding capacity was approximately 4-fold higher at the D2S- than at D2L- receptor expressing cells, while affinity was similar at these cells. The results indicate that "aripiprazole acts as a partial agonist at both D2S and D2L receptors expressed on rat pituitary cells with high affinity, and that its agonist-antagonist properties may depend upon the amount of D2 binding capacity on the cells."

Study 015812: Validation of the assay method for OPC-14597 in 1% lactic acid solution by high performance liquid chromatography.
This study validated a modified HLPC method for analyzing the purity of OPC-14597 (dissolved in 1% aqueous solution in lactic acid) with regard to specificity, linearity, accuracy, reproducibility, precision, and stability during the assay period.

Conclusion: None of the preclinical studies submitted with this application provide information that can have an impact on ABILIFY™ labeling.

Recommendation: No action indicated

CENTER FOR DRUG EVALUATION AND RESEARCH

APPLICATION NUMBER:

21-436/S-005 & S-008 & 21-713/S-003

STATISTICAL REVIEW(S)

U.S. DEPARTMENT OF HEALTH AND HUMAN SERVICES
FOOD AND DRUG ADMINISTRATION
CENTER FOR DRUG EVALUATION AND RESEARCH
OFFICE OF PHARMACOEPIDEMIOLOGY AND STATISTICAL SCIENCE
OFFICE OF BIOSTATISTICS

STATISTICAL REVIEW AND EVALUATION

Clinical Studies

**NDA/Serial Number:**	21-436/S-005
**Drug Name:**	Abilify ® (aripiprazole)
**Indication:**	Bipolar
**Sponsor:**	Bristol_myers Squibb
**Date:**	1/28/2004
**Review Priority:**	Standard
**Biometrics Division:**	Biometrics I (HFD 710)
**Statistical Reviewer:**	Kun He
**Concurring Reviewers:**	Kun Jin, , Ph.D.,Team Leader
	James Hung, Ph.D., Acting Deputy Director
**Medical Division:**	Neuropharmacological Drug Products (HFD 120)
**Clinical Team:**	Teresa Podruchny, M.D., Clinical Reviewer
	Paul Andreason, M.D., Team Leader
	Russell Katz, M.D., Director
**Project Manager:**	Doris Bates, Ph.D.

Keywords: Log-rank test, Bipolar, Aripiprazole

TABLE OF CONTENTS

1. Executive Summary **3**

1.1 Conclusions and Recommendations *3*

1.2 Brief Overview of Clinical Studies *3*

1.3 Statistical Issues and Findings *3*

2. Introduction **4**

2.1 Overview *4*

2.2 Data Sources *4*

3. Statistical Evaluation **4**

3.1 Evaluation of Efficacy *4*

3.1.1 Objective 4
3.1.2 Study Design 4
3.1.3 Efficacy Measures 5
3.1.4 Statistical Analysis Plan 6
3.1.5 Protocol Amendments and Deviations 6
3.1.6 Study Population 7
3.1.7 Sponsor's Efficacy Results 14
3.1.8 Reviewer's Analysis 17
3.1.8.1 Primary and Secondary Analyses 17
3.1.8.2 All-Cause Analysis 17
3.1.8.3 Protocol Deviation 18
3.1.8.4 Analysis by Country 18

3.2 Evaluation of Safety *19*

4. Findings in Special/Subgroup Populations **20**

4.1 Gender, Race, and Age *20*

4.2 Other Special/Subgroup Populations *20*

5. Summary and Conclusions **20**

5.1 Statistical Issues and Collective Evidence *20*

5.2 Conclusions and Recommendations *21*

Statistical Review and Evaluation

1. Executive Summary

1.1 Conclusions and Recommendations

The conclusion is that the primary analysis for the time from randomization to relapse during the maintenance phase is significant comparing aripiprazole and placebo in evaluating subjects with Bipolar I Disorder but one should consider whether the quality of operations in center 093 is high, which was suggested to be inspected by DSI, when making final decision.

1.2 Brief Overview of Clinical Studies

This was a randomized, double-blind, multicenter, placebo-controlled trial in USA, Mexico, and Argentina, evaluating the use of aripiprazole in the maintenance of stability of patients with Bipolar I Disorder. There were 3 phases in this study: a Stabilization Phase, a Maintenance Phase, and an Extension Phase. A total of 633 subjects enrolled in the study, and resulting 161 randomized to maintenance phase. ITT included 83 subjects in placebo group and 77 subjects in aripiprazole group. The primary efficacy endpoint was the time from randomization to relapse during the maintenance phase. The primary analysis is log-rank test.

1.3 Statistical Issues and Findings

The primary analysis is log-rank test which gives p-value .0199 where there were 36 out of 83 (43%) relapsed in placebo, and 19 out of 77 (25%) relapsed in aripiprazole groups, respectively.

One issue is whether the study is robust because center 093 in Mexico, where there were 7 in placebo and 6 in aripiprazole groups, respectively, had 5 (71%) relapsed in placebo and 0 (0%) relapsed in aripiprazole groups, respectively. The primary analysis is not significant after removing center 093.
One should consider whether the quality of operations in center 093 is high, which was suggested to be inspected by DSI, when making final decision.

Appears This Way
On Original

2. Introduction

2.1 Overview

The current submission, NDA 21436 S005 was to support aripiprazole in treating subjects with Bipolar I Disorder. The study was a randomized, double-blind, multicenter, placebo-controlled trial in USA, Mexico, and Argentina, evaluating the use of aripiprazole in the maintenance of stability of patients with Bipolar I Disorder. There were 3 phases in this study: a Stabilization Phase, a Maintenance Phase, and an Extension Phase. Stabilization phase, maintenance phase, and extension phase. A total of 633 enrolled in the study, and resulting 161 randomized to maintenance phase.

2.2 Data Sources

The path to the CDER Electronic Document Room (EDR) is:

\\Cdsesub1\n21436\S_005\2004-01-28

3. Statistical Evaluation

3.1 Evaluation of Efficacy

Texts, tables, and graphs in Sections 3.1.1 – 3.1.7 are mainly adapted from the Applicant's Study Report.

3.1.1 Objective

The primary objective of this study was to compare the maintenance of stability of aripiprazole versus placebo as measured by the time to relapse (i.e., discontinuation due to lack of efficacy) during the Maintenance Phase. Patients were discontinued from the study due to lack of efficacy if they were hospitalized for manic or depressive symptoms or required an addition to or increase in their allowed psychotropic medications.

3.1.2 Study Design

This was a randomized, double-blind, multicenter, placebo-controlled trial evaluating the use of aripiprazole in the maintenance of stability of patients with Bipolar I Disorder. The patient sample was diagnosed with Bipolar I Disorder, according to Diagnostic and Statistical Manual of Mental Disorders, Fourth Edition (DSM-IV) criteria, and had recently experienced a manic or mixed episode.

There were 2 routes of entry into this study. Patients who had recently completed a 3-week acute mania study of aripiprazole (CN138007, CN138009, CN138062, CN138074, or CN138077) were eligible to enter this study. Also, patients who had recently experienced (≤ 3 months) a manic or mixed episode requiring hospitalization and treatment, but who had not participated in a 3-week aripiprazole study were eligible to enter this study. Patients who were eligible to participate in a 3-week aripiprazole acute mania study, but declined participation, were considered for this maintenance of stability study. Patients entered the study as inpatients or as outpatients.

There were 3 phases in this study: a Stabilization Phase, a Maintenance Phase, and an Extension Phase.

Stabilization Phase: During this phase patients received open-label aripiprazole treatment with a starting dose of 30 mg/day. The dose could be decreased to 15 mg/day at any time, if necessary for tolerability. The Stabilization Phase was from 6 to 18 weeks in duration, with visits every 2 weeks. Patients continued in the Stabilization Phase until symptoms of their Bipolar Disorder were stable. Stability was defined by a Young-Mania Rating Scale (Y-MRS) Score of ≤ 10 and a Montgomery-Asberg Depression Rating Scale (MADRS) Score of ≤ 13 during 4 consecutive visits over a minimum of 6 weeks.

Patients entered the Maintenance Phase only after meeting stabilization criteria for 4 consecutive weeks and after remaining in the Stabilization Phase for a minimum of 6 weeks. Patients who entered the study and did not roll over directly from an acute mania study participated in a screening period of up to 28 days before entering the Stabilization Phase. There was a minimum 1 day wash-out period for antipsychotics. All antipsychotic treatment and psychotropic medications outside of those prescribed by this protocol were discontinued during the screening phase.

Maintenance Phase: Patients meeting stabilization criteria during the Stabilization Phase were randomized to either aripiprazole or placebo. Patients assigned to aripiprazole started the Maintenance Phase at the same dose they were taking at the end of the Stabilization Phase. The dose of aripiprazole was 15 mg/day or 30 mg/day and could be changed at any time during the study, as necessary based on therapeutic effect and tolerability. Patients continued in the Maintenance Phase of the study for up to 26 weeks (6 months).

Extension Phase: Patients who completed 26 weeks of the Maintenance Phase without a relapse had the option to continue on their current double-blind study drug treatment in the Extension Phase for an additional 74 weeks (17 months).

3.1.3 Efficacy Measures

The primary efficacy outcome measure was the time to relapse (as defined by discontinuation due to lack of efficacy) from randomization in the 26-week Maintenance Phase. Patients were

discontinued from the study due to lack of efficacy if they were hospitalized and/or required an addition to or increase in their allowed psychotropic medications, other than study medication, for manic or depressive symptoms.

Secondary efficacy measures included the time to manic relapse and the time to depressive relapse during the Maintenance Phase.

Relapses were classified into 3 categories: manic type, depressive type, or mixed type. A relapse was classified as a manic or depressive type if the patient was hospitalized for manic or depressive symptoms or required an addition to or increase in allowed psychotropic medication, other than study medication, for manic or depressive symptoms, as indicated on the relapse CRF page. A relapse was classified as a mixed type if the patient required intervention for both manic and depressive symptoms, as indicated on the relapse CRF page. The numbers and percentages of relapses falling into each of the 3 categories are presented by treatment group.

3.1.4 Statistical Analysis Plan

For time-to-event analyses, such as time to relapse, the log-rank test was used to compare the survival distributions of the 2 treatment groups. The estimated survival curves for each treatment group were obtained from the Kaplan-Meier estimates. Analysis of the primary efficacy measure will be performed using the Maintenance Safety Sample, which comprises all patients in the Randomized Sample who take at least one dose of study medication during the double-blind treatment phase, as identified on the dosing record. Other efficacy analyses will be performed using the Maintenance Efficacy Sample, which comprises all patients who are in the Maintenance Safety Sample Phase and have at least one post-randomization efficacy evaluation.

Sample size calculation: it was expected that the 6-month placebo relapse rate would be 45% and the aripiprazole relapse rate would be 20%. A total of 45 events would be required to yield 87% power to detect a 25% difference in the percentage of patients relapsing between placebo and the aripiprazole treatment groups, assuming these relapse rates, a dropout rate for reasons other than relapse of 18%, and a 2-sided test at the 0.05 level. These assumptions were based on results from 3 previous studies. Based on these assumptions, it was expected that 152 patients would have to be randomized to obtain 150 evaluable patients (75 per treatment group) to yield 45 events (number of patients who relapsed). The hazard ratio for these relapse rates and sample size was 2.7.

3.1.5 Protocol Amendments and Deviations

Protocol Amendment: There were 6 amendments and 4 administrative letters during the study. Amendments 4, 5, and 6 affected the analysis of the study.

Amendment 4 added an Extension Phase so that patients may have continued on double-blind therapy upon completion of 26 weeks of the Maintenance Phase. In addition, data handling for those

patients who inadvertently received unblended Maintenance Phase study medication was addressed. The amendment clarified that these patients were to be replaced and only safety data were to be analyzed.

Amendment 5 modified the criteria for closing and completing the study. The rationale of the amendment was to incorporate new information on maintenance treatment that became available after the initiation of the study. The original power calculations, which assumed relapse rates of 17% for aripiprazole and 47% for placebo, were based on a 30% difference in the expected relapse rate between aripiprazole and placebo; however, new information from maintenance treatment studies in bipolar patients indicated that the differences in the relapse rates between an active treatment and placebo might be less than 30%. Thus, new expected relapse rates for aripiprazole and placebo were calculated and were based on the assumption of a 25% expected difference in relapse rates between the 2 treatments, a clinically meaningful difference. It was then assumed that the placebo relapse rate would be approximately 45% and the aripiprazole relapse rate would be approximately 20%. Based on these new sample size calculations, the number of patients needed for relapse changed from 36 to 45.

Amendment 6 added 2 key secondary efficacy analyses to the study: time to manic relapse and time to depressive relapse. These were to be analyzed using a hierarchical testing procedure.

Protocol Deviations: On December 7, 2000 it was discovered that blinded supplies for the Maintenance Phase of the study were labeled in error with product information. Due to this labeling error, randomization was closed, and the 35 patients who had been randomized into the Maintenance Phase of the study at that time were discontinued from the study. Randomization was resumed on January 1, 2001, after appropriately repackaged supplies were available. The 35 patients who received unblinded study medication were not included in the analyses of efficacy or the Maintenance Safety Sample, but were analyzed separately. The safety data for these patients are presented in supplemental tables.

3.1.6 Study Population

A total of 633 patients were enrolled in the study, and 567 entered the Stabilization Phase, where 361 (64%) discontinued from this Phase, and 206 (37%) completed. Of the 206 patients who completed the Stabilization Phase, 161 were randomized to double-blind treatment in the Maintenance Phase. An additional 35 patients were randomized to the double-blind Maintenance Phase, but are not included in the Randomized Sample because of a labeling error, as described in Protocol deviation section. Ninety-four (58%) of the 161 patients discontinued from the Maintenance Phase of the study: 55 (66%) placebo-treated patients and 39 (50%) aripiprazole-treated patients. The most common reason for discontinuing from therapy in both treatment groups was because of lack of efficacy (43% placebo; 24% aripiprazole).

The disposition of all patients enrolled in the study is presented by treatment and study phase in Table 3.1.6.1.

Table 3.1.6.1 Disposition of Patients

Patient Status	Number (%) of Patients Placebo	Aripiprazole	Total
**Enrolled**	n/a	n/a	633
Baseline failures	n/a	n/a	66
**Entered Stabilization Phase**	n/a	567	567
Discontinued Stabilization Phase	n/a	361 (64)	361 (64)
Adverse event[a]	n/a	126 (22)	126 (22)
Lack of efficacy	n/a	66 (12)	66 (12)
Subject withdrew consent	n/a	66 (12)	66 (12)
Subject unreliability	n/a	25 (4)	25 (4)
Lost to follow-up	n/a	49 (9)	49 (9)
Pregnancy	n/a	1	1
Death	n/a	1	1
Other known cause[b]	n/a	27 (5)	27 (5)
Completed Stabilization Phase	n/a	206 (37)	206 (37)
**Randomized to Double-Blind Treatment**[c]	83	78	161
Discontinued from Maintenance	55 (66)	39 (50)	94 (58)
Lack of Efficacy	36 (43)	19 (24)	55 (34)
Subject withdrew consent	6 (7)	6 (8)	12 (7)
Subject Unreliability	5 (6)	3 (4)	8 (5)
Adverse Event[a]	1 (1)	5 (6)	6 (4)
Lost to Follow-up	1 (1)	1 (1)	2 (1)
Missing	0	1 (1)	1 (1)
Other known cause[d]	6 (7)	4 (5)	10 (6)
Completed Maintenance Phase	28 (34)	39 (50)	67 (42)
**Entered Extension**	27	39	66
Discontinued from Extension	22 (81)	32 (82)	54 (82)
Lack of efficacy[e]	7 (26)	5 (13)	12 (18)
Subject withdrew consent	3 (11)	8 (21)	11 (17)
Subject unreliability	2 (7)	2 (5)	4 (6)
Lost to follow-up	0	1 (3)	1 (2)

Patient Status	Number (%) of Patients		
	Placebo	Aripiprazole	Total
Pregnancy	0	1 (3)	1 (2)
Adverse event[a]	0	1 (3)	1 (2)
Other known cause[f]	10 (37)	14 (36)	24 (36)
Completed Extension Phase	5 (19)	7 (18)	12 (18)

Protocol CN138010

Source: Appendix 8.1A

[a] Data obtained from end-of-study CRF page.

[b] During the Stabilization Phase, "other known causes" included such things as screen failure, positive drug screen, did not meet inclusion criteria, and site closed by sponsor because prespecified number of relapses had been attained. In addition, 1 patient discontinued because of an SAE (thought suicidal) and was included in this category.

[c] Forty-six patients completed the Stabilization Phase: 35 patients were randomized to the double-blind Maintenance Phase but were discontinued because of a labeling error; 11 patients discontinued because of other reasons (eg, Y-MRS or MADRS criteria not met for randomization, reason not stated) and were not randomized to the double-blind Maintenance Phase; and 1 patient (Patient 138010-141-266) did not complete the Stabilization Phase but was randomized in error to double-blind treatment.

[d] During the Maintenance Phase, "other known causes" included positive drug screen, patient relocating, and site closed by sponsor because prespecified number of relapses had been attained.

[e] Patient 138010-147-604 relapsed during the Extension Phase, according to the relapse form, but discontinued from the Extension Phase because of "other known cause" according to the end-of-study form.

[f] During the Extension Phase, the primary "other known cause" (study closed by sponsor because prespecified number of relapses had been attained)

For the double-blind Maintenance Phase of the study, the patients were similarly distributed between the placebo and aripiprazole groups. Since a greater percentage of aripiprazole-treated patients (50%) than placebo-treated patients (34%) completed the Maintenance Phase, there were more patients in the aripiprazole group in the Extension Phase than in the placebo group. The distribution of all randomized patients within each of the patient samples is presented by treatment group in Table 3.1.6.2.

Table 3.1.6.2 Number of Patients in Samples

Sample	Placebo N	Aripiprazole N	Total N
Stabilization (Enrolled)	n/a	633	633
Stabilization Safety	n/a	553	553
Stabilization Efficacy	n/a	514	514
Excluded due to Labeling Error	15	20	35
Randomized	83	78	161
Maintenance Safety	83	77	160
Maintenance Efficacy	82	76	158
Extension Safety	27	39	66
Extension Efficacy	27	38	65

Protocol CN138010
Source: Appendix 8.1A

One patient 138010-141-266 was excluded from Maintenance Safety Sample because of pregnancy. Two patients were excluded from Maintenance Efficacy Sample: one placebo 138010-132-350 was due to withdrawal of subject consent/patient request; one aripiprazole 138010-122-205 was due to inclusion/exclusion criteria not met.

There were 35 patients excluded from the Samples because of a labeling error. After these 35 patients had been randomized to the Maintenance Phase, it was discovered that blinded supplies for this phase were labeled in error with product information. Table 3.1.6.3 presents the patient identification number of these patients.

Appears This Way
On Original

Table 3.1.6.3 Discontinuation Reasons for Patients Excluded Due to Labeling Error

Treatment Group	Patient Number	Discontinuation Reason[a]	Study Day Discontinued[b]
Placebo	138010-3-2	Other	268
	138010-5-32	Lost to follow-up	132
	138010-8-69[b]	Treatment failure/lack of efficacy	155
	138010-49-3	Withdrawal of subject consent/patient request	124
	138010-49-66	Other	143
	138010-53-63	Other	182
	138010-63-87	Other	127
	138010-66-10[b]	Treatment failure/lack of efficacy	139
	138010-72-31	Treatment failure/lack of efficacy	176
	138010-72-51	Treatment failure/lack of efficacy	158
	138010-73-7	Other	245
	138010-82-57	Treatment failure/lack of efficacy	127
	138010-92-81	Other	150
	138010-93-102	Other	98
	138010-93-114	Other	95
Aripiprazole	138010-3-1	Other	281
	138010-3-33	Other	224
	138010-12-16	Non-compliance	204
	138010-32-36	Withdrawal of subject consent/patient request	111
	138010-33-124	Treatment failure/lack of efficacy	57
	138010-49-20	Other	223
	138010-49-41	Other	183
	138010-53-113	Lost to follow-up	82
	138010-54-98[c]	Treatment failure/lack of efficacy	48
	138010-64-104	Other	93
	138010-68-6	Other	62
	138010-72-54	Other	166
	138010-92-91	Other	111
	138010-92-121	Other	79

Treatment Group	Patient Number	Discontinuation Reason[a]	Study Day Discontinued[b]
	138010-93-101	Other	99
	138010-94-60	Non-compliance	93
	138010-100-72	Other	149
	138010-109-27	Non-compliance	112
	138010-111-133	Other	63
	138010-118-77	Other	159

Protocol CN138010

Source: Appendix 8.1A, 9.1

a The discontinuation reason of "other" was the labeling error.

b Patient experienced an SAE. The narrative for this patient may be found in Supplemental Table S.12.3G.

c Study day was from beginning of dosing in the Stabilization Phase.

In the Randomized Sample, the demographic characteristics of the treatment groups were similar with the exception of gender: more men were randomized to the aripiprazole group (38%) than to the placebo group (28%), and conversely, fewer women were randomized to the aripiprazole group (62%) than to the placebo group (72%). Demographic characteristics of the Enrolled the Randomized Sample is presented by treatment group in Table 3.1.6.4.

Appears This Way
On Original

Table 3.1.6.4 Demographic Characteristics: Randomized Sample

Variable		Placebo N = 83	Aripiprazole N = 78	Total N = 161
Age (years)	Mean	40.3	39.0	39.6
	Median	40.0	38.5	40.0
	Range	18.0-62.0	18.0-80.0	18.0-80.0
	SE	1.2	1.5	0.9
Gender N (%)	Male	23 (28)	30 (38)	53 (33)
	Female	60 (72)	48 (62)	108 (67)
Race N (%)	White	56 (67)	48 (62)	104 (65)
	Hispanic/Latino	17 (20)	20 (26)	37 (23)
	Black	5 (6)	5 (6)	10 (6)
	Asian/Pacific Islander	4 (5)	2 (3)	6 (4)
	American/Alaskan Native	0	1 (1)	1 (1)
	Other	1 (1)	2 (3)	3 (2)

Protocol CN138010
Source: Appendix 8.3

The psychiatric history of bipolar disorder of patients in the Randomized Samples is presented in Tables 3.1.6.5.

Table 3.1.6.5 Psychiatric History of Bipolar Disorder: Randomized Sample

Variable		Placebo N = 83	Aripiprazole N = 78	Total N = 161
Age current episode began	Mean	40.4	39.1	39.8
(derived from date of onset	Median	40.0	39.0	40.0
of episode)	Minimum-Maximum	18.0-62.0	18.0-80.0	18.0-80.0
	SE	1.2	1.5	1.0
	Missing	3	1	4
Rapid Cycling	Yes	14 (17)	14 (18)	28 (17)
	No	69 (83)	64 (82)	133 (83)
Current Episode is N (%)	Manic	65 (78)	48 (62)	113 (70)
	Mixed	18 (22)	30 (38)	48 (30)

Protocol CN138010
Source: Appendices 8.4A, 8.4B

Table 3.1.6.6 lists number of patients receiving study medication and mean and range of daily dose for the Maintenance Safety Sample.

Table 3.1.6.6 Number of Patients Receiving Study Medication and Mean and Range of Daily Dose: Maintenance Safety Sample

	Placebo		Aripiprazole		
Day (Interval)	N	Mean (no. tablets)	N	Mean (mg)	Range of Daily Dose[a]
Number (%)of patients with endpoint dose of 15 mg, Maintenance Safety Sample					27 (35%)
Number (%)of patients with endpoint dose of 30 mg, Maintenance Safety Sample					50 (65%)
Endpoint for Patients Who Relapsed	36	1.65	19	25.71	12.86 - 30.00
Number (%)of patients with endpoint dose of 15 mg					4 (21%)
Number (%)of patients with endpoint dose of 30 mg					15 (79%)
Endpoint for Patients Who Completed	28	1.60	39	23.85	15.00 - 30.00
Number (%)of patients with endpoint dose of 15 mg					16 (41%)
Number (%)of patients with endpoint dose of 30 mg					23 (59%)

Protocol CN138010

Source: Appendix 9.1

[a] Range of daily doses take into account patients who deviated from the dose specified in the protocol or who were noncompliant.

3.1.7 Sponsor's Efficacy Results

The primary efficacy endpoint was the time from randomization to relapse during the Maintenance Phase (as defined by discontinuation due to lack of efficacy). Patients were discontinued from the study because of lack of efficacy if they were hospitalized and/or required an addition to or increase in their allowed psychotropic medications, other than study medication, for manic or depressive symptoms.

As shown in Table 3.1.7.1 and Figure 3.1.7.1, patients in the placebo group relapsed sooner than patients in the aripiprazole group, as evidenced by the log-rank P-value 0.020. Moreover, the probability of not experiencing relapse by Week 26 was 49% for placebo-treated patients and 72% for aripiprazole-treated patients.

Table 3.1.7.1 Time from Randomization to Relapse, Maintenance Safety Sample

Time from Randomization to Relapse[a,b]	
Log-rank test p-value for equality of survival curves	0.020
Hazard ratio (Aripiprazole:Placebo), 95% CI[c]	0.523 (0.300, 0.913)

Patients Not Experiencing Relapse

	Placebo		Aripiprazole	
Study Week	Number at Risk	Proportion[d] (SE)[e]	Number at Risk	Proportion[d] (SE)[e]
0	83	1.00 (0.00)	77	1.00 (0.00)
1	74	0.91 (0.03)	75	0.97 (0.02)
2	71	0.88 (0.04)	73	0.95 (0.03)
3	64	0.84 (0.04)	61	0.91 (0.03)
4	59	0.80 (0.05)	58	0.89 (0.04)
6	54	0.76 (0.05)	52	0.81 (0.05)
8	53	0.74 (0.05)	49	0.77 (0.05)
10	48	0.70 (0.05)	47	0.75 (0.05)
12	48	0.70 (0.05)	44	0.73 (0.05)
14	43	0.65 (0.06)	42	0.72 (0.06)
16	39	0.61 (0.06)	42	0.72 (0.06)
18	36	0.58 (0.06)	42	0.72 (0.06)
20	32	0.53 (0.06)	42	0.72 (0.06)
22	30	0.49 (0.06)	42	0.72 (0.06)
24	30	0.49 (0.06)	42	0.72 (0.06)
26	30	0.49 (0.06)	42	0.72 (0.06)

Protocol CN138010

Source: Appendix 10.1A

NOTE: Median time to relapse and 95% CIs were not reported, as they were not estimable in the aripiprazole group.

[a] Defined as discontinuation due to lack of efficacy.

[b] For Patients 138010-118-214 and 138010-147-604, who were randomized in error upon entry into the Stabilization Phase, time from randomization to relapse is measured from the first day of dosing in the Maintenance Phase.

[c] Cox's proportional hazards model. Hazard ratio = aripiprazole:placebo. A hazard ratio < 1 favors aripiprazole.

[d] Kaplan-Meier Estimated Survival Rates.

[e] SE using Greenwood's formula from PROC LIFETEST.

Appears This Way
On Original

Figure 3.1.7.1 Time from Randomization to Relapse, Maintenance Safety Sample

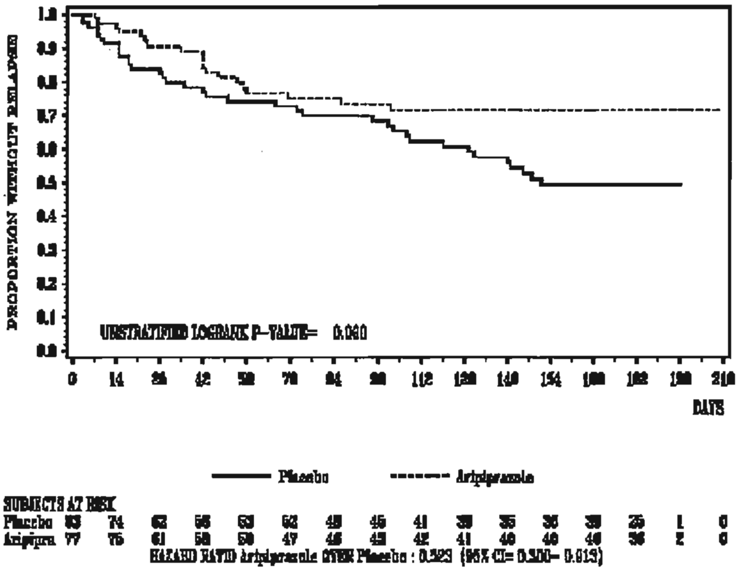

Key secondary efficacy measures were the time to manic relapse and the time to depressive relapse during the Maintenance Phase. For these analyses, a hierarchical testing procedure was used. If aripiprazole was significant versus placebo in the primary efficacy analysis, then testing of the key secondary endpoints proceeded sequentially. First, time to manic relapse was tested and if this was significant, then time to depressive relapse was tested.

The results, as displayed in Table 3.1.7.2, showed a statistically significant difference in favor of aripiprazole in time to manic relapse (p = 0.008), but no significant difference in time to depressive relapse (p = 0.684) during the Maintenance Phase.

Appears This Way
On Original

Table 3.1.7.1 Analysis of Time to Manic and Depressive Relapse: Maintenance Safety Sample

Relapse Type	Analysis of Time to Relapse[a]
Manic N = 160	
Log-rank test p-value for equality of 2 survival curves	0.008
Hazard ratio[b] (Aripiprazole : Placebo), 95% CI	0.309 (0.123, 0.774)
Depressive N = 160	
Log-rank test p-value for equality of 2 survival curves	0.684
Hazard ratio[b] (Aripiprazole : Placebo), 95% CI	0.833 (0.345, 2.011)

Protocol CN138010
Source: Appendices 10.2A-1, 10.2A-2

[a] For Patients 138010-118-214 and 138010-147-604, who were randomized in error upon entry into the Stabilization Phase, time from randomization to relapse is measured from the first day of dosing in the Maintenance Phase.

[b] Cox proportional hazard's model. Hazard ratio = aripiprazole:placebo. A hazard ratio < 1 favors aripiprazole.

3.1.8 Reviewer's Analysis

3.1.8.1 Primary and Secondary Analyses

The reviewer validated the sponsor's analysis according to the protocol. The log-rank test for the primary analysis gives p-value .0199. The log-rank for the key secondary analysis gives p-value .008 for the time to a manic relapse, where there were 19 relapse in placebo and 6 relapse in aripiprazole groups, respectively; and .6838 for the time to a depressive relapse, where there were 11 relapse in placebo and 9 relapse in aripiprazole groups, respectively .

There is one subject 00093 00533 who was randomized to Aripiprazole but actual treatment was placebo. If using randomization code, the subject was in Aripiprazole group. The log-rank test gives p-value .0391.

3.1.8.2 All-Cause Analysis

There are 36 relapsed in placebo and 19 relapsed in aripiprazole, and 28 completers in placebo and 39 completers in aripiprazole groups, respectively. Table 3.1.8.2.1 presents the withdrawals information for other subjects.

Table 3.1.8.2 Withdrawals Information

Reason	Placebo	Aripiprazole
AE	1	5
Withdrawal of subject consent/patient request	6	6
Lost to follow-up	1	1
Non-compliance	5	5
Other	6	4

If subjects in the above table are treated as relapsed, the log-rank test gives p-value .0640 using actual treatment. The log-rank test gives p-value .0991 using randomization code.

3.1.8.3 Protocol Deviation

There were 35 patients excluded from the Samples because of a labeling error. Table 3.1.8.3 presents reasons.

Table 3.1.8.3 Discontinuation Reasons for Patients Excluded Due to Labeling Error

Reason	Placebo	Aripiprazole
Withdrawal of subject consent/patient request	1	1
Lost to follow-up	1	1
Relapsed	5	2
Non-compliance	0	3
Other	8	13

The impact due to this exclusion is difficult to evaluate.

3.1.8.4 Analysis by Country

Table 3.1.8.4.1 indicates that aripiprazole has a smaller relapse rate in all three countries using actual treatment code.

Table 3.1.8.4.1 Relapse Rate by Country

Country	Placebo		Aripiprazole	
	N	Relapsed	N	Relapsed
Argentina	3	3 (100%)	4	1 (25%)
Mexico	16	7 (44%)	14	1 (7%)
USA	64	26 (41%)	59	17 (29%)

The log-rank test for USA gives p-value .1952, although the sample size is not powered for this subgroup. Notice that the relapse rate of aripiprazole in both Mexico and Argentina are lower than that in USA, and the relapse rate of placebo in both Mexico and Argentina are higher than that in USA. The relapse rate of aripiprazole in Mexico is extremely lower relative to both those in Argentina and USA. The log-rank test gives p-value .1125 after removing Mexico. The log-rank test gives p-value .0379 after removing Argentina.

Using Cox regression, p-values for country and interaction by treatment are not significant. There are 50 centers in the study. Centers in Mexico are largest. The next largest has 8 subjects so analysis based on center is not performed.

Table 3.1.8.4.2 presents relapse rates by two centers in Mexico.

Table 3.1.8.4.2 Relapse Rate by Center in Mexico

Center	Placebo		Aripiprazole	
	N	Relapsed	N	Relapsed
093	7	5 (71%)	6	0 (0%)
118	9	2 (22%)	8	1 (13%)

The log-rank test gives p-value .1043 after removing center 093 due to its relatively lower relapse rate. Baseline measures are balanced between two groups for center 093. Center 093 was suggested to DSI to have an inspection after filing meeting. If the inspection result indicates that the quality of operation in center 093 is poor, one needs to be very cautions when making final decision since we don't have much experience in Mexico.

There is one subject 00093 00533 who was randomized to Aripiprazole but actual treatment was placebo. If using randomization code, the subject was in Aripiprazole group. Table 3.1.8.4.3 presents relapse rates by Center 093 using the randomization code.

Table 3.1.8.4.3 Relapse Rate by Center in Mexico Using Randomization Code

Center	Placebo		Aripiprazole	
	N	Relapsed	N	Relapsed
093	6	4 (67%)	7	1 (14%)

3.2 Evaluation of Safety

See Clinical Review.

4. Findings in Special/Subgroup Populations

4.1 Gender, Race, and Age

Since the Study was not powered for subgroup analyses, analytical analysis is not performed. Table 4.1.1 indicates that aripiprazole has smaller relapse rate in both male and female groups.

Table 4.1.1 Relapse Rate by Gender

Gender	Placebo		Aripiprazole	
	N	Relapsed	N	Relapsed
Male	23	9 (39%)	30	7 (23%)
Female	60	27 (45%)	47	12 (26%)

Since majority subjects are white, no separate analysis on race is performed.

Table 4.1.2 indicates that aripiprazole has smaller relapse rate in both age groups (use median to divide age group because no subject in placebo group is older than 65).

Table 4.1.2 Relapse Rate by Age

Age	Placebo		Aripiprazole	
	N	Relapsed	N	Relapsed
≤ 40	43	17 (40%)	42	7 (17%)
> 40	40	19 (48%)	35	12 (34%)

4.2 Other Special/Subgroup Populations

There is no other subgroup analysis performed.

5. Summary and Conclusions

5.1 Statistical Issues and Collective Evidence

The primary analysis is log-rank test which gives p-value .0199 where there were 36 out of 83 (43%) relapsed in placebo, and 19 out of 77 (25%) relapsed in aripiprazole groups, respectively.

One issue is whether the study is robust because center 093 in Mexico, where there were 7 in placebo and 6 in aripiprazole groups, respectively, had 5 (71%) relapsed in placebo and 0 (0%) relapsed in aripiprazole groups, respectively. The primary analysis is not significant after removing this center.

5.2 Conclusions and Recommendations

The conclusion is that the primary analysis for the time from randomization to relapse during the maintenance phase is significant comparing aripiprazole and placebo in evaluating subjects with Bipolar I Disorder but one should consider whether the quality of operations in center 093 is high, which was suggested to be inspected by DSI, when making final decision.

Appears This Way
On Original

This is a representation of an electronic record that was signed electronically and this page is the manifestation of the electronic signature.

/s/

Kun He
11/9/04 10:32:24 AM
BIOMETRICS

Kun Jin
11/9/04 02:22:56 PM
BIOMETRICS

James Hung
11/9/04 02:32:42 PM
BIOMETRICS

CENTER FOR DRUG EVALUATION AND RESEARCH

APPLICATION NUMBER:

21-436/S-005 & S-008 & 21-713/S-003

ADMINISTRATIVE and CORRESPONDENCE DOCUMENTS

Department of Health and Human Services<br>Food and Drug Administration<br>**PATENT INFORMATION SUBMITTED WITH THE FILING OF AN NDA, AMENDMENT, OR SUPPLEMENT**<br>***For Each Patent That Claims a Drug Substance (Active Ingredient), Drug Product (Formulation and Composition) and/or Method of Use***	Form Approved: OMB No. 0910-0513<br>Expiration Date: 07/31/06<br>*See OMB Statement on Page 3.*<br>NDA NUMBER<br>21-436<br>NAME OF APPLICANT / NDA HOLDER<br>Otsuka Pharmaceutical Co., Ltd.

The following is provided in accordance with Section 505(b) and (c) of the Federal Food, Drug, and Cosmetic Act.

TRADE NAME (OR PROPOSED TRADE NAME)<br>ABILIFY	
ACTIVE INGREDIENT(S)<br>ARIPIPRAZOLE	STRENGTH(S)<br>2, 5, 10, 15, 20 & 30mg
DOSAGE FORM<br>Tablet	

This patent declaration form is required to be submitted to the Food and Drug Administration (FDA) with an NDA application, amendment, or supplement as required by 21 CFR 314.53 at the address provided in 21 CFR 314.53(d)(4).
Within thirty (30) days after approval of an NDA or supplement, or within thirty (30) days of issuance of a new patent, a new patent declaration must be submitted pursuant to 21 CFR 314.53(c)(2)(ii) with all of the required information based on the approved NDA or supplement. The information submitted in the declaration form submitted upon or after approval will be the only information relied upon by FDA for listing a patent in the Orange Book.

For hand-written or typewriter versions (only) of this report: If additional space is required for any narrative answer (i.e., one that does not require a "Yes" or "No" response), please attach an additional page referencing the question number.

FDA will not list patent information if you file an incomplete patent declaration or the patent declaration indicates the patent is not eligible for listing.

For each patent submitted for the pending NDA, amendment, or supplement referenced above, you must submit all the information described below. If you are not submitting any patents for this pending NDA, amendment, or supplement, complete above section and sections 5 and 6.

1. GENERAL

a. United States Patent Number	b. Issue Date of Patent	c. Expiration Date of Patent
5,006,528	4/9/1991	10/20/2009

d. Name of Patent Owner	Address *(of Patent Owner)*	
Otsuka Pharmaceutical Co., Ltd.	2-9 Kanda Tsukasa-cho, Chiyoda-ku	
	City/State<br>Tokyo, Japan	
	ZIP Code<br>101-8535	FAX Number *(if available)*
	Telephone Number<br>81-3-3292-0021	E-Mail Address *(if available)*
e. Name of agent or representative who resides or maintains a place of business within the United States authorized to receive notice of patent certification under section 505(b)(3) and (j)(2)(B) of the Federal Food, Drug, and Cosmetic Act and 21 CFR 314.52 and 314.95 (if patent owner or NDA applicant/holder does not reside or have a place of business within the United States)	Address *(of agent or representative named in 1.e.)*<br>Otsuka America Pharmaceutical, Inc.	
	City/State<br>2440 Research Boulevard<br>Rockville, MD	
	ZIP Code<br>20850	FAX Number *(if available)*<br>(301) 212-8643
	Telephone Number<br>(240) 683-3049	E-Mail Address *(if available)*<br>sheilac@otsuka.com

f. Is the patent referenced above a patent that has been submitted previously for the approved NDA or supplement referenced above? ☒ Yes ☐ No

g. If the patent referenced above has been submitted previously for listing, is the expiration date a new expiration date? ☐ Yes ☒ No

For the patent referenced above, provide the following information on the drug substance, drug product and/or method of use that is the subject of the pending NDA, amendment, or supplement.

2. Drug Substance (Active Ingredient)

2.1	Does the patent claim the drug substance that is the active ingredient in the drug product described in the pending NDA, amendment, or supplement?	☒ Yes	☐ No
2.2	Does the patent claim a drug substance that is a different polymorph of the active ingredient described in the pending NDA, amendment, or supplement?	☐ Yes	☒ No
2.3	If the answer to question **2.2** is "Yes," do you certify that, as of the date of this declaration, you have test data demonstrating that a drug product containing the polymorph will perform the same as the drug product described in the NDA? The type of test data required is described at 21 CFR 314.53(b).	☐ Yes	☐ No
2.4	Specify the polymorphic form(s) claimed by the patent for which you have the test results described in **2.3**.		
2.5	Does the patent claim only a metabolite of the active ingredient pending in the NDA or supplement? (Complete the information in section 4 below if the patent claims a pending method of using the pending drug product to administer the metabolite.)	☐ Yes	☒ No
2.6	Does the patent claim only an intermediate?	☐ Yes	☒ No
2.7	If the patent referenced in 2.1 is a product-by-process patent, is the product claimed in the patent novel? (An answer is required only if the patent is a product-by-process patent.)	☐ Yes	☐ No

3. Drug Product (Composition/Formulation)

3.1	Does the patent claim the drug product, as defined in 21 CFR 314.3, in the pending NDA, amendment, or supplement?	☒ Yes	☐ No
3.2	Does the patent claim only an intermediate?	☐ Yes	☒ No
3.3	If the patent referenced in 3.1 is a product-by-process patent, is the product claimed in the patent novel? (An answer is required only if the patent is a product-by-process patent.)	☐ Yes	☐ No

4. Method of Use

Sponsors must submit the information in section 4 separately for each patent claim claiming a method of using the pending drug product for which approval is being sought. For each method of use claim referenced, provide the following information:

4.1	Does the patent claim one or more methods of use for which approval is being sought in the pending NDA, amendment, or supplement?		☐ Yes	☒ No
4.2	Patent Claim Number *(as listed in the patent)*	Does the patent claim referenced in **4.2** claim a pending method of use for which approval is being sought in the pending NDA, amendment, or supplement?	☐ Yes	☐ No
4.2a	If the answer to **4.2** is "Yes," identify with specificity the use with reference to the proposed labeling for the drug product.	Use: *(Submit indication or method of use information as identified specifically in the approved labeling.)*		

5. No Relevant Patents

For this pending NDA, amendment, or supplement, there are no relevant patents that claim the drug substance (active ingredient), drug product (formulation or composition) or method(s) of use, for which the applicant is seeking approval and with respect to which a claim of patent infringement could reasonably be asserted if a person not licensed by the owner of the patent engaged in the manufacture, use, or sale of the drug product.	☐ Yes

6. Declaration Certification

6.1 ***The undersigned declares that this is an accurate and complete submission of patent information for the NDA, amendment, or supplement pending under section 505 of the Federal Food, Drug, and Cosmetic Act. This time-sensitive patent information is submitted pursuant to 21 CFR 314.53. I attest that I am familiar with 21 CFR 314.53 and this submission complies with the requirements of the regulation. I verify under penalty of perjury that the foregoing is true and correct.***

Warning: A willfully and knowingly false statement is a criminal offense under 18 U.S.C. 1001.

6.2 Authorized Signature of NDA Applicant/Holder or Patent Owner *(Attorney, Agent, Representative or other Authorized Official) (Provide Information below)*	Date Signed
Sheila A. Cleary	12/10/2003

NOTE: Only an NDA applicant/holder may submit this declaration directly to the FDA. A patent owner who is not the NDA applicant/holder is authorized to sign the declaration but may not submit it directly to FDA. 21 CFR 314.53(c)(4) and (d)(4).

Check applicable box and provide information below.

☐ NDA Applicant/Holder	☒ NDA Applicant's/Holder's Attorney, Agent (Representative) or other Authorized Official
☐ Patent Owner	☐ Patent Owner's Attorney, Agent (Representative) or Other Authorized Official
Name<br>Sheila A. Cleary	
Address<br>Otsuka America Pharmaceutical, Inc.	City/State<br>Rockville, MD
ZIP Code<br>20850	Telephone Number<br>(240) 683-3049
FAX Number *(if available)*<br>(301) 212-8643	E-Mail Address *(if available)*<br>sheilac@otsuka.com

The public reporting burden for this collection of information has been estimated to average 9 hours per response, including the time for reviewing instructions, searching existing data sources, gathering and maintaining the data needed, and completing and reviewing the collection of information. Send comments regarding this burden estimate or any other aspect of this collection of information, including suggestions for reducing this burden to:

Food and Drug Administration
CDER (HFD-007)
5600 Fishers Lane
Rockville, MD 20857

An agency may not conduct or sponsor, and a person is not required to respond to, a collection of information unless it displays a currently valid OMB control number.

Department of Health and Human Services<br>Food and Drug Administration<br>**PATENT INFORMATION SUBMITTED WITH THE FILING OF AN NDA, AMENDMENT, OR SUPPLEMENT**<br>***For Each Patent That Claims a Drug Substance (Active Ingredient), Drug Product (Formulation and Composition) and/or Method of Use***	Form Approved: OMB No. 0910-0513<br>Expiration Date: 07/31/06<br>*See OMB Statement on Page 3.*<br>NDA NUMBER<br>21-436<br>NAME OF APPLICANT / NDA HOLDER<br>Otsuka Pharmaceutical Co., Ltd.

The following is provided in accordance with Section 505(b) and (c) of the Federal Food, Drug, and Cosmetic Act.

TRADE NAME (OR PROPOSED TRADE NAME)<br>ABILIFY	
ACTIVE INGREDIENT(S)<br>ARIPIPRAZOLE	STRENGTH(S)<br>2, 5, 10, 15, 20 & 30mg
DOSAGE FORM<br>Tablet	

This patent declaration form is required to be submitted to the Food and Drug Administration (FDA) with an NDA application, amendment, or supplement as required by 21 CFR 314.53 at the address provided in 21 CFR 314.53(d)(4).
Within thirty (30) days after approval of an NDA or supplement, or within thirty (30) days of issuance of a new patent, a new patent declaration must be submitted pursuant to 21 CFR 314.53(c)(2)(ii) with all of the required information based on the approved NDA or supplement. The information submitted in the declaration form submitted upon or after approval will be the only information relied upon by FDA for listing a patent in the Orange Book.

For hand-written or typewriter versions (only) of this report: If additional space is required for any narrative answer (i.e., one that does not require a "Yes" or "No" response), please attach an additional page referencing the question number.

FDA will not list patent information if you file an incomplete patent declaration or the patent declaration indicates the patent is not eligible for listing.

For each patent submitted for the pending NDA, amendment, or supplement referenced above, you must submit all the information described below. If you are not submitting any patents for this pending NDA, amendment, or supplement, complete above section and sections 5 and 6.

1. GENERAL

a. United States Patent Number	b. Issue Date of Patent	c. Expiration Date of Patent
4,734,416	3/29/1988	3/29/2005
d. Name of Patent Owner<br>Otsuka Pharmaceutical Co., Ltd.	Address *(of Patent Owner)*<br>2-9 Kanda Tsukasa-cho, Chiyoda-ku	
	City/State<br>Tokyo, Japan	
	ZIP Code<br>101-8535	FAX Number *(if available)*
	Telephone Number<br>81-3-3292-0021	E-Mail Address *(if available)*
e. Name of agent or representative who resides or maintains a place of business within the United States authorized to receive notice of patent certification under section 505(b)(3) and (j)(2)(B) of the Federal Food, Drug, and Cosmetic Act and 21 CFR 314.52 and 314.95 (if patent owner or NDA applicant/holder does not reside or have a place of business within the United States)	Address *(of agent or representative named in 1.e.)*<br>Otsuka America Pharmaceutical, Inc.	
	City/State<br>2440 Research Boulevard<br>Rockville, MD	
	ZIP Code<br>20850	FAX Number *(if available)*<br>(301) 212-8643
	Telephone Number<br>(240) 683-3049	E-Mail Address *(if available)*<br>sheilac@otsuka.com

f. Is the patent referenced above a patent that has been submitted previously for the approved NDA or supplement referenced above?	☒ Yes	☐ No
g. If the patent referenced above has been submitted previously for listing, is the expiration date a new expiration date?	☐ Yes	☒ No

PSC Media Arts (301) 443-1090 EF

Approved 1.0 Item 13 patinfo.pdf

For the patent referenced above, provide the following information on the drug substance, drug product and/or method of use that is the subject of the pending NDA, amendment, or supplement.

2. Drug Substance (Active Ingredient)

**2.1**	Does the patent claim the drug substance that is the active ingredient in the drug product described in the pending NDA, amendment, or supplement?	☒ Yes	☐ No
**2.2**	Does the patent claim a drug substance that is a different polymorph of the active ingredient described in the pending NDA, amendment, or supplement?	☐ Yes	☒ No
**2.3**	If the answer to question **2.2** is "Yes," do you certify that, as of the date of this declaration, you have test data demonstrating that a drug product containing the polymorph will perform the same as the drug product described in the NDA? The type of test data required is described at 21 CFR 314.53(b).	☐ Yes	☐ No
**2.4**	Specify the polymorphic form(s) claimed by the patent for which you have the test results described in **2.3**.		
2.5	Does the patent claim only a metabolite of the active ingredient pending in the NDA or supplement? (Complete the information in section 4 below if the patent claims a pending method of using the pending drug product to administer the metabolite.)	☐ Yes	☒ No
**2.6**	Does the patent claim only an intermediate?	☐ Yes	☒ No
**2.7**	If the patent referenced in **2.1** is a product-by-process patent, is the product claimed in the patent novel? (An answer is required only if the patent is a product-by-process patent.)	☐ Yes	☐ No

3. Drug Product (Composition/Formulation)

3.1	Does the patent claim the drug product, as defined in 21 CFR 314.3, in the pending NDA, amendment, or supplement?	☒ Yes	☐ No
3.2	Does the patent claim only an intermediate?	☐ Yes	☒ No
3.3	If the patent referenced in 3.1 is a product-by-process patent, is the product claimed in the patent novel? (An answer is required only if the patent is a product-by-process patent.)	☐ Yes	☐ No

4. Method of Use

Sponsors must submit the information in section 4 separately for each patent claim claiming a method of using the pending drug product for which approval is being sought. For each method of use claim referenced, provide the following information:

**4.1**	Does the patent claim one or more methods of use for which approval is being sought in the pending NDA, amendment, or supplement?	☐ Yes	☒ No
**4.2**	Patent Claim Number *(as listed in the patent)* — Does the patent claim referenced in **4.2** claim a pending method of use for which approval is being sought in the pending NDA, amendment, or supplement?	☐ Yes	☐ No
**4.2a**	If the answer to **4.2** is "Yes," identify with specificity the use with reference to the proposed labeling for the drug product.	Use: *(Submit indication or method of use information as identified specifically in the approved labeling.)*	

5. No Relevant Patents

For this pending NDA, amendment, or supplement, there are no relevant patents that claim the drug substance (active ingredient), drug product (formulation or composition) or method(s) of use, for which the applicant is seeking approval and with respect to which a claim of patent infringement could reasonably be asserted if a person not licensed by the owner of the patent engaged in the manufacture, use, or sale of the drug product.	☐ Yes

**6. Declaration Certification**	
6.1 ***The undersigned declares that this is an accurate and complete submission of patent information for the NDA, amendment, or supplement pending under section 505 of the Federal Food, Drug, and Cosmetic Act. This time-sensitive patent information is submitted pursuant to 21 CFR 314.53. I attest that I am familiar with 21 CFR 314.53 and this submission complies with the requirements of the regulation. I verify under penalty of perjury that the foregoing is true and correct.***<br>***Warning: A willfully and knowingly false statement is a criminal offense under 18 U.S.C. 1001.***	
6.2 Authorized Signature of NDA Applicant/Holder or Patent Owner *(Attorney, Agent, Representative or other Authorized Official) (Provide Information below)*<br>Sheila A. Cleary	Date Signed<br>12/10/2003
**NOTE: Only an NDA applicant/holder may submit this declaration directly to the FDA. A patent owner who is not the NDA applicant/holder is authorized to sign the declaration but may not submit it directly to FDA. 21 CFR 314.53(c)(4) and (d)(4).**	
**Check applicable box and provide information below.**	
☐ NDA Applicant/Holder	☒ NDA Applicant's/Holder's Attorney, Agent (Representative) or other Authorized Official
☐ Patent Owner	☐ Patent Owner's Attorney, Agent (Representative) or Other Authorized Official
Name<br>Sheila A. Cleary	
Address<br>Otsuka America Pharmaceutical, Inc.	City/State<br>Rockville, MD
ZIP Code<br>20850	Telephone Number<br>(240) 683-3049
FAX Number *(if available)*<br>(301) 212-8643	E-Mail Address *(if available)*<br>sheilac@otsuka.com

The public reporting burden for this collection of information has been estimated to average 9 hours per response, including the time for reviewing instructions, searching existing data sources, gathering and maintaining the data needed, and completing and reviewing the collection of information. Send comments regarding this burden estimate or any other aspect of this collection of information, including suggestions for reducing this burden to:

Food and Drug Administration
CDER (HFD-007)
5600 Fishers Lane
Rockville, MD 20857

An agency may not conduct or sponsor, and a person is not required to respond to, a collection of information unless it displays a currently valid OMB control number.

EXCLUSIVITY SUMMARY FOR NDA # __21436_____________ SUPPL #__005__

Trade Name _ABILIFY_______________ Generic Name _aripiprazole___

Applicant Name _Otsuka___________ HFD # _120___________________

Approval Date If Known _see electronic signature page_

CLAIM: The use of aripiprazole as maintenance therapy in Bipolar I Disorder.

PART I IS AN EXCLUSIVITY DETERMINATION NEEDED?

1. An exclusivity determination will be made for all original applications, and all efficacy supplements. Complete PARTS II and III of this Exclusivity Summary only if you answer "yes" to one or more of the following question about the submission.

a) Is it a 505(b)(1), 505(b)(2) or efficacy supplement?
YES /_✓__/ NO /___/

If yes, what type? Specify 505(b)(1), 505(b)(2), SE1, SE2, SE3,SE4, SE5, SE6, SE7, SE8

__SE1_______________

c) Did it require the review of clinical data other than to support a safety claim or change in labeling related to safety? (If it required review only of bioavailability or bioequivalence data, answer "no.")

YES /_✓__/ NO /___/

If your answer is "no" because you believe the study is a bioavailability study and, therefore, not eligible for exclusivity, EXPLAIN why it is a bioavailability study, including your reasons for disagreeing with any arguments made by the applicant that the study was not simply a bioavailability study.

____

____

If it is a supplement requiring the review of clinical data but it is not an effectiveness supplement, describe the change or claim that is supported by the clinical data:

____

____

d) Did the applicant request exclusivity?

YES /_✓__/ NO /___/

If the answer to (d) is "yes," how many years of exclusivity did the applicant request?

__Three (3)________

e) Has pediatric exclusivity been granted for this Active Moiety?

YES /___/ NO /_✓__/

<u>If the answer to the above question in YES,</u> is this approval a result of the studies submitted in response to the Pediatric Written Request?

____

IF YOU HAVE ANSWERED "NO" TO <u>ALL</u> OF THE ABOVE QUESTIONS, GO DIRECTLY TO THE SIGNATURE BLOCKS AT THE END OF THIS DOCUMENT.

2. Is this drug product or indication a DESI upgrade?

YES /___/ NO /_✓__/

IF THE ANSWER TO QUESTION 2 IS "YES," GO DIRECTLY TO THE SIGNATURE BLOCKS ON PAGE 8 (even if a study was required for the upgrade).

PART II FIVE-YEAR EXCLUSIVITY FOR NEW CHEMICAL ENTITIES

(Answer either #1 or #2 as appropriate)

1. <u>Single active ingredient product</u>.

Has FDA previously approved under section 505 of the Act any drug product containing the same active moiety as the drug under consideration? Answer "yes" if the active moiety (including other esterified forms, salts, complexes, chelates or clathrates) has been previously approved, but this particular form of the active moiety, e.g., this particular ester or salt (including salts with hydrogen or coordination bonding) or other non-covalent derivative (such as a complex, chelate, or clathrate) has not been approved. Answer "no" if the compound requires metabolic conversion (other than deesterification of an esterified form of the drug) to produce an already approved active moiety.

YES /_✓__/ NO /___/

If "yes," identify the approved drug product(s) containing the active moiety, and, if known, the NDA #(s).

NDA# _21-436______________ __Abilify Tablets___

NDA# ______________ ______________

NDA# ______________ ______________

2. Combination product.

If the product contains more than one active moiety(as defined in Part II, #1), has FDA previously approved an application under section 505 containing any one of the active moieties in the drug product? If, for example, the combination contains one never-before-approved active moiety and one previously approved active moiety, answer "yes." (An active moiety that is marketed under an OTC monograph, but that was never approved under an NDA, is considered not previously approved.) NOT APPLICABLE

YES /___/ NO /___/

If "yes," identify the approved drug product(s) containing the active moiety, and, if known, the NDA #(s).

NDA# __________ ______________

NDA# __________ ______________

NDA# __________ ______________

IF THE ANSWER TO QUESTION 1 OR 2 UNDER PART II IS "NO," GO DIRECTLY TO THE SIGNATURE BLOCKS ON PAGE 8. (Caution: The questions in part II of the summary should only be answered "NO" for original approvals of new molecular entities.) IF "YES" GO TO PART III.

PART III THREE-YEAR EXCLUSIVITY FOR NDA'S AND SUPPLEMENTS

To qualify for three years of exclusivity, an application or supplement must contain "reports of new clinical investigations (other than bioavailability studies) essential to the approval of the application and conducted or sponsored by the applicant." This section should be completed only if the answer to PART II, Question 1 or 2 was "yes."

1. Does the application contain reports of clinical investigations? (The Agency interprets "clinical investigations" to mean investigations conducted on humans other than bioavailability studies.) If the application contains clinical investigations only by virtue of a right of reference to clinical investigations in another application, answer "yes," then skip to question 3(a). If the answer to 3(a) is "yes" for any investigation referred to in another application, do not complete remainder of summary for that investigation.

YES /_✓__/ NO /___/

IF "NO," GO DIRECTLY TO THE SIGNATURE BLOCKS ON PAGE 8.

2. A clinical investigation is "essential to the approval" if the Agency could not have approved the application or supplement without relying on that investigation. Thus, the investigation is not essential to the approval if 1) no clinical investigation is necessary to support the supplement or application in light of previously approved applications (i.e., information other than clinical trials, such as bioavailability data, would be sufficient to provide a basis for approval as an ANDA or 505(b)(2) application because of what is already known about a previously approved product), or 2) there are published reports of studies (other than those conducted or sponsored by the applicant) or other publicly available data that independently would have been sufficient to support approval of the application, without reference to the clinical investigation submitted in the application.

(a) In light of previously approved applications, is a clinical investigation (either conducted by the applicant or available from some other source, including the published literature) necessary to support approval of the application or supplement?

YES /_✓__/ NO /___/

If "no," state the basis for your conclusion that a clinical trial is not necessary for approval AND GO DIRECTLY TO SIGNATURE BLOCK ON PAGE 8:

____

(b) Did the applicant submit a list of published studies relevant to the safety and effectiveness of this drug product and a statement that the publicly available data would not independently support approval of the application?

YES /_____/ NO /__✓__/

(1) If the answer to 2(b) is "yes," do you personally know of any reason to disagree with the applicant's conclusion? If not applicable, answer NO.

YES /___/ NO /_✓__/

If yes, explain:

---

(2) If the answer to 2(b) is "no," are you aware of published studies not conducted or sponsored by the applicant or other publicly available data that could independently demonstrate the safety and effectiveness of this drug product?

YES /___/ NO /_✓__/

If yes, explain:

(c) If the answers to (b)(1) and (b)(2) were both "no," identify the clinical investigations submitted in the application that are essential to the approval:

__CN138010 _(Investigation 1: note only one study was required for this indication)____________________

Studies comparing two products with the same ingredient(s) are considered to be bioavailability studies for the purpose of this section.

3. In addition to being essential, investigations must be "new" to support exclusivity. The agency interprets "new clinical investigation" to mean an investigation that 1) has not been relied on by the agency to demonstrate the effectiveness of a previously approved drug for any indication and 2) does not duplicate the results of another investigation that was relied on by the agency to demonstrate the effectiveness of a previously approved drug product, i.e., does not redemonstrate something the agency considers to have been demonstrated in an already approved application.

a) For each investigation identified as "essential to the approval," has the investigation been relied on by the agency to demonstrate the effectiveness of a previously approved drug product? (If the investigation was relied on only to support the safety of a previously approved drug, answer "no.")

Investigation #1 YES /___/ NO /_✓__/

Investigation #2 YES /___/ NO /___/

If you have answered "yes" for one or more investigations, identify each such investigation and the NDA in which each was relied upon:

_______________________ _________________________

_______________________ _________________________

b) For each investigation identified as "essential to the approval", does the investigation duplicate the results of another investigation that was relied on by the agency to support the effectiveness of a previously approved drug product?

Investigation #1 YES /___/ NO /_✓__/

Investigation #2 YES /___/ NO /___/

If you have answered "yes" for one or more investigation, identify the NDA in which a similar investigation was relied on:

_______________________ _________________________

_______________________ _________________________

c) If the answers to 3(a) and 3(b) are no, identify each "new" investigation in the application or supplement that is essential to the approval (i.e., the investigations listed in #2(c), less any that are not "new"):

_ CN138010 (Investigation 1) _________________________

____

4. To be eligible for exclusivity, a new investigation that is

essential to approval must also have been conducted or sponsored by the applicant. An investigation was "conducted or sponsored by" the applicant if, before or during the conduct of the investigation, 1) the applicant was the sponsor of the IND named in the form FDA 1571 filed with the Agency, or 2) the applicant (or its predecessor in interest) provided substantial support for the study. Ordinarily, substantial support will mean providing 50 percent or more of the cost of the study.

a) For each investigation identified in response to question 3(c): if the investigation was carried out under an IND, was the applicant identified on the FDA 1571 as the sponsor?

Investigation #1 !

IND # _42776_____ YES /_✓__/ ! NO /___/ Explain: ________

Investigation #2 !

IND # __________ YES /___/ ! NO /___/ Explain: ________

(b) For each investigation not carried out under an IND or for which the applicant was not identified as the sponsor, did the applicant certify that it or the applicant's predecessor in interest provided substantial support for the study?
NOT APPLICABLE

Investigation #1 !

YES /___/ Explain _____ ! NO /___/ Explain _________

Investigation #2 !

YES /___/ Explain _____ ! NO /___/ Explain _________

(c) Notwithstanding an answer of "yes" to (a) or (b), are there other reasons to believe that the applicant should not be credited with having "conducted or sponsored" the study?

(Purchased studies may not be used as the basis for exclusivity. However, if all rights to the drug are purchased (not just studies on the drug), the applicant may be considered to have sponsored or conducted the studies sponsored or conducted by its predecessor in interest.)

YES /___/ NO /_✓__/

If yes, explain: ____

____

See electronic signature page
Signature Date
Title:

See electronic signature page
Signature of Office/ Date
Division Director

Form OGD-011347 Revised 05/10/2004

cc:
Archival NDA
HFD-   /Division File
HFD-   /RPM
HFD-610/Mary Ann Holovac
HFD-104/PEDS/T.Crescenzi

This is a representation of an electronic record that was signed electronically and this page is the manifestation of the electronic signature.

/s/

Doris Bates
2/14/2006 04:31:56 PM

Thomas Laughren
2/14/2006 05:22:25 PM

PEDIATRIC PAGE

NDA/BLA # : 21-436 / 21-713 Supplement Type (e.g. SE5): 21436: SE1 21713: SLR Supplement Number: SE1 005 [ ] **b(4)**

Stamp Date: 30-JAN-2004 Action Date: 4-MAR-2005 (4-JAN-2005 Resubmission)

HFD 120 Trade and generic names/dosage form: ABILIFY (aripiprazole)

Applicant: Otsuka Pharmaceuticals, Ltd. Therapeutic Class: Antimanic

Indication(s) previously approved: schizophrenia, longer-term treatment of schizophrenia,m onotherapy in treatment of acute manic or mixed episodes associated with bipolar disorder.

Each approved indication must have pediatric studies: Completed, Deferred, and/or Waived.

Number of indications for this application(s): 1

Indication #1: maintenance therapy in Bipolar I Disorder

Is there a full waiver for this indication (check one)?

☑ Yes for N 21713: Please proceed to Section A.

☑ No for N 21436: Please check all that apply: ✓ Partial Waiver ✓ Deferred ___ Completed
NOTE: More than one may apply
Please proceed to Section B, Section C, and/or Section D and complete as necessary.

Section A: Fully Waived Studies

Reason(s) for full waiver: NDA 21713 only:

- ☐ Products in this class for this indication have been studied/labeled for pediatric population
- ☐ Disease/condition does not exist in children
- ☐ Too few children with disease to study
- ☐ There are safety concerns
- ☑ Other: studies under NDA 21436 will address issues germane to NDA 21713.

If studies are fully waived, then pediatric information is complete for this indication. If there is another indication, please see Attachment A. Otherwise, this Pediatric Page is complete and should be entered into DFS.

Section B: Partially Waived Studies

Age/weight range being partially waived NDA 21-436 ONLY:

Min___	kg___	mo.___	yr. 0	Tanner Stage___
Max___	kg___	mo.___	yr. 10	Tanner Stage___

Reason(s) for partial waiver:

- ☐ Products in this class for this indication have been studied/labeled for pediatric population
- ☐ Disease/condition does not exist in children
- ☐ Too few children with disease to study
- ☐ There are safety concerns
- ☐ Adult studies ready for approval
- ☐ Formulation needed
- ☑ Other: Disease/condition not known to exist in this age group

If studies are deferred, proceed to Section C. If studies are completed, proceed to Section D. Otherwise, this Pediatric Page is complete and should be entered into DFS.

Section C: Deferred Studies

Age/weight range being deferred: NDA 21-436 ONLY

Min____ kg____ mo.____ yr._10____ Tanner Stage____
Max____ kg____ mo.____ yr._17____ Tanner Stage____

Reason(s) for deferral:

- ☐ **Products in this class for this indication have been studied/labeled for pediatric population**
- ☐ **Disease/condition does not exist in children**
- ☐ **Too few children with disease to study**
- ☐ **There are safety concerns**
- ☑ **Adult studies ready for approval**
- ☐ **Formulation needed**

Other:____________________

Date studies are due (mm/dd/yy): _April 1, 2009______

If studies are completed, proceed to Section D. Otherwise, this Pediatric Page is complete and should be entered into DFS.

Section D: Completed Studies

Age/weight range of completed studies:

Min____ kg____ mo.____ yr.____ Tanner Stage____
Max____ kg____ mo.____ yr.____ Tanner Stage____

Comments:

If there are additional indications, please proceed to Attachment A. Otherwise, this Pediatric Page is complete and should be entered into DFS.

This page was completed by:

{See appended electronic signature page}
_*Doris J. Bates, Ph.D.*______________
Regulatory Project Manager

cc: NDA
HFD-960/ Grace Carmouze
(revised 12-22-03)

FOR QUESTIONS ON COMPLETING THIS FORM CONTACT THE DIVISION OF PEDIATRIC DRUG DEVELOPMENT, HFD-960, 301-594-7337.

This is a representation of an electronic record that was signed electronically and this page is the manifestation of the electronic signature.

/s/

Doris Bates
3/1/05 02:00:01 PM
Approval anticipated on date this form signed. Note that the oral solution submission is a labeling supplement only. Peds requirements re the solution are waived because the tablets studies will cover them.

REQUEST FOR DEFERRAL OF SUBMISSION OF DATA ASSESSING THE SAFETY AND EFFICACY OF ARIPIPRAZOLE IN PEDIATRIC PATIENTS WITH BIPOLAR I DISORDER

We are hereby requesting a deferral of the requirement to provide data assessing the safety and efficacy of aripiprazole in pediatric patients with mania associated with Bipolar I Disorder in this application. None of the adequate and well-controlled studies in patients with bipolar mania in this application have included patients less than 18 years of age and no safety and efficacy studies in pediatric patients with bipolar illness have been initiated as yet.

As discussed during the November 13, 2003 meeting that was held to reach concurrence on the Abilify Pediatric Exclusivity program, we intend to initiate studies with Abilify in pediatric patients with acute bipolar mania in 2004, in response to the Division's Written Request of February 11, 2003 for this indication.

Therefore, in accordance with 21CFR314.55(b), we are requesting a deferral of the requirement to provide safety and efficacy data in pediatric patients with acute mania in this SNDA.

**Appears This Way
On Original**

PEDIATRIC PAGE

NDA/BLA # : 21-436 **Supplement Type (e.g. SE5): SE1** **Supplement Number: 005**

Stamp Date: 30-JAN-2004 **Action Date: 30-NOV-2004**

HFD 120 **Trade and generic names/dosage form: ABILIFY (aripiprazole)**

Applicant: Otsuka Pharmaceuticals, Ltd. **Therapeutic Class: Antimanic**

Indication(s) previously approved: schizophrenia, longer-term treatment of schizophrenia, monotherapy in treatment of acute manic or mixed episodes associated with bipolar disorder.

Each approved indication must have pediatric studies: Completed, Deferred, and/or Waived.

Number of indications for this application(s): 1

Indication #1: longer term monotherapy in treatment of acute manic or mixed episodes associated with bipolar disorder

Is there a full waiver for this indication (check one)?

- ☐ Yes: Please proceed to Section A.
- ☑ No: Please check all that apply: ✓ Partial Waiver ✓ Deferred ____ Completed
  NOTE: More than one may apply
  Please proceed to Section B, Section C, and/or Section D and complete as necessary.

Section A: Fully Waived Studies

Reason(s) for full waiver:

- ☐ Products in this class for this indication have been studied/labeled for pediatric population
- ☐ Disease/condition does not exist in children
- ☐ Too few children with disease to study
- ☐ There are safety concerns
- ☐ Other: ____________________

If studies are fully waived, then pediatric information is complete for this indication. If there is another indication, please see Attachment A. Otherwise, this Pediatric Page is complete and should be entered into DFS.

Section B: Partially Waived Studies

Age/weight range being partially waived:

Min____	kg____	mo.____	yr. 0	Tanner Stage____
Max____	kg____	mo.____	yr. 10	Tanner Stage____

Reason(s) for partial waiver:

- ☐ Products in this class for this indication have been studied/labeled for pediatric population
- ☐ Disease/condition does not exist in children
- ☐ Too few children with disease to study
- ☐ There are safety concerns
- ☐ Adult studies ready for approval
- ☐ Formulation needed
- ☑ Other: Disease/condition not known to exist in this age group

If studies are deferred, proceed to Section C. If studies are completed, proceed to Section D. Otherwise, this Pediatric Page is complete and should be entered into DFS.

Section C: Deferred Studies

Age/weight range being deferred:

**Min** ____	**kg**____	**mo.**____	**yr.** 10	**Tanner Stage**____
**Max** ____	**kg**____	**mo.**____	**yr.** 17	**Tanner Stage**____

Reason(s) for deferral:

- ☐ **Products in this class for this indication have been studied/labeled for pediatric population**
- ☐ **Disease/condition does not exist in children**
- ☐ **Too few children with disease to study**
- ☐ **There are safety concerns**
- ☑ **Adult studies ready for approval**
- ☐ **Formulation needed**

Other:______________________________

Date studies are due (mm/dd/yy): To be determined when applicant responds to AE letter

If studies are completed, proceed to Section D. Otherwise, this Pediatric Page is complete and should be entered into DFS.

Section D: Completed Studies

Age/weight range of completed studies:

**Min** ____	**kg**____	**mo.**____	**yr.**____	**Tanner Stage**____
**Max** ____	**kg**____	**mo.**____	**yr.**____	**Tanner Stage**____

Comments:

If there are additional indications, please proceed to Attachment A. Otherwise, this Pediatric Page is complete and should be entered into DFS.

This page was completed by:

{See appended electronic signature page}
_*Doris J. Bates, Ph.D.*_______________
Regulatory Project Manager

cc: NDA
HFD-960/ Grace Carmouze
(revised 12-22-03)

FOR QUESTIONS ON COMPLETING THIS FORM CONTACT THE DIVISION OF PEDIATRIC DRUG DEVELOPMENT, HFD-960, 301-594-7337.

NDA NO. 21-436

ABILIFY™ TABLETS

CERTIFICATION: DEBARRED PERSONS

As required by Section 306(k)(1) of the Federal Food, Drug and Cosmetics Act, Otsuka America Pharmaceutical, Inc. certifies that it has not used and will not use in any capacity the services of any person listed as debarred as of the Date of Debarment List Debarment List under Section 306 (a) or (b) of the Federal Food, Drug and Cosmetics Act in connection with this Application.

William H. Carson, M.D. (signature) — 12/2/03

William H. Carson, M.D.
Vice President, Global Product Development
Otsuka America Pharmaceutical, Inc.
100 Overlook Drive
Princeton, NJ 08540
609-452-2922

Certification Date

NDA NO. 21-436

ABILIFY™ TABLETS

CERTIFICATION: DEBARRED PERSONS

As required by Section 306(k)(1) of the Federal Food, Drug and Cosmetics Act, Bristol-Myers Squibb Company certifies that it has not used and will not use in any capacity the services of any person listed as debarred as of the Date of Debarment List Debarment List under Section 306 (a) or (b) of the Federal Food, Drug and Cosmetics Act in connection with this Application.

Susan H. Behling

December 10, 200

Susan H. Behling,
Director, Global Regulatory Science
Bristol-Myers Squibb Pharmaceutical Company
5 Research Parkway, Dept. 718
Signature 91 Building
Wallingford, CT 06492
(203) 677-3810

Certification Date

NDA/EFFICACY SUPPLEMENT ACTION PACKAGE CHECKLIST

NDA 21-436	Efficacy Supplement Type SE-1	Supplement Number 005	
Drug: Abilify (aripiprazole) Tablets		Applicant: Otsuka Pharmaceutical Company, Ltd.	
RPM: Doris J. Bates, Ph.D.		HFD-120	Phone # 301.594.2850

Application Type: (✓) 505(b)(1) ( ) 505(b)(2)<br>(This can be determined by consulting page 1 of the NDA Regulatory Filing Review for this application or Appendix A to this Action Package Checklist.)<br><br>**If this is a 505(b)(2) application, please review and confirm the information previously provided in Appendix B to the NDA Regulatory Filing Review. Please update any information (including patent certification information) that is no longer correct.**<br><br>( ) Confirmed and/or corrected	Listed drug(s) referred to in 505(b)(2) application (NDA #(s), Drug name(s)):

❖ Application Classifications:	
• Review priority	(✓) Standard ( ) Priority
• Chem class (NDAs only)	Not Applicable
• Other (e.g., orphan, OTC)	Not Applicable
❖ User Fee Goal Dates	March 4, 2005
❖ Special programs (indicate all that apply)	(✓) None<br>Subpart H<br>( ) 21 CFR 314.510 (accelerated approval)<br>( ) 21 CFR 314.520 (restricted distribution)<br>( ) Fast Track<br>( ) Rolling Review<br>( ) CMA Pilot 1<br>( ) CMA Pilot 2
❖ User Fee Information	
• User Fee	(✓) Paid UF ID number 4667
• User Fee waiver	( ) Small business<br>( ) Public health<br>( ) Barrier-to-Innovation<br>( ) Other (specify)
• User Fee exception	( ) Orphan designation<br>( ) No-fee 505(b)(2) (see NDA Regulatory Filing Review for instructions)<br>( ) Other (specify)
❖ Application Integrity Policy (AIP)	
• Applicant is on the AIP	( ) Yes (✓) No

• This application is on the AIP	( ) Yes (✓) No
• Exception for review (Center Director's memo)	Not Applicable
• OC clearance for approval	Not Applicable
❖ Debarment certification: verified that qualifying language (e.g., willingly, knowingly) was not used in certification & certifications from foreign applicants are cosigned by US agent.	(✓) Verified
❖ Patent	
• Information: Verify that form FDA-3542a was submitted for patents that claim the drug for which approval is sought.	Patent Information Submitted
• Patent certification [505(b)(2) applications]: Verify that a certification was submitted for each patent for the listed drug(s) in the Orange Book and identify the type of certification submitted for each patent.	Not Applicable 21 CFR 314.50(i)(1)(*i*)(A)<br>( ) Verified<br><br>21 CFR 314.50(i)(1)<br>( ) (ii) ( ) (iii)
• [505(b)(2) applications] If the application includes a paragraph III certification, it cannot be approved until the date that the patent to which the certification pertains expires (but may be tentatively approved if it is otherwise ready for approval).	Not Applicable
• [505(b)(2) applications] For each paragraph IV certification, verify that the applicant notified the NDA holder and patent owner(s) of its certification that the patent(s) is invalid, unenforceable, or will not be infringed (review documentation of notification by applicant and documentation of receipt of notice by patent owner and NDA holder). *(If the application does not include any paragraph IV certifications, mark "N/A" and skip to the next box below (Exclusivity)).*	Not Applicable<br>( ) N/A (no paragraph IV certification)<br>( ) Verified
• [505(b)(2) applications] For each paragraph IV certification, based on the questions below, determine whether a 30-month stay of approval is in effect due to patent infringement litigation.<br><br>Answer the following questions for each paragraph IV certification:	
(1) Have 45 days passed since the patent owner's receipt of the applicant's notice of certification?<br><br>(Note: The date that the patent owner received the applicant's notice of certification can be determined by checking the application. The applicant is required to amend its 505(b)(2) application to include documentation of this date (e.g., copy of return receipt or letter from recipient acknowledging its receipt of the notice) (see 21 CFR 314.52(e))).<br><br>*If "**Yes,**" skip to question (4) below. If "No," continue with question (2).*	( ) Yes ( ) No
(2) Has the patent owner (or NDA holder, if it is an exclusive patent licensee) submitted a written waiver of its right to file a legal action for patent infringement after receiving the applicant's notice of certification, as provided for by 21 CFR 314.107(f)(3)?<br><br>*If "**Yes,**" there is no stay of approval based on this certification. Analyze the next paragraph IV certification in the application, if any. If there are no other paragraph IV certifications, skip to the next box below (Exclusivity).*<br><br>*If "No," continue with question (3).*	( ) Yes ( ) No
(3) Has the patent owner, its representative, or the exclusive patent licensee filed a lawsuit for patent infringement against the applicant?	( ) Yes ( ) No

(Note: This can be determined by confirming whether the Division has received a written notice from the applicant (or the patent owner or its representative) stating that a legal action was filed within 45 days of receipt of its notice of certification. The applicant is required to notify the Division in writing whenever an action has been filed within this 45-day period (see 21 CFR 314.107(f)(2))).<br><br>*If "No," the patent owner (or NDA holder, if it is an exclusive patent licensee) has until the expiration of the 45-day period described in question (1) to waive its right to bring a patent infringement action or to bring such an action. After the 45-day period expires, continue with question (4) below.*	
(4) Did the patent owner (or NDA holder, if it is an exclusive patent licensee) submit a written waiver of its right to file a legal action for patent infringement within the 45-day period described in question (1), as provided for by 21 CFR 314.107(f)(3)?<br><br>*If "Yes," there is no stay of approval based on this certification. Analyze the next paragraph IV certification in the application, if any. If there are no other paragraph IV certifications, skip to the next box below (Exclusivity).*<br><br>*If "No," continue with question (5).*	() Yes () No
(5) Did the patent owner, its representative, or the exclusive patent licensee bring suit against the applicant for patent infringement within 45 days of the patent owner's receipt of the applicant's notice of certification?<br><br>(Note: This can be determined by confirming whether the Division has received a written notice from the applicant (or the patent owner or its representative) stating that a legal action was filed within 45 days of receipt of its notice of certification. The applicant is required to notify the Division in writing whenever an action has been filed within this 45-day period (see 21 CFR 314.107(f)(2)). If no written notice appears in the NDA file, confirm with the applicant whether a lawsuit was commenced within the 45-day period).<br><br>*If "No," there is no stay of approval based on this certification. Analyze the next paragraph IV certification in the application, if any. If there are no other paragraph IV certifications, skip to the next box below (Exclusivity).*<br><br>*If "Yes," a stay of approval may be in effect. To determine if a 30-month stay is in effect, consult with the Director, Division of Regulatory Policy II, Office of Regulatory Policy (HFD-007) and attach a summary of the response.*	() Yes () No
❖ Exclusivity (approvals only)	
• Exclusivity summary<br>• Is there remaining 3-year exclusivity that would bar effective approval of a 505(b)(2) application? (Note that, even if exclusivity remains, the application may be tentatively approved if it is otherwise ready for approval.)	Yes
• Is there existing orphan drug exclusivity protection for the "same drug" for the proposed indication(s)? *Refer to 21 CFR 316.3(b)(13) for the definition of "same drug" for an orphan drug (i.e., active moiety). This definition is NOT the same as that used for NDA chemical classification.*	() Yes, Application #________<br>(✓) No
Administrative Reviews (Project Manager, ADRA) *(indicate date of each review)*	Not Applicable

General Information	
Actions	
• Proposed action	(✓) AP () TA () AE () NA
• Previous actions (specify type and date for each action taken)	Approvable, 11-30-04
• Status of advertising (approvals only)	(✓) Materials requested in AP letter<br>() Reviewed for Subpart H
❖ Public communications	
• Press Office notified of action (approval only)	(✓) Yes () Not applicable
• Indicate what types (if any) of information dissemination are anticipated<br>*To be determined by Press Office.*	() None<br>() Press Release<br>() Talk Paper<br>() Dear Health Care Professional Letter
❖ Labeling (package insert, patient package insert (if applicable), MedGuide (if applicable))	
• Division's proposed labeling (only if generated after latest applicant submission of labeling)	✓ final agreed upon with firm
• Most recent applicant-proposed labeling	Not Applicable
• Original applicant-proposed labeling	Not Applicable
• Labeling reviews (including DDMAC, DMETS, DSRCS) and minutes of labeling meetings *(indicate dates of reviews and meetings)*	Not Applicable
• Other relevant labeling (e.g., most recent 3 in class, class labeling)	Not Applicable
❖ Labels (immediate container & carton labels)	
• Division proposed (only if generated after latest applicant submission)	Not Applicable
• Applicant proposed	Not Applicable
• Reviews	Not Applicable
❖ Post-marketing commitments	
• Agency request for post-marketing commitments	YES, see AP letter
• Documentation of discussions and/or agreements relating to post-marketing commitments	See AP letter
❖ Outgoing correspondence (i.e., letters, E-mails, faxes)	✓
❖ Memoranda and Telecons	See AE Package
❖ Minutes of Meetings	
• EOP2 meeting	Not Applicable
• Pre-sNDA meeting	See AE Package
• Pre-Approval Safety Conference	Not Applicable
• Other	See AE Package
❖ Advisory Committee Meeting	
• Date of Meeting	Not Applicable
• 48-hour alert	Not Applicable
❖ Federal Register Notices, DESI documents, NAS/NRC reports (if applicable)	Not Applicable

**Summary Application Review**	
Summary Reviews (e.g., Office Director, Division Director, Medical Team Leader))	✓
**Clinical Information**	
❖ Clinical review(s)	✓
❖ Microbiology (efficacy) review(s)	Not Applicable
❖ Safety Update review(s)	Not Applicable
❖ Risk Management Plan review(s)	Not Applicable
❖ Pediatric Page(separate page for each indication addressing status of all age groups)	✓
❖ Demographic Worksheet *(NME approvals only)*	Not Applicable
❖ Statistical review(s)	See AE Package
❖ Biopharmaceutical review(s)	Not Applicable
❖ Controlled Substance Staff review(s) and recommendation for scheduling	Not Applicable
❖ Clinical Inspection Review Summary (DSI)	
• Clinical studies	See AE Package
• Bioequivalence studies	Not Applicable
**CMC Information**	
❖ CMC review(s)	See AE Package
❖ Environmental Assessment	
• Categorical Exclusion	See AE Package
• Review & FONSI	Not Applicable
• Review & Environmental Impact Statement	Not Applicable
❖ Microbiology (validation of sterilization & product sterility) review(s)	Not Applicable
❖ Facilities inspection (provide EER report)	Not Applicable<br>Date completed:<br>( ) Acceptable<br>( ) Withhold recommendation
❖ Methods validation	Not Applicable<br>( ) Completed<br>( ) Requested<br>( ) Not yet requested
**Nonclinical Pharm/Tox Information**	
❖ Pharm/tox review(s), including referenced IND reviews	Not Applicable
❖ Nonclinical inspection review summary	Not Applicable
❖ Statistical review(s) of carcinogenicity studies	Not Applicable
❖ CAC/ECAC report	Not Applicable

This is a representation of an electronic record that was signed electronically and this page is the manifestation of the electronic signature.

/s/

Doris Bates
3/1/05 02:03:56 PM
AP checklist: covers one SE1 and 2 SLRs

DEPARTMENT OF HEALTH & HUMAN SERVICES

Public Health Service

Food and Drug Administration
Rockville, MD 20857

NDA 21-436, S-005

Otsuka Maryland Research Institute
Attn: Dr. Kusuma Mallikaarjun
Director, Regulatory Affairs
2440 Research Boulevard
Rockville, Maryland 20850

Dear Dr. Mallikaarjun:

We acknowledge receipt on January 4, 2005 of your January 3, 2005 submission to the above referenced supplemental new drug application for ABILIFY (aripiprazole) Tablets.

We have completed our initial evaluation of this submission, and we consider it a complete, class 1 response to our November 30, 2004 action letter. Therefore, the primary user fee goal date is March 4, 2005.

As you are also aware, under the Pediatric Research Equity Act (PREA), all applications for new active ingredients, new dosage forms, new indications, new routes of administration, and new dosing regimens are required to contain an assessment of the safety and effectiveness of the product in pediatric patients unless this requirement is waived or deferred. In connection with this requirement, we reference our action letter of November 30, 2004 and your secure emails of January 18, 2005 confirming that clinical and supporting preclinical studies are in development to support both S-002 and S-005.

If you have any questions, please call the undersigned, at 301-594-2850.

Sincerely,

{See appended electronic signature page}

Doris J. Bates, Ph.D.
Regulatory Project Manager
Division of Neuropharmacological Drug Products
Office of Drug Evaluation I
Center for Drug Evaluation and Research

This is a representation of an electronic record that was signed electronically and this page is the manifestation of the electronic signature.

/s/

Doris Bates
1/18/05 02:45:44 PM

Bates, Doris J

From: Bates, Doris J
Sent: Tuesday, January 18, 2005 2:49 PM
To: 'Susan H Behling'; Bates, Doris J
Cc: kusuma mallikaarjun
Subject: RE: URGENT RE: S-005 Phase IV Commitments

Complete
ss 1 LetrDFS.pd

Good afternoon Susan and Kusuma,

Attached is our official acknowledgement that your January 3 submission to NDA 21-436 S-005 is a complete Class I response, with an action due date of March 4, 2005.

Doris J. Bates, Ph.D.
Regulatory Project Manager
Division of Neuropharmacological Drug Products
Office of Drug Evaluation I
Center for Drug Evaluation and Research

-----Original Message-----
From: Susan H Behling [mailto:Susan.Behling@bms.com]
ent: Tuesday, January 18, 2005 1:33 PM
To: Bates, Doris J
Cc: kusuma mallikaarjun
Subject: Re: URGENT RE: S-005 Phase IV Commitments

Hi again Doris- Hope you got my previous e-mail. I just wanted to point out that the approvable letter states "No additional commitment is necessary for S-005 if the previous agreed upon commitment is met" for items 1 and 2. This is the only reason there was no mention of it in the response. For item 2, juvenile toxicology studies, our S-002 is still applicable with no changes.

Sue
Bates, Doris J wrote:

>Doris J. Bates, Ph.D.
>Regulatory Project Manager
>Division of Neuropharmacological Drug Products
>Office of Drug Evaluation I
>Center for Drug Evaluation and Research
>
>Good morning Susan, this is a rather urgent question.
>
>We note that the January 3 response for S-005 makes no mention of the
two pending Phase 4 commitments which we consider will be met for S-005
>if they are met for S-002. We need some kind of information on the
>status of these two commitments, in order for your response to be
>considered complete. There is no mention of either at all, and we are

>thus unable to determine BMS' intentions with respect to S-005 from
>what we have in hand.
>
>As the deadline for our making this decision is COB today, can you
>please update us via secure email as soon as possible.
>
>Thank you,
>
>Doris J. Bates, Ph.D.
>Regulatory Project Manager
>Division of Neuropharmacological Drug Products
>Office of Drug Evaluation I
>Center for Drug Evaluation and Research
>Food and Drug Administration
>
>
>

"MMS <cder.fda.gov>" made the following annotations.

This message was sent from Bristol-Myers Squibb, Co. across the Internet in encrypted format and was successfully decrypted, unless otherwise noted.
Bristol-Myers Squibb
===

Appears This Way
On Original

Bates, Doris J

**~rom:**	Bates, Doris J
**.ent:**	Tuesday, January 18, 2005 1:58 PM
**To:**	'Susan H Behling'; Bates, Doris J
**Cc:**	kusuma mallikaarjun
**Subject:**	RE: URGENT RE: S-005 Phase IV Commitments

No problem Susan, it's perfectly understandable.

We tend to see point-by-point responses acknowledging each of the questions in an action letter, even where an issue has been settled or subsumed into another point, so we were concerned that we didn't see anything regarding these two points. We weren't sure how to interpret that. (If we'd been here yesterday we'd have been in touch then.)

All looks well at this point for a Complete Class 1 response, with a two month date. You will get a formal letter from us later today, confirming this.

Thanks again,

Doris J. Bates, Ph.D.
Regulatory Project Manager
Division of Neuropharmacological Drug Products
Office of Drug Evaluation I
enter for Drug Evaluation and Research

-----Original Message-----
From: Susan H Behling [mailto:Susan.Behling@bms.com]
Sent: Tuesday, January 18, 2005 1:33 PM
To: Bates, Doris J
Cc: kusuma mallikaarjun
Subject: Re: URGENT RE: S-005 Phase IV Commitments

Hi again Doris- Hope you got my previous e-mail. I just wanted to point out that the approvable letter states "No additional commitment is necessary for S-005 if the previous agreed upon commitment is met" for items 1 and 2. This is the only reason there was no mention of it in the response. For item 2, juvenile toxicology studies, our S-002 is still applicable with no changes.

Sue
Bates, Doris J wrote:

>Doris J. Bates, Ph.D.
>Regulatory Project Manager
>Division of Neuropharmacological Drug Products
>Office of Drug Evaluation I
Center for Drug Evaluation and Research
>
>Good morning Susan, this is a rather urgent question.
>

>We note that the January 3 response for S-005 makes no mention of the
>two pending Phase 4 commitments which we consider will be met for S-005
>if they are met for S-002. We need some kind of information on the
status of these two commitments, in order for your response to be
>considered complete. There is no mention of either at all, and we are
>thus unable to determine BMS' intentions with respect to S-005 from
>what we have in hand.
>
>As the deadline for our making this decision is COB today, can you
>please update us via secure email as soon as possible.
>
>Thank you,
>
>Doris J. Bates, Ph.D.
>Regulatory Project Manager
>Division of Neuropharmacological Drug Products
>Office of Drug Evaluation I
>Center for Drug Evaluation and Research
>Food and Drug Administration
>
>
>

"MMS <cder.fda.gov>" made the following annotations.

This message was sent from Bristol-Myers Squibb, Co. across the Internet in ncrypted format and was successfully decrypted, unless otherwise noted.
Bristol-Myers Squibb
===

**APPEARS THIS WAY
ON ORIGINAL**

Bates, Doris J

**From:**	Susan H Behling [Susan.Behling@bms.com]
**Sent:**	Tuesday, January 18, 2005 12:49 PM
**To:**	Bates, Doris J
**Cc:**	kusuma mallikaarjun
**Subject:**	Re: URGENT RE: S-005 Phase IV Commitments

Hi Doris- We commit to the 2 relevant S-002 commitments for S-005 with the same proposed timeframes as for S-002. The protocol for the acute add-on trial has been submitted to the IND already and was initiated in November. The long term add-on study is planned as per the S-002 commitment. Please let me know if you need further details.

Sue

Bates, Doris J wrote:

>Doris J. Bates, Ph.D.
>Regulatory Project Manager
>Division of Neuropharmacological Drug Products
>Office of Drug Evaluation I
>Center for Drug Evaluation and Research
>
>Good morning Susan, this is a rather urgent question.
>
We note that the January 3 response for S-005 makes no mention of the
>two pending Phase 4 commitments which we consider will be met for S-005
>if they are met for S-002. We need some kind of information on the
>status of these two commitments, in order for your response to be
>considered complete. There is no mention of either at all, and we are
>thus unable to determine BMS' intentions with respect to S-005 from
>what we have in hand.
>
>As the deadline for our making this decision is COB today, can you
>please update us via secure email as soon as possible.
>
>Thank you,
>
>Doris J. Bates, Ph.D.
>Regulatory Project Manager
>Division of Neuropharmacological Drug Products
>Office of Drug Evaluation I
>Center for Drug Evaluation and Research
>Food and Drug Administration
>
>
>

"MMS <cder.fda.gov>" made the following annotations.

This message was sent from Bristol-Myers Squibb, Co. across the Internet in encrypted format and was successfully decrypted, unless otherwise noted.

APPEARS THIS WAY
ON ORIGINAL

Bristol-Myers Squibb
Pharmaceutical Research Institute
Richard L. Gelb Center for Pharmaceutical Research and Development
5 Research Parkway P.O. Box 5100 Wallingford, CT 06492-7660

ABILIFY ® (aripiprazole) Tablets, S-005 **Response to Approvable Letter**

January 3, 2005

Russell Katz, M.D., Director
Division of Neuropharmacologic Drug Products
Center for Drug Evaluation and Research
Food and Drug Administration
Woodmont II Building
Attention: Document Control Room - HFD #120
1451 Rockville Pike
Rockville, MD 20852

Dear Dr. Katz:

Reference is made to NDA 21-436 for ABILIFY Tablets and to the November 30, 2004 Approvable Letter for S-005 for the maintenance of efficacy in the treatment of patients with Bipolar I Disorder. Further reference is made to Submission No. 223 (dated November 16, 1999) to IND 42, 776 in which we informed the Division of the collaborative agreement between Otsuka Pharmaceutical Co. Ltd. (OPC) and Bristol-Myers Squibb Company (BMS) such that BMS is delegated to act on behalf of OPC in correspondence with this Division. We also refer to the 'Changes Being Effected' (CBE) supplemented submitted on December 27, 2004 and approved NDA 21-713 for ABILIFY Oral Solution.

Provided herewith is our response to the approvable letter for this application. In this response we have addressed the two 'Efficacy' comments highlighted in the Approvable Letter, and we provide a revised labeling proposal that reflects our consideration of the Division's comments. Please note that while the Approvable Letter included a request to include WARNING language on the risk of cerebrovascular adverse events (CVAEs) in elderly patients with dementia on the basis of data submitted on July 30, 2003 in response to the Division's request of January 30, 2003, we do not agree that the data available at the time of our July, 2003 response warranted a labeling revision. However, in our May 20, 2004 update, we did propose a change to the label on the basis of the results of 3 completed studies, and we have since had feedback from the Division on this labeling language. We have agreed to proceed with the submission of a CBE supplement to implement some modified language for this 'WARNING' and this revised language is reflected in this labeling. This labeling also includes the revisions approved by the Division on December 10, 2004 with the approval of the Oral Solution NDA. Please also note that the approved labeling for this product should carry the Otsuka copyright statement as delineated in the labeling included with this response.

We commit to providing reports of the two drug interaction studies (lithium and valproate) by June 30, 2005. In addition, we will submit the proposed promotional materials subsequent to the approval of this application.

We believe this response addresses all of the deficiencies listed in the approvable letter. We look forward to working with the Division to complete the review and approval of this application. If you have any questions, please call me at 203-677-3810 or contact me via e-mail at Susan.Behling@bms.com.

Sincerely,

Susan H Behling

Susan H. Behling, Director
Global Regulatory Science

Desk Copy Cover Letter: Dr. Doris Bates

Bates, Doris J

From: Racoosin, Judith A
Sent: Monday, November 22, 2004 11:25 AM
To: Andreason, Paul J
Cc: Stone, Marc; Hardeman, Steven D; Bates, Doris J
Subject: CVAE labeling for aripiprazole

Warnings

b(4)

Cerebrovascular Adverse Events, Including Stroke, in Elderly Patients with Dementia —
[ ] (e.g., stroke, transient ischemic attack), including fatalities, [ ]
[ ] patients [ ] In [ ] fixed dose [ ]
there was a statistically significant dose response relationship for cerebrovascular adverse events in patients treated with aripiprazole. Aripiprazole is not approved for the treatment of patients with dementia-related psychosis.

APPEARS THIS WAY
ON ORIGINAL

Bates, Doris J

From: Bates, Doris J
Sent: Tuesday, November 16, 2004 10:52 AM
To: Bates, Doris J
Subject: FW: FW: NDA 21-436 S-005: Answers to Questions, Dr. Podruchny

-----Original Message-----
From: Susan H Behling [mailto:Susan.Behling@bms.com]
Sent: Monday, November 15, 2004 1:58 PM
To: Podruchny, Teresa
Cc: Bates, Doris J; Andreason, Paul J; kusuma mallikaarjun
Subject: Re: FW: NDA 21-436 S-005

Hi Teresa: Please see our responses to your questions below:

1) All of the AEs for CN138-037 are contained in ISSQADR3.xpt and identified by the variable AE_PROT when it takes the value 'CN138-037'. These records may be selected by using the ROWS , ROW SELECTION, SELECT WHERE, selecting variable AE_PROT in the variable window, selecting 'equals' for the comparator and typing 'CN138-037' in the value window. If you need to find an AE that occurred in CN138-037 from a specific patient and have their uniq_id from CN138-037 you can use the variable UNIQ_ID2 to select the AEs reported by the patient in CN138-037. AEs that occurred during CN138-037 are summarized in the CSR for CN138-037.

2) We are not certain about your question regarding 'additional information' on the 35 patients who were unblinded. We did send an e-mail dated April 16, 2004 in which we responded to clinical bullets #1 & #2 in the 74-day letter (formal submission of this information was also sent on July 22). In that response we described how to find demographic, efficacy, and safety data for the 35 patients in the CN138-010 Individual SAS Data Sets. In addition, we mentioned that the safety data for these patients is also contained in the Composite Safety Data Set "Merged" Files Structured for Viewing in SAS+JMP, but added that since these files were originally designed for review of the overall safety of aripiprazole, there was no way to specifically select out the 35 patients. However, the data for the 35 patients are included in the overall safety databases and are flagged as such.

The following tables in the Clinical Study Report provide separate safety summaries and listings during the Maintenance Phase for the 35 patients:

Table Number	Table Title
8.2D	Discontinuation Reasons for Patients Excluded Due to Labeling Error
S.12.1F	Incidence of Treatment-Emergent Adverse Events for Patients Who Received Unblinded Study Medication During the Maintenance Phase
S.12.3E	Listing of Patients with Serious Adverse Events Other Than Death, for Patients Who Received Unblinded Study Medication During the Maintenance Phase
S.12.3F	Incidence of Serious Adverse Events During the Maintenance Phase for Patients who Received Unblinded Study Medication During the Maintenance Phase
S.12.3G	Narratives for Patients who Received Unblinded Study Medication During the Maintenance Phase and Who Experienced Serious Adverse Events

S.12.4D	Listing of Discontinuations for Adverse Events during the Maintenance Phase for Patients Who Received Unblinded Study Medication During the Maintenance Phase
S.12.4E	Incidence of Discontinuations for Adverse Events During the Maintenance Phase for Patients Who Received Unblinded Study Medication During the Maintenance Phase
S.12.5.4B	Incidence of Treatment-Emergent EPS-Related Adverse Events for Patients Who Received Unblinded Study Medication During the Maintenance Phase
S.12.6E	Patients with Potentially Clinically Significant Laboratory Abnormalities Who Received Unblinded Study Medication During the Maintenance Phase
S.12.8.1E	Patients with Potentially Clinically Significant Vital Sign Abnormalities for Patients Who Received Unblinded Study Medication in the Maintenance Phase
Note: No clinically significant ECG abnormalities were observed during the Maintenance Phase for patients who received unblinded study medication in the Maintenance Phase. Therefore, no such tables were provided.	

Please let us know if this does not adequately respond to your questions.

Sincerely,

Sue
Podruchny, Teresa wrote:

Hello,

I am unable to locate a few things and was hoping you could help.
1) Regarding ISSqadr3.xpt (maint submission ISS JMP files), I do not see trial 138037 included in this dataset although Appendix 2 of the define file for the ISS (1-28-04 submission) lists this as present in the file mentioned. Are all AEs for this trial contained in the CSR?
2) Perhaps I am mistaken, however, as I recall, you submitted additional information regarding the 35 patients who received unblinded medication. Also, as I recall, the safety data for these patients were evaluated separately. Could you please provide the submission date and number or the location of that additional information on the 35 patients and also the location of the safety data for those patients.

Thanks for your assistance in these matters.
Kind regards,
Teresa
P.S. I am out of the office for the rest of the day.

"MMS <cder.fda.gov>" made the following annotations.

This message was sent from Bristol-Myers Squibb, Co. across the Internet in encrypted format and was successfully decrypted, unless otherwise noted. Bristol-Myers Squibb
==

This is a representation of an electronic record that was signed electronically and this page is the manifestation of the electronic signature.

/s/

Doris Bates
11/16/04 04:16:08 PM
CSO
see emails for correspondence times and dates.

FILING MEETING MINUTES
NDA 21-436/S-005

DATE OF MEETING: March 22, 2004
FILING DATE: March 30, 2004

BACKGROUND: ABILIFY (aripiprazole) is approved for the treatment of schizophrenia. Mania supplements currently in hand for this chemical entity are listed below:

Supplement Number	Indication	Submitted	PDUFA Date
S-002	acute manic/mixed bipolar I, monotherapy	23JUN03	23APR04
S-005	maintaining stability, bipolar I monotherapy	28JAN04	30NOV04

b(4)

ATTENDEES: listed below.

ASSIGNED REVIEW TEAM:

Discipline	Team Leader /Primary Reviewer
Medical:	Paul Andreason / Teresa Podruchny
Secondary Medical:	none
Statistical:	Kun Jin / Kun He
Pharmacology:	Lois Freed / Sonia Tabacova
Statistical Pharmacology:	none
Chemistry:	Tom Oliver / Sherita McLamore
Environmental Assessment (if needed):	none (cat. exclusion requested)
Biopharmaceutical:	Ray Baweja / Kofi Kumi
Microbiology, sterility:	none
Microbiology, clinical (for antimicrobials only):	none
DSI:	Ni Khin
Regulatory Project Management:	D. J. Bates
Other Consults:	no ODS, CSS, or DDMAC consults needed.

Per reviewers, are all parts in English or English translation? YES NO

CLINICAL FILE __ see comments___ REFUSE TO FILE _____

- Clinical site inspection needed: YES, overseas NO
- Advisory Committee Meeting needed? YES, date if known _________ NO
- If the application is affected by the AIP, has the division made a recommendation regarding whether or not an exception to the AIP should be granted to permit review based on medical necessity or public health significance?

  N/A YES NO

CLINICAL MICROBIOLOGY NA FILE ______ REFUSE TO FILE ______

STATISTICS FILE ______ REFUSE TO FILE __see comments_____

BIOPHARMACEUTICS FILE __✓____ REFUSE TO FILE ______

- Biopharm. inspection needed: YES ***NO***

PHARMACOLOGY NA ______ FILE __✓_____ REFUSE TO FILE ______

- GLP inspection needed: YES ***NO***

CHEMISTRY FILE __✓_____ REFUSE TO FILE ______

- Establishment(s) ready for inspection? *NA* YES NO
- Microbiology *NA* YES NO

ELECTRONIC SUBMISSION: see below.

GENERAL COMMENTS:

- Clinical reviewer noted that the e-submission is disorganized and therefore difficult to review. 35 patients appear to have been excluded from analysis. No explanation was found. This is a potential filing issue.
- Statistical reviewer comments that primary efficacy data appear to have been placed in appendices that were not included as part of the submission, but must be requested by FDA if desired. This is a definite RTF issue.
- CMC review was completed prior to the filing meeting. Categorical exclusion can be granted. Approval recommendation for CMC.
- Biopharm has no significant review responsibility. Interaction studies were adequately addressed in the context of S-002, the acute mania supplement. *These meeting minutes serve as documentation that no OCPB review will be required for this supplement.*
- PharmTox has two studies to examine which have not been previously submitted but are not expected to have significant impact on labeling.
- A foreign DSI inspection (Mexico) will be required; a consult will be prepared.

REGULATORY CONCLUSIONS/DEFICIENCIES:

Meeting consensus: RTF (Statistics) if missing data cannot be provided and made accessible to primary statistics reviewer with sufficient time to evaluate their completeness prior to Filing Date.

The firm was contacted immediately and the filing issues explained. The firm was informed that the missing information had to be posted to the server in the EDR before the afternoon of March 26 (Friday) in order to allow the clinical and statistical reviewers a modicum of time to examine the contents before the filing decision date of March 30, 2004. The application, following repair by the applicant of these RTF-level severe deficiencies, was rendered suitable for filing prior to the official filing date. It was therefore filed.

Clinical and statistics non-RTF filing review issues were identified after receipt of the missing information. These will be communicated in the 74-Day Letter.

FINAL CONCLUSIONS/ACTION ITEMS:

Submission Filed Following Repair of RTF-Level Severe Deficiencies By Applicant Prior To Official Filing Date.

POST MEETING NOTES:

1. S-005 was filed on March 30, 2004.
2. The 74-day letter for S-005 was issued on April 12, 2004.
3. An AE action was taken on S-002 on April 23, 2004. **b(4)**
4. [ ]
5. Incomplete responses for S-002 were received by the Division on May 26 and July 19, 2004. The S-002 response was finally completed by a submission received July 28, 2004.
6. S-002 was approved on September 29, 2004. Approval included partial waiver/partial deferral for pediatric studies and three Phase 4 commitments (acute add-on therapy study in adults, longer-term add-on therapy study in adults, and juvenile animal toxicology to support pediatric studies).

APPEARS THIS WAY
ON ORIGINAL

This is a representation of an electronic record that was signed electronically and this page is the manifestation of the electronic signature.

/s/

Doris Bates
11/16/04 04:31:17 PM
These minutes will also be cross-filed as an OCPB review, since they document the fact that no OCPB review is needed for this supplement.

This is a representation of an electronic record that was signed electronically and this page is the manifestation of the electronic signature.

/s/

Doris Bates
11/16/04 04:51:10 PM
CSO
This document confirms that no OCPB review of S-005 is required.

DEPARTMENT OF HEALTH & HUMAN SERVICES Public Health Service

Division of Scientific Investigations
Office of Medical Policy
Center for Drug Evaluation and Research
Food and Drug Administration
Rockville MD 20855

CLINICAL INSPECTION SUMMARY

DATE: November 3, 2004

TO: Doris Bates, Ph.D, Regulatory Project Manager
Teresa A. Podruchny, M.D., Medical Officer
Division of Neuropharmacological Drug Products, HFD-120

THROUGH: Joseph Salewski, Deputy Director
Division of Scientific Investigations

FROM: Ni A. Khin, M.D., Branch Chief
Good Clinical Practice Branch I, HFD-46
Division of Scientific Investigations

SUBJECT: Evaluation of Clinical Inspection

NDA: NDA 21-436/SE1-005

APPLICANT: Bristol-Myers Squibb/Otsuka Pharmaceuticals

DRUG: Abilify (aripiprazole)

THERAPEUTIC CLASSIFICATION: Type S

PROPOSED INDICATION: Maintenance Treatment of Bipolar I Disorder

CONSULTATION REQUEST DATE: March 22, 2004

ACTION GOAL DATE: November 30, 2004

I. BACKGROUND:

Abilify (aripiprazole) is an atypical antipsychotic agent. It is approved for use in treatment of schizophrenia. In this application, the sponsor has requested the use of aripiprazole in maintenance treatment of Bipolar I Disorder. The application included the result of protocol CN138-010 entitled "a multicenter, randomized, double-blind, placebo-controlled study of aripiprazole in the Maintenance Treatment of Patients with Bipolar Disorder."

This study was a multi-center, randomized, double-blind, placebo-controlled study. The primary objective of the study was the maintenance of stability of aripiprazole versus placebo as measured

by the time to relapse (i.e., discontinuation due to lack of efficacy) during the Maintenance phase. Patients were discontinued from the study due to lack of efficacy if they were hospitalized for manic or depressive symptoms or required an addition or increase in their allowed psychotropic medication.

Patients who completed an acute mania study of aripiprazole were eligible to enter the study. Patients who recently experienced a manic episode but did not participate in an aripiprazole trial were eligible to enter this study. For those patients who entered as outpatient would enroll into the stabilization phase and could participate in a screening period up to 28 days with a minimum one-day washout period for antipsychotics.

The study consisted of stabilization phase (up to 18 weeks), maintenance phase (up to 26 weeks) and extension phase (up to 74 weeks). During the stabilization phase, patients would receive open-label treatment with aripiprazole 30 mg/day. The dose could be decreased to 15 mg/day at any time if necessary for side effects. Patients would continue in the stabilization phase until a Young Mania Rating Scale (Y-MRS) score of $\leq$10 and a Montgomery Asberg Depression Rating Scale (MADRS) score of $\leq$13 during four consecutive visits. Eligible patients would then be randomized to one of two treatment groups (aripiprazole vs. placebo) during the maintenance phase. Patients assigned to aripiprazole would start the maintenance phase at the same dose they were taking at the end of the stabilization phase. The dose of study drug could be changed at any time during the study as necessary to enhance therapeutic effect and/or tolerability. The patients would continue in the maintenance phase for up to 26 weeks. If the patients continued in the extension phase, they could continue on their current study drug treatment for up to 74 weeks.

The primary efficacy outcome measure was the time to relapse as defined by discontinuation due to lack of efficacy from randomization. Patients were discontinued from the study due to lack of efficacy if they were hospitalized for manic or depressive symptoms or required an addition to or increase in their allowed psychotropic medications.

As per the request of the Review Division (HFD-120), inspection assignments were issued in May 2004 for three sites: one U.S. and two sites in Mexico. These clinical investigators were chosen for the sample size and/or their contribution for significant results.

II. RESULTS (by site):

NAME	Protocol (site #)	Location	ASSIGNED DATE	DATE EIR RECEIVED	CLASSIFICATION
Tram K. Tran-Johnson, Pharm.D.	CN138-010 (site 64)	San Diego, CA	5/7/2004	7/16/2004	VAI
Ignatio Rosales, M.D.	CN138-010 (site 93)	Mexico City, Mexico	5/7/2004	9/24/2004	VAI-RR
Miguel Herrera-Estrella	CN138-010 (site 118)	Mexico City, Mexico	5/7/2004	9/30/2004	VAI-RR

1. Tram K. Tran-Johnson, Pharm.D. (site 64)

What was inspected:

For protocol CN138-010, 27 subjects were enrolled in the stabilization phase of open label treatment with aripiprazole 30 mg/day. Reasons for discontinuation from the stabilization phase included withdrawal of consent, lost to follow up or adverse events.

Out of these 27 subjects, 8 subjects were randomized in the maintenance phase and only two subjects completed the study. Reasons for discontinuation included the following.

- Subject 104 was discontinued after week 4 visit due to compromising the blind as the package was imprinted with the name and dosage (aripiprazole 15 mg) by the manufacturer.
- Subject 179 was terminated due to lack of efficacy: depression which required treatment with paroxetine and neurontin after week 4 visit.
- Subject 238 was lost to follow up after week 12 visit.
- Subject 279 was listed as lack of efficacy on week 1.
- Subject 349 was due to lack of efficacy based on the Y-MRS scores at week 14. This subject was enrolled in the maintenance phase without meeting the inclusion criterion that the patient must have 4 consecutive Y-MRS scores of equal or less than 10 to enter the maintenance phase. The study coordinator noticed this error and notified the sponsor. The sponsor approved the patient to continue in the study.
- Subject 429 withdrew the consent after week 3 visit as it was noted that he was fearful to lose his [redacted] because of his participation in the clinical study. b(6)

Two subjects participated in the extension phase. Subject 416 was terminated at week 60 of the extension phase as the investigator determined that the subject met the criteria for relapse. Subject 288 discontinued from the study after week 44 due to worsening of involuntary movement in the right leg and tongue tremor (tardive dyskinesia).

Limitations of inspection: N/A

General observations/commentary:

The protocol specified a positive screen of lithium, divalproex acid or drug of abuse as one of the exclusionary criteria for enrollment in maintenance phase of the study. The lithium and divalproex acid levels for two subjects (416 and 179) were not performed as required by the protocol.

For three subjects, the following source documents were not available for review and therefore, the FDA investigator was not able to verify the data.
Subject 104: all source documents
Subject 160: signed informed consent, screening source documents
Subject 039: source documents from baseline evaluations

The protocol specified that the patient must have 4 consecutive Y-MRS scores of equal or less than 10 to enter the maintenance phase. Subject 349 was enrolled in the maintenance phase without meeting this inclusion criterion. The study coordinator noticed the error and notified the sponsor. The sponsor approved the patient to continue in the study.

Recommendation:

The review division should note above protocol deviations and record keeping deficiencies. The review division should consider any impact of these findings on study data. Otherwise, data appear acceptable.

2. Ignatio Rosales, M.D. (site 93)

What was inspected:

For protocol CN138-010, 18 subjects were screened and enrolled in the stabilization phase of open label treatment with aripiprazole 30 mg/day. During the stabilization phase, 2 subjects (126 and 514) were reported by the clinical investigator that they withdrew their consent and subject 427 discontinued because of adverse event.

During the maintenance phase of the study, three subjects (101, 102 and 114) were discontinued due to compromising the blind as the package was imprinted with the name and dosage (aripiprazole 15 mg) by the manufacturer. Five subjects (184, 198, 495, 533 and 542) were discontinued for lack of efficacy. Subject 504 discontinued because of adverse event/lack of efficacy. Five subjects (154, 196, 501, 532, 535) entered in the extension phase of the study. There were 3 serious adverse events reported at this site.

An audit of all 18 subjects' records was conducted. A Form FDA-483 was issued at the end of inspection. Dr. Rosales responded to the FDA-483 in writing. DSI received a copy of Dr. Rosales response on September 28, 2004.

Limitations of inspection: The source documents were written in Spanish.

General observations/commentary:

The protocol specified that patients who have a positive screen for lithium, divalproex acid or drugs of abuse be excluded from entering the maintenance phase of the study. The site did not perform the lithium and divalproex acid levels for four subjects (# 533, 535, 538 and 542). The site performed these protocol required tests after randomization to enrollment in the maintenance phase for three subjects (# 101, 102, and 114).

There were multiple instances of protocol required clinical laboratory tests (hematology, urine), prolactin levels, pregnancy tests and drug screens that were not performed. For all 18 subjects, the number of missing tests ranged from one to fifteen tests per subject.

AE reporting: the following AE were not reported to the sponsor.

Subject #	Week	Event
101	Wk # 1 (Stabilization)	Precordial pain
	Wk # 2 (Stabilization)	Photosensitivity, loss of appetite, Increased daytime urination
495	Wk # 6 (Stabilization)	Slowness in thinking

Inadequate record keeping

There were several instances when events documented on the source document (clinic record) did not coincide with the CRF. For example,

For subject #126, it was documented in the source document that the subject has poor compliance with the study medication and the family could not monitor the subject. The subject was discontinued from the study. The reason for discontinuation is reported on the CRF as withdrawal of consent.

The protocol specified that subjects enter the stabilization phase with a recent manic or mixed episode requiring hospitalization that began no more than 3 months before entry into the stabilization phase. During the hospitalization, the subject #198 was treated with medication for acute mania. The source document for this subject reports no prior hospitalization.

Subject #427 refused hospitalization for the last manic episode that occurred more than three months before entry in the study. There was no documentation that the sponsor was notified and was granted permission for the subject to enter the study. The source documents report that this subject was experiencing adverse events, no mention of depressive symptoms. However, the subject was discontinued from the study for relapse with a MADRS score of 17. The CRF listed the reason for discontinuation as Lack of Efficacy while the comment section in the CRF reported that the subject did not want to continue in trial because of the adverse events.

Other observations:

Although Dr. Rosales signed the Form FDA-1572, the sponsor reported that it was not submitted to the Agency and therefore, this site was listed as a non-IND site.

The protocol consisted of stabilization phase (up to 18 weeks), maintenance phase (up to 26 weeks) and extension phase (up to 74 weeks). During the stabilization phase, subjects would receive open-label treatment with aripiprazole 30 mg/day. The dose could be decreased to 15 mg/day at any time if necessary for side effects. The protocol specified that subjects would continue in the stabilization phase until a Young Mania Rating Scale (Y-MRS) score of $\leq$10 and a

Montgomery Asberg Depression Rating Scale (MADRS) score of ≤13 during four consecutive visits. Eligible subjects would then be randomized to one of two treatment groups (aripiprazole vs. placebo) during the maintenance phase.

During the maintenance phase of the study, three subjects (101, 102 and 114) were discontinued due to compromising the blind as the package was imprinted with the name and dosage (aripiprazole 15 mg) by the manufacturer. The sponsor reported that these 3 subjects' data were excluded from primary efficacy data analysis.

Following this incidence, the sponsor continued to have manufacturing issue and the site was not able to receive the double-blind study medication. Four subjects (154, 184, 196 and 198) met the protocol specified criteria to be entered in maintenance phase; i.e., a Young Mania Rating Scale (Y-MRS) score of ≤10 and a Montgomery Asberg Depression Rating Scale (MADRS) score of ≤13 during four consecutive visits. The site, however, continued these 4 subjects on open-label stabilization phase up to 18 weeks. The sponsor approved these 4 subjects to be continued in the stabilization phase of study.

In addition, four subjects (# 495, 504, 532 and 533) were continued on the open label stabilization phase after they had met the same protocol requirement for entry to the maintenance phase.

The review division should consider whether this issue would have any differential effect on primary efficacy data.

Recommendation:

DSI suggests the review division to consider excluding data from the subjects who did not meet all eligibility criteria and to note lack of laboratory tests and missing AE for two subjects in safety data.

3. Miguel Herrera-Estrella, M.D. (Site 118)

What was inspected:

For protocol CN138-010, 28 subjects were screened and 25 subjects were enrolled in the stabilization phase of open label treatment with aripiprazole 30 mg/day. During the stabilization phase, subject 164 was reported by the clinical investigator that the subject withdrew the consent. Subject 422 was discontinued due to lack of efficacy and 2 subjects (251 and 454) were discontinued because of adverse event.

During the maintenance phase of the study, three subjects (148, 214 and 269) were discontinued for lack of efficacy. Subject 438 was discontinued due to positive drug screen for cocaine.

During the extension phase, two subjects (97 and 261) were discontinued from the study and their reason for discontinuation was listed as lost to follow up/non-compliance. Five subjects (222, 230, 246, 335 and 446) discontinued from the study due to lack of efficacy.

There were 13 serious adverse events reported at this site including 10 subjects with mild to severe mania during the study.

An audit of 10 subjects' records was conducted. A Form FDA-483 was issued at the end of inspection. Dr. Herrera Estrella responded to the FDA-483 in writing. DSI received a copy of Dr. Herrera Estrella's response on September 27, 2004.

Limitations of inspection: The source documents were written in Spanish.

General observations/commentary:

The protocol specified that patients who have a positive screen for lithium, divalproex acid or drugs of abuse be excluded from entering the maintenance phase of the study. The site did not perform the lithium, divalproex acid levels and drug screen for subject 405.

The protocol specified that the concomitant use of lorazepam up to a dose of 4 mg per day be allowed during the first 4 weeks of the stabilization phase of the study. The dose would be decreased to 3 mg/day for the 5th week and to 2 mg/day thereafter. During the maintenance phase of the study, the patient may take lorazepam 2 mg/day during the first month, 1 mg/day during the second month and up to 1 mg/day 4 times a week during the remaining 18 weeks. The following subjects received lorazepam outside the protocol specified dose.

Subject 97: 4 mg (wk #6, stabilization; wk #8-9, stabilization)
Subject 222: 3 mg (wk #28-32, extension)
Subject 390: 6 mg (wk #5-7, stabilization); 3 mg. (wk #8-9, stabilization)
Subject 422: 4 mg (wk # 7, stabilization)
Subject 438: 4 mg (wk # 5, stabilization); 3 mg (wk #6-7, stabilization)

According to the protocol, the subject would enter the maintenance phase of the study when patient is stable as evidenced by a Y-MRS scores that have been ≤ 10 during four consecutive visits and a MADRS score that have been ≤ 13 during four consecutive visits. The site enrolled subject #77 into the maintenance phase of the study at week #6.

According to the protocol, investigational drug supplies should be stored in a secure area, at 15 - 25°C (59 – 77°F). Temperature Logs document that minimum temperatures were as low as 3°C and maximum temperatures were as high as 28°C on several occasions at the site.

Subject 438 showed a positive test for cocaine at week 8 and also at week 16 during the maintenance phase of the study. Yet, this subject was allowed to continue in the study.

The site did not report the following adverse events experienced by the subjects during the study.

Subject 148 experienced loss of appetite at week 18. The subject also experienced nausea and dry mouth at week 20 during the study. The site did not report these adverse events to the sponsor.

The site submitted serious adverse event (SAE) reports to the sponsor for two subjects several weeks after the event occurred. Subject 148 experienced a severe manic episode and the subject was hospitalized on [redacted] The clinical investigator did not b(6)
report this serious adverse event to the sponsor until July 17, 2002. Similarly, subject 405 experienced a moderate manic episode on August 20, 2002, which was not reported to the sponsor until September 16, 2002.

There were several minor discrepancies between the source document (clinic record) and the CRF. For example,

Subject 77 had a baseline YMRS score of 25, which was recorded as 29.

For subject 222, the starting date for tongue protrusion was recorded as June 8, 2001 in the source document but recorded as June 29, 2001 in the CRF.

Recommendation:

The review division should note these findings of protocol deviations, adverse event reporting and record keeping issues.

In the final study report, the sponsor reported that there were 17 ECG missing for the stabilization phase. During the FDA inspection, the sponsor had identified that all these ECGs, except for one, were found upon querying the study data differently. It was noted in the EIR that some ECG tracings were missing but the ECG reports were available for review. There was no other specific information provided in the EIR.

According to the EIR, it is unclear if hospital charts were reviewed during the routine monitoring visits. The sponsor performed a pre-audit at this site prior to this FDA inspection. The sponsor sent a copy of additional findings to the Review Division via email in August 2004. Although it is less likely that these additional findings will have major impact on study outcome, the review division should consider including all these issues in your review.

III. OVERALL ASSESSMENT OF FINDINGS AND GENERAL RECOMMENDATIONS

As stated above, multiple instances of protocol deviations, adverse event reporting and record keeping issues noted in three study sites inspected.

The protocol specified that patients who have a positive screen for lithium, divalproex acid or

drugs of abuse be excluded from entering the maintenance phase of the study. The sites did not obtain serum levels of lithium and valproate levels in certain study subjects. Given the common use of mood stabilizers like lithium and valproate in treatment of Bipolar Disorder patients, the sites should have ensured that these levels were done prior to study drug treatment.

During the maintenance phase of the study, three subjects (101, 102 and 114) at Dr. Rosales site were discontinued due to compromising the blind as the package was imprinted with the name and dosage (aripiprazole 15 mg) by the manufacturer. The sponsor reported that these 3 subjects' data were excluded from primary efficacy data analysis. Following this incidence, the sponsor continued to have manufacturing issue and the site was not able to receive the double-blind study medication. Four subjects (154, 184, 196 and 198) met the protocol specified criteria to be entered in maintenance phase; i.e., a Young Mania Rating Scale (Y-MRS) score of ≤10 and a Montgomery Asberg Depression Rating Scale (MADRS) score of ≤13 during four consecutive visits. The site obtained approval from the sponsor to continue these 4 subjects in the open-label stabilization phase up to 18 weeks.

DSI suggests the Review Division should consider excluding the subjects who did not meet all eligibility criteria and reanalyze the data to see any impact on study outcome. The Review Division should include the non-reported AEs in safety database. There were multiple instances of protocol required clinical laboratory tests, prolactin levels, pregnancy tests and drug screens that were not performed at Dr. Rosales site. Although it is less likely to have major impact on adequacy of safety data, the review division should note this issue of missing laboratory data. Otherwise, data from these centers that had been inspected appear acceptable for use in support of this NDA.

Ni A. Khin, M.D., Branch Chief
Good Clinical Practice Branch I, HFD-46
Division of Scientific Investigations

CONCURRENCE:

Joseph Salewski
Deputy Director
Division of Scientific Investigations

Key to Classifications
NAI = No deviation from regulations. Data acceptable
VAI = Minor deviations(s) from regulations. Data acceptable
VAI-RR= Deviation(s) form regulations, response received and reviewed.
OAI = Significant deviations for regulations. Data unreliable
Pending = Inspection not completed

cc:
NDA 21-436/SE1-005
HFD-45/Division File / Reading File
HFD-45/Program Management Staff (electronic copy)
HFD-46/Khin
HFD-46/GCPB1 Files

rd:NK:10/22-25/04

O:\NK\CIS\NDA21436SE1005 arip Bipolar Maintenance CIS.doc

This is a representation of an electronic record that was signed electronically and this page is the manifestation of the electronic signature.

/s/

Ni Aye Khin
11/3/04 06:15:43 PM
MEDICAL OFFICER

Joseph Salewski
11/8/04 09:05:26 AM
CSO

Bates, Doris J

From: Bates, Doris J
Sent: Friday, October 22, 2004 4:24 PM
To: 'Susan H Behling'
Cc: Podruchny, Teresa; 'kusumam@otsuka.com'; Andreason, Paul J
Subject: RE: Abilify N 21436 S005

Good afternoon Susan,

I have questions from our clinical reviewer, Dr. Podruchny. Please feel free to include her as a CC in any response via email (we will need a submission to the official file as well).

****

Please indicate where the narrative can be found for patient 138010-108-348 and please clarify the reason for discontinuation of patient 138010-146-459. The text on page 226 of 2220 notes that 146-459 discontinued secondary to elevated prolactin. However, the narrative for this patient on page 593 says that discontinuation was secondary to "moderate tremors, dizziness (lightheadedness), nervousness and nausea". We need clarification on this point.

****

Thanks as always, Susan.

Sincerely,

Doris J. Bates, Ph.D.
Regulatory Project Manager
Division of Neuropharmacological Drug Products
Office of Drug Evaluation I
Center for Drug Evaluation and Research

Tracking:

Recipient	Delivery
'Susan H Behling'	
Podruchny, Teresa	Delivered: 10/22/2004 4:24 PM
'kusumam@otsuka.com'	
Andreason, Paul J	Delivered: 10/22/2004 4:24 PM

This is a representation of an electronic record that was signed electronically and this page is the manifestation of the electronic signature.

/s/

Doris Bates
10/22/04 04:26:47 PM
CSO
sent to firm on date entered in DFS. See
email for time of transmittal.

Full thread attached.

-----Original Message-----
From: Charles D Wolleben [mailto:Charles.Wolleben@bms.com]
Sent: Tuesday, August 24, 2004 2:33 PM
To: Bates, Doris J; Podruchny, Teresa
Cc: Andreason, Paul J; Susan Behling; Mallikaarjun, Kusuma
Subject: Re: FW: NDA 21-436 S-002 and S-005: Urgent Question from Clinical Rev iewer

Doris/Dr Podruchny:

There were no Non-IND sites in -009 and -074 (all US).

Regarding -010, the following 5 sites were non-IND:
089 (Argentina)
091 (Argentina)
093 (Mexico)
111 (Argentina)
118 (Mexico).

Hope this helps. Call or email if this does not address your questions.

Chuck

Bates, Doris J wrote:

Hello Chuck, I received Susan's out of office email right after sending this, so am copying it to you as well.

Sincerely,

Doris J. Bates, Ph.D.
Regulatory Project Manager
Division of Neuropharmacological Drug Products
Office of Drug Evaluation I
Center for Drug Evaluation and Research

-----Original Message-----
From: Bates, Doris J
Sent: Tuesday, August 24, 2004 1:38 PM
To: 'Susan H Behling'; 'kusuma mallikaarjun'
Cc: Andreason, Paul J; Podruchny, Teresa; Bates, Doris J
Subject: RE: NDA 21-436 S-002 and S-005: Urgent Question from Clinical Reviewer

Dear Susan and Kusuma,

Our clinical reviewer has identified an urgent question related to both S-002 and S-005.

In the case of S-002, we will need a response as soon as possible because of the very limited time remaining in the review cycle for this

submission; please respond by COB a week from today. (Secure email is fine for this response.)

For S-005, we can wait a bit longer for your reply but would like the information by mid-September if possible. (Secure email is again fine.)

****
Please identify, by number, all of the non-IND sites

- in studies 009 and 074 for supplement 002

- in study 010 for supplement 005.

****

Thank you in advance; for S-002 especially, it will help if you can include Drs. Podruchny and Andreason as CC recipients on any e-mail responses (to minimize routing delays).

Very sincerely,

Doris J. Bates, Ph.D.
Regulatory Project Manager
Division of Neuropharmacological Drug Products
Office of Drug Evaluation I
Center for Drug Evaluation and Research

This is a representation of an electronic record that was signed electronically and this page is the manifestation of the electronic signature.

/s/

Doris Bates
8/24/04 04:16:54 PM
CSO

Bates, Doris J

**From:**	Susan H Behling [Susan.Behling@bms.com]
**`ent:**	Friday, April 16, 2004 5:36 PM
**'o:**	batesd@cder.fda.gov; podruchnyt@cder.fda.gov
**Cc:**	Charles D Wolleben; kusumam@otsuka.com
**Subject:**	S-005 NDA 21-436, 74 day Letter
**Importance:**	High

Ari_Response &2to74dayletter

Drs. Bates and Podruchny:

A response to the first and second request in the 74 day letter for this supplement is provided attached to this e-mail. We would like some further clarifications on the requests as indicated in the attachment. Primarily, if, after reviewing the response, you are interested in JMP datasets for the secondary variables, it would be very helpful to know which specific variables would be of interest.

Please note that the request for all appendices has been fulfilled (submitted on March 25, 2004). Please let us know if you are unable to locate them.

'e are in the process of compiling the information for the remaining items requested and will be providing those in a separate correspondence.

As I will be on vacation next week, please contact my colleague, Dr. Chuck Wolleben, at the above e-mail address, or by phone at 203-677-3834 for further communications on this matter or for any other requests related to this application.

Sincerely,

Sue

"MMS <cder.fda.gov>" made the following annotations.

This message was sent from Bristol-Myers Squibb, Co. across the Internet in encrypted format and was successfully decrypted, unless otherwise noted.
Bristol-Myers Squibb
===

Bullet #1: (Request for efficacy and demographics information for patients enrolled by end of Dec 2000)

The efficacy and demographics information for all 633 patients enrolled into study CN138-010 is contained in the individual SAS data sets for Study CN138-010, which have been provided previously.

The efficacy scale data (Y-MRS, MADRS, CGI, and PANSS scores) are contained in the data sets fda_seff.xpt, fda_meff.xpt, and fda_eeff.xpt, for the Stabilization, Maintenance, and Extension Phases, respectively. The demographics information and the variables required for the primary and key secondary efficacy analyses (indicators for relapse, relapse dates, and relapse types) are contained in the data set fda_dem.xpt.

One can select the patients enrolled between the beginning of enrollment and the end of December 2000 by selecting those patients with a consent date prior to January 1, 2001. The variable which contains the consent date is called CNSNTD and is in the data set fda_dem.xpt. The 35 patients who were randomized prior to January 1, 2001 (and excluded from the primary analysis due to the study medication labeling error) can be identified by the indicator variable INRANDB1 in the fda_dem.xpt data set. INRANDB1 has a value of 1 if the patient was randomized before January 1, 2001. Otherwise it has a value of 0.

Further details on the data sets, and the names and descriptions of the variables that they contain, can be found in the data definition tables located in the file define.pdf, which has been provided previously.

These files can be loaded into either PC SAS or SAS-JMP. Since these data sets contain a large number of variables they may not be convenient to view using SAS-JMP. If the reviewer specifies what variables are of primary interest we can provide versions of the data set that may be easier to view in SAS-JMP.

Bullet #2: (Request for safety data for "any of those patients randomized")

<u>CN138-010 Individual SAS Data Sets</u>

The safety data for all 633 patients enrolled into study CN138-010 is contained in the individual SAS data sets for Study CN138-010, which have been provided previously.

The EPS-rating scale data (AIMS, Barnes-Akathisia Scale, and SAS scores) are contained in the data sets fda_ssaf.xpt, fda_msaf.xpt, and fda_esaf.xpt, for the Stabilization, Maintenance, and Extension Phases, respectively. The adverse event data for all phases of the study are contained in the data set fda_qadr.xpt. The vital sign data are contained in the data sets fda_svit.xpt, fda_mvit.xpt, and fda_evit.xpt, for the Stabilization, Maintenance, and Extension Phases, respectively. The electrocardiogram data are contained in the data sets fda_secg.xpt, fda_mecg.xpt, and fda_eecg.xpt, for the Stabilization, Maintenance, and Extension Phases, respectively. The laboratory data are

contained in the data sets fda_slab.xpt, fda_mlab.xpt, and fda_elab.xpt, for the Stabilization, Maintenance, and Extension Phases, respectively.

The 35 patients who were randomized prior to January 1, 2001 can be identified by the indicator variable INRANDB1 in the fda_dem.xpt data set. INRANDB1 has a value of 1 if the patient was randomized before January 1, 2001. Otherwise it has a value of 0. The 161 patients who were randomized after January 1, 2001 can be identified by the indicator variable INRAND in the fda_dem.xpt data set. INRAND has a value of 1 if the patient was randomized after January 1, 2001. Otherwise it has a value of 0. The fda_dem.xpt data set would need to be merged into the data set of interest (merging by the variable UNIQ_ID) to subset the desired safety data for the these patients.

Composite Safety Data Set "Merged" Files Structured for Viewing in SAS-JMP

The safety and demographics data for all patients enrolled in CN138-010 (including those who were randomized into the maintenance phase) are included in (.xpt) data sets structured for viewing in SAS-JMP. These tables combine AE, Previous/Concomitant Medication, and demographic data with either lab, ECG or vital sign data. The list below provides the names of the data sets:

Vital Signs/AE/Prev.&Concom Meds/Demographics	MVITBIPO
ECG/AE/Prev.&Concom Meds/Demographics	MECGBIPO
Labs - split into four data sets due to size, based on tests	
Hematology/AE/Prev.&Concom Meds/Demographics	MHEMBIPO
Chemistry/AE/Prev.&Concom Meds/Demographics	MCHMBIPO
Electrolytes/AE/Prev.&Concom Meds/Demographics	MELEBIPO
Metabolics/AE/Prev.&Concom Meds/Demographics	MMETBIPO

The data definition tables for these data sets provide more detailed information. These data sets actually contain data for all the patients in the Bipolar Mania studies. To select only those patients enrolled in CN138-010 use the Rows > Row Selection > Select Where command sequence and in the Row Selection window click on the variable UNIQ_ID2 choose 'contains' and type '138010' in the value box and click OK. This will select all rows of data from patients in CN138-010. To create a separate table for these patients use the Tables > Subset command sequence. Be sure the 'Selected Rows' Row Option is selected and click ok. This will copy all the rows of data for these patients in a new table. A similar sequence of commands can be done to create a separate table for a given patients just use the 'equals' in the row selection window and type in the specific patients unique id from CN138-010 instead of '138010'. Since these files were originally designed for review of the overall safety of aripiprazole, there is not a way to select out the group of patients randomized in the maintenance phase. If this were needed we would be happy to provide a version of all 6 data sets with just those patients included.

DEPARTMENT OF HEALTH & HUMAN SERVICES Public Health Service

Food and Drug Administration
Rockville, MD 20857

**SUPPLEMENTAL NDA ACKNOWLEDGED/FILED:
FILING REVIEW ISSUES IDENTIFIED
(CLINICAL / STATISTICS)**

NDA 21-436 / S-005

Otsuka America Pharmaceutical Inc.
Attn: Dr. Kusuma Mallikaarjun
Director, Regulatory Affairs / Abilify™
2440 Research Boulevard
Rockville, Maryland 20850

Dear Dr. Mallikaarjun:

Please refer to your supplemental new drug application (sNDA), referenced above, which was submitted on March 25, 2004 and received on March 26, 2004 under section 505(b) of the Federal Food, Drug, and Cosmetic Act for ABILIFY (aripiprazole) Tablets.

The supplemental application provides for the use of aripiprazole monotherapy in maintaining stability in patients with Bipolar I Disorder.

We have completed our filing review for the supplemental application and have determined that your application is sufficiently complete to permit a substantive review. As you were informed via secure e-mail, this application has been filed on March 31, 2004 under section 505(b) of the Act and in accordance with 21 CFR 314.101(a). Our goal date for acting on the submission is November 30, 2004.

In our filing review, we have identified the following review issues:

Clinical
Please submit the following information:

- Please provide, preferably in JMP (.xpt) tables, the efficacy and demographics information on all patients enrolled between the beginning of enrollment and the end of December, 2000. The clinical reviewer has been unable to locate this information.
- Please provide the safety data in similar fashion for any of those patients randomized, or reference that this is contained within the safety dataset if it is. Please feel free to contact the clinical reviewer for clarification of this request as necessary.
- Please explain what the headers in the appendix tables mean, by providing a list for each table (unless all headers mean the same thing in all appendix tables) or reference where the reviewer can find this information. For example, please compare the headers on appendices 7.3A and 7.3B.
- Please provide information about electrolyte laboratory results (to include bicarbonate); the incidence of potentially clinically significant values and the criteria for these.
- Please provide Appendix 7.1- patient accrual tabulated by month.

- Please provide Appendix 8.3, noted as available upon request in the original submission.

Statistics

- Please submit the SAS codes used to produce the results found in Table 10A, Figure 8.1 and Figure S.10.1.

Please respond to the above requests for additional information as soon as possible. While we anticipate that any response submitted in a timely manner will be reviewed during this review cycle, such review decisions will be made on a case-by-case basis at the time of receipt of the submission.

Please also note that our filing review is only a preliminary evaluation of the application, and is not indicative of all deficiencies that may be identified during our review. Issues may be added, deleted, expanded upon, or modified as we review the application.

If you have any questions, please call Doris J. Bates, Regulatory Project Manager, at (301) 594-2850.

Sincerely,

{See appended electronic signature page}

Russell Katz, M.D.
Director
Division of Neuropharmacological Drug Products
Office of Drug Evaluation I
Center for Drug Evaluation and Research

This is a representation of an electronic record that was signed electronically and this page is the manifestation of the electronic signature.

/s/

Russell Katz
4/12/04 11:57:08 AM

DSI CONSULT: Request for Clinical Inspections

Date: April 2, 2004

To: Khin Maung U, M.D., HFD-46
Ni Aye Khin, M.D., HFD-47

Through: Joanne L. Rhoads, M.D., M.P.H., Director, HFD-45

From: Russell G. Katz, M.D., Division Director, HFD-120
Paul Andreason, M.D... Team Leader, HFD-120
Doris J. Bates, Ph.D., Regulatory Project Manager, HFD-120
(Please see electronic signature page)

Subject: **Request for Clinical Inspections**
NDA 21-436 / SE1-005
Otsuka America Pharmaceutical, Inc.
ABILIFY (aripiprazole) Tablets

Protocol/Site Identification:

As recently discussed with you, the following protocols/sites essential for approval of the subject NDAs have been identified for inspection. These sites are listed in order of priority.

Please note that the third site listed is optional, and may be inspected at the discretion of DSI.

Indication	NDA and Site #	Site (Name and Address)	Number of Subjects
Maintenance of Stability in the Treatment of Bipolar I Disorder	NDA 21-436 Site 93	Ignacio Rosales, M.D.<br>San Rafael Clinic<br>Avenida Insurgentes Sur #4177<br>Col. Santa Ursula Xitle<br>Mexico City, Mexico CP 14420	18/13
See above	NDA 21-436 Site #64	Tram K. Tran-Johnson, Pharm.D.,<br>California Neuropsychopharmacology Clinical Research<br>9466 Black Mountain Road<br>Suite 100<br>San Diego, CA 92126.	TBD
See above	NDA 21-436 Site # 118	Miguel Angel Herrera Estrella, MD<br>Fray Bernardino Alvarez Hospital<br>Nino Jesus #2 Col. Tlalpan<br>Mexico City, Mexico C.P. 14000	25/17

Note: International inspection requests or requests for five or more inspections require sign-off by the ORM Division Director and forwarding through the Director, DSI.

Goal Date for Completion:

We request that the inspections be performed and the Inspection Summary Results be provided by (inspection summary goal date) **October 11, 2004**. We intend to issue an action letter on this application by (action goal date) **November 30, 2004**. We are willing to accept a draft of the Inspection Summary Results, in either hard copy or e-mail format, for the October 2004 request date.

Should you require any additional information, please contact Doris J. Bates, Ph.D. at 301-594-5536 or via e-mail at batesd@cder.fda.gov.

Previously provided information (hard copy): List of investigators and sites, NDA 21-436

Appears This Way
On Original

This is a representation of an electronic record that was signed electronically and this page is the manifestation of the electronic signature.

/s/

Doris Bates
4/6/04 04:21:19 PM

Paul Andreason
4/7/04 01:44:34 PM

Russell Katz
4/8/04 09:22:15 AM

Bates, Doris J

From: Bates, Doris J
Sent: Tuesday, March 30, 2004 2:49 PM
To: 'Susan H Behling'
Cc: 'Mallikaarjun, Kusuma'
Subject: RE: NDA 21-436, S-005: The Supplement Has Been Filed

Good afternoon Ms. Behling and Dr. Mallikaarjun,

This email is to confirm that the Division met briefly today and agreed that S-005 to NDA 21-436 is fileable as amended with the statistical appendices received last Friday.

The filing date is today, based on the original receipt date for the submission. (Submission date January 28, 2004; receipt date January 30, 2004.) The submission has therefore been filed as of this date. You may cite this email as an official communication of this decision from the Division.

We will also be sending you a 74-day letter on or before April 13, 2004, which will confirm this information and will include any review questions (not fileability issues) that have arisen in this interval. I will send you a copy of this letter via secure e-mail as soon as it is officially signed.

Our reviewers have expressed their appreciation of your firms' willingness to assist them in navigating through the submission, and will be in touch with any questions of that type.

Sincerely,

Doris J. Bates, Ph.D.
Regulatory Project Manager
Division of Neuropharmacological Drug Products
Office of Drug Evaluation I
Center for Drug Evaluation and Research

This is a representation of an electronic record that was signed electronically and this page is the manifestation of the electronic signature.

/s/

Doris Bates
3/30/04 02:48:05 PM
CSO

Bates, Doris J

From: Bates, Doris J
Sent: Tuesday, February 03, 2004 3:11 PM
To: 'Susan H Behling'
Cc: Bates, Doris J
Subject: RE: aripiprazole bipolar: Minutes from December 5, 2003 Meeting

Good afternoon Sue --

I've discussed our December 5 meeting with Dr. Andreason in light of the proposed plan for the safety update to (newly submitted) S-005; his response follows.

> During the meeting, the Division indicated that if BMS submits the supplement as proposed, it could not go directly to approval in the first review cycle.
>
> The reason for this is that there would be safety data on 900 people that we would know existed, but that would not be available to us within the PDUFA mandated review time frame for the new submission (under the BMS proposed submission date). Safety data on 900 patients, for a drug that has limited market experience, is a significant amount; indeed, it is significant enough that we would not be able to approve the submission without the opportunity to review this additional data.
>
> If these data were not included within a safety update that arrived early enough for us to review, then we would have to take an Approvable action in the first cycle, so that we would have a chance to see the data as part of the Complete Response.
>
> However, this does not mean that we would refuse to file this submission based on this potential deficiency alone. It merely means that such a submission would not, on face, be approved within the first review cycle, due to lack of the additional safety data as described.

Doris J. Bates, Ph.D.
Regulatory Project Manager
Division of Neuropharmacological Drug Products
Office of Drug Evaluation I
Center for Drug Evaluation and Research

-----Original Message-----
From: Susan H Behling [mailto:Susan.Behling@bms.com]
Sent: Thursday, January 29, 2004 11:38 AM
To: Bates, Doris J
Subject: Re: aripiprazole bipolar: Minutes from December 5, 2003

Meeting

Thanks Doris-We will review and get back to you. At first glance I
am a
bit concerned that the
minutes do not reflect the discussion of our proposal to combine the
requirements for a 120 day
safety update for the maintenance SNDA with the 'final' safety update
requirement for S-002[ ] b(4)
and that a Jan 8 cutoff for data for would be appropriate. We are
currently working under this
premise and if there is any issue with that proposal we would need to
know ASAP.

The maintenance filing will be delivered by courier tomorrow (1/30).
Dr. Andreason's requested
algorithms are contained in the CRT subfolder called 'merged dataset'
and is described in an
Appendix in the Definition document.

Best regards,

Sue

"Bates, Doris J" wrote:

> I am attaching the Division's minutes from our December 5, 2003
meeting -
> and my sincere apologies for our delay in issuing them. Please let
me
know
> if you have any questions related to these minutes,
>
> Doris J. Bates, Ph.D.
> Regulatory Project Manager
> Division of Neuropharmacological Drug Products
> Office of Drug Evaluation I
> Center for Drug Evaluation and Research
>
> "WorldSecure <wmghpwwsecp01.hpw.stf.bms.com>" made the following
> annotations on 01/28/04 16:36:17
>

>
> [INFO] -- Access Manager:
> This message was sent from CDER in an encrypted format, and was
decrtyped by BMS mail servers.
>
>

```
=================================================================
>
>    ------------------------------------------------------------
>                                               Name: Minutes for
December 5 mtg DFS.pdf
>    Minutes for December 5 mtg DFS.pdf         Type: Acrobat
(application/pdf)
>                                           Encoding: base64
>                                    Download Status: Not
downloaded
with message
```

"MMS <cder.fda.gov>" made the following annotations.

This message was sent from Bristol-Myers Squibb, Co. across the Internet
in encrypted format and was successfully decrypted, unless otherwise noted. Bristol-Myers Squibb

===

APPEARS THIS WAY
ON ORIGINAL

This is a representation of an electronic record that was signed electronically and this page is the manifestation of the electronic signature.

/s/

Doris Bates
2/3/04 03:20:12 PM
CSO
See date and time on e-mail for actual date
and time of transmission to firm.

IND 42,776 / NDA 21-436
Aripiprazole (Bipolar Mania)
Minutes of PreSNDA Meeting (Otsuka America / Bristol-Myers Squibb)
December 5, 2003

Participants: *FDA:* R. Katz, T. Laughren, P. Andreason, T. Podruchny, K. Jin, Y.-F. Chen, D. Bates
Otsuka America: W. Carson, T. Iwamoto, K. Mallikaarjun
BMS: D. Archibald, S. Behling, F. Fiedorek, R. Sanchez, E. Stock, R. Wolgemuth, C. Wolleben

Background: Aripiprazole has been approved in the treatment of schizophrenia (NDA 21-436) and is under review for the acute treatment of bipolar mania (S-002). [ ] **b(4)**

This pre-sNDA meeting was held to discuss:
1. the content and format of an additional sNDA for aripiprazole in maintenance treatment of Bipolar I patients, and

[ ] **b(4)**
The maintenance supplement will be submitted in late 2003 – early 2004.

Discussion:
BIPOLAR MAINTENANCE
1) *Does study CN138010 [6 to 18 week open label stabilization on aripiprazole, followed by 26-week randomization to aripiprazole or placebo] support an sNDA for maintenance treatment of bipolar disorder?*

FDA Comment:
- The study design will support some additional language in labeling, but FDA's concern is with the open-label phase of the study, rather than the randomized withdrawal phase. The open-label phase is considered to define the duration of effect for this (and similarly designed) study(ies).
- Based on the above feedback, the Division suggested an optimal study design (for future purposes) would consist of a six month open-label stabilization phase and randomized withdrawal of patient subgroups at specified times subsequent to this (e.g. 3 months, 6 months, etc.).

2) *Does the Division agree that the proposed indication may be feasible given the design and outcome of Study CN138010?*

FDA Comment: see discussion above.

3) *Does the Division agree to accept an electronic-only sNDA containing the final study report for CN138010, an ISS, labeling, CRTs, CRFs, updated literature review and administrative documents?*

FDA Comment:
- More hyperlinking was requested, as this makes it easier for the reviewer to navigate through the submission. E.g., if the text mentions a table, a hyperlink to the table would be helpful.
- Dr. Andreason requested that all deaths, discontinuations due to SAEs, and SAEs be in one place within the submission.

- BMS agreed to explore these options. [Post meeting note: BMS will add hyperlinks within the study report and the ISS and proposes to add supplemental tables to these documents to group deaths, discontinuations from SAEs, and SAEs as requested.]

4) *The maintenance sNDA is planned for submission during [late 2003 – early 2004] during the review of pending supplements S-002 [ ] BMS proposes to include CRFs for any new cases, as of June 30, 2003, that have been reported since the November 30, 2002 and February 7, 2003 cutoffs for the S-002 [ ] joint submission. This would include CRFs for all SAEs, discontinuations due to AEs, and deaths.* b(4)

<u>FDA Comment:</u>

- See above concerning grouping of information on deaths, discontinuations, and SAEs. FDA also indicated that narratives would be needed for deaths, discontinuations due to SAEs, and SAEs.
- Note that the pending supplement pair (S-002[ ] has not received a safety update since October 23, 2003; the timing of the proposed maintenance supplement could result in a situation where the acute safety data is truncated at June 30, 2003 for the new supplement's 120-day safety update, and no further updates can be reviewed in the time available for the older supplements (actions due April 25, 2004). b(4)
- BMS clarified that the next safety update would cover all bipolar studies; none would be ongoing. Presently, there are ca. 500 pts. in an open-label schizophrenia study, and ca. 360 in an open-label dementia study. Dr. Katz noted that the additional data on these patients (totaling nearly 900) would not be available to the Division under this scenario.
- Given the proposed submission timing and the difficulty of reviewing data submitted later than January for S-002 [ ] FDA noted that an approvable action on the first two supplements could be necessary to assure that all relevant safety information was received and reviewed for them. b(4)
- The ultimate submission date for the maintenance supplement will be decided subsequent to this meeting.

5) *This question relates to the timing of the safety update (SU) for the to-be-submitted bipolar supplement and its impact on the safety data for the bipolar supplements already pending. This includes BMS' proposal that the 120-day SU for the new supplement serve as the final SU for those already pending.*

<u>FDA Comment:</u>

- See above. The Division is uncomfortable about the prospect of approving the bipolar supplements without also seeing the safety data for the currently open studies in schizophrenia and dementia. Safety updates will be required, given the relative newness of this drug in the marketplace

6) *Does the Division have any other issues or concerns with the proposed dossier?*

<u>FDA Comment:</u>

- FDA inquired about the treatment of data from 35 patients who received unblinded medication during the randomized phase of the study. BMS clarified that the data from these patients was not included in the efficacy analysis. Re-randomization was not performed.
- The makeup of primary efficacy and primary safety databases was clarified: any patient who received drug was included in the primary safety database.

- Dr. Andreason further clarified FDA needs regarding patient data:
  - Group narratives by event, then treatment
  - Use unique patient identifiers
  - List date, visit number, and days post-randomization in each dataset
  - Provide an algorithm for assembling patient profiles, such that the reviewer can find all information pertinent to one patient efficiently.
  - Provide an explanation for patient disposition (e.g., why a patient withdrew from the study) (requested by Dr. Podruchny)

b(4)

1 Page(s) Withheld

X Trade Secret / Confidential (b4)

______ Draft Labeling (b4)

______ Draft Labeling (b5)

______ Deliberative Process (b5)

b(4)

POST MEETING NOTES:

- BMS is advised to obtain feedback on the User Fee implications for specific studies/submissions, to minimize the likelihood of post-submission unbundling and additional User Fees.]
- BMS provided Dr. Andreason with a proposed algorithm for construction of patient profiles, on December 18; this was discussed with Dr. Andreason in a December 23 teleconference and found acceptable, provided it can be run using JMP SAS.
- In this telecon, Dr. Andreason also requested that BMS provide clinical notes, CRF comments, etc. for patients with elevated LFTs, elevated glucose, and those with neutrophil counts below 500 within 60 days from the submission date.
- BMS is also working on the hyperlinking requested by the Division.

PLEASE SEE ELECTRONIC SIGNATURE PAGE
WHICH INCLUDES DR. KATZ' SIGNATURE TO CONFIRM ACCEPTANCE AND RELEASE OF MINUTES
AT THE DIVISION LEVEL.

Doris J. Bates, Ph.D.
Regulatory Project Manager

Paul Andreason, M.D.
Team Leader, Psychiatric Drugs Group II

This is a representation of an electronic record that was signed electronically and this page is the manifestation of the electronic signature.

/s/

Doris Bates
1/8/04 01:37:11 PM

Paul Andreason
1/12/04 10:13:18 AM

Russell Katz
1/26/04 08:24:32 AM
